# Supplementary material for: Optical deciphering of multinary chiral compound mixtures through organic reaction based chemometric chirality sensing
Source: Nat Commun. 2021 Nov 8;12:6451. doi: 10.1038/s41467-021-26874-9 (PMC8575934; doi:10.1038/s41467-021-26874-9)
Supplement: Supplementary file 1 — Supplementary Information [file 41467_2021_26874_MOESM1_ESM.pdf]

Supplementary Information

Optical Deciphering of Multinary Chiral Compound Mixtures Through Organic Reaction Based Chemometric Chirality Sensing

Diandra S. Hassan and Christian Wolf*

Department of Chemistry, Georgetown University, 37th and O Streets, Washington, DC 20057, USA. Email: cw27@georgetown.edu

Supplementary Methods

All commercially available reagents and solvents were used without further purification. Circular dichroism spectra were recorded on a JASCO J-710 spectropolarimeter. CD spectra were collected with a standard sensitivity of 100 mdeg, a data pitch of 0.5 nm, a band width of 0.5 nm, a scanning speed of 1000 nm min^{-1}, and a response of 0.5 s using a quartz cuvette (10 mm). The data were baseline corrected and smoothed using a binomial equation. UV-Vis spectra were recorded on a Cary 50 Bio spectrometer.

Chiroptical sensing using **A**

A solution of **A** (10.0 mM), a chiral amine or amino alcohol (10.0 mM) and Et_3N (10.0 mM) in 1.0 mL of dichloroethane was stirred overnight. The mixtures were diluted with 1.0 mL of dichloroethane and CD analysis was carried out by diluting 15 µL aliquots with 2.0 mL of dichloroethane or with 2.0 mL of methanol. UV analysis was carried out by diluting 10.0 µL aliquots with 3.0 mL of dichloroethane or with 3.0 mL of methanol.

Chiroptical sensing using **B**

A solution of **B** (10.0 mM), a chiral amine or amino alcohol (10.0 mM) and Et_3N (10.0 mM) in 1.0 mL of chloroform was stirred overnight. The mixtures were diluted with 3.0 mL of chloroform and CD analysis was carried out by diluting 20.0 µL aliquots with another 2.0 mL of chloroform. UV analysis was carried out by diluting 5.0 µL aliquots with 3.0 mL of chloroform.

Chiroptical sensing using **C**

A solution of **C** (10.0 mM), a chiral amine or amino alcohol (10.0 mM) and Et_3N (10.0 mM) in 1.0 mL of ACN was stirred overnight. The mixtures were diluted using 2.0 mL of ACN and CD analysis was carried out by diluting 25.0 µL aliquots with 2.0 mL of ACN. UV analysis was carried out by diluting 10.0 µL aliquots with 3.0 mL of ACN.

Chiroptical sensing using **D**

A solution of **D** (10.0 mM), a chiral amine or amino alcohol (10.0 mM) and Et_3N (10.0 mM) in 1.0 mL of DMSO was stirred overnight. The mixtures were diluted with 2.0 mL of DMSO and CD analysis was carried out by diluting 200.0 µL aliquots with 2.0 mL of DMSO. UV analysis was carried out by diluting 15.0 µL aliquots with 3.0 mL of DMSO.

Chiroptical sensing using **E**

A solution of **E** (10.0 mM), a chiral amine or amino alcohol (10.0 mM) and Et_3N (10.0 mM) in 1.0 mL of DMSO was stirred overnight. The mixtures were diluted with 2.0 mL of DMSO and CD analysis was carried out by diluting 200.0 µL aliquots with 2.0 mL of ACN. UV analysis was carried out by diluting 20.0 µL aliquots with 3.0 mL of ACN.

Chiroptical quaternary mixture sensing using **A**

Mixtures containing (*R*)-**PEA**, (*S*)-**PEA**, (*R*)-**PMP**, and (*S*)-**PMP** in varying enantiomeric ratios and 20.0 mM total concentration (equimolar amounts of PEA and PMP) were prepared from stock solutions in dichloroethane. To each solution, 21.0 mM of **A** and Et_3N were added and the

reaction mixture was stirred overnight. The solutions were then diluted using 2.0 mL of dichloroethane and CD analysis was carried out by diluting 40.0 µL aliquots in 3.0 mL of dichloroethane. UV analysis was carried out by diluting 20.0 µL aliquots in 3.0 mL of dichloroethane. See Supplementary Figures 18-32. Mixture sensing with A are shown in

Analysis of the CD sensing results with **PEA** and **PMP** showed that the coumarin product gives CD amplitudes that change linearly with the enantiomeric sample composition. **PEA-A** is CD silent at 400 nm while **PMP-A** is CD active. The CD response at this wavelength can be used to calculate the enantiomeric excess (*ee*) of **PMP**. Linear regression of the CD amplitude at 400 nm ($mdeg_{400\ nm}$) versus the *ee* of **PMP** ($ee_{\mathbf{PMP}}$) yields equation 1. The *ee* of **PEA** can be calculated by linear regression of the CD amplitude at 340 nm ($mdeg_{340\ nm}$), where only **PEA-A** contributes to the signal and **PMP-A** is CD silent. This yields equation 2.

Chemometric concentration and enantiomeric ratio quaternary mixture sensing using **A**

Mixtures containing varying concentrations and enantiomeric ratios of (*R*)-**PEA**, (*S*)-**PEA**, (*R*)-**PMP**, and (*S*)-**PMP** were prepared in dichloroethane. To these solutions, 200.0 µL of **A** (0.105 mM) and an equimolar amount of Et_3N were added and the reactions were stirred overnight. The mixtures were then diluted using 4.0 mL of dichloroethane and CD analysis was carried out by diluting 60 µL aliquots with 2.0 mL of dichloroethane. UV analysis was carried out by diluting 30 µL aliquots with 2.0 mL of dichloroethane. See Supplementary Figures 39-93.

Comprehensive octonary mixture sensing.

Mixtures containing (*R*)-**PEA**, (*S*)-**PEA**, (*R*)-**PMP**, (*S*)-**PMP**, (1*R*, 2*S*)-**PPA**, (1*S*, 2*R*)-**PPA**, (*R*)-**PGL**, and (*S*)-**PGL** in varying concentrations and enantiomeric ratios (at 20.0 mM total) were prepared from stock solutions in dichloroethane. To these samples, 21.0 mM of **A** and Et_3N were added and the reaction mixtures were stirred overnight. The solutions were then diluted using 7.0 mL of dichloroethane and CD analysis was carried out by diluting 30.0 µL aliquots in 2.0 mL of dichloroethane and 70.0 µL in 2.0 mL of methanol, respectively. UV analysis was carried out by diluting 20.0 µL aliquots in 2.0 mL of dichloroethane and 30.0 µL in 2.0 mL of methanol, respectively. See Supplementary Figures 98-199.

Supplementary Figures

Supplementary Figure 1. Structures of the target compounds

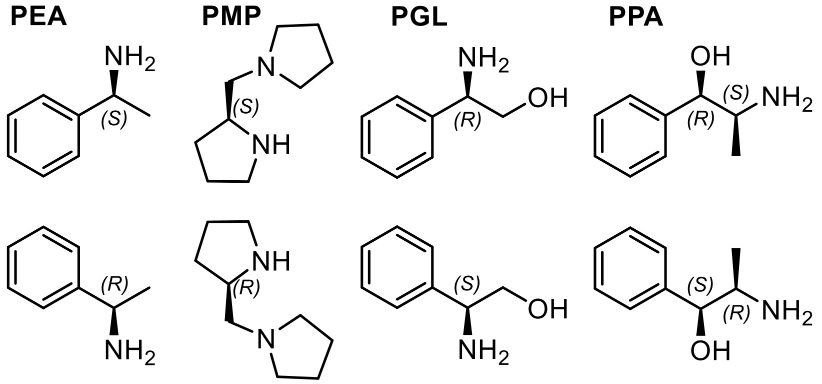

Supplementary Figure 2. CD spectra of the target compounds in the absence of a probe

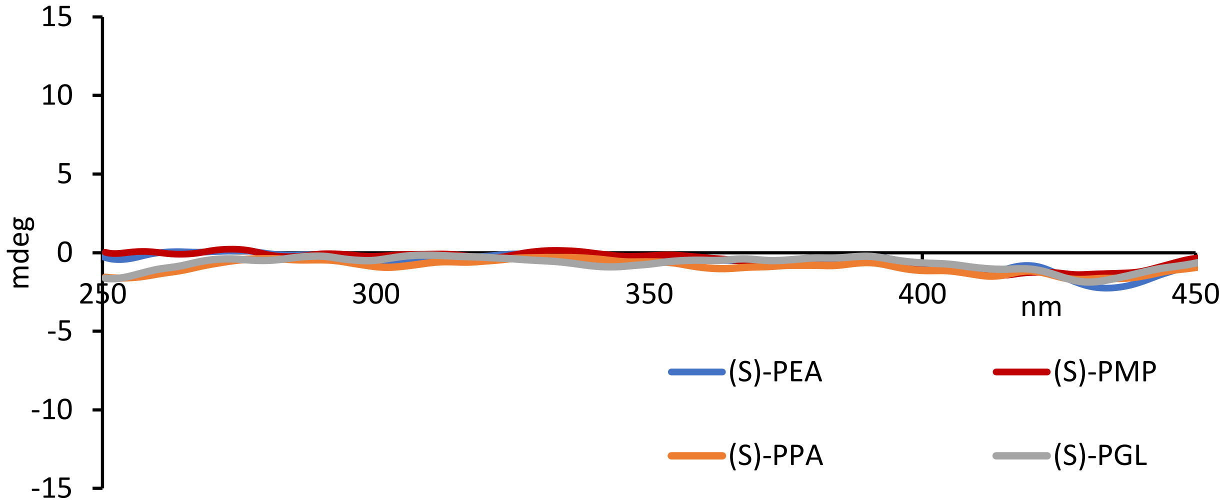

CD measurements were taken at 0.094 mM in dichloroethane.

Supplementary Figure 3. UV spectra of the target compounds in the absence of a probe

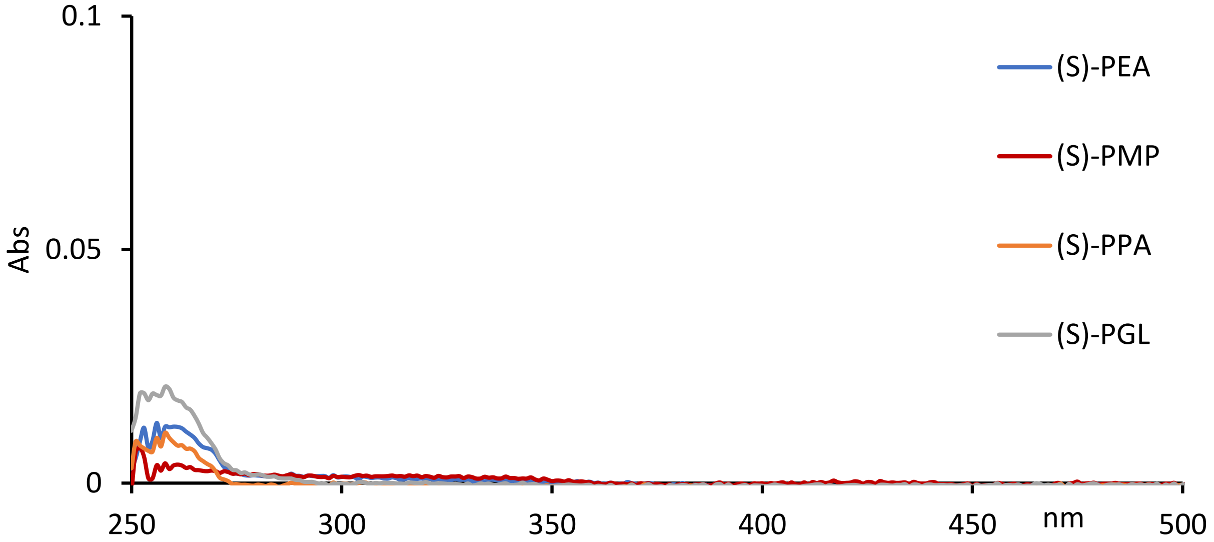

UV measurements were taken at 0.042 mM in dichloroethane.

Supplementary Figure 4. Structures of chiroptical probes **A**-**E**

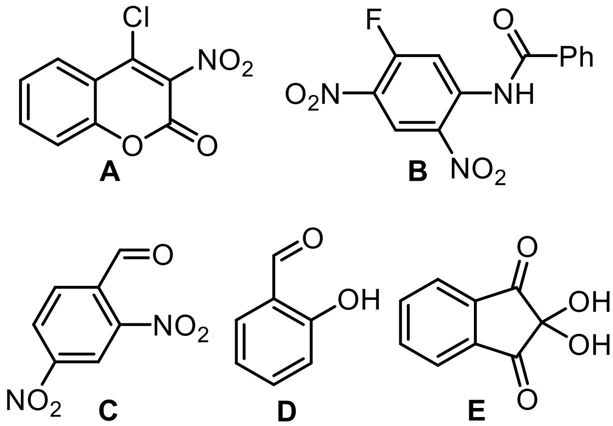

Supplementary Figure 5. UV spectra of the chiroptical probes

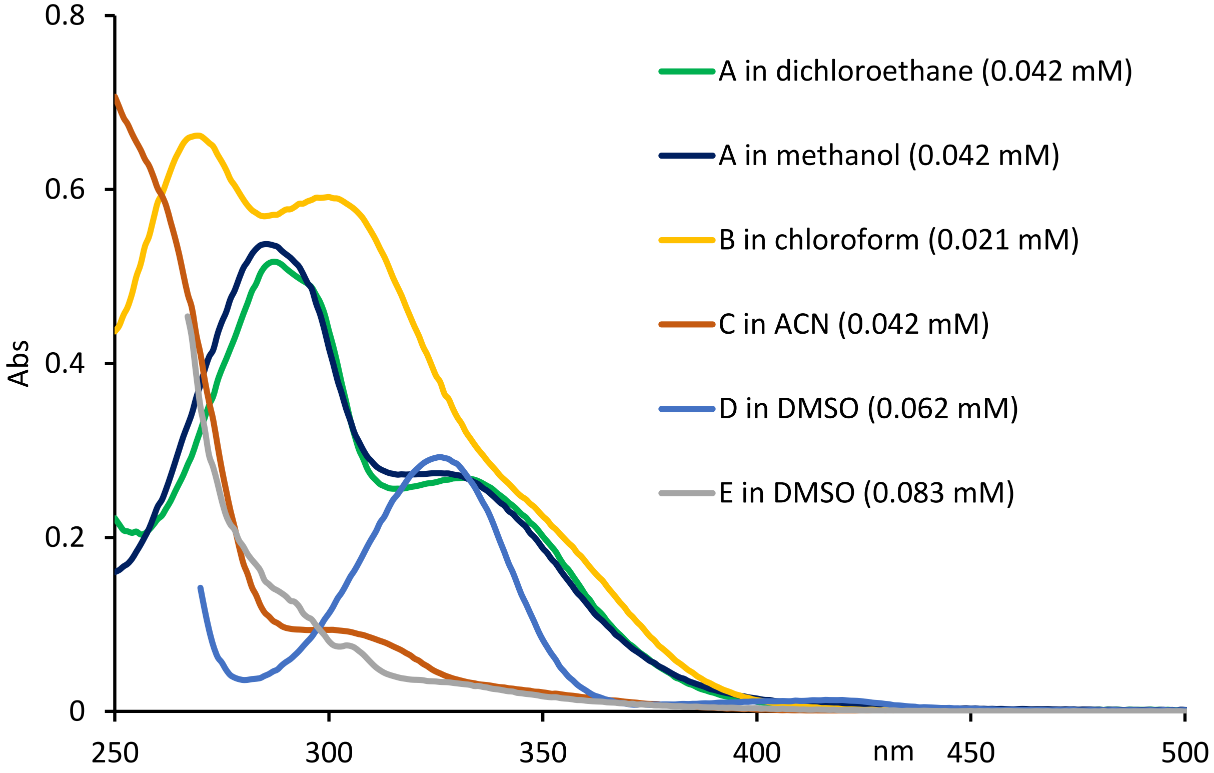

Supplementary Figure 6. CD spectra obtained by sensing of (*S*)-**PEA** (blue), (*S*)-**PMP** (red), (*S*)-**PPA** (orange), (*S*)-**PGL** (grey) with probe **A** in dichloroethane

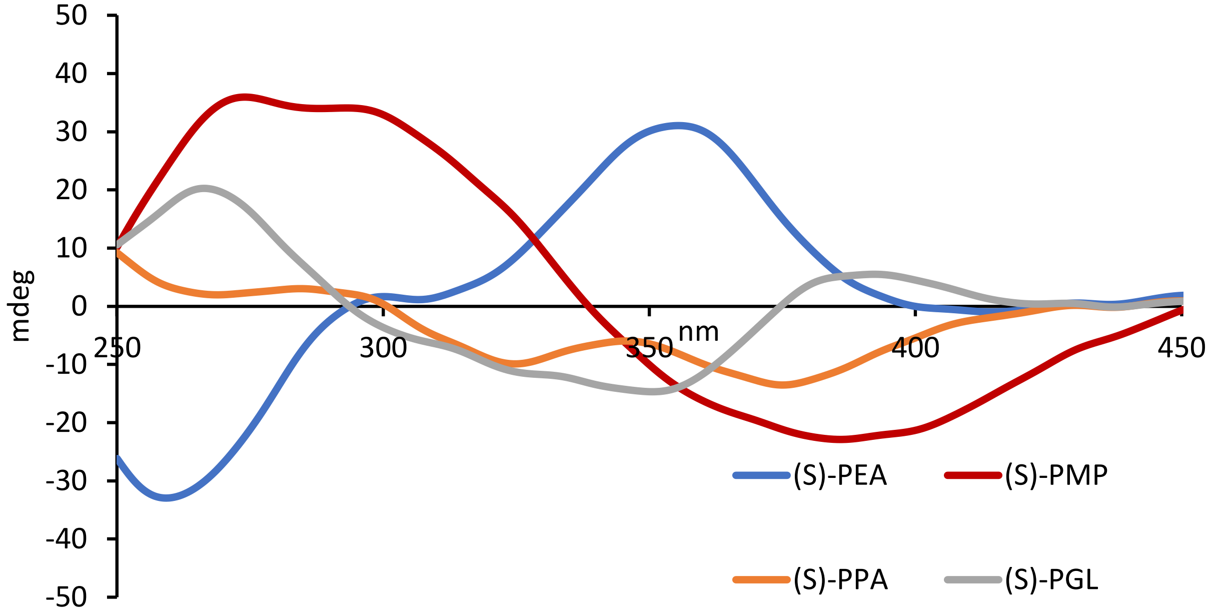

CD measurements were taken at 0.094 mM in dichloroethane.

Supplementary Figure 7. UV spectra obtained by sensing of (*S*)-**PEA** (blue), (*S*)-**PMP** (red), (*S*)-**PPA** (orange), (*S*)-**PGL** (grey) with probe **A** in dichloroethane

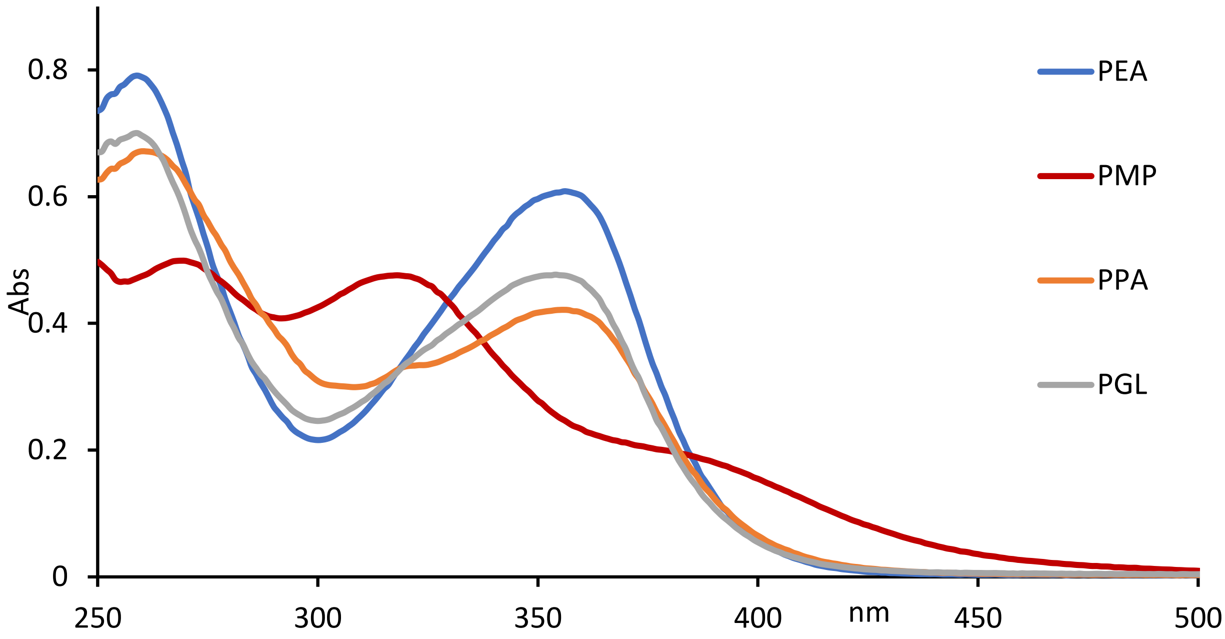

UV measurements were taken at 0.042 mM in dichloroethane.

Supplementary Figure 8. CD spectra obtained by sensing of (*S*)-**PEA** (blue), (*S*)-**PMP** (red), (*S*)-**PPA** (orange), (*S*)-**PGL** (grey) with probe **A** in methanol

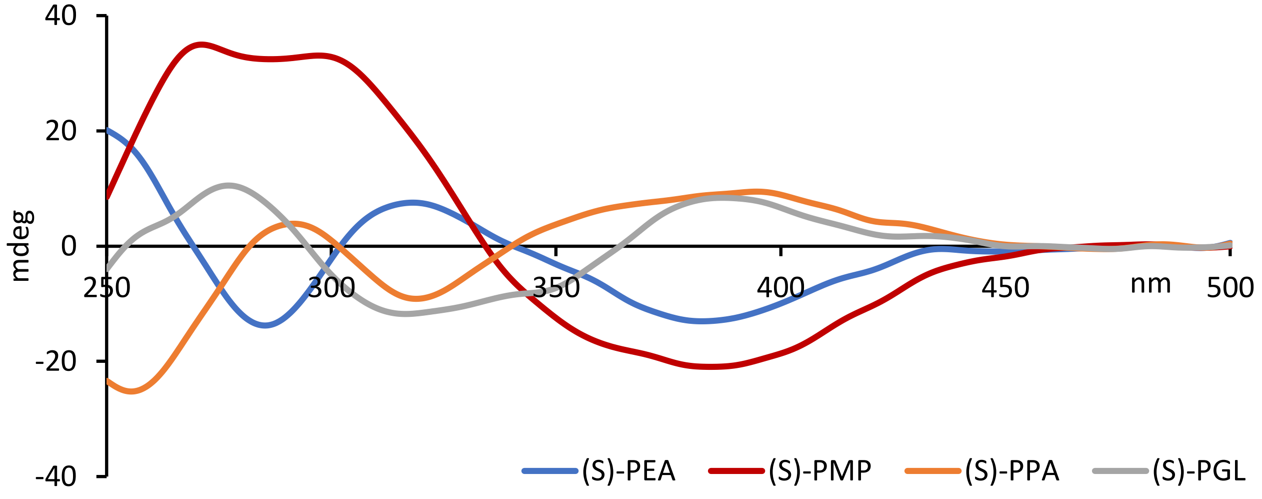

CD measurements were taken at 0.094 mM in methanol.

Supplementary Figure 9. UV spectra obtained by sensing of (*S*)-**PEA** (blue), (*S*)-**PMP** (red), (*S*)-**PPA** (orange), (*S*)-**PGL** (grey) with probe **A** in methanol

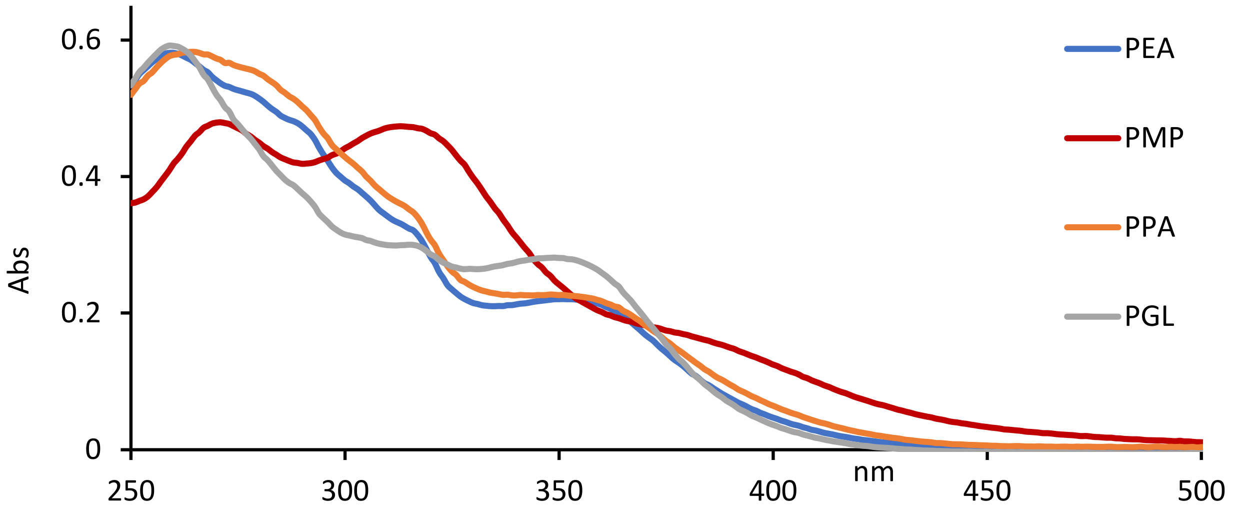

UV measurements were taken at 0.042 mM in methanol.

Supplementary Figure 10. CD spectra obtained by sensing of (*S*)-**PEA** (blue), (*S*)-**PMP** (red), (*S*)-**PPA** (orange), (*S*)-**PGL** (grey) with probe **B**

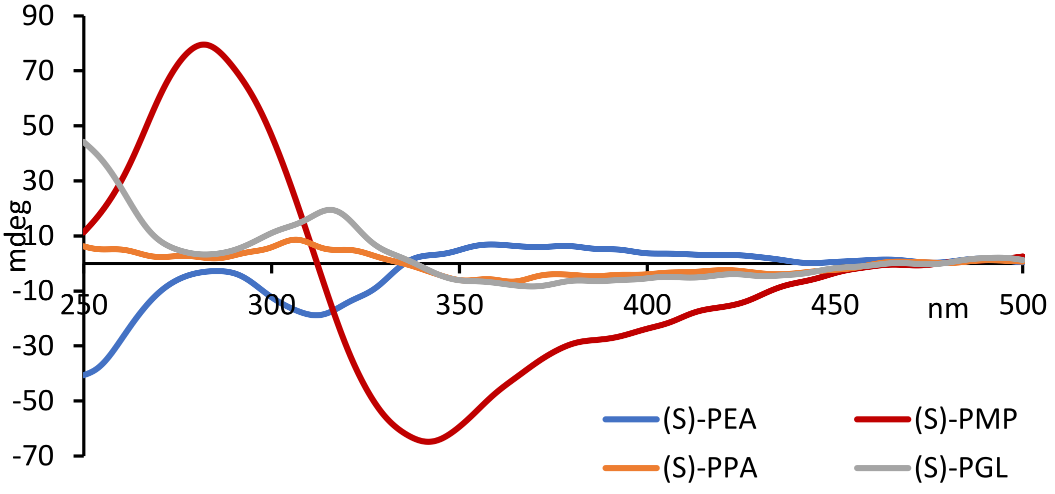

CD measurements were taken at 0.083 mM in chloroform.

Supplementary Figure 11. UV spectra obtained by sensing of (*S*)-**PEA** (blue), (*S*)-**PMP** (red), (*S*)-**PPA** (orange), (*S*)-**PGL** (grey) with probe **B**

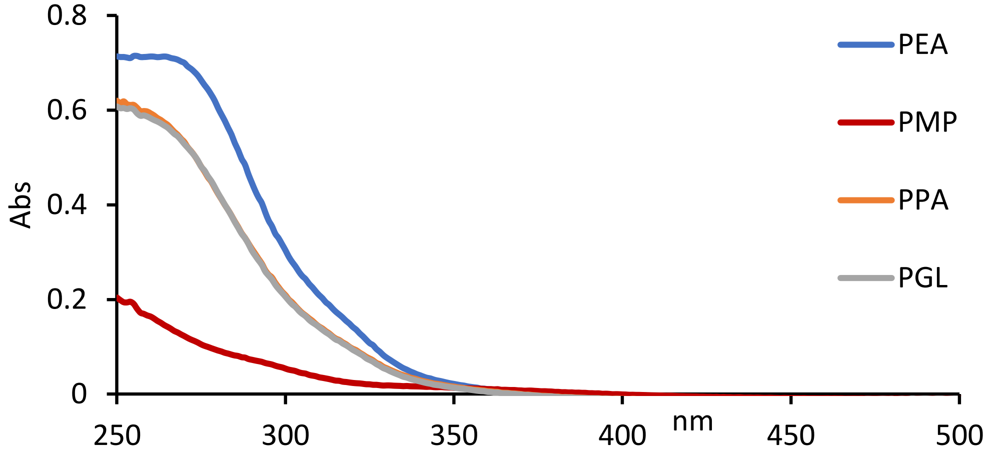

UV measurements were taken at 0.021 mM in chloroform.

Supplementary Figure 12. CD spectra obtained by sensing of (*S*)-**PEA** (blue), (*S*)-**PMP** (red), (*S*)-**PPA** (orange), (*S*)-**PGL** (grey) with probe **C**

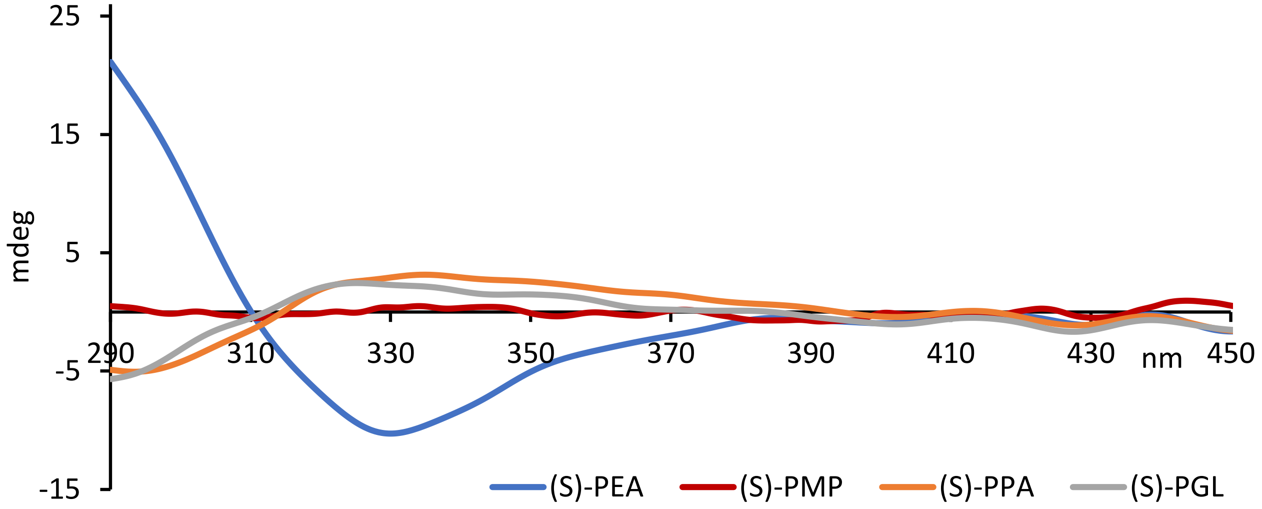

CD measurements were taken at 0.15 mM in acetonitrile.

Supplementary Figure 13. UV spectra obtained by sensing of (*S*)-**PEA** (blue), (*S*)-**PMP** (red), (*S*)-**PPA** (orange), (*S*)-**PGL** (grey) with probe **C**

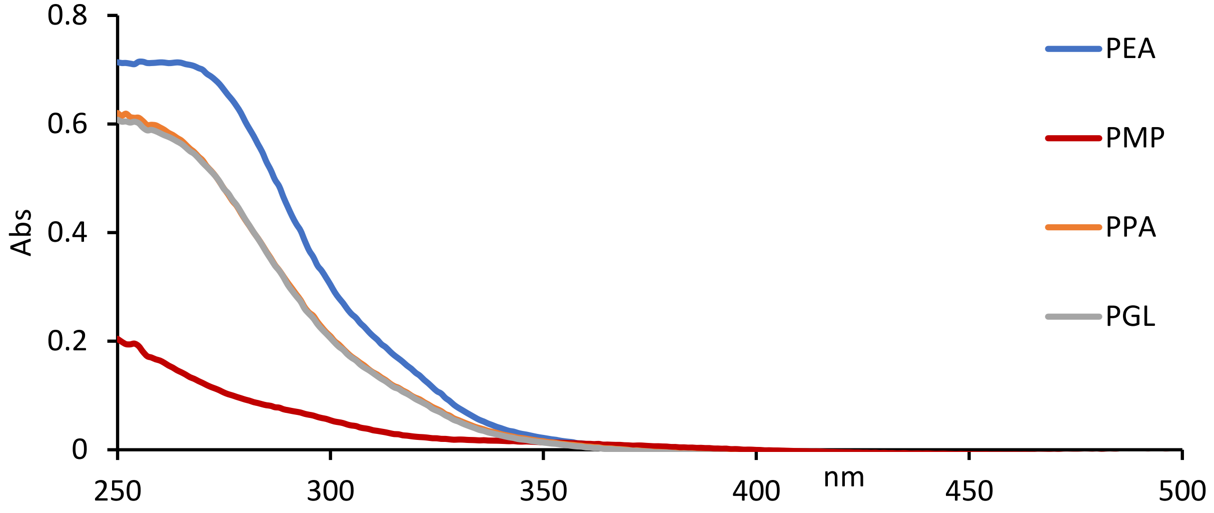

UV measurements were taken at 0.042 mM in acetonitrile.

Supplementary Figure 14. CD spectra obtained by sensing of (*S*)-**PEA** (blue), (*S*)-**PMP** (red), (*S*)-**PPA** (orange), (*S*)-**PGL** (grey) with probe **D**

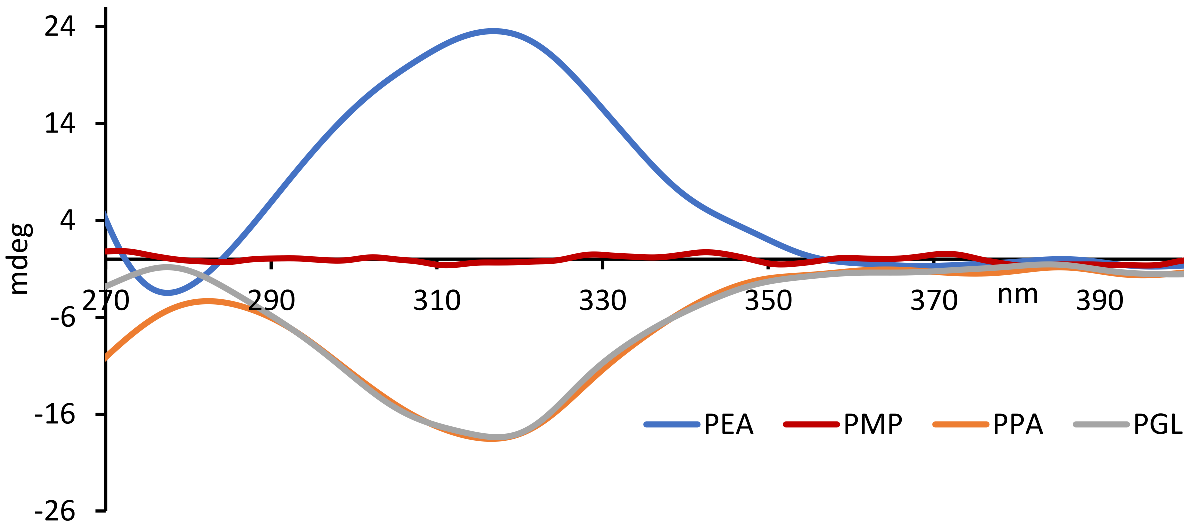

CD measurements were taken at 0.125 mM in DMSO.

Supplementary Figure 15. UV spectra obtained by sensing of (*S*)-**PEA** (blue), (*S*)-**PMP** (red), (*S*)-**PPA** (orange), (*S*)-**PGL** (grey) with probe **D**

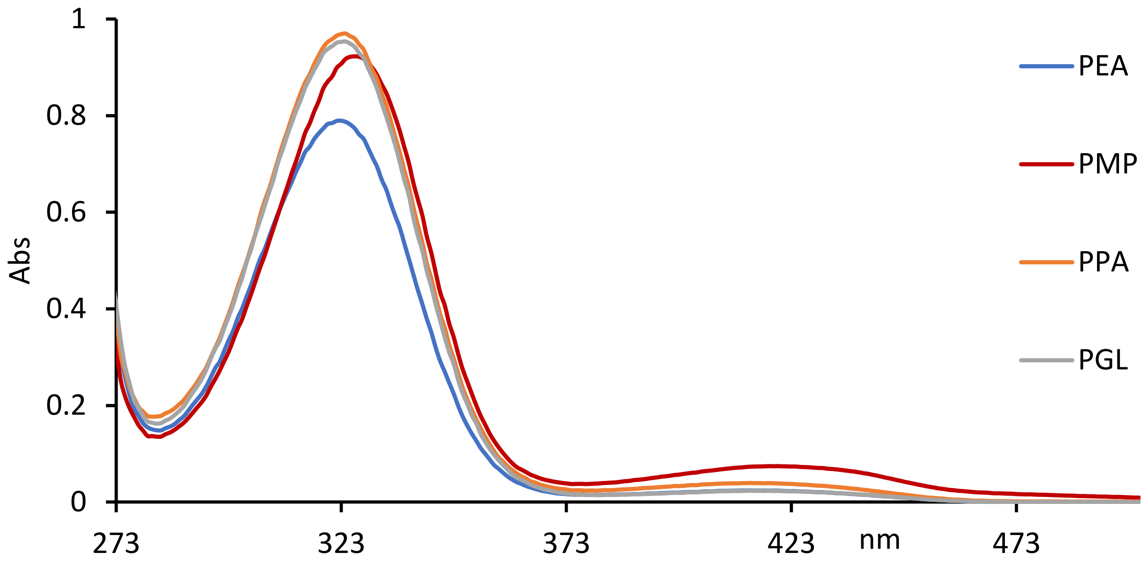

UV measurements were taken at 0.062 mM in DMSO.

Supplementary Figure 16. CD spectra obtained by sensing (*S*)-**PEA** (blue), (*S*)-**PMP** (red), (*S*)-**PPA** (orange), (*S*)-**PGL** (grey) with probe **E**

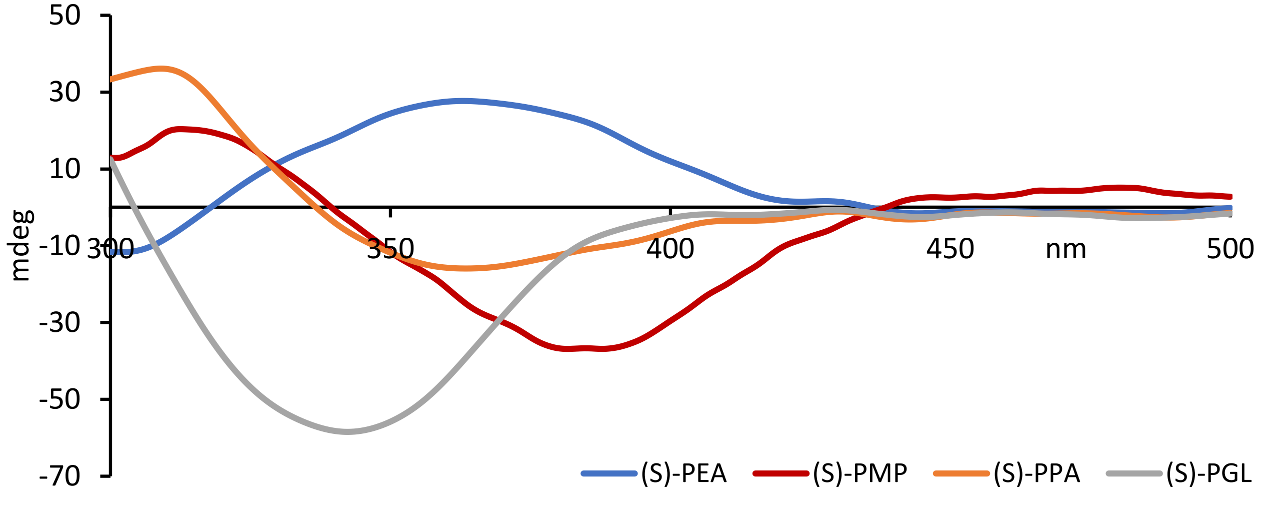

CD measurements were taken at 1.25 mM in acetonitrile.

Supplementary Figure 17. UV spectra obtained by sensing (*S*)-**PEA** (blue), (*S*)-**PMP** (red), (*S*)-**PPA** (orange), (*S*)-**PGL** (grey) with probe **E**

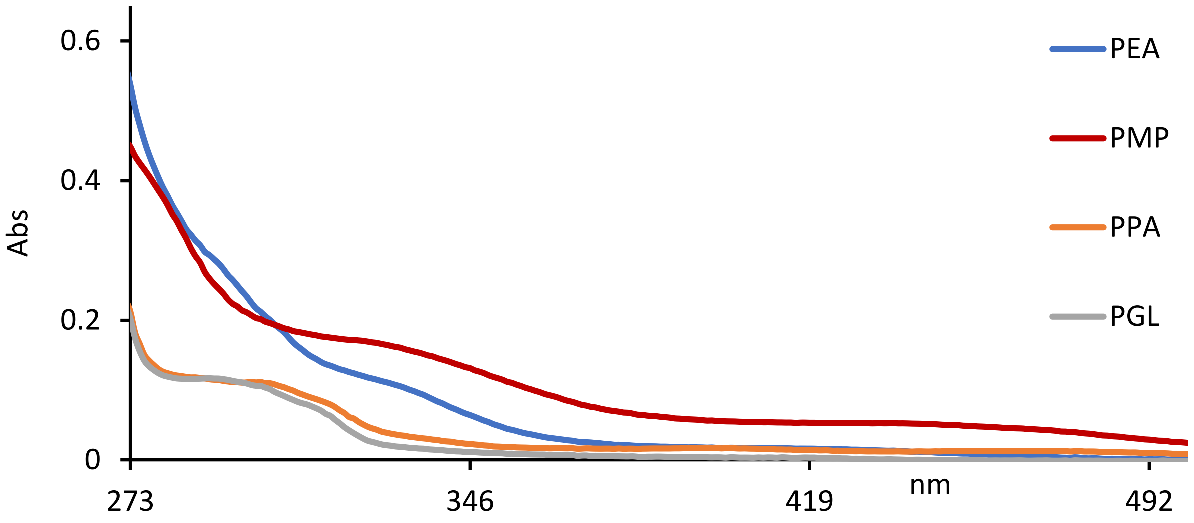

UV measurements were taken at 0.083 mM in acetonitrile.

Supplementary Figure 18. CD spectra obtained by sensing of (*R*)-**PEA** (dashed) and (*S*)-**PEA** (solid) with probe **A** in dichloroethane

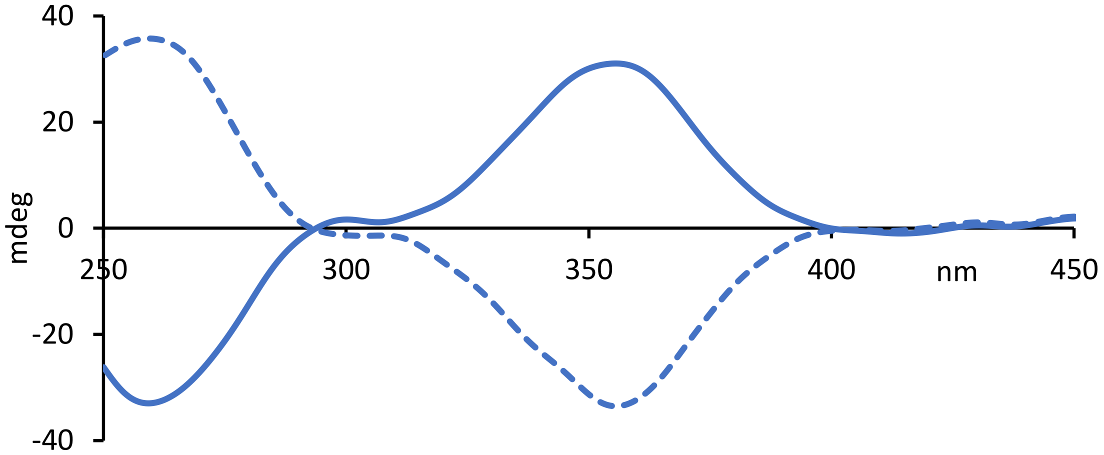

CD measurements were taken at 0.094 mM in dichloroethane.

Supplementary Figure 19. CD spectra obtained by sensing of (*R*)-**PMP** (dashed) and (*S*)-**PMP** (solid) with probe **A** in dichloroethane

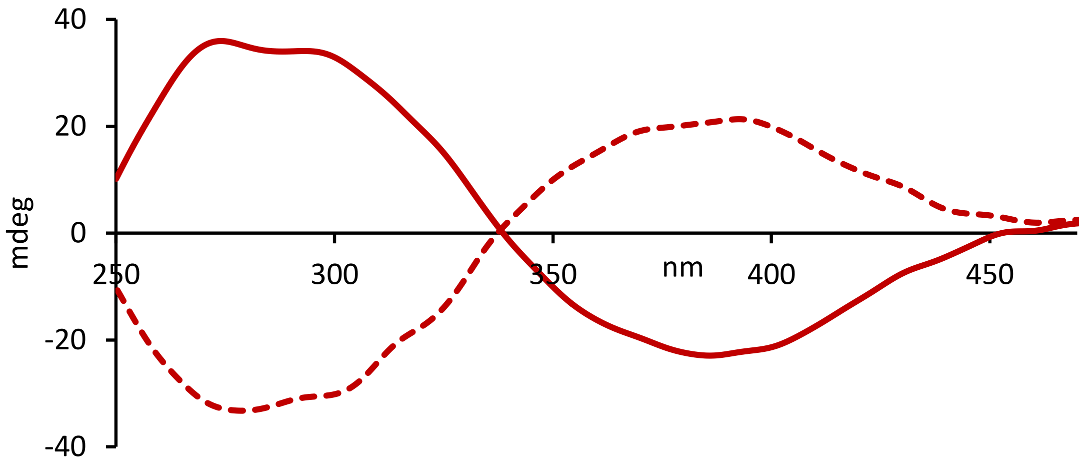

CD measurements were taken at 0.094 mM in dichloroethane.

Supplementary Figure 20. CD spectra obtained by sensing of (*R*)-**PPA** (dashed) and (*S*)-**PPA** (solid) with probe **A** in dichloroethane

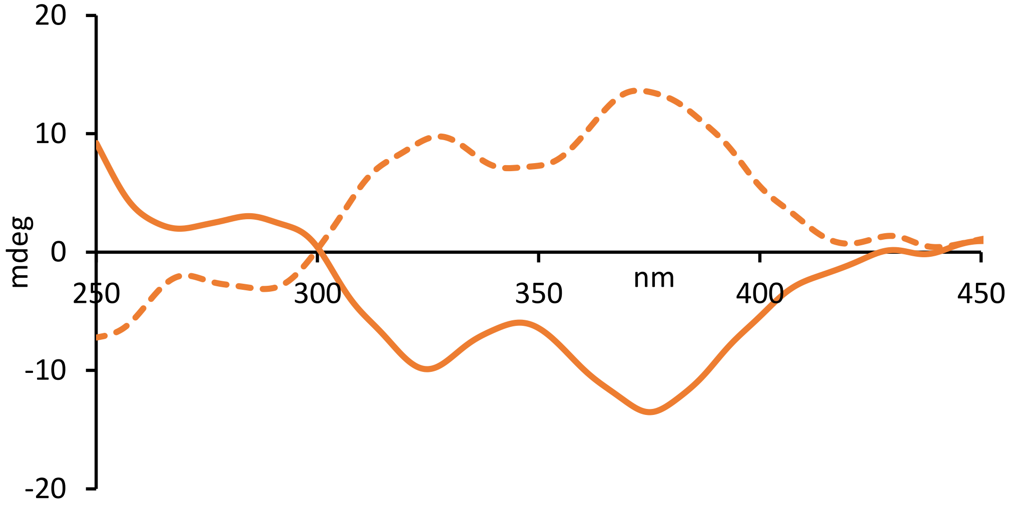

CD measurements were taken at 0.094 mM in dichloroethane.

Supplementary Figure 21. CD spectra obtained by sensing of (*R*)-**PGL** (dashed) and (*S*)-**PGL** (solid) with probe **A** in dichloroethane

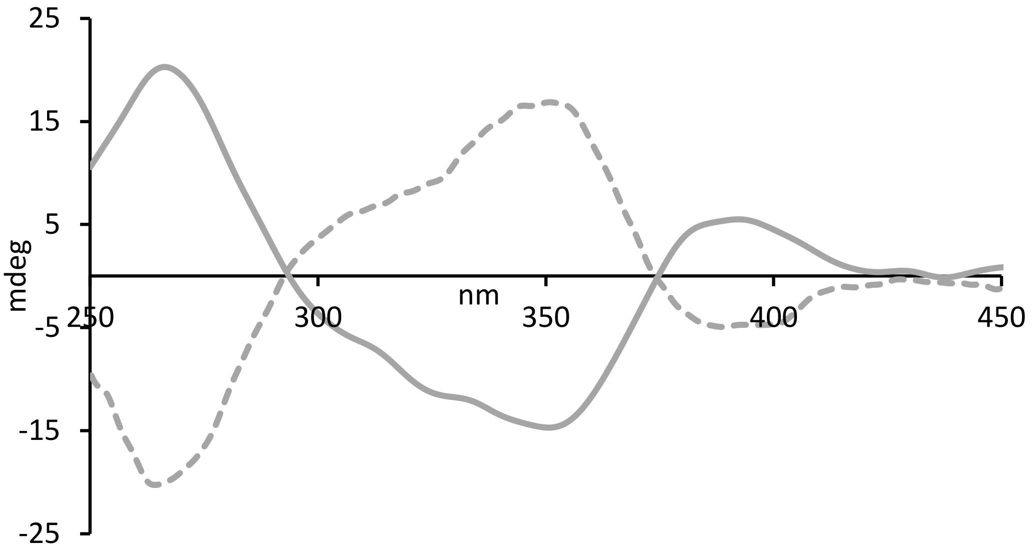

CD measurements were taken at 0.094 mM in dichloroethane.

Supplementary Figure 22. Comparison of CD spectra obtained by sensing of **PEA** (blue) and **PMP** (red) with probe **A** in dichloroethane

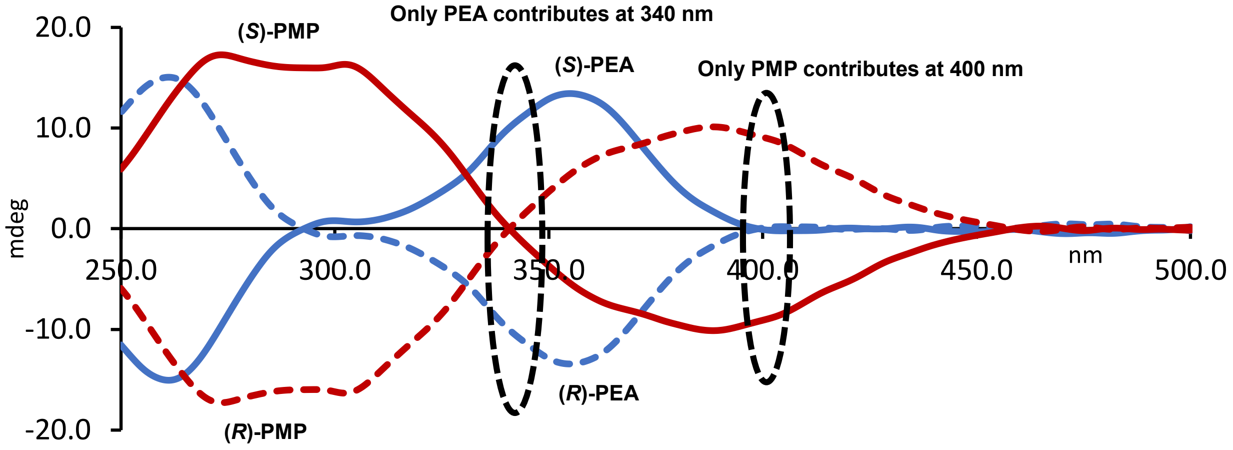

CD measurements were taken at 0.094 mM in dichloroethane.

Supplementary Figure 23. Entry 1: Mixture of 10.0 mM of (*R*)-**PEA** and 10.0 mM of (*R*)-**PMP**

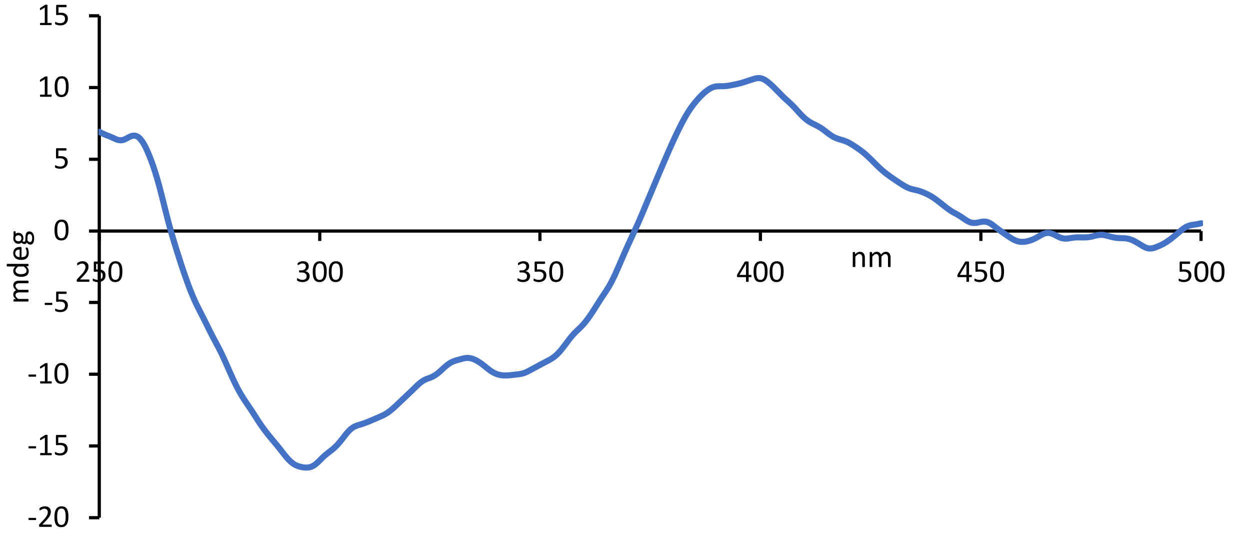

CD measurements were taken at 0.071 mM total analyte concentration in dichloroethane.

Supplementary Figure 24. Entry 2: Mixture of 10.0 mM of (*R*)-**PEA** and 10.0 mM of (*S*)-**PMP**

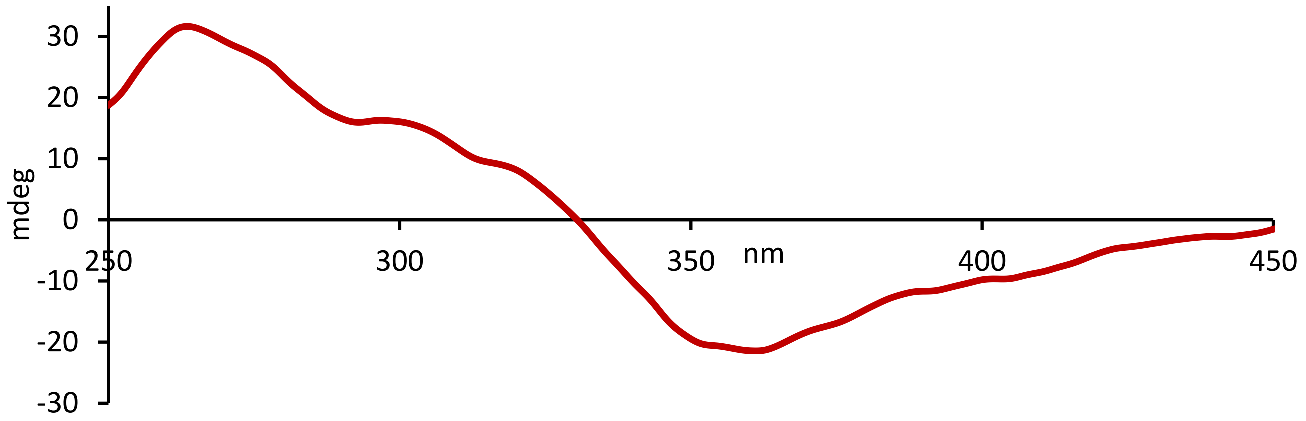

CD measurements were taken at 0.071 mM total analyte concentration in dichloroethane.

Supplementary Figure 25. Entry 3: Mixture of 10.0 mM of (*R*)-**PEA**, 7.0 mM of (*R*)-**PMP**, and 3.0 mM of (*S*)-**PMP**

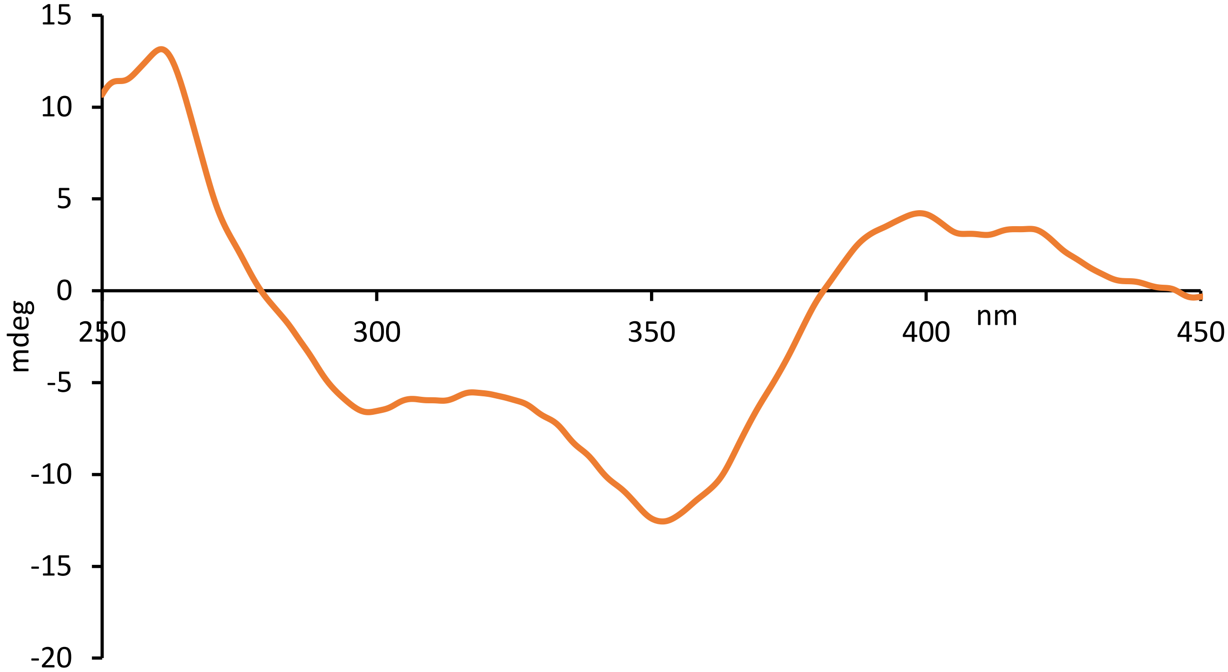

CD measurements were taken at 0.071 mM total analyte concentration in dichloroethane.

Supplementary Figure 26. Entry 4: Mixture of 10.0 mM of (*R*)-**PEA**, 7.0 mM of (*R*)-**PMP**, and 3.0 mM of (*S*)-**PMP**

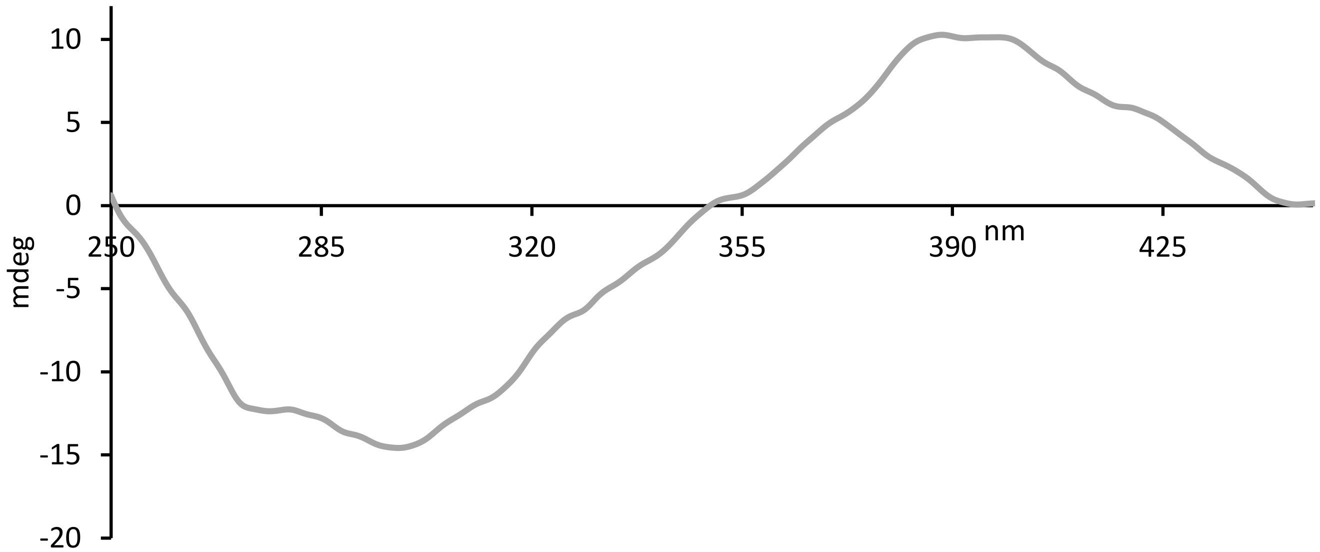

CD measurements were taken at 0.071 mM total analyte concentration in dichloroethane.

Supplementary Figure 27. Entry 5: Mixture of 8.0 mM of (*R*)-**PEA**, 2.0 mM of (*S*)-**PEA**, 2.0 mM of (*R*)-**PMP**, and 8.0 mM of (*S*)-**PMP**

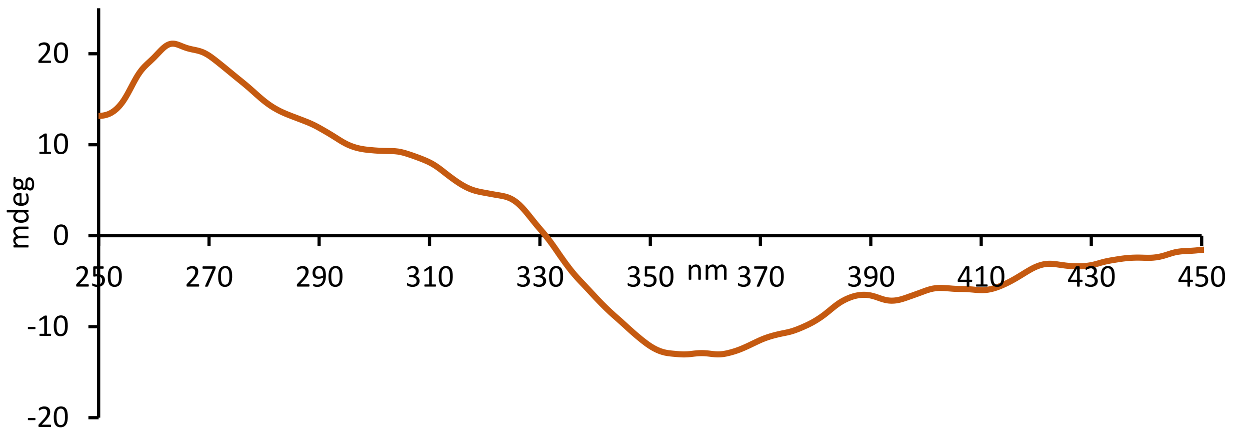

CD measurements were taken at 0.071 mM total analyte concentration in dichloroethane.

Supplementary Figure 28. Entry 6: Mixture of 10.0 mM (*S*)-**PEA**, 3.0 mM of (*R*)-**PMP**, and 7.0 mM of (*S*)-**PMP**

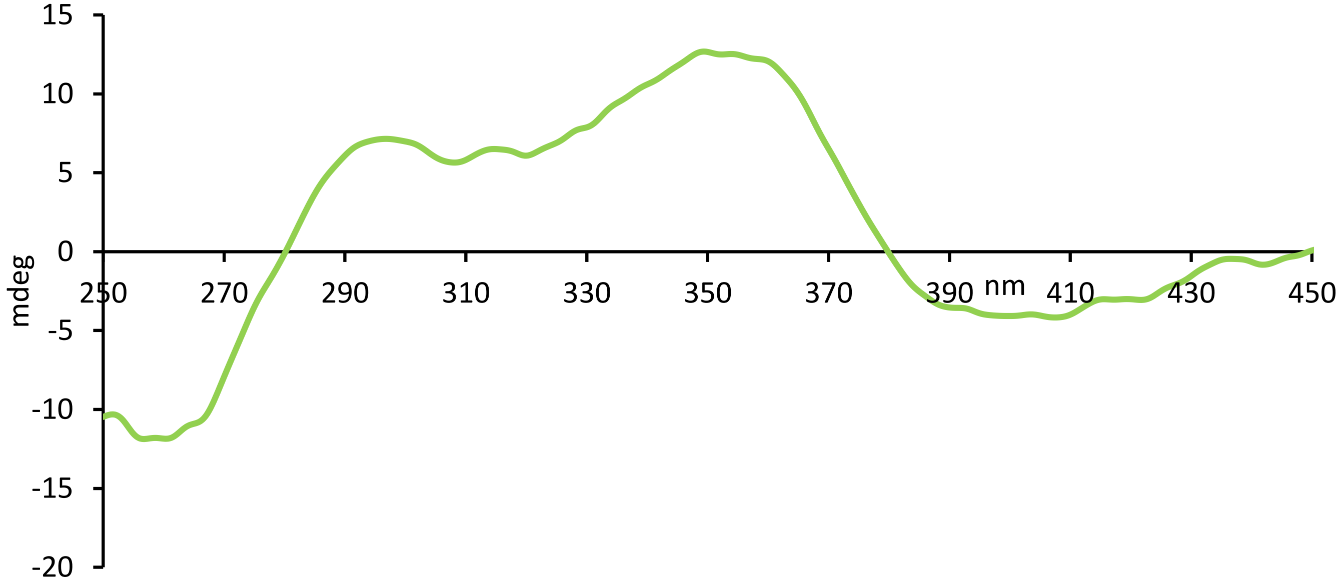

CD measurements were taken at 0.071 mM total analyte concentration in dichloroethane.

Supplementary Figure 29. Entry 7: Mixture of 6.0 mM of (*R*)-**PEA**, 4.0 mM of (*S*)-**PEA**, 6.0 mM of (*R*)-**PMP**, and 4.0 mM of (*S*)-**PMP**

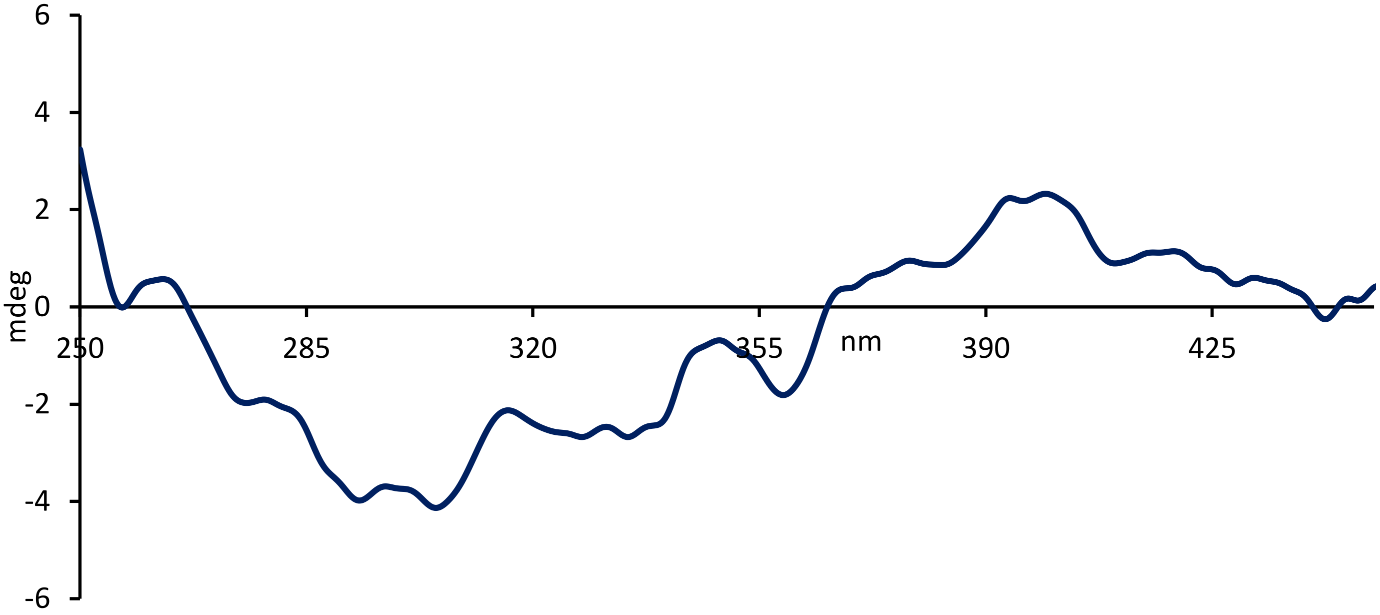

CD measurements were taken at 0.071 mM total analyte concentration in dichloroethane.

Supplementary Figure 30. Entry 8: Mixture of 4.5 mM of (*R*)-**PEA**, 5.5 mM of (*S*)-**PEA**, 4.5 mM of (*R*)-**PMP**, and 5.5 mM of (*S*)-**PMP**

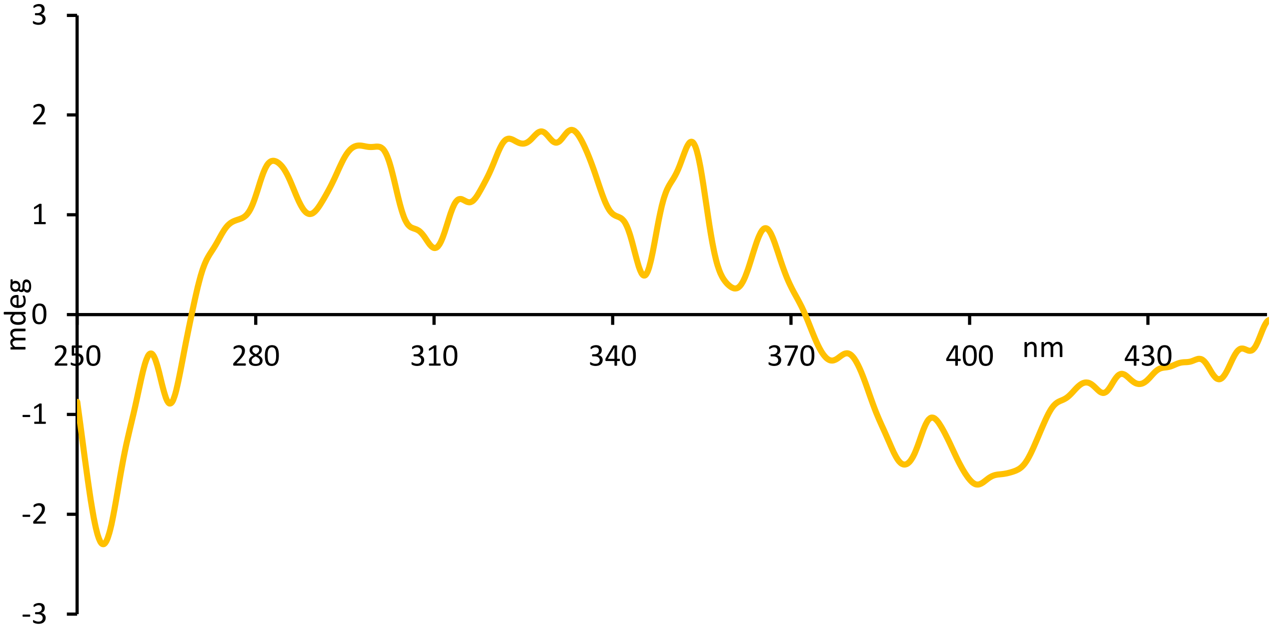

CD measurements were taken at 0.071 mM total analyte concentration in dichloroethane.

Supplementary Figure 31. Entry 9: Mixture of 2.0 mM of (*R*)-**PEA**, 8.0 mM of (*S*)-**PEA**, 7.0 mM of (*R*)-**PMP**, and 3.0 mM of (*S*)-**PMP**

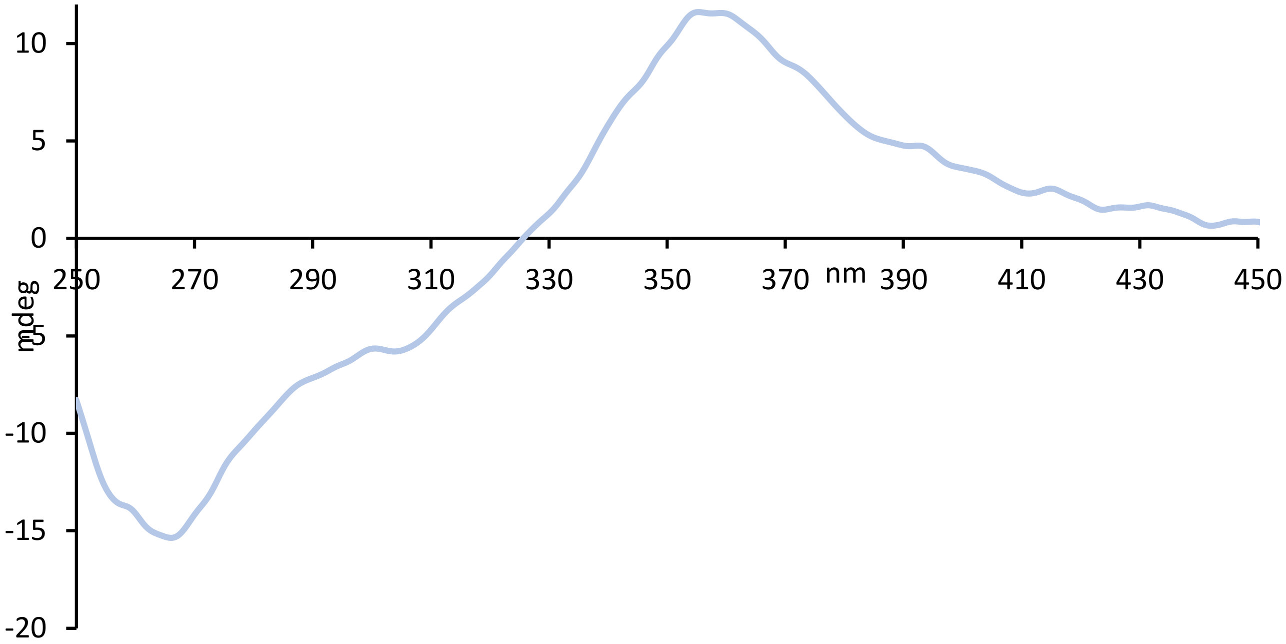

CD measurements were taken at 0.071 mM total analyte concentration in dichloroethane.

Supplementary Figure 32. Entry 10: Mixture of 4.0 mM of (*R*)-**PEA**, 6.0 mM of (*S*)-**PEA**, 9.0 mM of (*R*)-**PMP**, and 1.0 mM of (*S*)-**PMP**

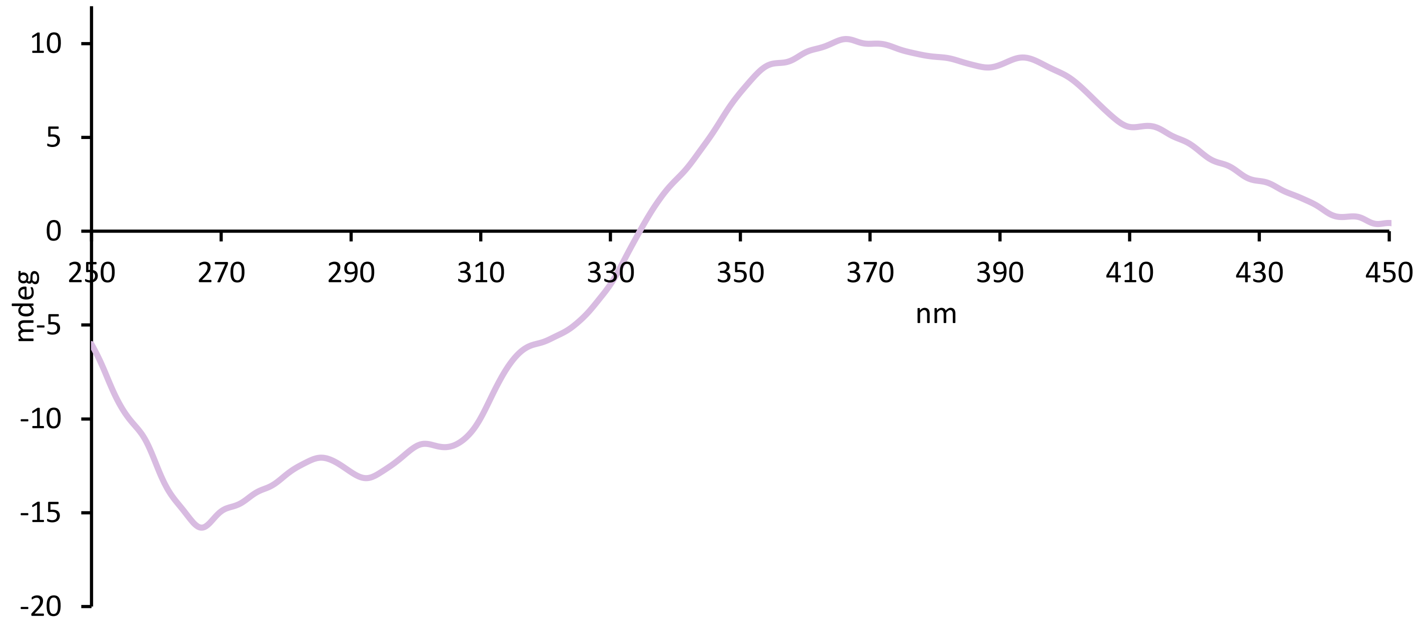

CD measurements were taken at 0.071 mM total analyte concentration in dichloroethane.

Supplementary Figure 33. CD Response (mdeg) of **A** at 340 nm to varying enantiomeric compositions of **PEA** in **PEA**/**PMP** mixtures

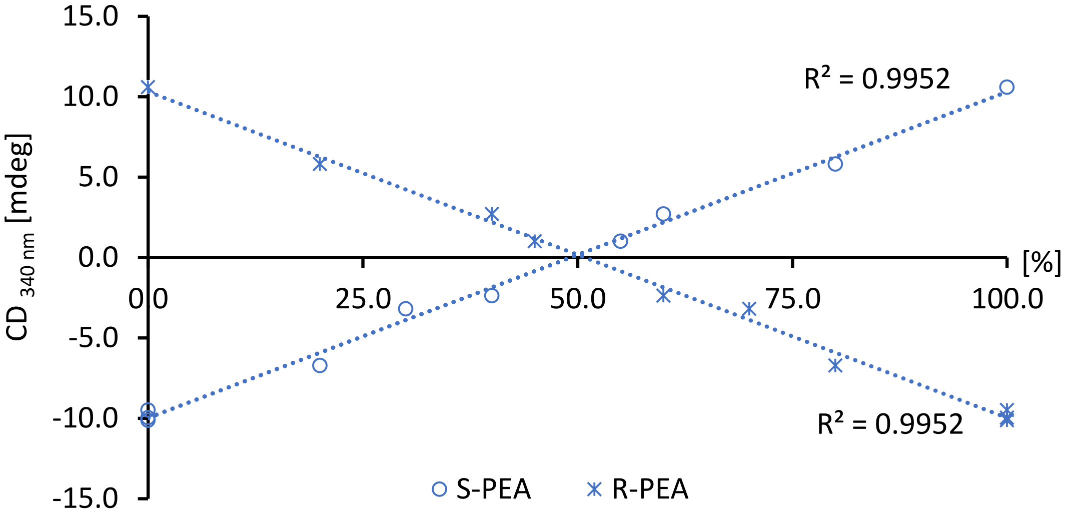

Supplementary Figure 34. CD Response (mdeg) of **A** at 400 nm to varying enantiomeric compositions of **PMP** in **PEA**/**PMP** mixtures

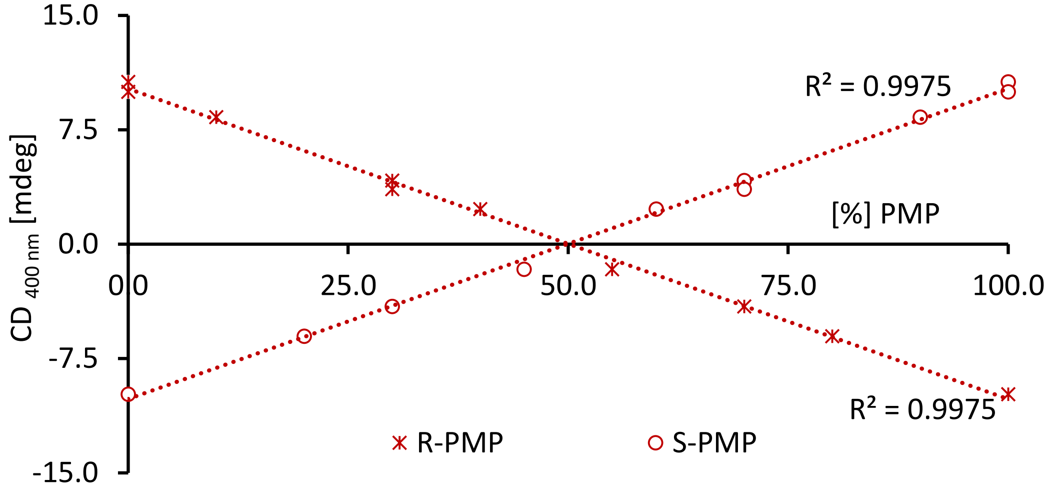

Supplementary Figure 35. Actual versus predicted (*R*)-**PEA** [%]

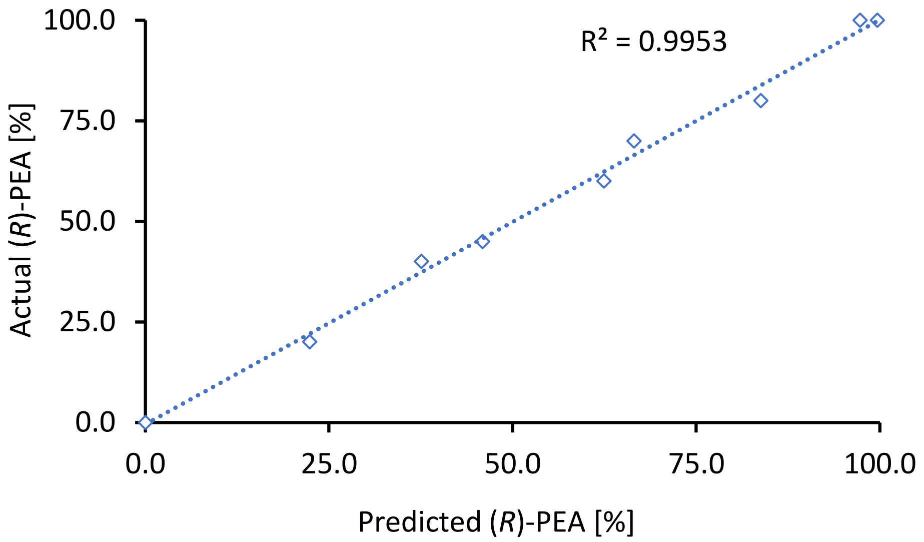

Supplementary Figure 36. Actual versus predicted (*S*)-**PEA** [%]

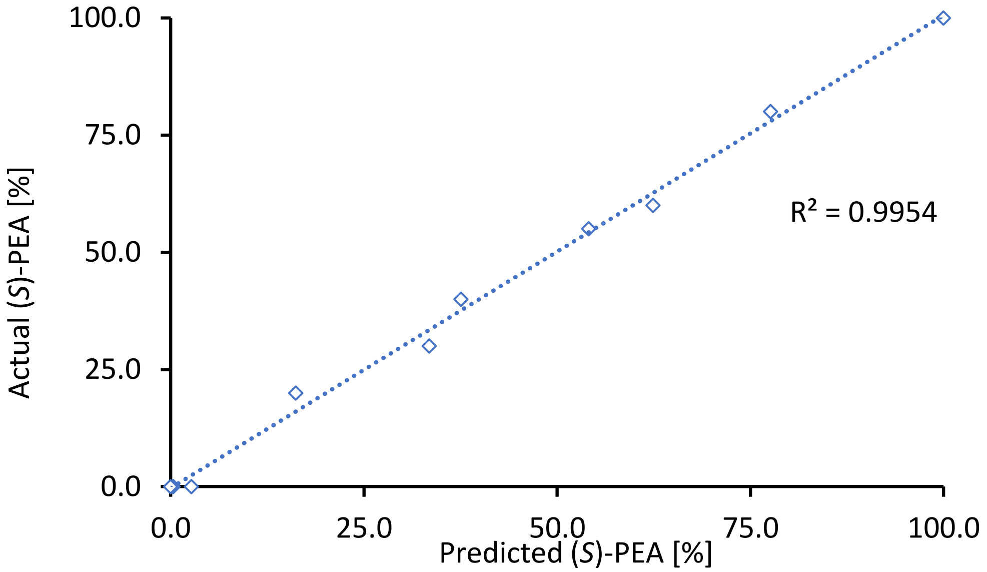

Supplementary Figure 37. Actual versus predicted (*R*)-**PMP** [%]

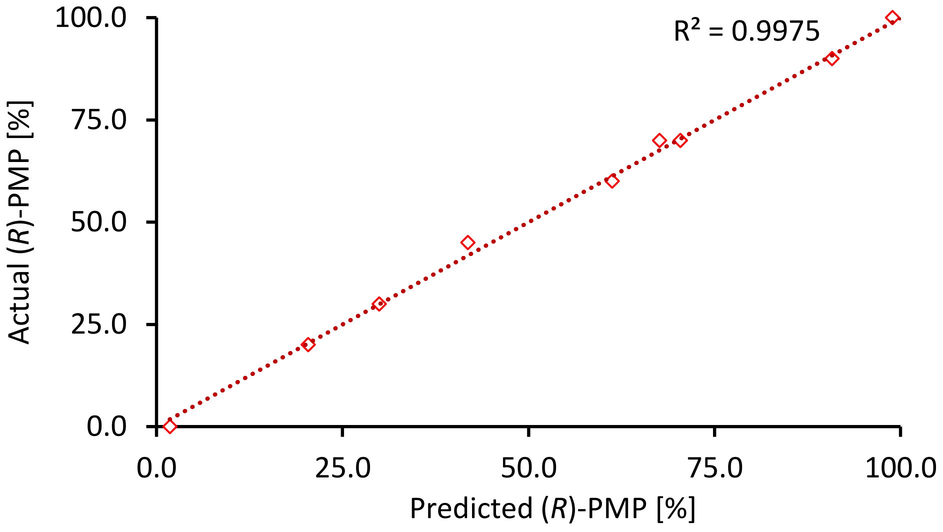

Supplementary Figure 38. Actual versus predicted (*S*)-**PMP** [%]

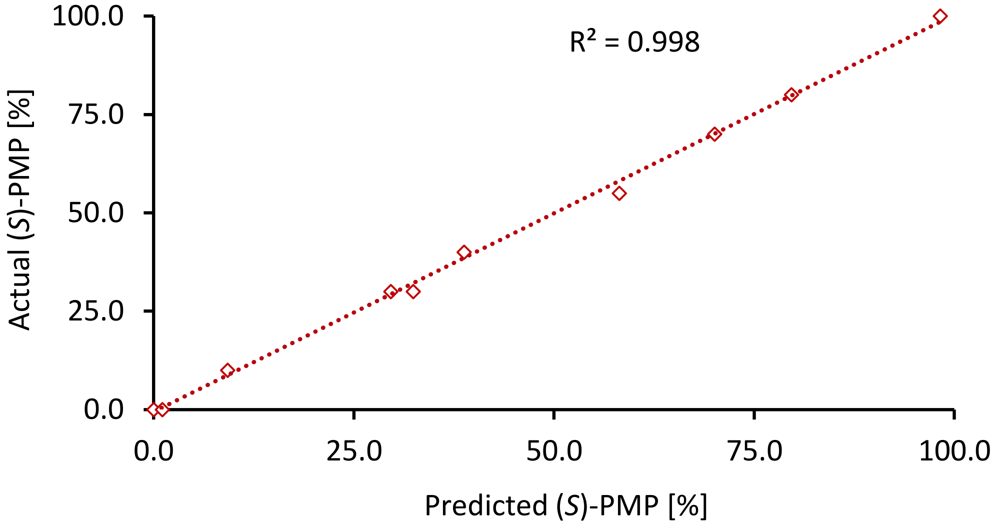

Supplementary Figure 39. CD spectrum of sample 1: Mixture of 1.88 mM of (*R*)-**PEA**, 3.13 mM of (*S*)-**PEA**, 5.63 mM of (*R*)-**PMP**, and 9.38 mM of (*S*)-**PMP**

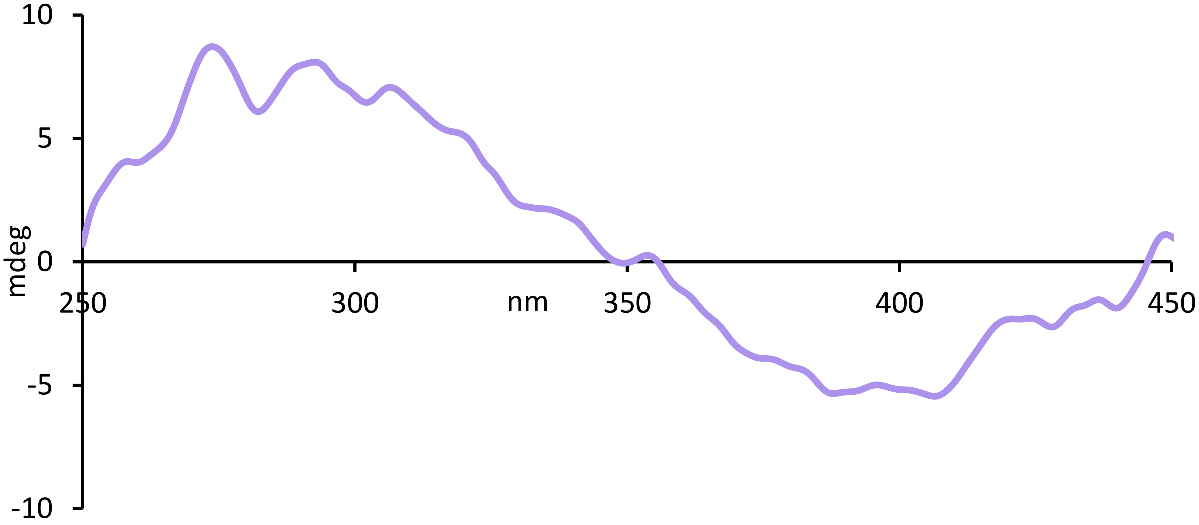

CD measurement was taken at 0.096 mM total analyte concentration in dichloroethane.

Supplementary Figure 40. CD spectrum of sample 2: Mixture of 3.75 mM of (*R*)-**PEA**, 1.25 mM of (*S*)-**PEA**, 11.25 mM of (*R*)-**PMP** and 3.75 mM of (*S*)**-PMP**

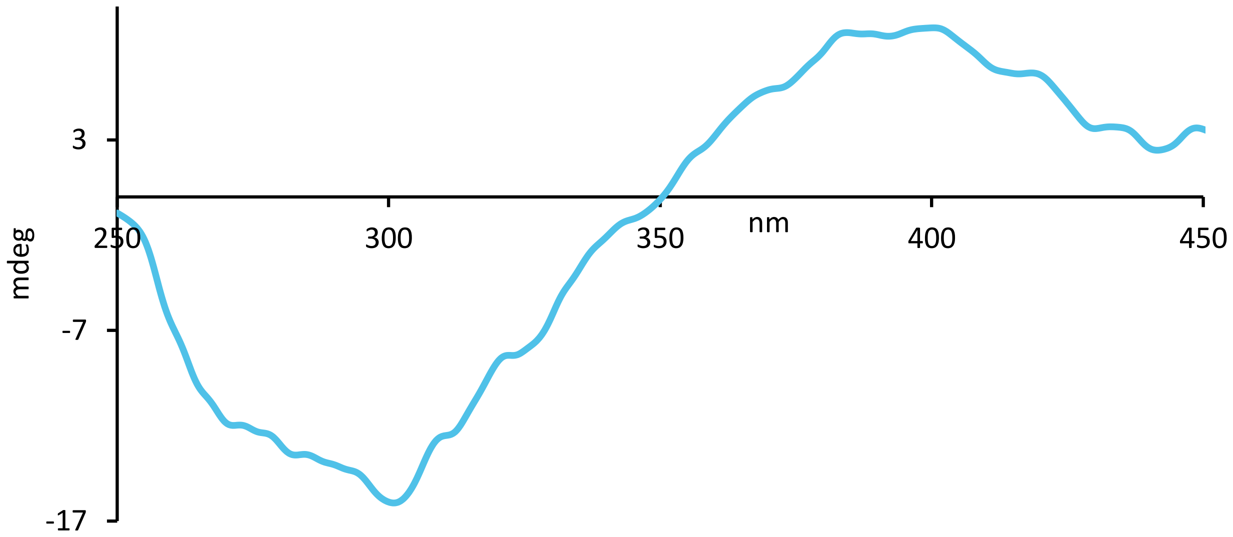

CD measurement was taken at 0.096 mM total analyte concentration in dichloroethane.

Supplementary Figure 41. CD spectrum of sample 3: Mixture of 6.75 mM of (*R*)-**PEA**, 2.25 mM of (*S*)-**PEA**, 8.25 mM of (*R*)-**PMP** and 2.75 mM of (*S*)**-PMP**

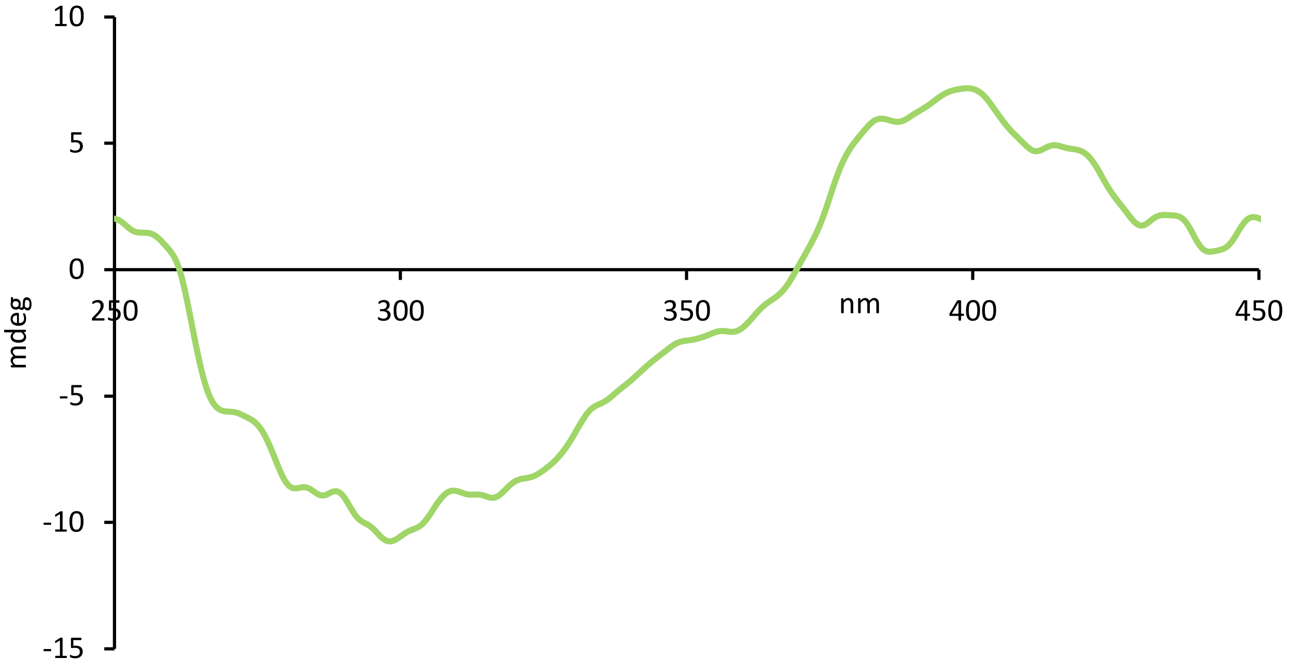

CD measurement was taken at 0.096 mM total analyte concentration in dichloroethane.

Supplementary Figure 42. CD spectrum of sample 4: Mixture of 9.75 mM of (*R*)-**PEA**, 3.25 mM of (*S*)-**PEA**, 5.25 mM of (*R*)-**PMP** and 1.75 mM of (*S*)**-PMP**

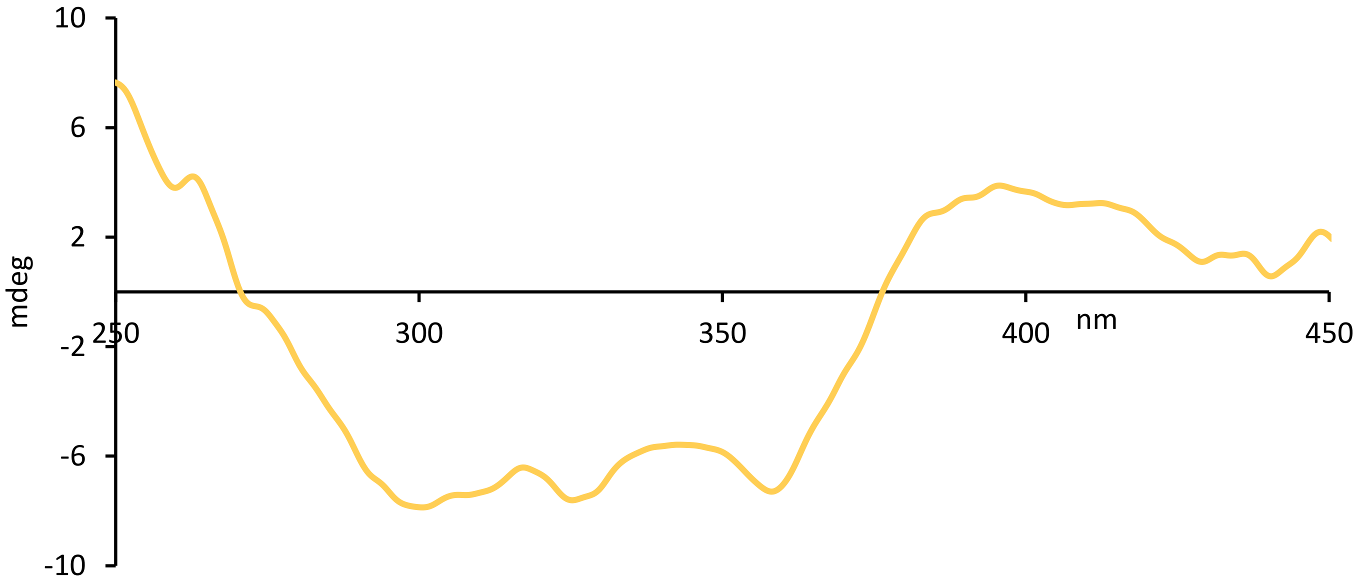

CD measurement was taken at 0.096 mM total analyte concentration in dichloroethane.

Supplementary Figure 43. CD spectrum of sample 5: Mixture of 7.50 mM of (*R*)-**PEA**, 7.50 mM of (*S*)-**PEA**, 2.50 mM of (*R*)-**PMP** and 2.50 mM of (*S*)**-PMP**

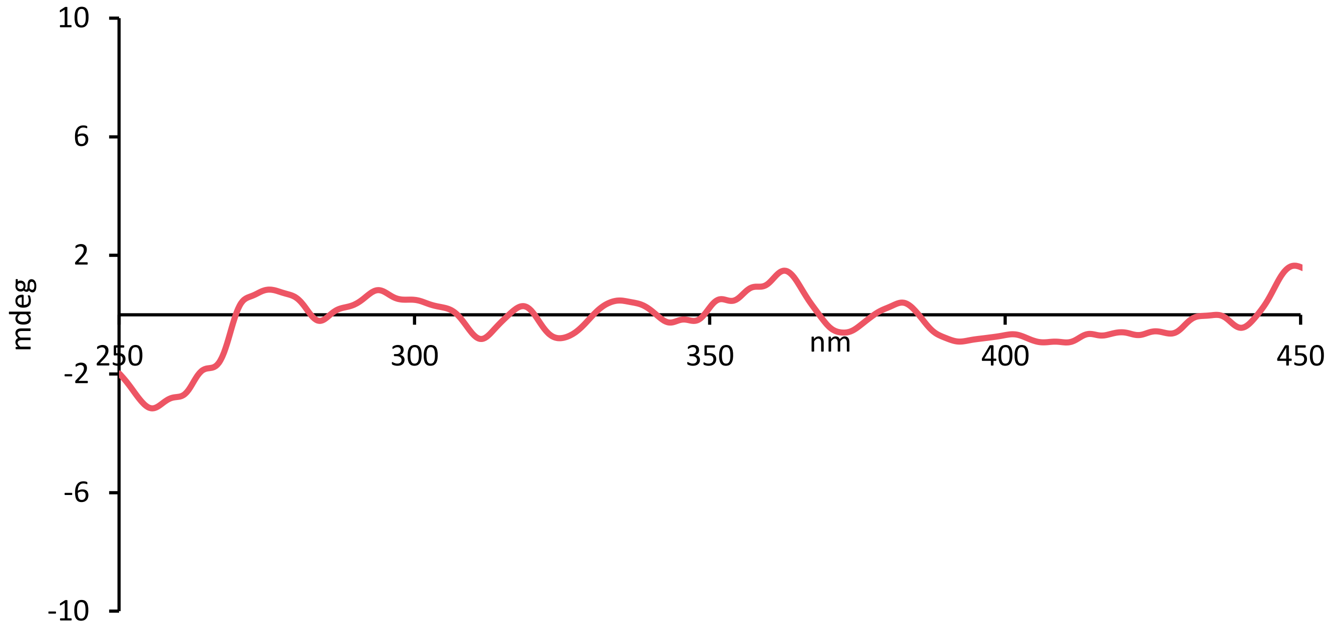

CD measurement was taken at 0.096 mM total analyte concentration in dichloroethane.

Supplementary Figure 44. UV spectrum of sample 1: Mixture of 1.88 mM of (*R*)-**PEA**, 3.13 mM of (*S*)-**PEA**, 5.63 mM of (*R*)-**PMP**, and 9.38 mM of (*S*)-**PMP**

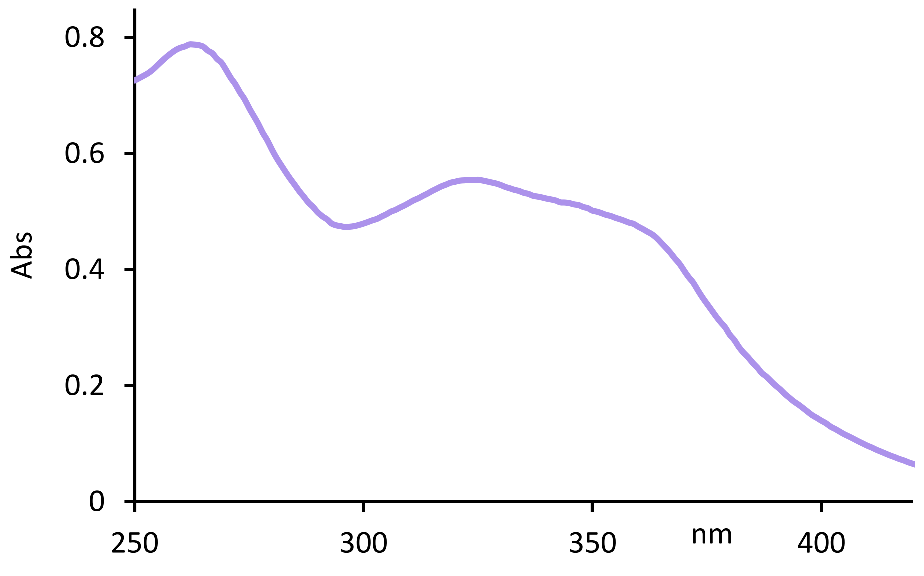

UV measurement was taken at 0.048 mM total analyte concentration in dichloroethane.

Supplementary Figure 45. UV spectrum of sample 2: Mixture of 3.75 mM of (*R*)-**PEA**, 1.25 mM of (*S*)-**PEA**, 11.25 mM of (*R*)-**PMP** and 3.75 mM of (*S*)-**PMP**

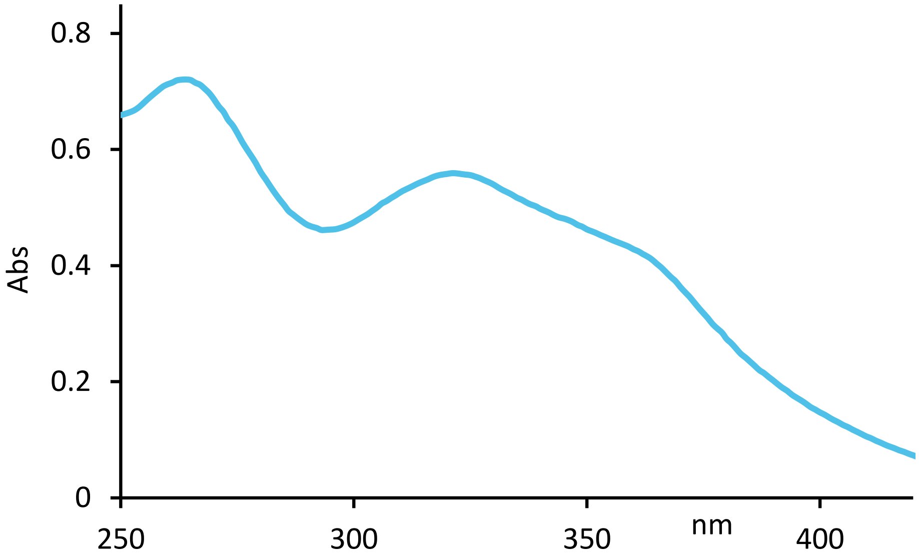

UV measurement was taken at 0.048 mM total analyte concentration in dichloroethane.

Supplementary Figure 46. UV spectrum of sample 3: Mixture of 6.75 mM of (*R*)-**PEA**, 2.25 mM of (*S*)-**PEA**, 8.25 mM of (*R*)-**PMP** and 2.75 mM of (*S*)-**PMP**

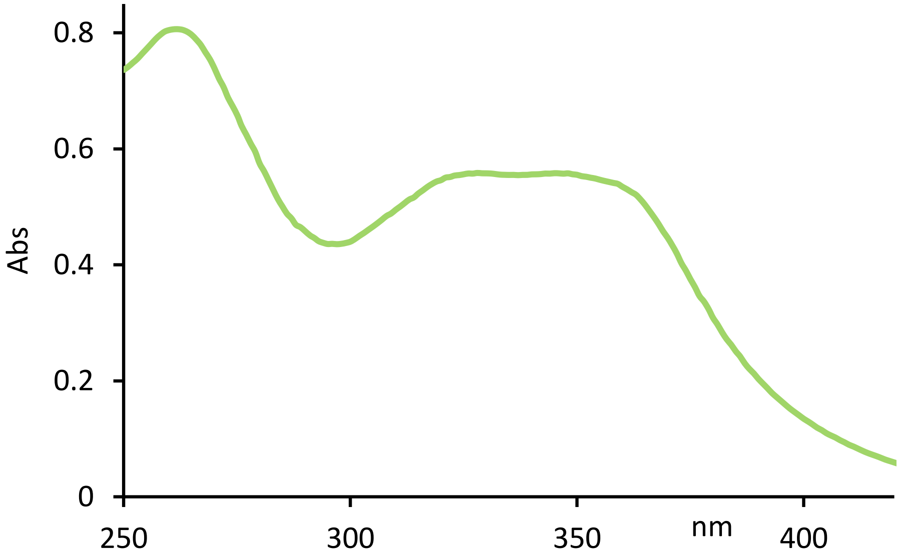

UV measurement was taken at 0.048 mM total analyte concentration in dichloroethane.

Supplementary Figure 47. UV spectrum of sample 4: Mixture of 9.75 mM of (*R*)-**PEA**, 3.25 mM of (*S*)-**PEA**, 5.25 mM of (*R*)-**PMP** and 1.75 mM of (*S*)**-PMP**

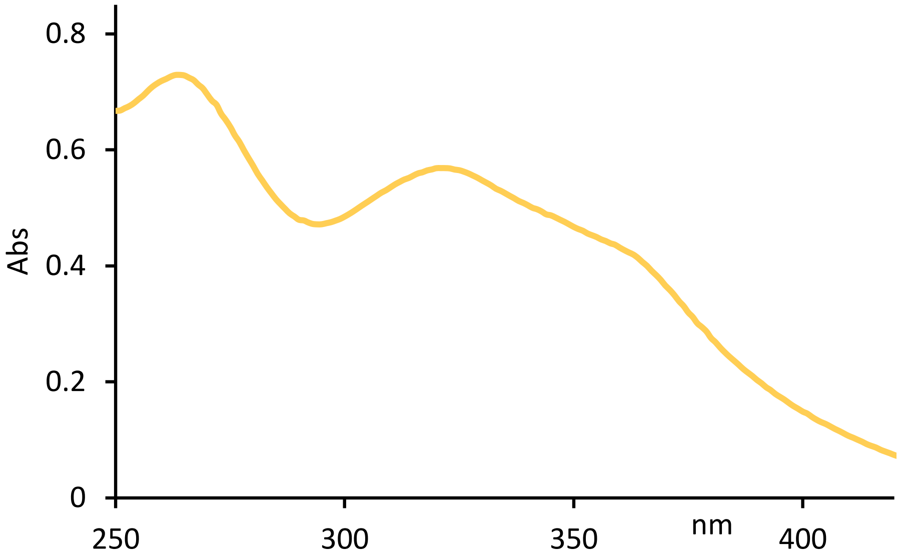

UV measurement was taken at 0.048 mM total analyte concentration in dichloroethane.

Supplementary Figure 48. UV spectrum of sample 5: Mixture of 7.50 mM of (*R*)-**PEA**, 7.50 mM of (*S*)-**PEA**, 2.50 mM of (*R*)-**PMP** and 2.50 mM of (*S*)**-PMP**

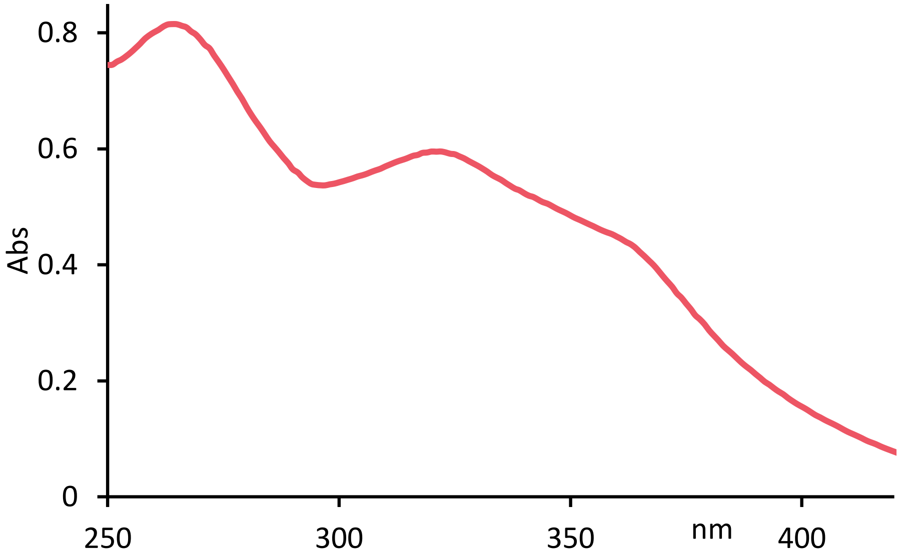

UV measurement was taken at 0.048 mM total analyte concentration in dichloroethane.

Supplementary Figure 49. Comparison of experimentally obtained and reconstructed CD spectra

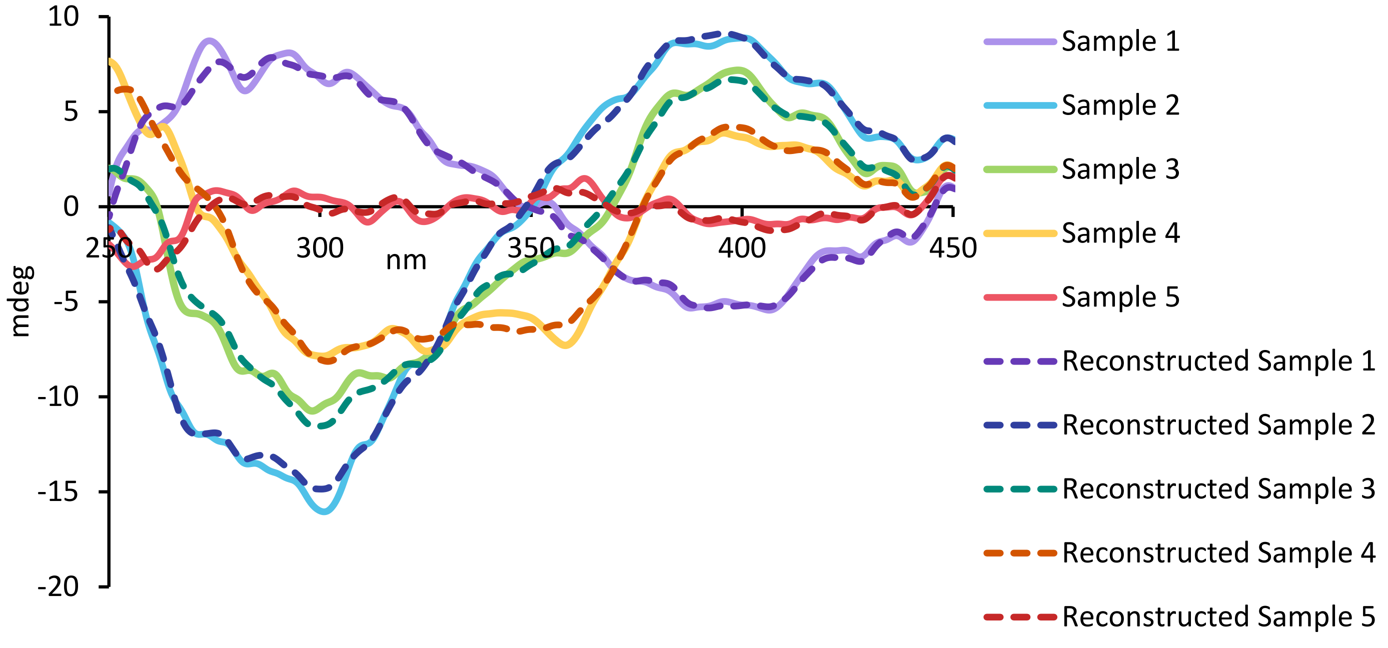

Supplementary Figure 50. Comparison of experimentally obtained and reconstructed UV spectra

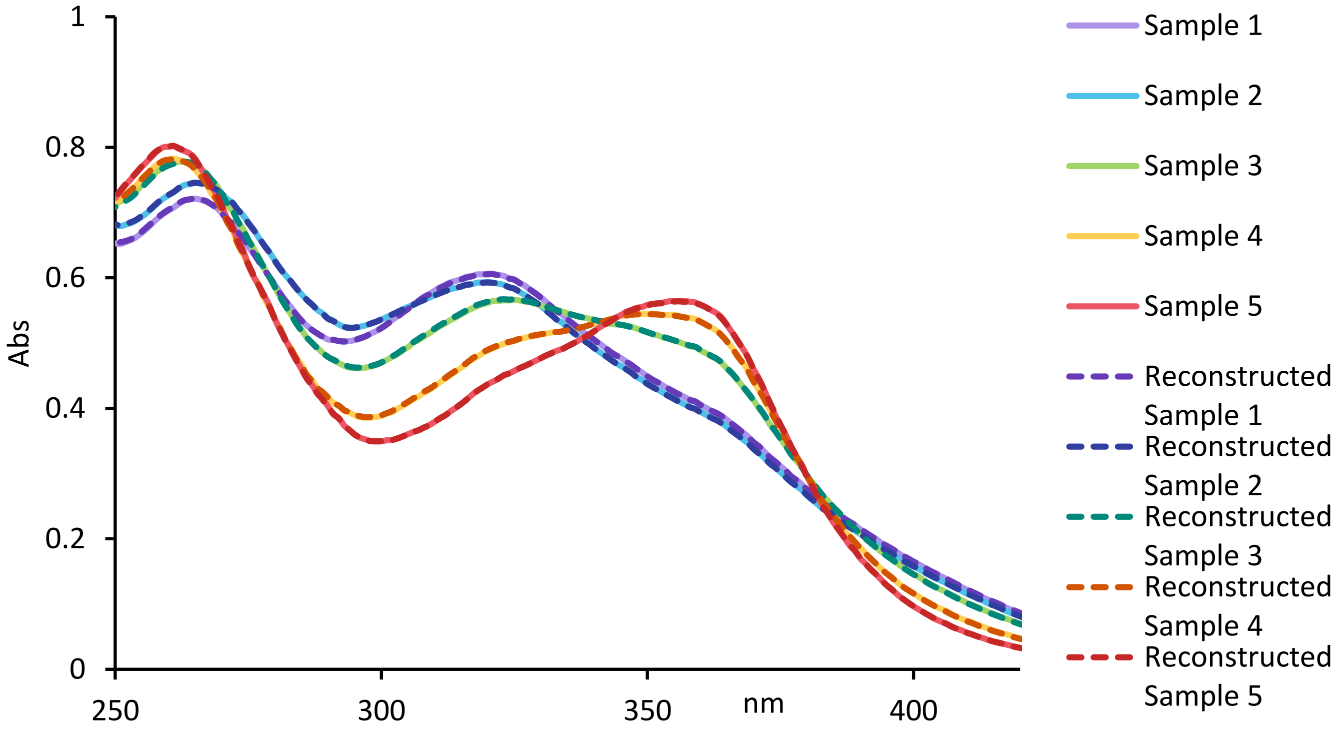

Supplementary Figure 51. Multidimensionality of the CD amplitude of **A** in response to the enantiomeric excess and concentration of **PMP**

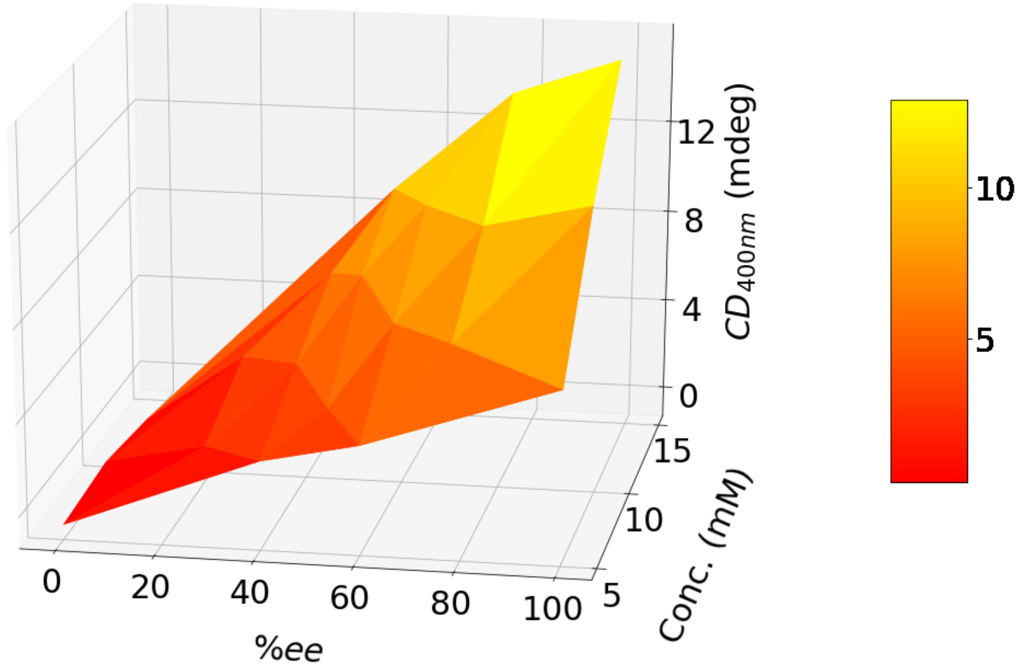

Supplementary Figure 52. CD sensing of training set 1: Mixture of 5.60 mM of (*R*)-**PEA**, 1.40 mM of (*S*)-**PEA**, 10.40 mM of (*R*)-**PMP** and 2.60 mM of (*S*)-**PMP**

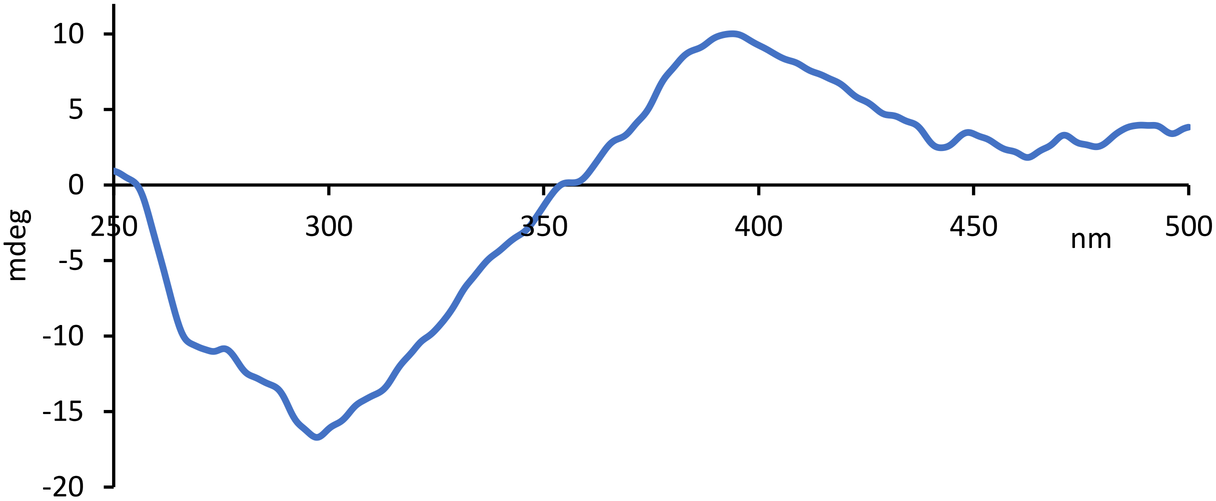

CD measurement was taken at 0.096 mM total analyte concentration in dichloroethane.

Supplementary Figure 53. CD sensing of training set 2: Mixture of 7.70 mM of (*R*)-**PEA**, 3.30 mM of (*S*)-**PEA**, 6.30 mM of (*R*)-**PMP** and 2.70 mM of (*S*)-**PMP**

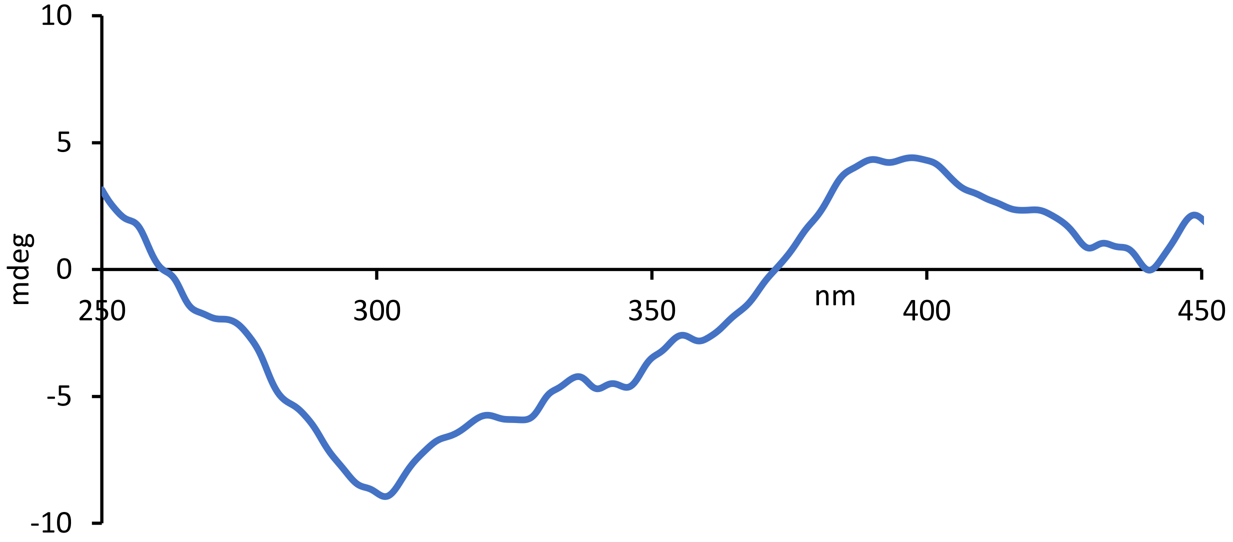

CD measurement was taken at 0.096 mM total analyte concentration in dichloroethane.

Supplementary Figure 54. CD sensing of training set 3: Mixture of 3.50 mM of (*R*)-**PEA**, 3.50 mM of (*S*)-**PEA**, 6.50 mM of (*R*)-**PMP** and 6.50 mM of (*S*)-**PMP**

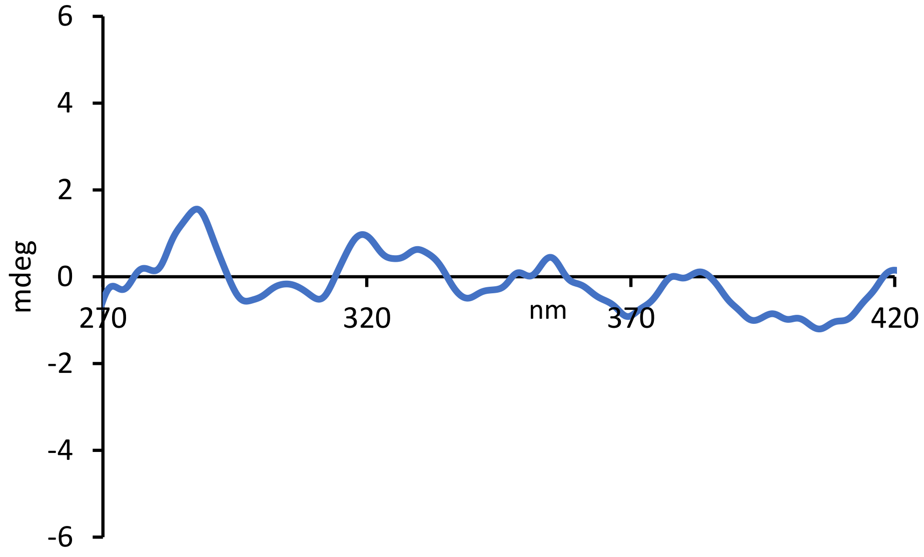

CD measurement was taken at 0.096 mM total analyte concentration in dichloroethane.

Supplementary Figure 55. CD sensing of training set 4: Mixture of 10.50 mM of (*R*)-**PEA**, 4.50 mM of (*S*)-**PEA**, 3.50 mM of (*R*)-**PMP** and 1.50 mM of (*S*)-**PMP**

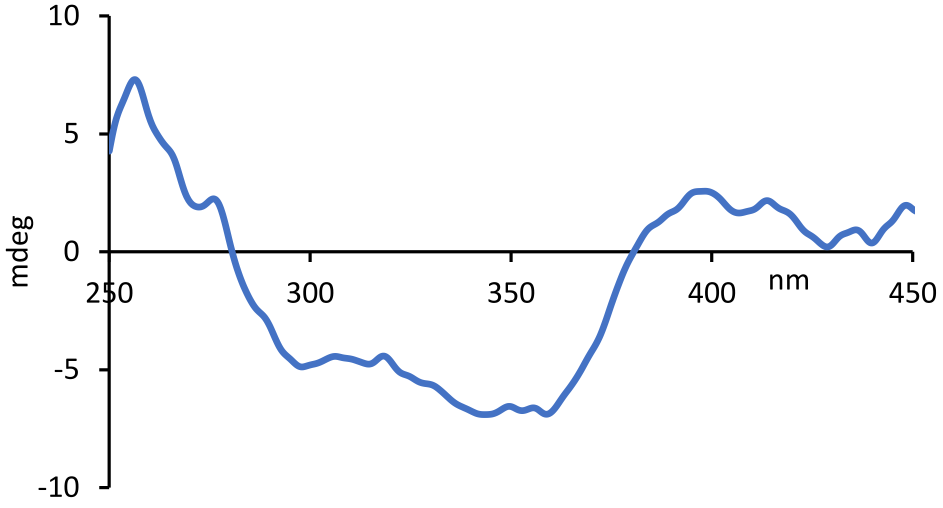

CD measurement was taken at 0.096 mM total analyte concentration in dichloroethane.

Supplementary Figure 56. CD sensing of training set 5: Mixture of 11.38 mM of (*R*)-**PEA**, 1.63 mM of (*S*)-**PEA**, 6.13 mM of (*R*)-**PMP** and 0.88 mM of (*S*)-**PMP**

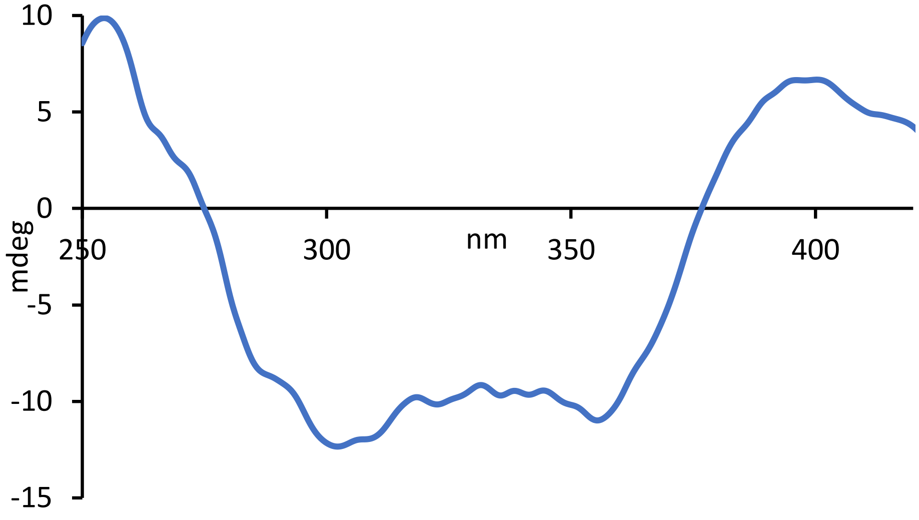

CD measurement was taken at 0.096 mM total analyte concentration in dichloroethane.

Supplementary Figure 57. CD sensing of training set 6: Mixture of 11.00 mM of (*R*)-**PEA**, 0.00 mM of (*S*)-**PEA**, 9.00 mM of (*R*)-**PMP** and 0.00 mM of (*S*)-**PMP**

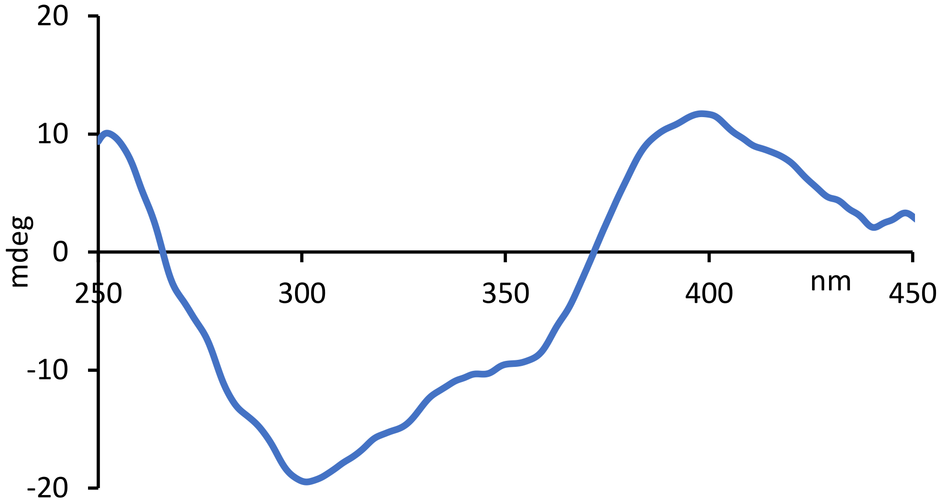

CD measurement was taken at 0.096 mM total analyte concentration in dichloroethane.

Supplementary Figure 58. CD sensing of training set 7: Mixture of 4.38 mM of (*R*)-**PEA**, 0.63 mM of (*S*)-**PEA**, 13.13 mM of (*R*)-**PMP** and 1.88 mM of (*S*)-**PMP**

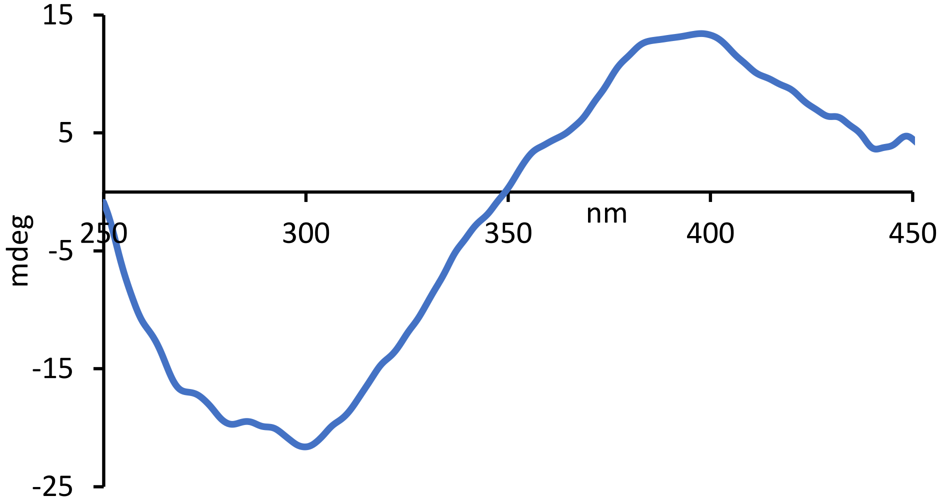

CD measurement was taken at 0.096 mM total analyte concentration in dichloroethane.

Supplementary Figure 59. CD sensing of training set 8: Mixture of 7.88 mM of (*R*)-**PEA**, 1.13 mM of (*S*)-**PEA**, 9.63 mM of (*R*)-**PMP** and 1.38 mM of (*S*)-**PMP**

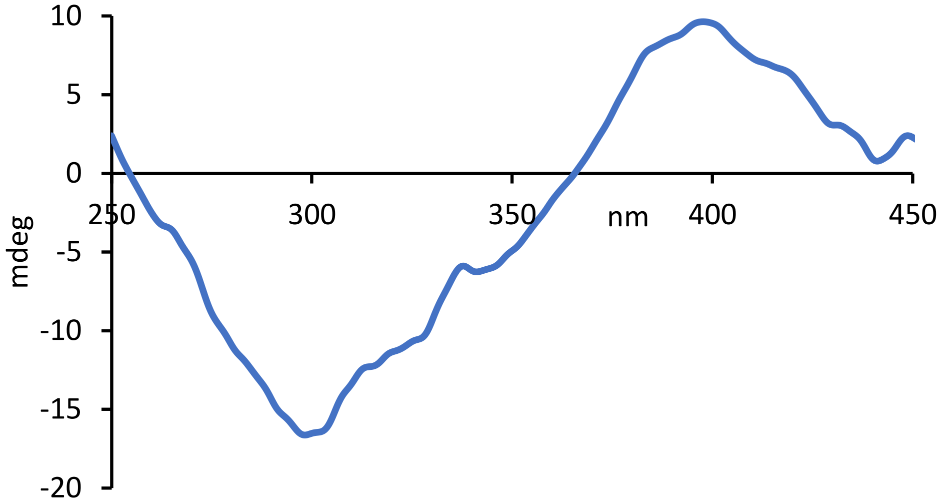

CD measurement was taken at 0.096 mM total analyte concentration in dichloroethane.

Supplementary Figure 60. CD sensing of training set 9: Mixture of 15.00 mM of (*R*)-**PEA**, 0.00 mM of (*S*)-**PEA**, 5.00 mM of (*R*)-**PMP** and 0.00 mM of (*S*)-**PMP**

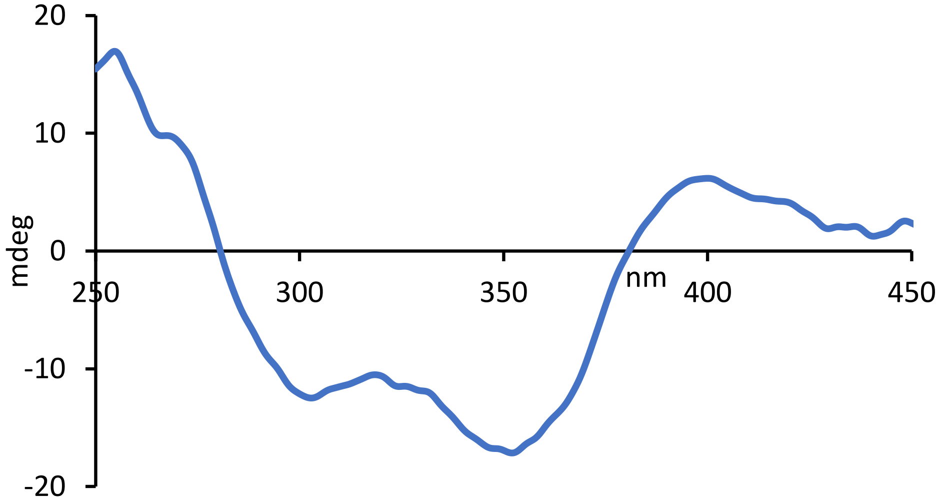

CD measurement was taken at 0.096 mM total analyte concentration in dichloroethane.

Supplementary Figure 61 CD sensing of training set 10: Mixture of 4.90 mM of (*R*)-**PEA**, 2.10 mM of (*S*)-**PEA**, 9.10 mM of (*R*)-**PMP** and 3.90 mM of (*S*)-**PMP**

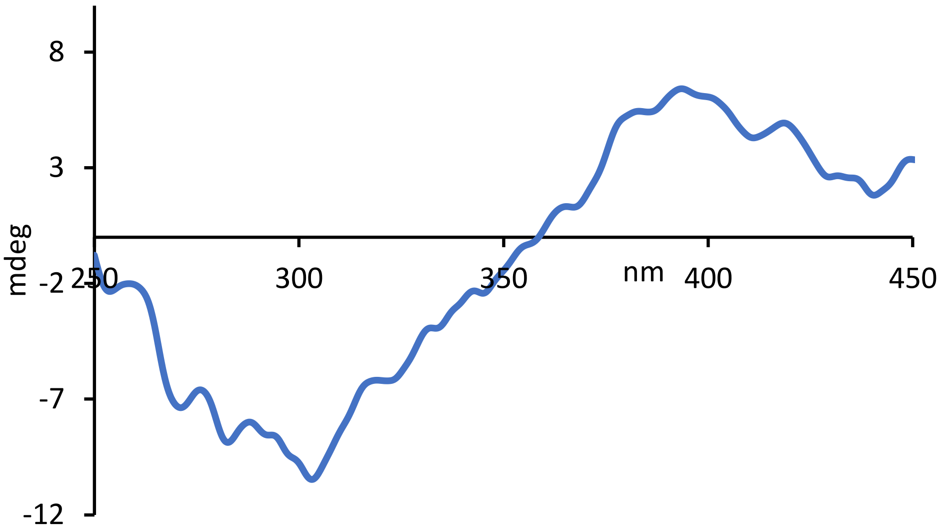

CD measurement was taken at 0.096 mM total analyte concentration in dichloroethane.

Supplementary Figure 62. CD sensing of training set 11: Mixture of 8.80 mM of (*R*)-**PEA**, 2.20 mM of (*S*)-**PEA**, 7.20 mM of (*R*)-**PMP** and 1.80 mM of (*S*)-**PMP**

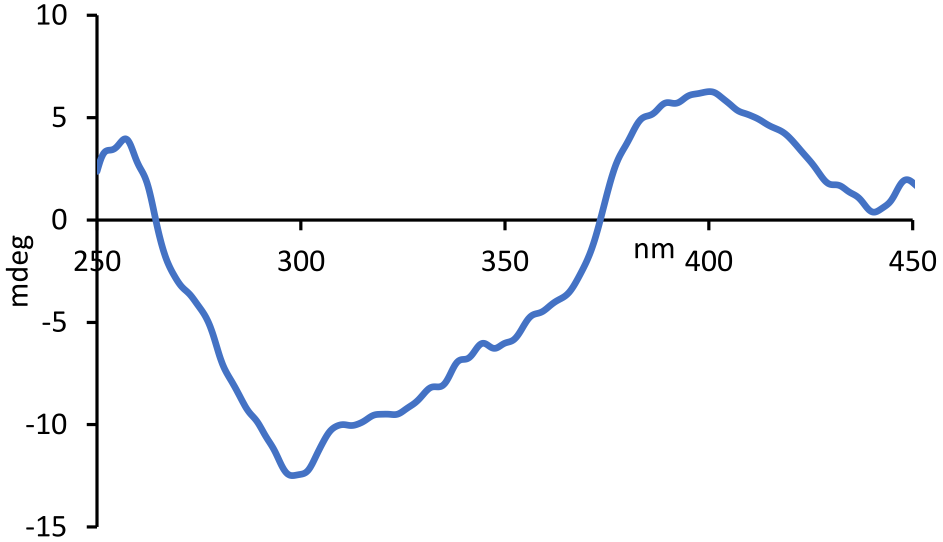

CD measurement was taken at 0.096 mM total analyte concentration in dichloroethane.

Supplementary Figure 63. CD sensing of training set 12: Mixture of 5.63 mM of (*R*)-**PEA**, 3.38 mM of (*S*)-**PEA**, 6.88 mM of (*R*)-**PMP** and 4.13 mM of (*S*)-**PMP**

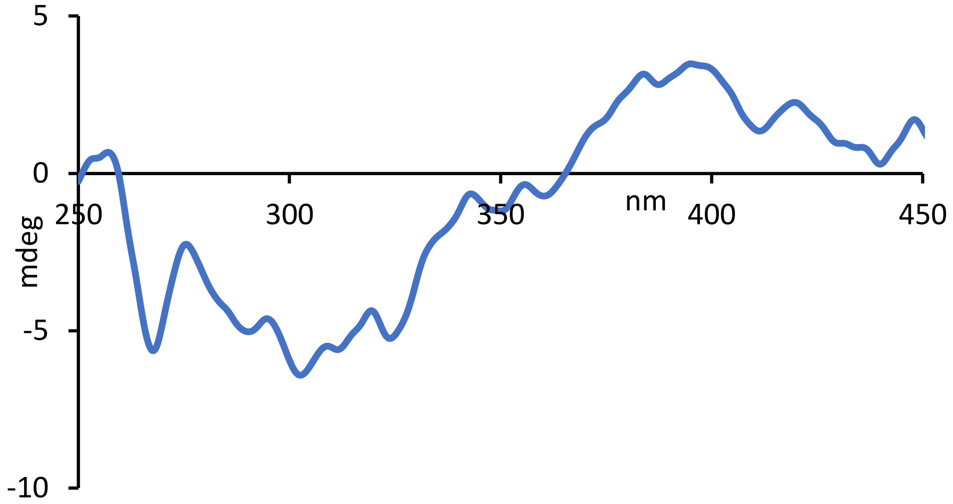

CD measurement was taken at 0.096 mM total analyte concentration in dichloroethane.

Supplementary Figure 64. CD sensing of training set 13: Mixture of 5.50 mM of (*R*)-**PEA**, 5.50 mM of (*S*)-**PEA**, 4.50 mM of (*R*)-**PMP** and 4.50 mM of (*S*)-**PMP**

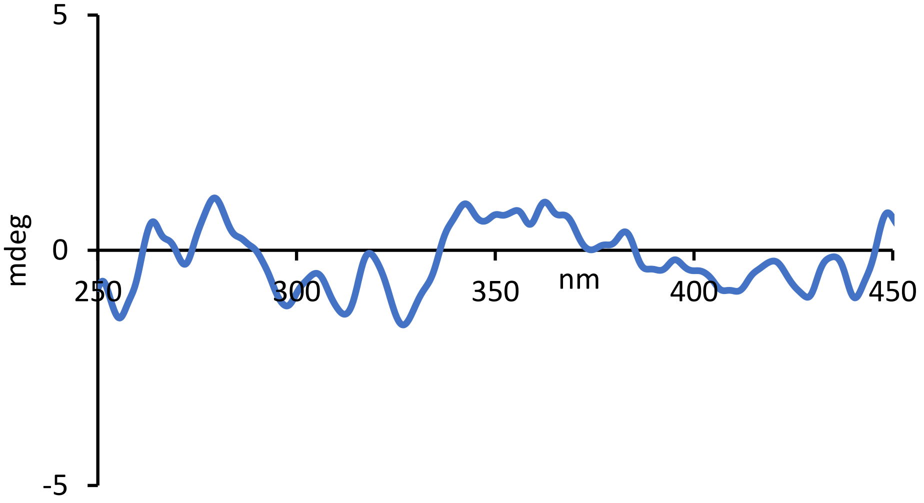

CD measurement was taken at 0.096 mM total analyte concentration in dichloroethane.

Supplementary Figure 65. CD sensing of training set 14: Mixture of 8.13 mM of (*R*)-**PEA**, 4.88 mM of (*S*)-**PEA**, 4.38 mM of (*R*)-**PMP** and 2.63 mM of (*S*)-**PMP**

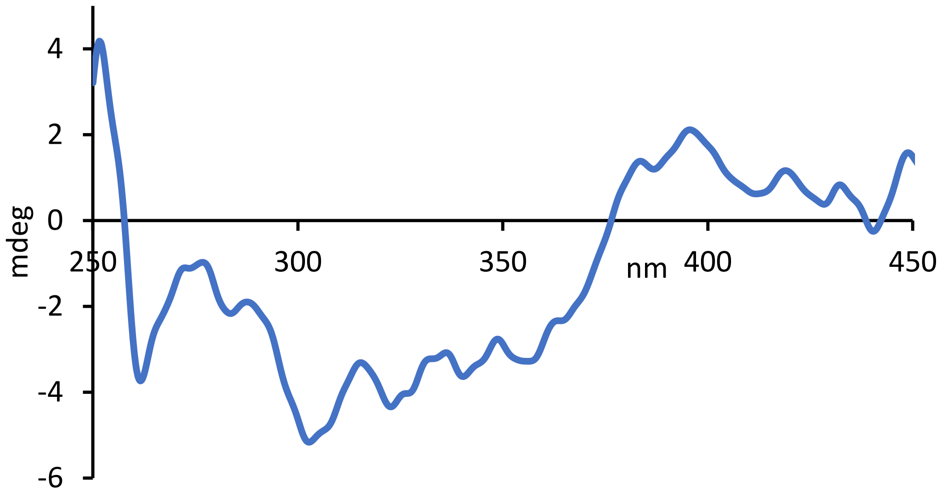

CD measurement was taken at 0.096 mM total analyte concentration in dichloroethane.

Supplementary Figure 66. CD sensing of training set 15: Mixture of 12.00 mM of (*R*)-**PEA**, 3.00 mM of (*S*)-**PEA**, 4.00 mM of (*R*)-**PMP** and 1.00 mM of (*S*)-**PMP**

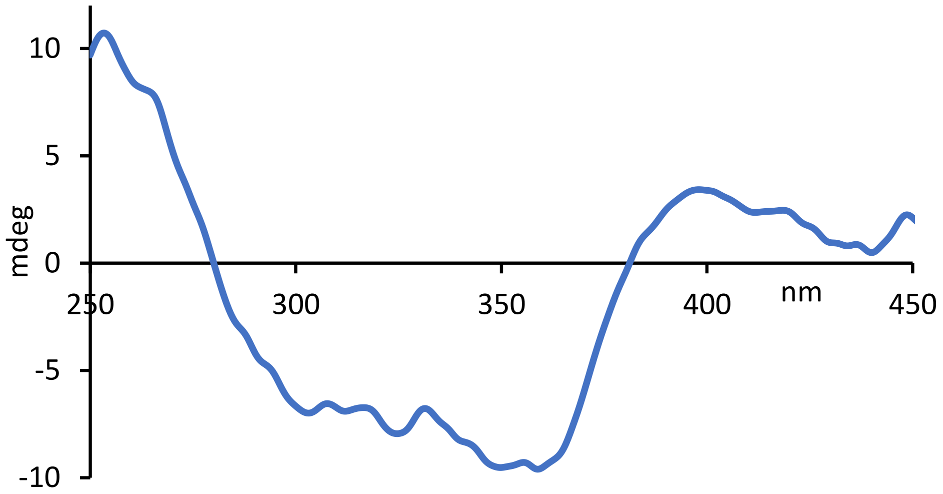

CD measurement was taken at 0.096 mM total analyte concentration in dichloroethane.

Supplementary Figure 67. CD sensing of training set 16: Mixture of 7.00 mM of (*R*)-**PEA**, 0.00 mM of (*S*)-**PEA**, 13.00 mM of (*R*)-**PMP** and 0.00 mM of (*S*)**-PMP**

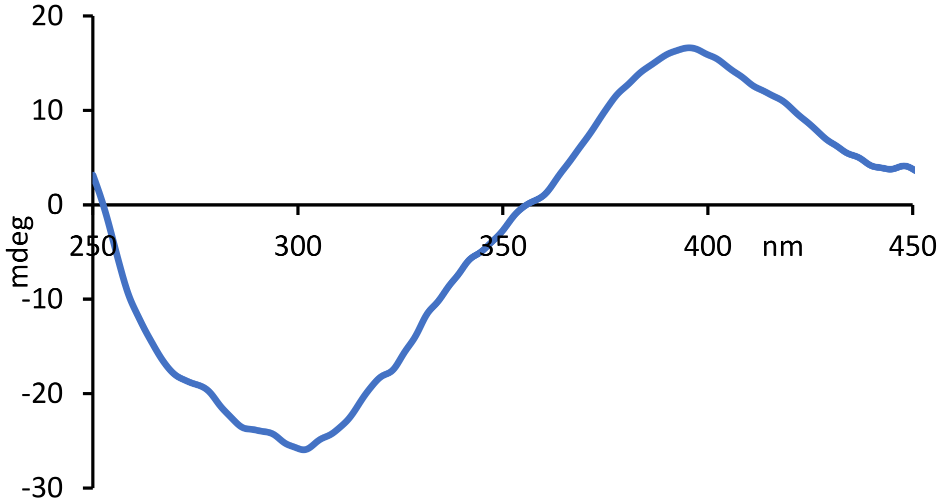

CD measurement was taken at 0.096 mM total analyte concentration in dichloroethane.

Supplementary Figure 68. CD sensing of test set 1: Mixture of 1.88 mM of (*R*)-**PEA**, 3.13 mM of (*S*)-**PEA**, 5.63 mM of (*R*)-**PMP** and 9.38 mM of (*S*)**-PMP**

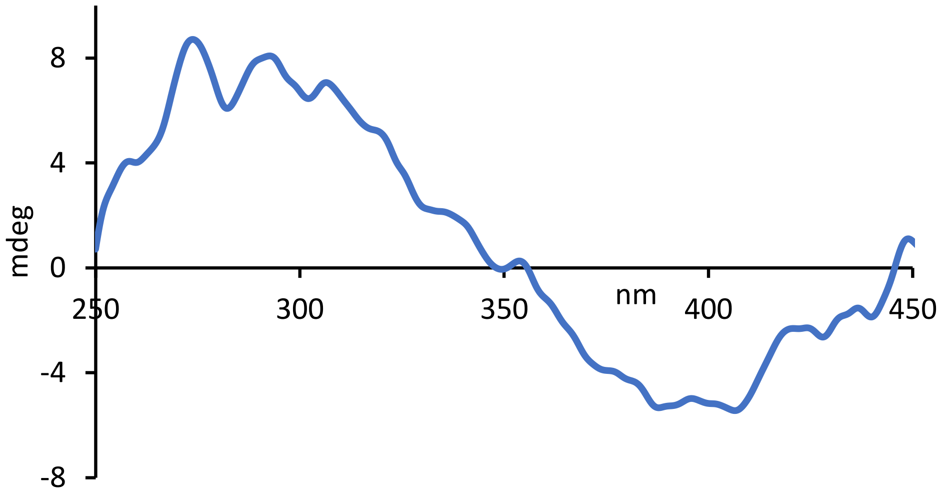

CD measurement was taken at 0.096 mM total analyte concentration in dichloroethane.

Supplementary Figure 69. CD sensing of test set 2: Mixture of 7.50 mM of (*R*)-**PEA**, 7.50 mM of (*S*)-**PEA**, 2.50 mM of (*R*)-**PMP** and 2.50 mM of (*S*)-**PMP**

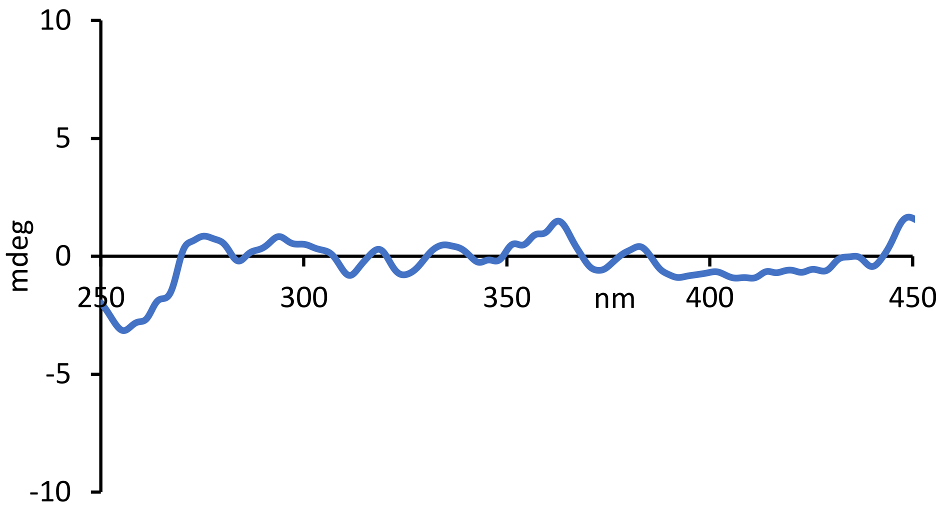

CD measurement was taken at 0.096 mM total analyte concentration in dichloroethane.

Supplementary Figure 70. CD sensing of test set 3: Mixture of 9.75 mM of (*R*)-**PEA**, 3.25 mM of (*S*)-**PEA**, 5.25 mM of (*R*)-**PMP** and 1.75 mM of (*S*)-**PMP**

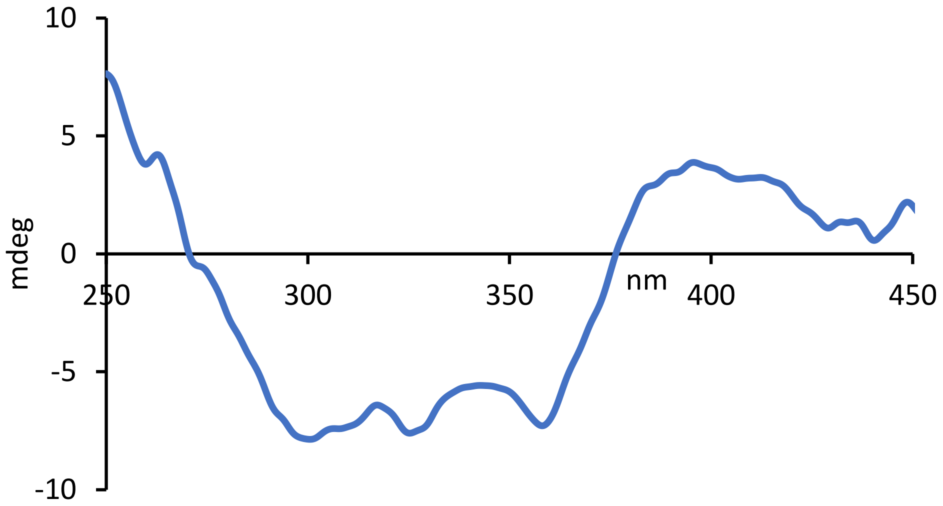

CD measurement was taken at 0.096 mM total analyte concentration in dichloroethane.

Supplementary Figure 71. CD sensing of test set 4: Mixture of 3.75 mM of (*R*)-**PEA**, 1.25 mM of (*S*)-**PEA**, 11.25 mM of (*R*)-**PMP** and 3.75 mM of (*S*)-**PMP**

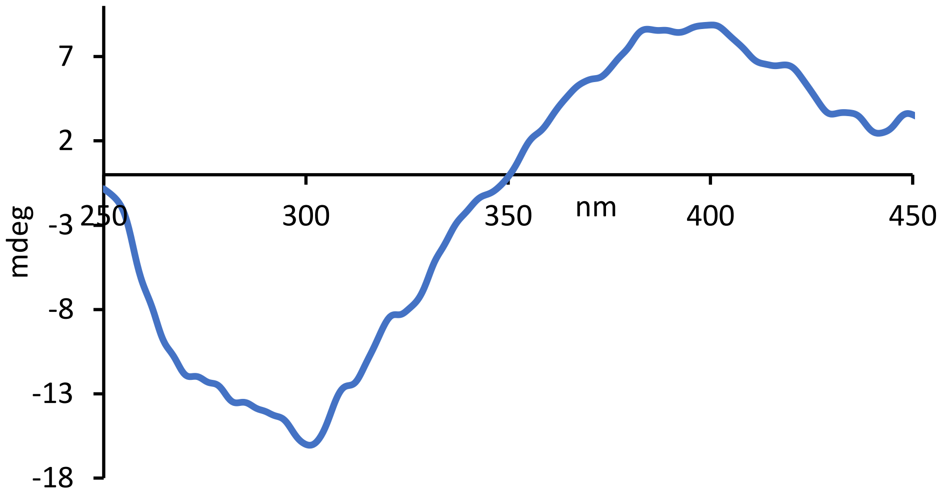

CD measurement was taken at 0.096 mM total analyte concentration in dichloroethane.

Supplementary Figure 72. CD sensing of test set 5: Mixture of 6.75 mM of (*R*)-**PEA**, 2.25 mM of (*S*)-**PEA**, 8.25 mM of (*R*)-**PMP** and 2.75 mM of (*S*)-**PMP**

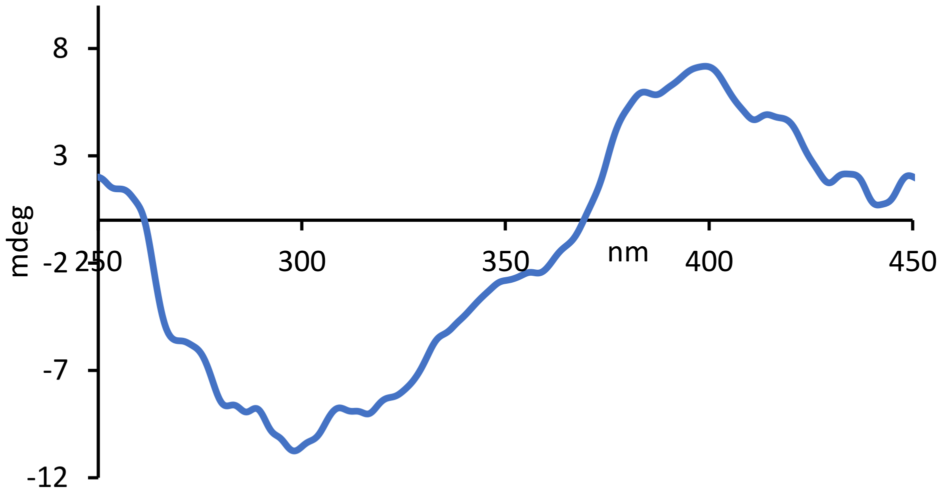

CD measurement was taken at 0.096 mM total analyte concentration in dichloroethane.

Supplementary Figure 73. UV sensing of training set 1: Mixture of 5.60 mM of (*R*)-**PEA**, 1.40 mM of (*S*)-**PEA**, 10.40 mM of (*R*)-**PMP** and 2.60 mM of (*S*)**-PMP**

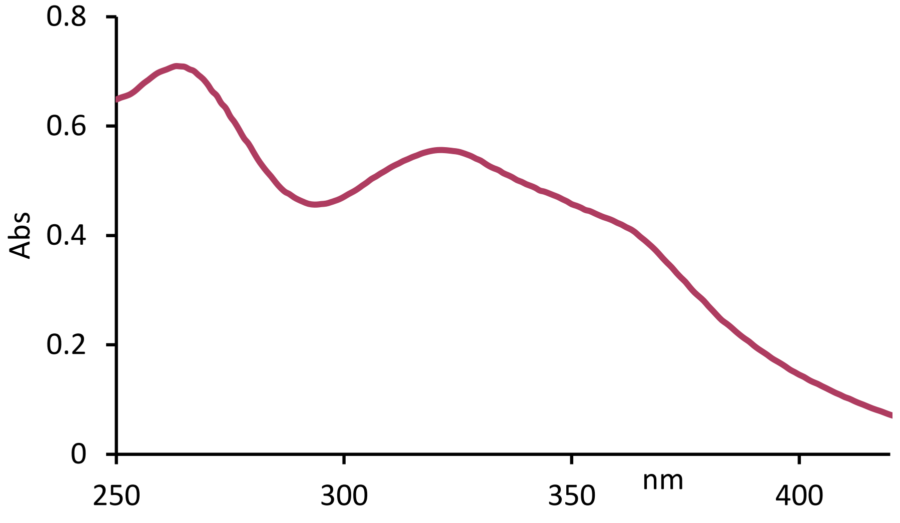

UV measurement was taken at 0.048 mM total analyte concentration in dichloroethane.

Supplementary Figure 74. UV sensing of training set 2: Mixture of 7.70 mM of (*R*)-**PEA**, 3.30 mM of (*S*)-**PEA**, 6.30 mM of (*R*)-**PMP** and 2.70 mM of (*S*)**-PMP**

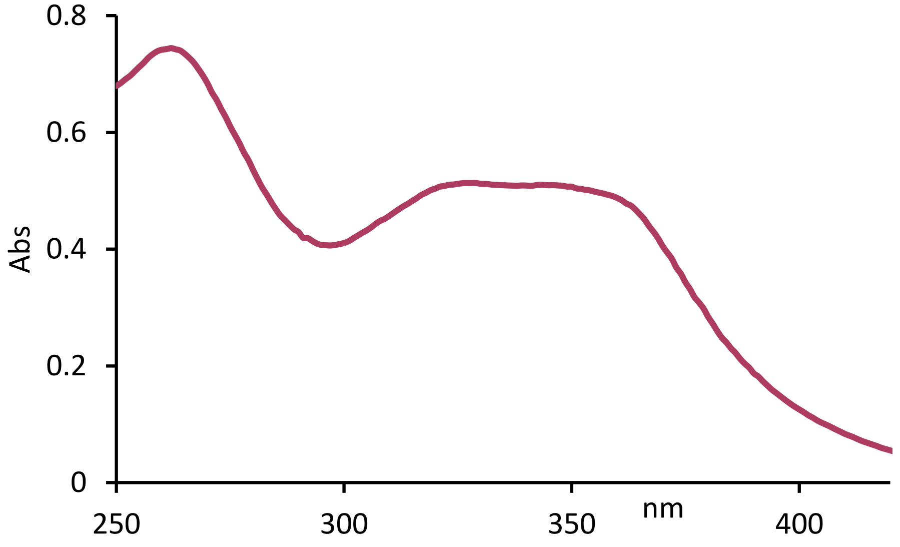

UV measurement was taken at 0.048 mM total analyte concentration in dichloroethane.

Supplementary Figure 75. UV sensing of training set 3: Mixture of 3.50 mM of (*R*)-**PEA**, 3.50 mM of (*S*)-**PEA**, 6.50 mM of (*R*)-**PMP** and 6.50 mM of (*S*)-**PMP**

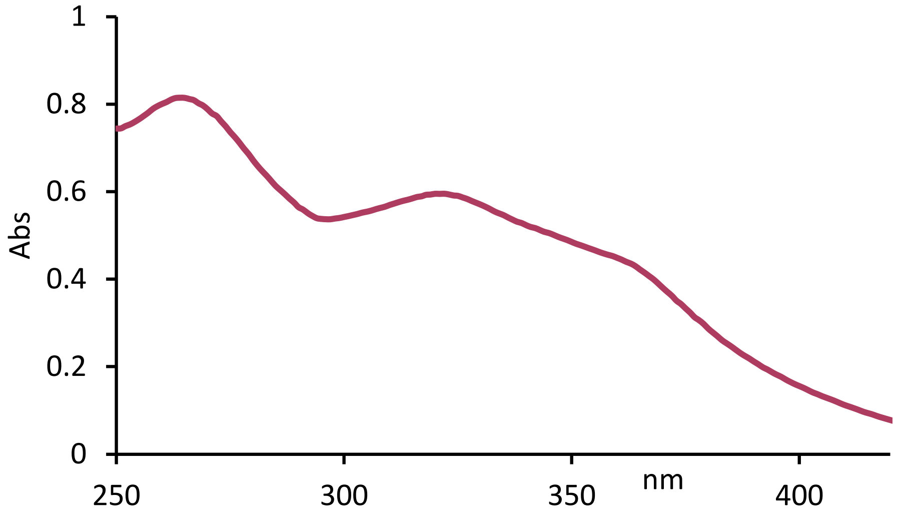

UV measurement was taken at 0.048 mM total analyte concentration in dichloroethane.

Supplementary Figure 76. UV sensing of training set 4: Mixture of 10.50 mM of (*R*)-**PEA**, 4.50 mM of (*S*)-**PEA**, 3.50 mM of (*R*)-**PMP** and 1.50 mM of (*S*)-**PMP**

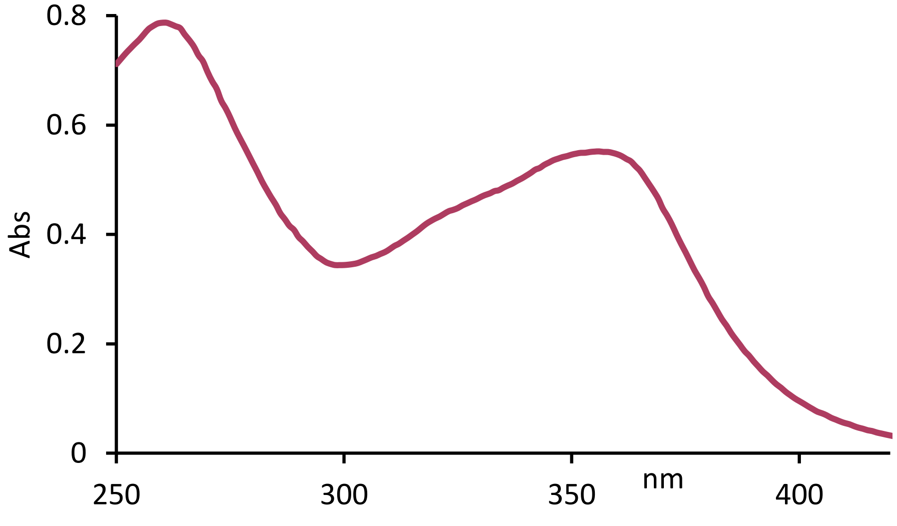

UV measurement was taken at 0.048 mM total analyte concentration in dichloroethane.

Supplementary Figure 77. UV sensing of training set 5: Mixture of 11.38 mM of (*R*)-**PEA**, 1.63 mM of (*S*)-**PEA**, 6.13 mM of (*R*)-**PMP** and 0.88 mM of (*S*)-**PMP**

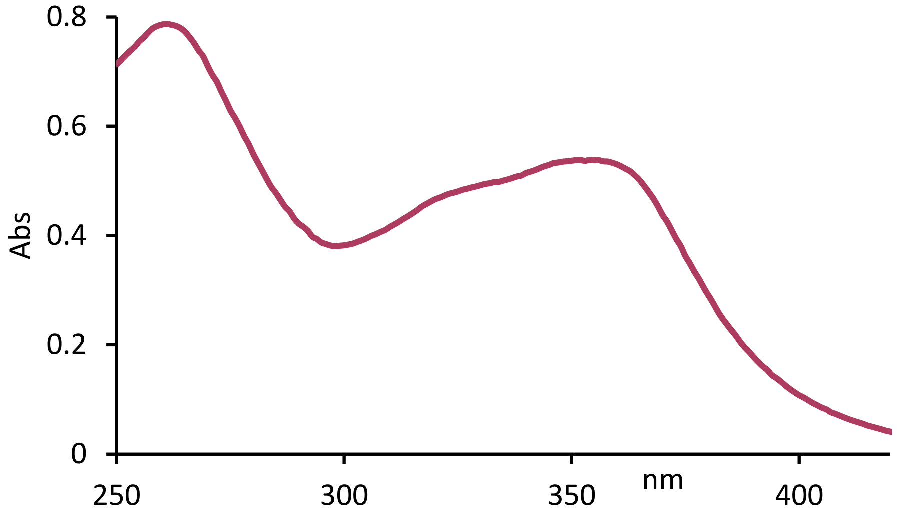

UV measurement was taken at 0.048 mM total analyte concentration in dichloroethane.

Supplementary Figure 78. UV sensing of training set 6: Mixture of 11.00 mM of (*R*)-**PEA**, 0.00 mM of (*S*)-**PEA**, 9.00 mM of (*R*)-**PMP** and 0.00 mM of (*S*)-**PMP**

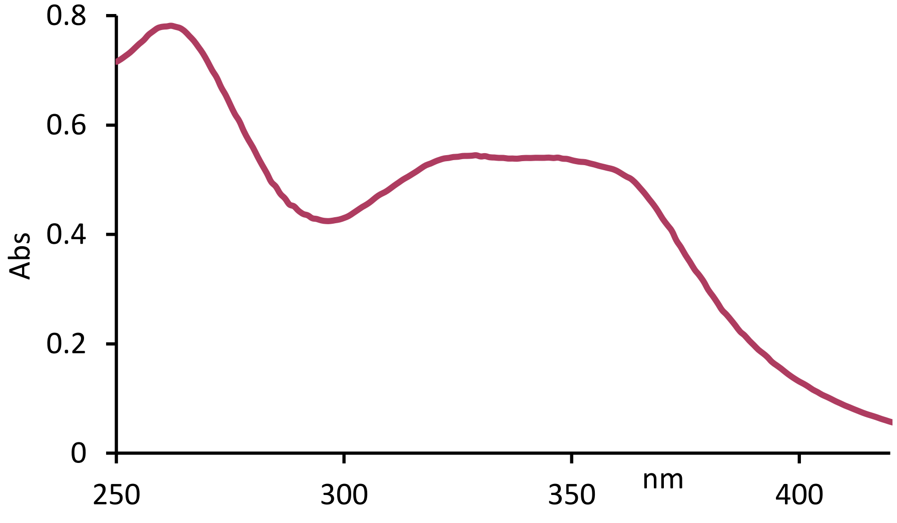

UV measurement was taken at 0.048 mM total analyte concentration in dichloroethane.

Supplementary Figure 79. UV sensing of training set 7: Mixture of 4.38 mM of (*R*)-**PEA**, 0.63 mM of (*S*)-**PEA**, 13.13 mM of (*R*)-**PMP** and 1.88 mM of (*S*)**-PMP**

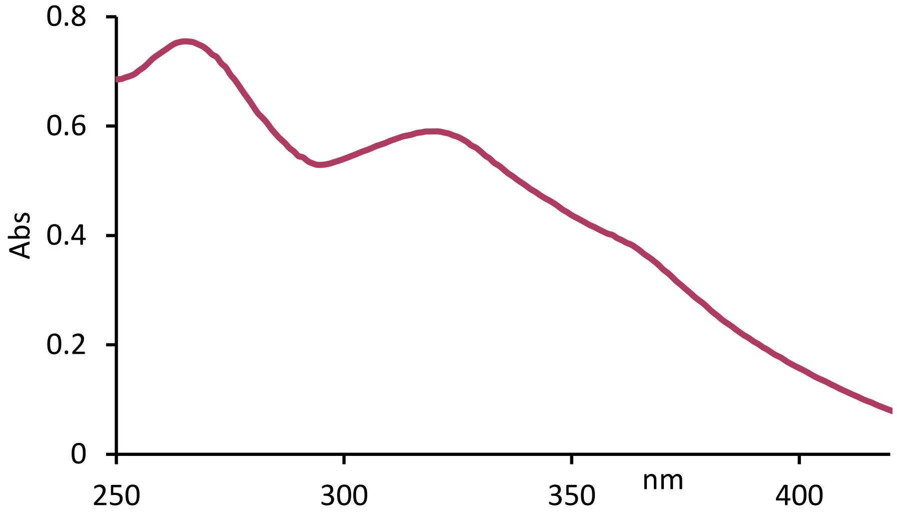

UV measurement was taken at 0.048 mM total analyte concentration in dichloroethane.

Supplementary Figure 80. UV sensing of training set 8: Mixture of 7.88 mM of (*R*)-**PEA**, 1.13 mM of (*S*)-**PEA**, 9.63 mM of (*R*)-**PMP** and 1.38 mM of (*S*)**-PMP**

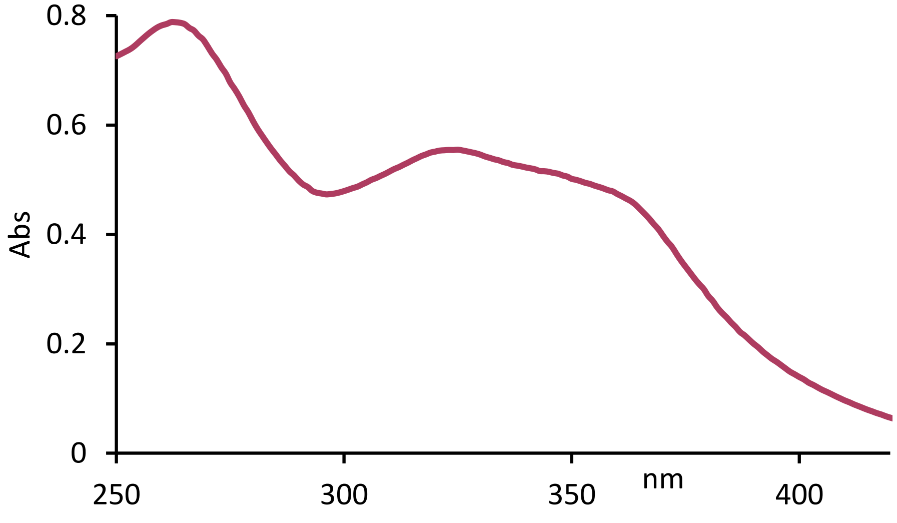

UV measurement was taken at 0.048 mM total analyte concentration in dichloroethane.

Supplementary Figure 81. UV sensing of training set 9: Mixture of 15.00 mM of (*R*)-**PEA**, 0.00 mM of (*S*)-**PEA**, 5.00 mM of (*R*)-**PMP** and 0.00 mM of (*S*)-**PMP**

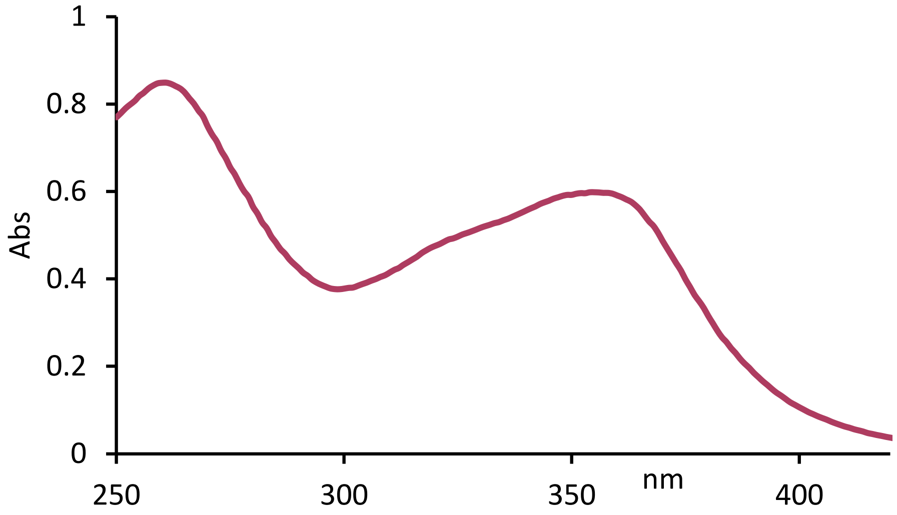

UV measurement was taken at 0.048 mM total analyte concentration in dichloroethane.

Supplementary Figure 82. CD sensing of training set 10: Mixture of 4.90 mM of (*R*)-**PEA**, 2.10 mM of (*S*)-**PEA**, 9.10 mM of (*R*)-**PMP** and 3.90 mM of (*S*)-**PMP**

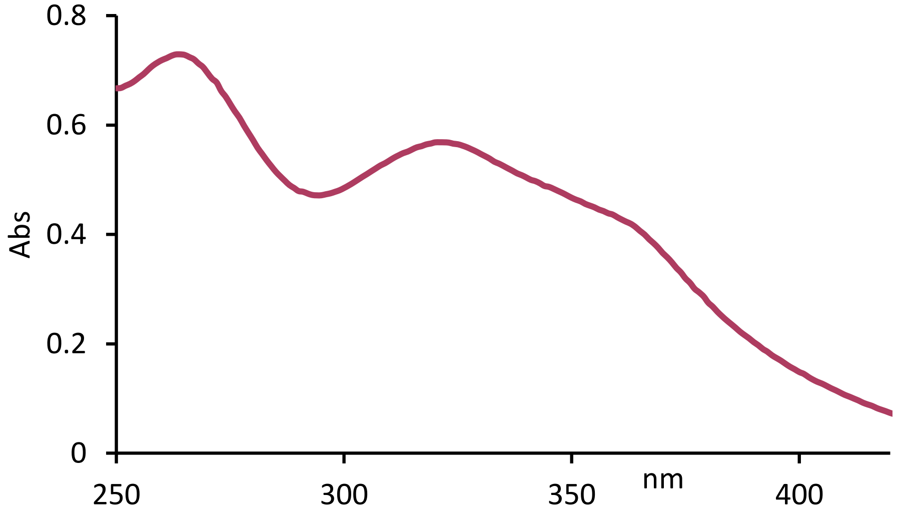

UV measurement was taken at 0.048 mM total analyte concentration in dichloroethane.

Supplementary Figure 83. UV sensing of training set 11: Mixture of 8.80 mM of (*R*)-**PEA**, 2.20 mM of (*S*)-**PEA**, 7.20 mM of (*R*)-**PMP** and 1.80 mM of (*S*)-**PMP**

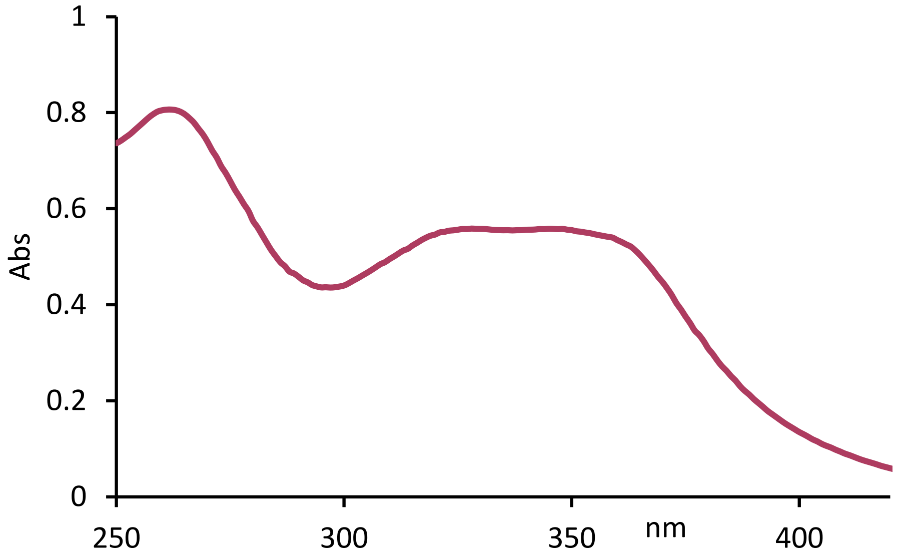

UV measurement was taken at 0.048 mM total analyte concentration in dichloroethane.

Supplementary Figure 84. UV sensing of training set 12: Mixture of 5.63 mM of (*R*)-**PEA**, 3.38 mM of (*S*)-**PEA**, 6.88 mM of (*R*)-**PMP** and 4.13 mM of (*S*)-**PMP**

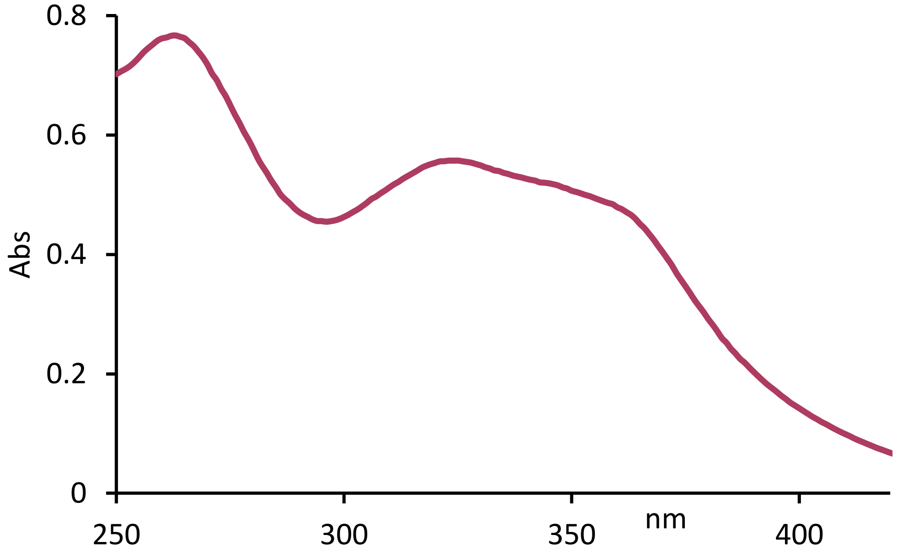

UV measurement was taken at 0.048 mM total analyte concentration in dichloroethane.

Supplementary Figure 85. UV sensing of training set 13: Mixture of 5.50 mM of (*R*)-**PEA**, 5.50 mM of (*S*)-**PEA**, 4.50 mM of (*R*)-**PMP** and 4.50 mM of (*S*)-**PMP**

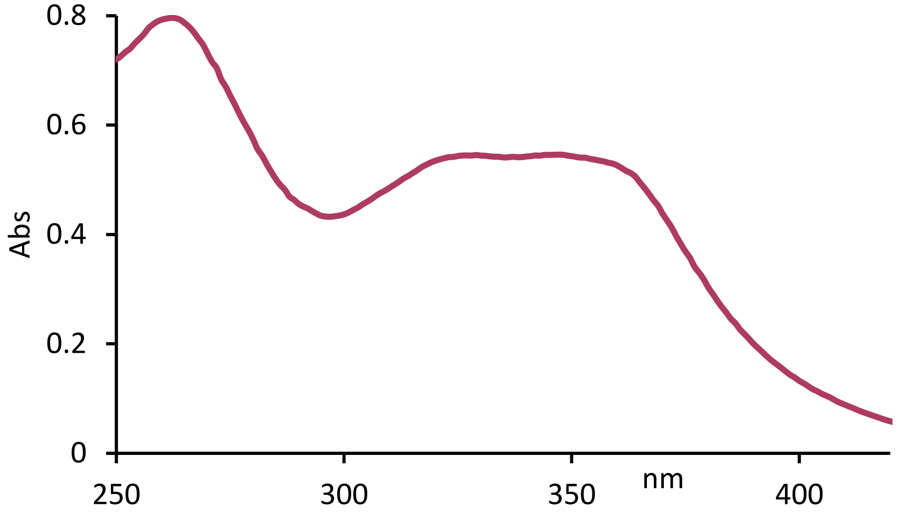

UV measurement was taken at 0.048 mM total analyte concentration in dichloroethane.

Supplementary Figure 86. UV sensing of training set 14: Mixture of 8.13 mM of (*R*)-**PEA**, 4.88 mM of (*S*)-**PEA**, 4.38 mM of (*R*)-**PMP** and 2.63 mM of (*S*)-**PMP**

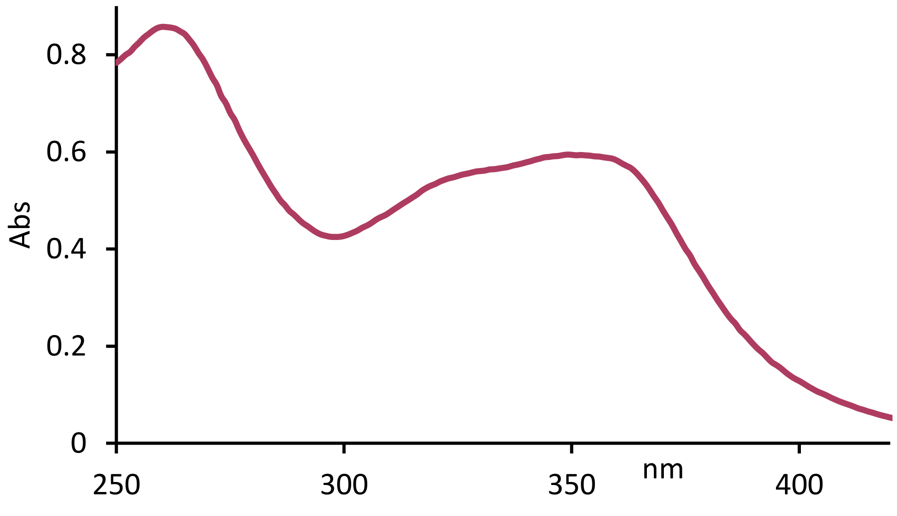

UV measurement was taken at 0.048 mM total analyte concentration in dichloroethane.

Supplementary Figure 87. UV sensing of training set 15: Mixture of 12.00 mM of (*R*)-**PEA**, 3.00 mM of (*S*)-**PEA**, 4.00 mM of (*R*)-**PMP** and 1.00 mM of (*S*)-**PMP**

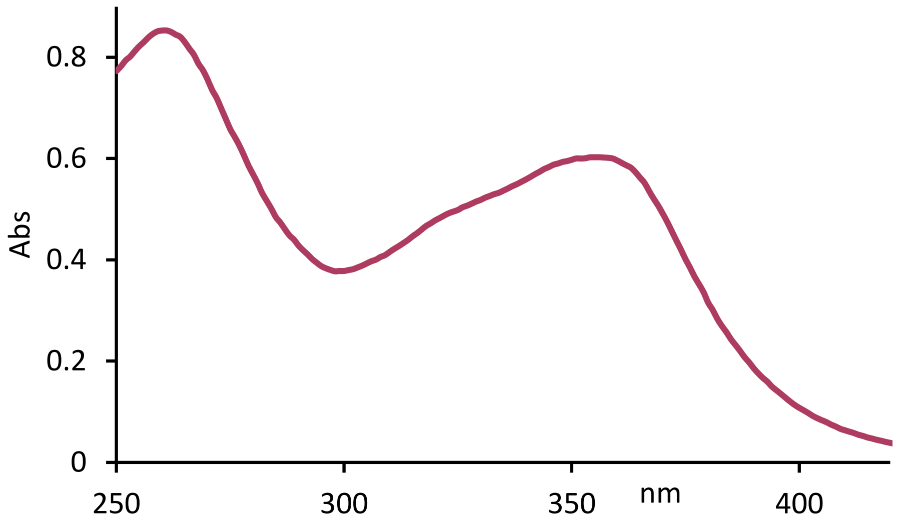

UV measurement was taken at 0.048 mM total analyte concentration in dichloroethane.

Supplementary Figure 88. UV sensing of training set 16: Mixture of 7.00 mM of (*R*)-**PEA**, 0.00 mM of (*S*)-**PEA**, 13.00 mM of (*R*)-**PMP** and 0.00 mM of (*S*)**-PMP**

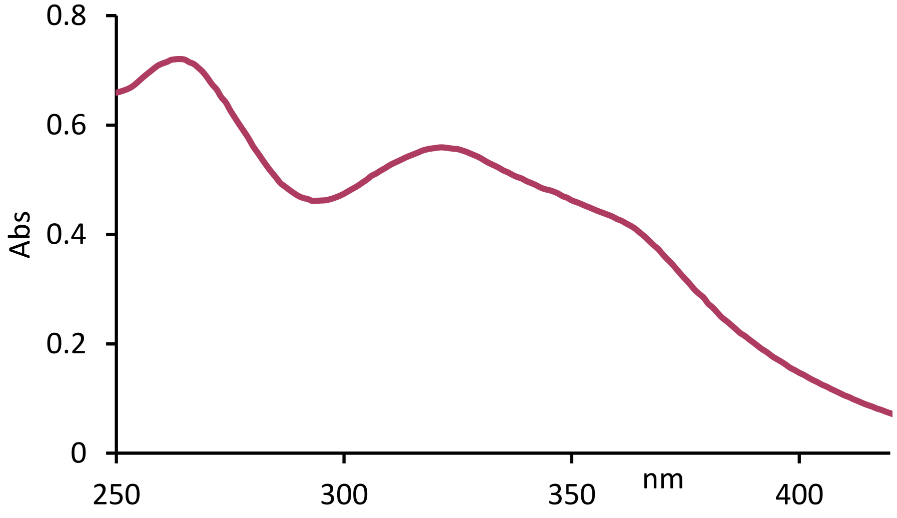

UV measurement was taken at 0.048 mM total analyte concentration in dichloroethane.

Supplementary Figure 89. UV sensing of test set 1: Mixture of 1.88 mM of (*R*)-**PEA**, 3.13 mM of (*S*)-**PEA**, 5.63 mM of (*R*)-**PMP** and 9.38 mM of (*S*)**-PMP**

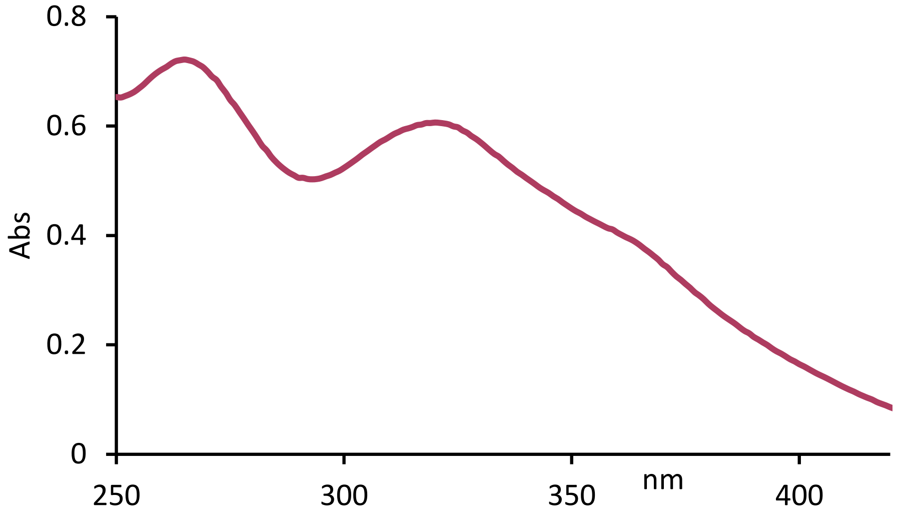

UV measurement was taken at 0.048 mM total analyte concentration in dichloroethane.

Supplementary Figure 90. UV sensing of test set 2: Mixture of 7.50 mM of (*R*)-**PEA**, 7.50 mM of (*S*)-**PEA**, 2.50 mM of (*R*)-**PMP** and 2.50 mM of (*S*)**-PMP**

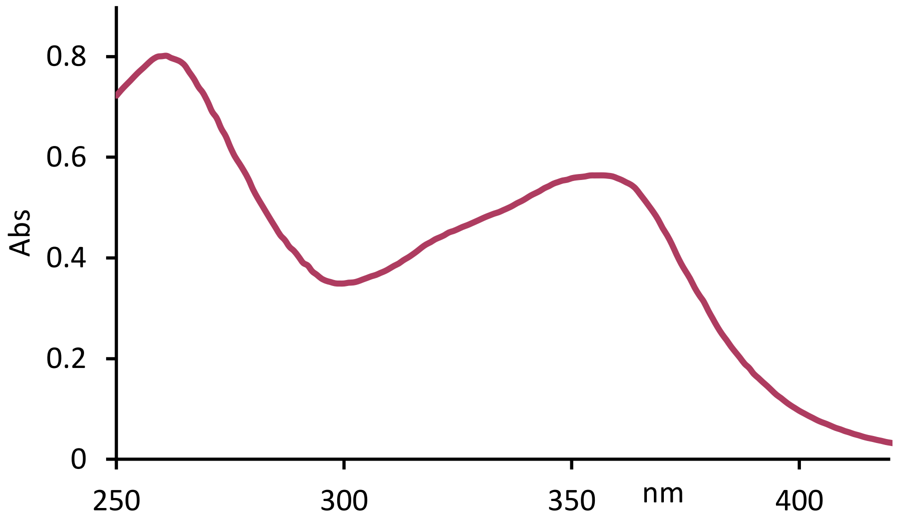

UV measurement was taken at 0.048 mM total analyte concentration in dichloroethane.

Supplementary Figure 91. UV sensing of test set 3: Mixture of 9.75 mM of (*R*)-**PEA**, 3.25 mM of (*S*)-**PEA**, 5.25 mM of (*R*)-**PMP** and 1.75 mM of (*S*)**-PMP**

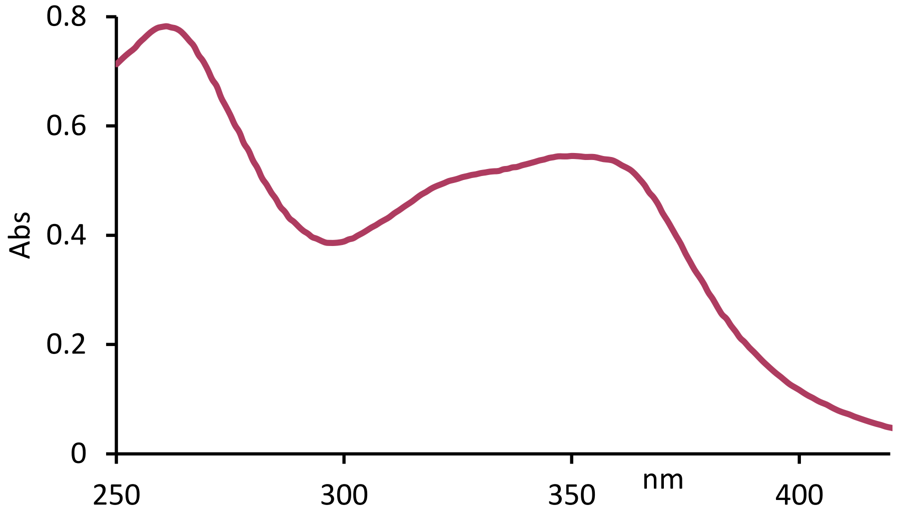

UV measurement was taken at 0.048 mM total analyte concentration in dichloroethane.

Supplementary Figure 92. UV sensing of test set 4: Mixture of 3.75 mM of (*R*)-**PEA**, 1.25 mM of (*S*)-**PEA**, 11.25 mM of (*R*)-**PMP** and 3.75 mM of (*S*)-**PMP**

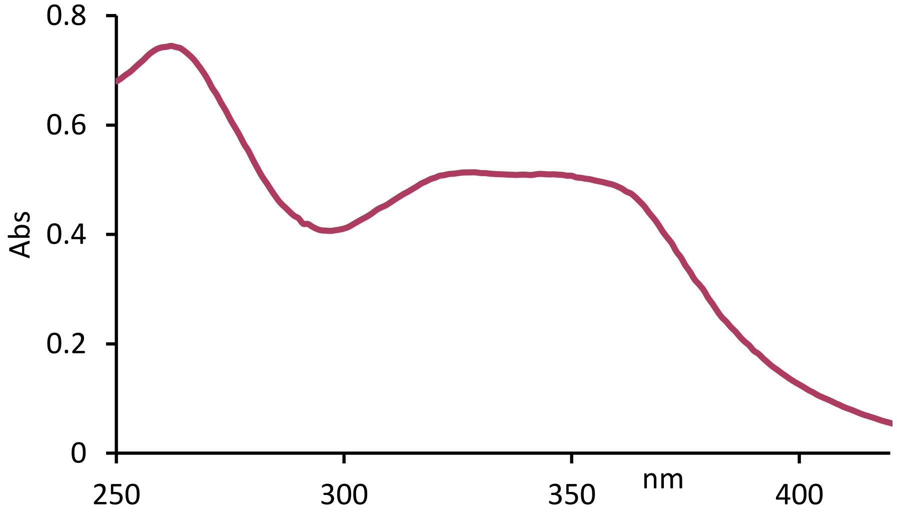

UV measurement was taken at 0.048 mM total analyte concentration in dichloroethane.

Supplementary Figure 93. UV sensing of test set 5: Mixture of 6.75 mM of (*R*)-**PEA**, 2.25 mM of (*S*)-**PEA**, 8.25 mM of (*R*)-**PMP** and 2.75 mM of (*S*)-**PMP**

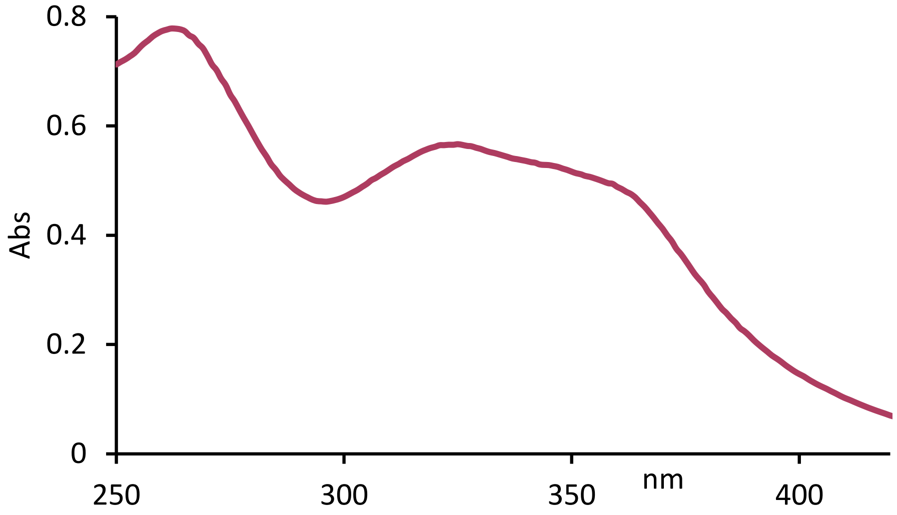

UV measurement was taken at 0.048 mM total analyte concentration in dichloroethane.

Supplementary Figure 94. Actual versus predicted concentration of each enantiomer of **PEA** and **PMP** with chemometric chirality sensing

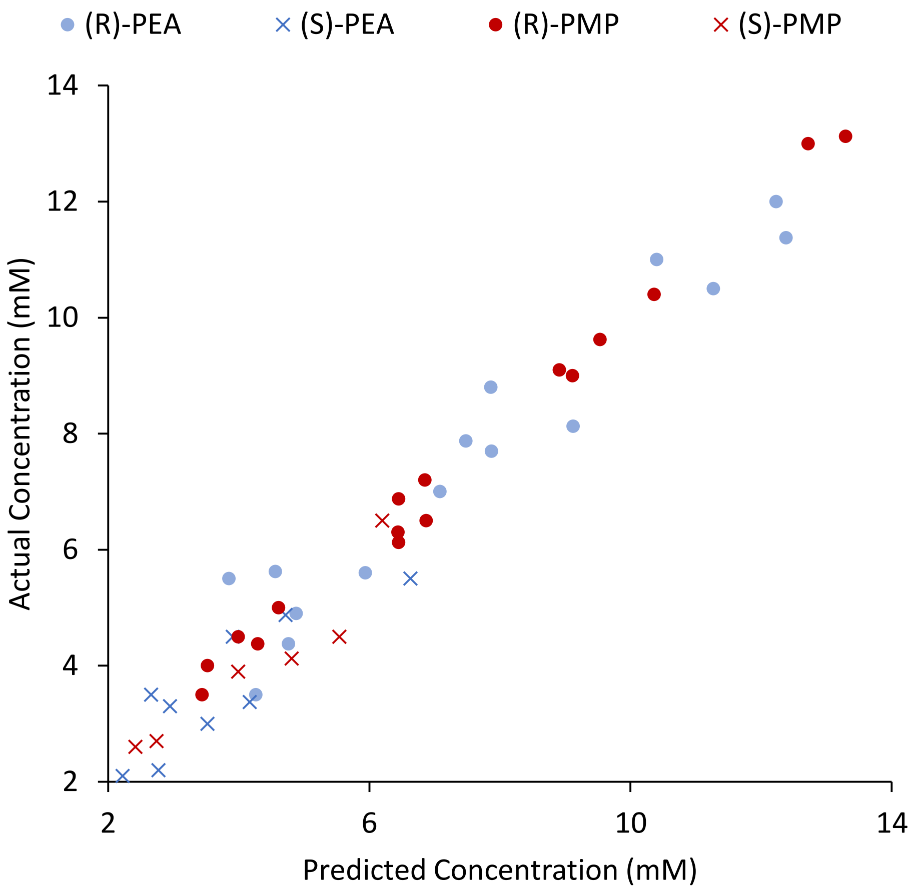

Supplementary Figure 95. Heat map for the 16 training sets and the 5 samples. The colors correspond to the absolute error of predicted [mM] versus actual [mM]. The numbers are the actual concentration of each analyte

**Sample**	(*R*)-**PEA** (mM)	(*S*)-**PEA** (mM)	(*R*)-**PMP** (mM)	(*S*)-**PMP** (mM)
Training Set 1	5.60	1.40	10.40	2.60
Training Set 2	7.70	3.30	6.30	2.70
Training Set 3	3.50	3.50	6.50	6.50
Training Set 4	10.50	4.50	3.50	1.50
Training Set 5	11.38	1.63	6.13	0.88
Training Set 6	11.00	0.00	9.00	0.00
Training Set 7	4.38	0.63	13.13	1.88
Training Set 8	7.88	1.13	9.63	1.38
Training Set 9	15.00	0.00	5.00	0.00
Training Set 10	4.90	2.10	9.10	3.90
Training Set 11	8.80	2.20	7.20	1.80
Training Set 12	5.63	3.38	6.88	4.13
Training Set 13	5.50	5.50	4.50	4.50
Training Set 14	8.13	4.88	4.38	2.63
Training Set 15	12.00	3.00	4.00	1.00
Training Set 16	7.00	0.00	13.00	0.00
Sample 1	1.88	3.13	5.63	9.38
Sample 2	7.50	7.50	2.50	2.50
Sample 3	9.75	3.25	5.25	1.75
Sample 4	3.75	1.25	11.25	3.75
Sample 5	6.75	2.25	8.25	2.75

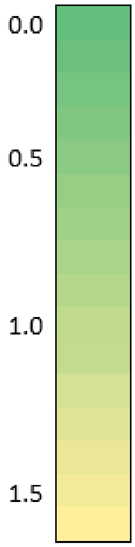

Supplementary Figure 96. R^2 results for cross-validation (CV) and test sets for quaternary mixture sensing

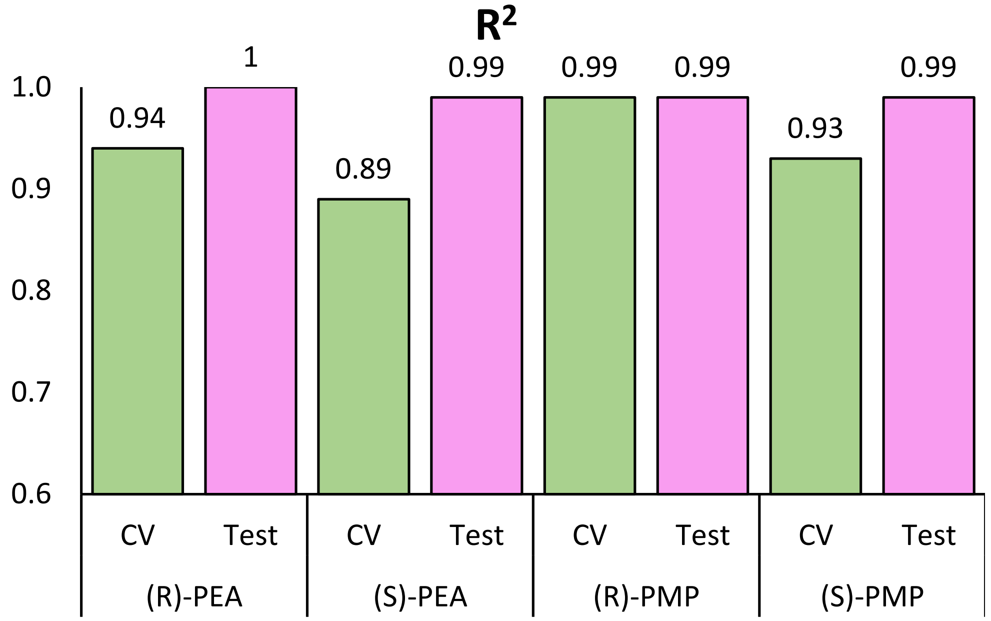

Supplementary Figure 97. RMSE results for cross-validation (CV) and test set for quaternary mixture sensing

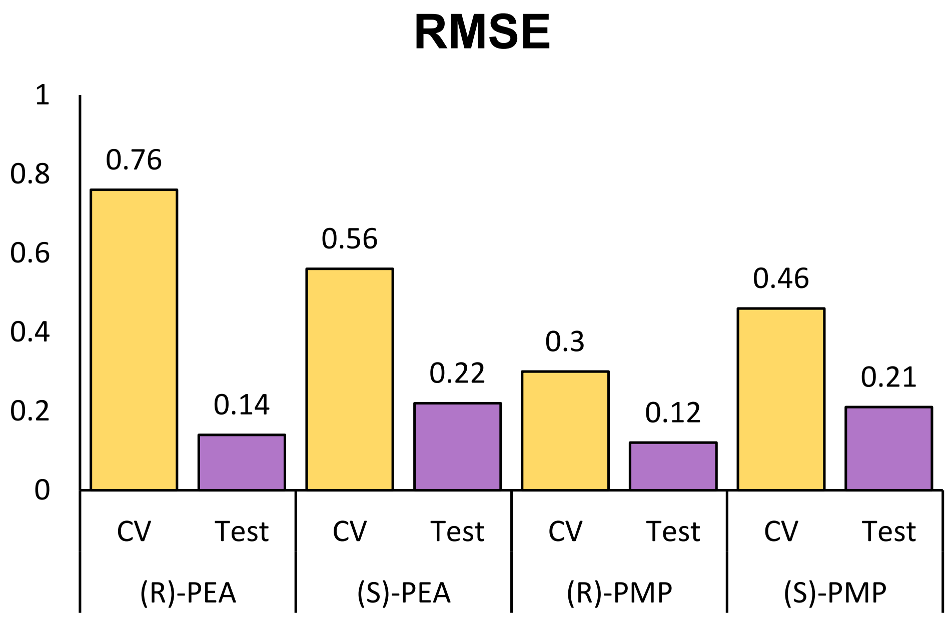

Supplementary Figure 98. CD sensing of training set 1: Mixture of 5.50 mM of *(R)*-**PEA**, 0.00 mM of (*S*)-**PEA**, 4.5 mM of (*R*)-**PMP**, 0.00 mM of (*S*)-**PMP**, 5.50 mM of (*R*)-**PPA**, 4.50 mM of (*S*)-**PPA**, 4.5 mM of (*R*)-**PGL** and 0.00 mM of (*S*)-**PGL**

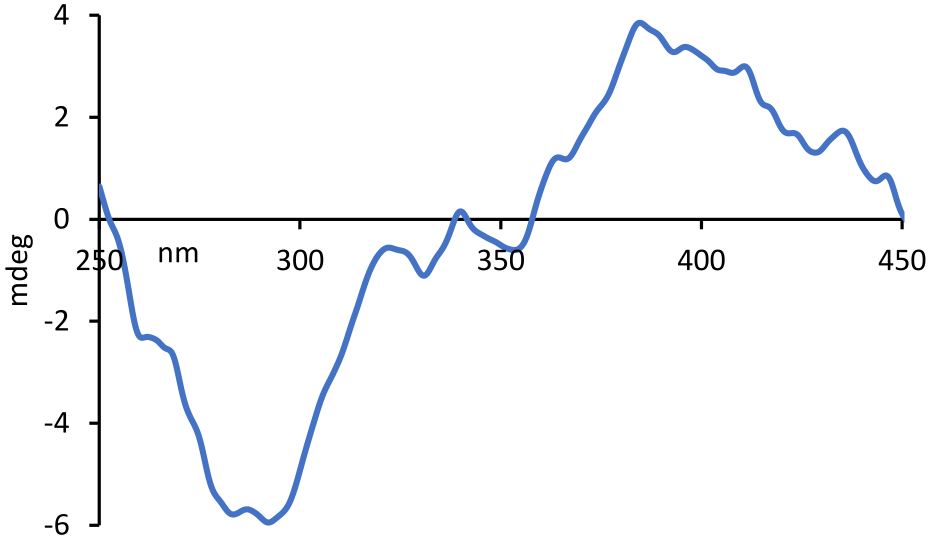

CD measurement was taken at 0.05 mM total analyte concentration in dichloroethane.

Supplementary Figure 99. CD sensing of training set 2: Mixture of 1.88 mM of (*R*)-**PEA**, 0.63 mM of (*S*)-**PEA**, 5.63 mM of (*R*)-**PMP**, 1.88 mM of (*S*)-**PMP**, 1.88 mM of (*R*)-**PPA**, 0.63 mM of (*S*)-**PPA**, 5.63 mM of (*R*)-**PGL** and 1.88 mM of (*S*)-**PGL**

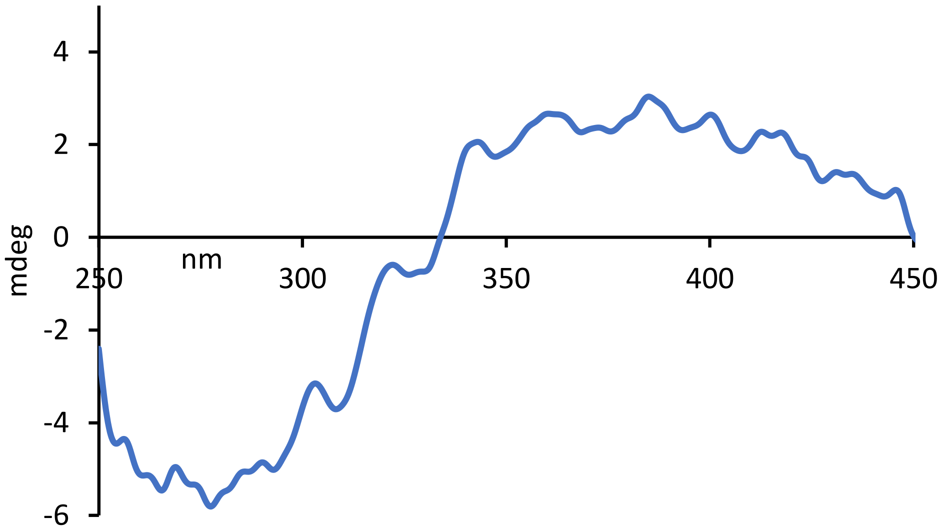

CD measurement was taken at 0.05 mM total analyte concentration in dichloroethane.

Supplementary Figure 100. CD sensing of training set 3: Mixture of 0.70 mM of (*R*)-**PEA**, 2.80 mM of (*S*)-**PEA**, 1.30 mM of (*R*)-**PMP**, 5.20 mM of (*S*)**-PMP**, 0.70 mM of (*R*)-**PPA**, 2.80 mM of (*S*)-**PPA**, 1.30 mM of (*R*)-**PGL** and 5.20 mM of (*S*)-**PGL**

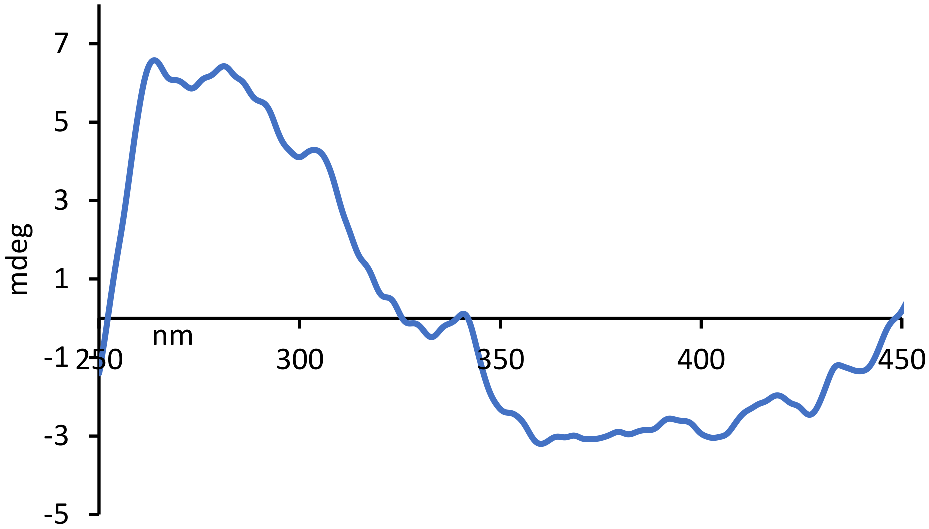

CD measurement was taken at 0.05 mM total analyte concentration in dichloroethane.

Supplementary Figure 101. CD sensing of training set 4: Mixture of 2.80 mM of (*R*)-**PEA**, 0.70 mM of (*S*)-**PEA**, 5.20 mM of (*R*)-**PMP**,1.30 mM of (*S*)**-PMP**, 2.80 mM of (*R*)-**PPA**, 0.70 mM of (*S*)-**PPA**, 5.20 mM of (*R*)-**PGL** and 1.30 mM of (*S*)-**PGL**

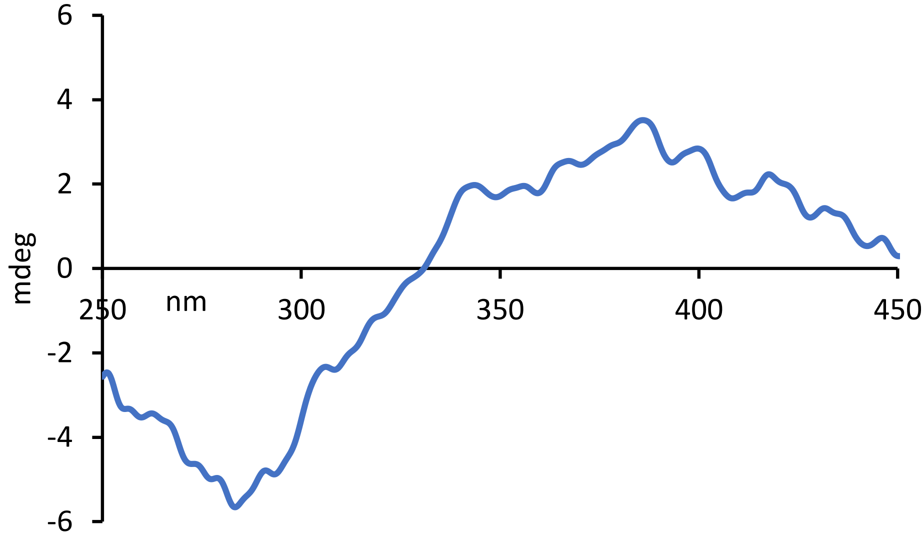

CD measurement was taken at 0.05 mM total analyte concentration in dichloroethane.

Supplementary Figure 102. CD sensing of training set 5: Mixture of 2.19 mM of (*R*)-**PEA**, 0.31 mM of (*S*)-**PEA**, 6.56 mM of (*R*)-**PMP**, 0.94 mM of (*S*)**-PMP**, 2.19 mM of (*R*)-**PPA**, 0.31 mM of (*S*)-**PPA**, 6.56 mM of (*R*)-**PGL** and 0.94 mM of (*S*)**-PGL**

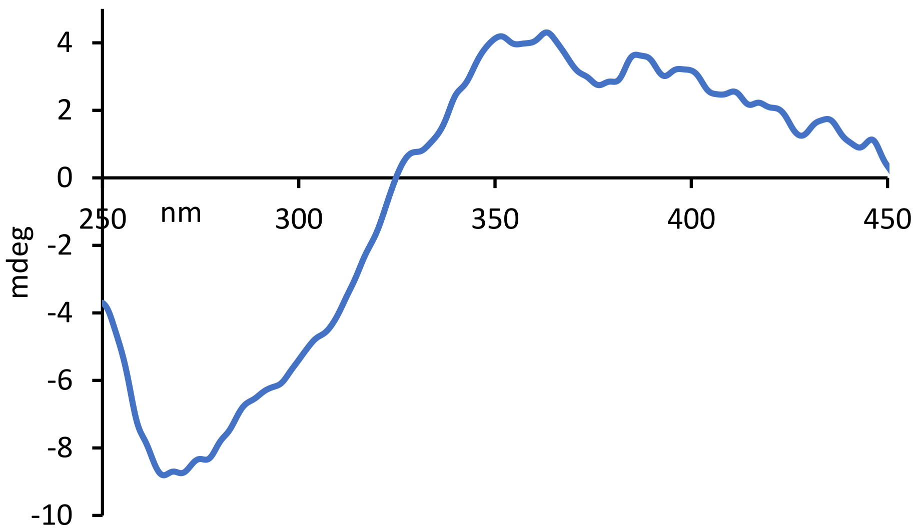

CD measurement was taken at 0.05 mM total analyte concentration in dichloroethane.

Supplementary Figure 103. CD sensing of training set 6: Mixture of 4.40 mM of (*R*)-**PEA**, 1.10 mM of (*S*)-**PEA**, 3,60 mM of (*R*)-**PMP**, 0.90 mM of (*S*)**-PMP**, 4.40 mM of (*R*)-**PPA**, 1.10 mM of (*S*)-**PPA**, 3.60 mM of (*R*)-**PGL** and 0.90 mM of (*S*)**-PGL**

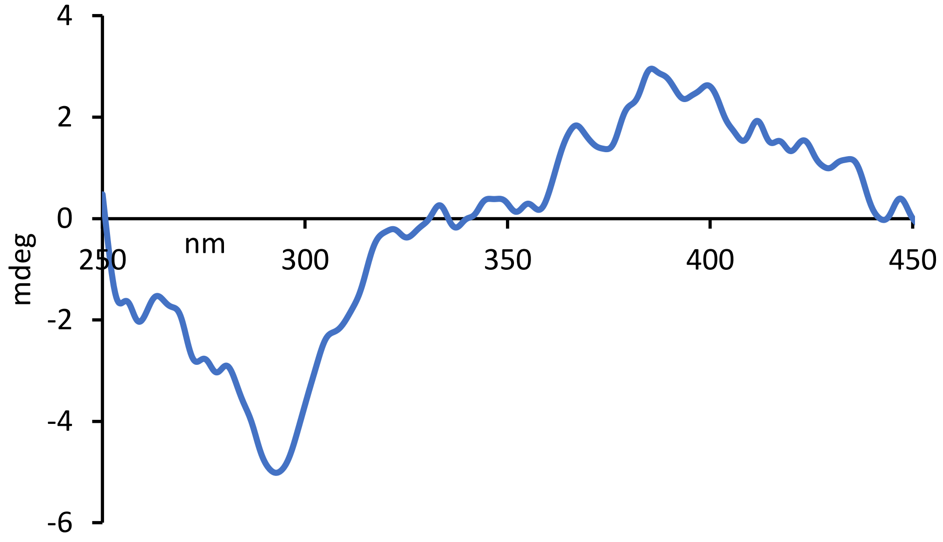

CD measurement was taken at 0.05 mM total analyte concentration in dichloroethane.

Supplementary Figure 104. CD sensing of training set 8: Mixture of 1.75 mM of (*R*)-**PEA**, 1.75 mM of (*S*)-**PEA**, 3.25 mM of (*R*)-**PMP**, 3.25 mM of (*S*)**-PMP**, 1.75 mM of (*R*)-**PPA**, 1.75 mM of (*S*)-**PPA**, 3.25 mM of (*R*)-**PGL** and 3.25 mM of (*S*)**-PGL**

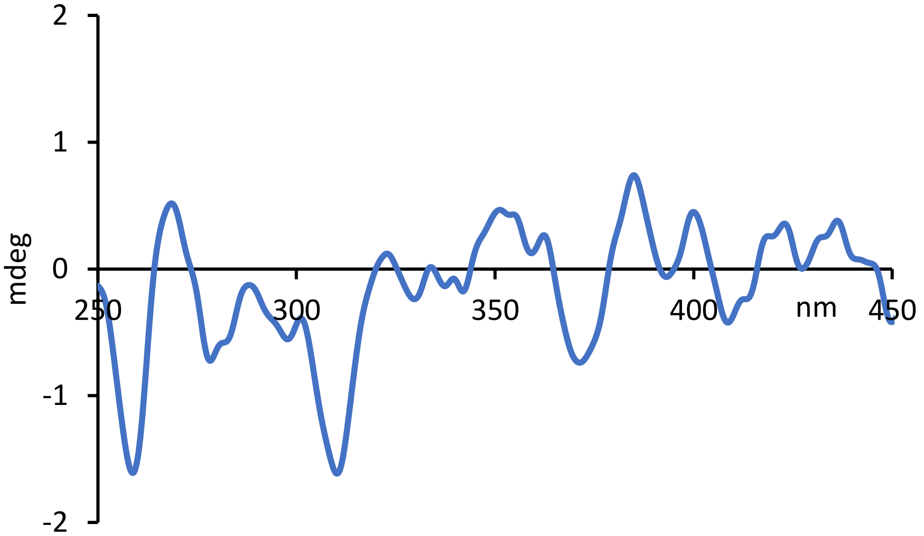

CD measurement was taken at 0.05 mM total analyte concentration in dichloroethane.

Supplementary Figure 105. CD sensing of training set 9: Mixture of 2.45 mM of (*R*)-**PEA**, 1.05 mM of (*S*)-**PEA**, 4.55 mM of (*R*)-**PMP** and 1.95 mM of (*S*)**-PMP**, 2.45 mM of (*R*)-**PPA**, 1.05 mM of (*S*)-**PPA**, 4.55 mM of (*R*)-**PGL** and 1.95 mM of (*S*)**-PGL**

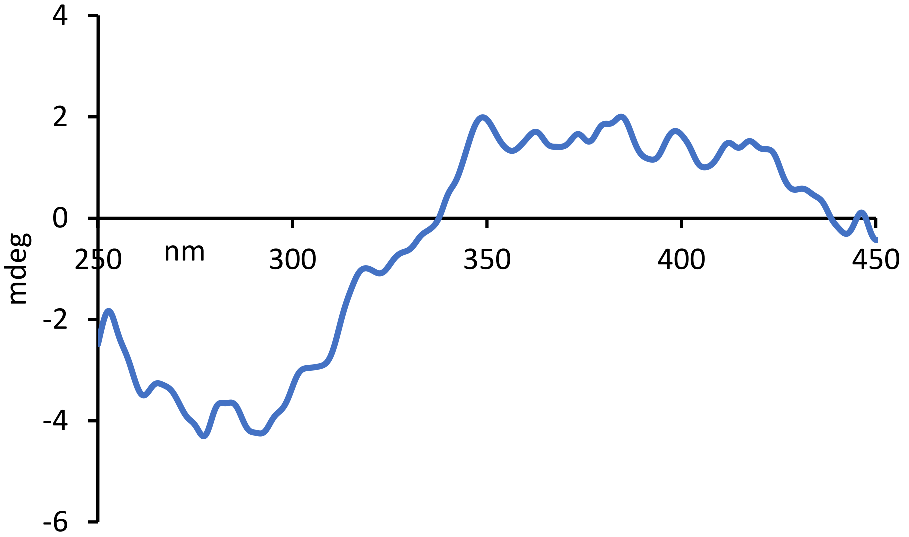

CD measurement was taken at 0.05 mM total analyte concentration in dichloroethane.

Supplementary Figure 106. CD sensing of training set 10: Mixture of 0.75 mM of (*R*)-**PEA**, 1.75 mM of (*S*)-**PEA**, 2.25 mM of (*R*)-**PMP**, 5.25 mM of (*S*)-**PMP**, 0.75 mM of (*R*)-**PPA**, 1.75 mM of (*S*)-**PPA**, 2.25 mM of (*R*)-**PGL** and 5.25 mM of (*S*)-**PGL**

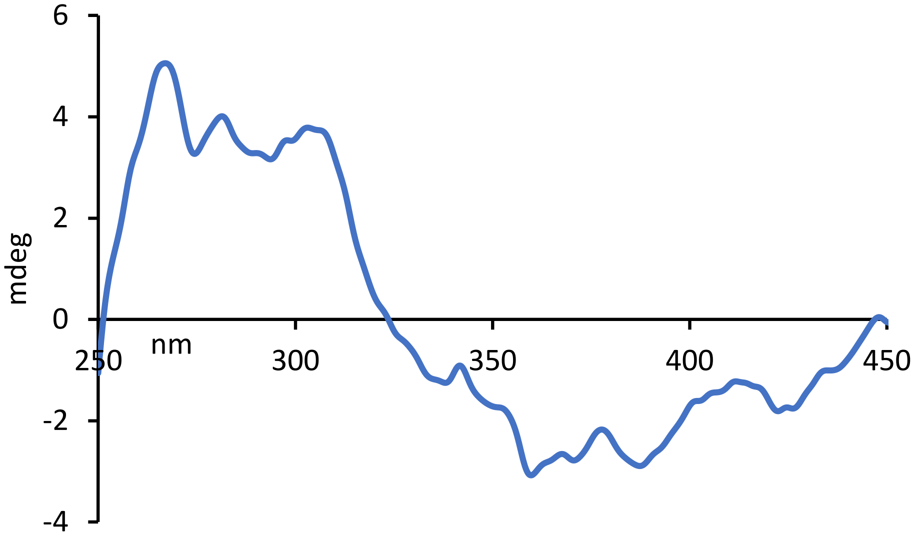

CD measurement was taken at 0.05 mM total analyte concentration in dichloroethane.

Supplementary Figure 107. CD sensing of training set 11: Mixture of 3.75 mM of (*R*)-**PEA**, 3.75 mM of (*S*)-**PEA**, 1.25 mM of (*R*)-**PMP**, 1.25 mM of (*S*)-**PMP**, 3.75 mM of (*R*)-**PPA**, 3.75 mM of (*S*)-**PPA**, 1.25 mM of (*R*)-**PGL** and 1.25 mM of (*S*)-**PGL**

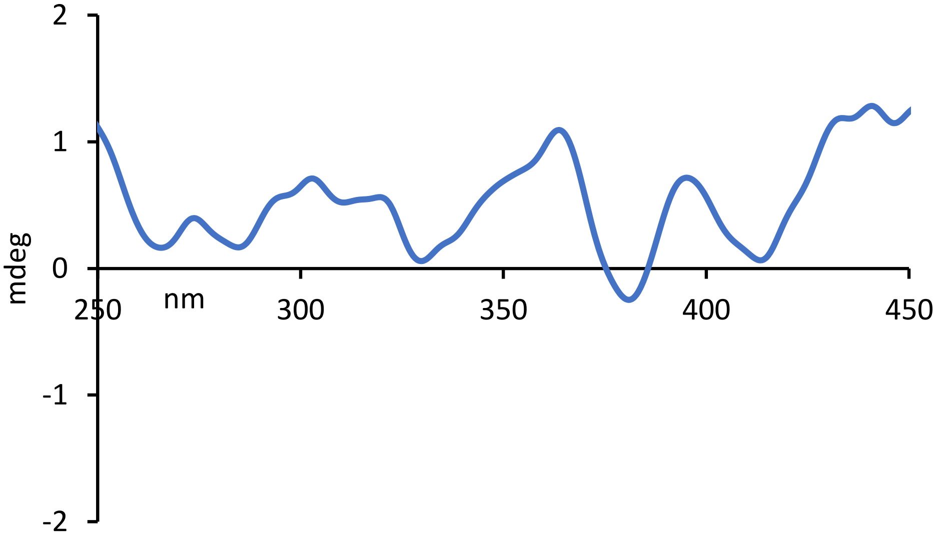

CD measurement was taken at 0.05 mM total analyte concentration in dichloroethane.

Supplementary Figure 108. CD sensing of training set 12: Mixture of 0.63 mM of (*R*)-**PEA**, 1.88 mM of (*S*)-**PEA**, 1.88 mM of (*R*)-**PMP**, 5.63 mM of (*S*)**-PMP**, 0.63 mM of (*R*)-**PPA**, 1.88 mM of (*S*)-**PPA**, 1.88 mM of (*R*)-**PGL** and 5.63 mM of (*S*)**-PGL**

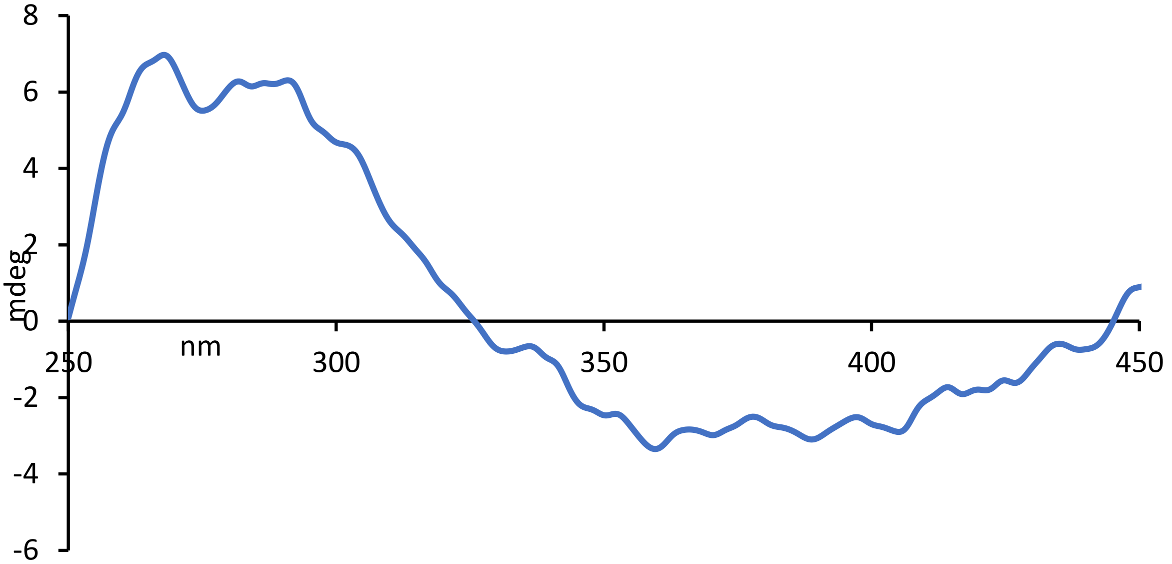

CD measurement was taken at 0.05 mM total analyte concentration in dichloroethane.

Supplementary Figure 109. CD sensing of training set 13: Mixture of 5.25 mM of (*R*)-**PEA**, 2.25 mM of (*S*)-**PEA**, 1.75 mM of (*R*)-**PMP**, 0.75 mM of (*S*)**-PMP**, 5.25 mM of (*R*)-**PPA**, 2.25 mM of (*S*)-**PPA**, 1.75 mM of (*R*)-**PGL** and 0.75 mM of (*S*)**-PGL**

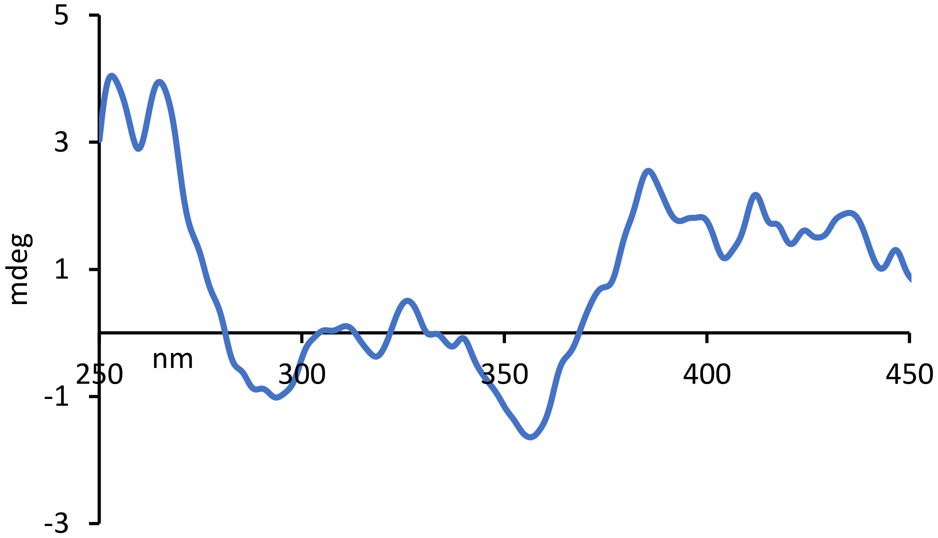

CD measurement was taken at 0.05 mM total analyte concentration in dichloroethane.

Supplementary Figure 110. CD sensing of training set 14: Mixture of 0.00 mM of (*R*)-**PEA**, 4.50 mM of (*S*)-**PEA**, 0.00 mM of (*R*)-**PMP**, 5.50 mM of (*S*)**-PMP**, 0.00 mM of (*R*)-**PPA**, 4.50 mM of (*S*)-**PPA**, 0.00 mM of (*R*)-**PGL** and 5.50 mM of (*S*)**-PGL**

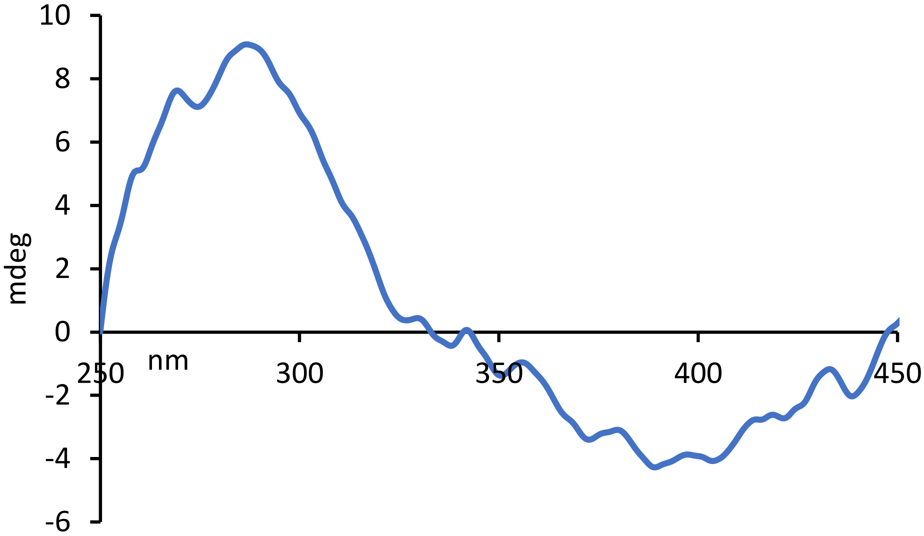

CD measurement was taken at 0.05 mM total analyte concentration in dichloroethane.

Supplementary Figure 111. CD sensing of training set 15: Mixture of 7.50 mM of (*R*)-**PEA**, 0.00 mM of (*S*)-**PEA**, 2.50 mM of (*R*)-**PMP**, 0.00 mM of (*S*)**-PMP**, 7.50 mM of (*R*)-**PPA**, 0.00 mM of (*S*)-**PPA**, 2.50 mM of (*R*)-**PGL** and 0.00 mM of (*S*)**-PGL**

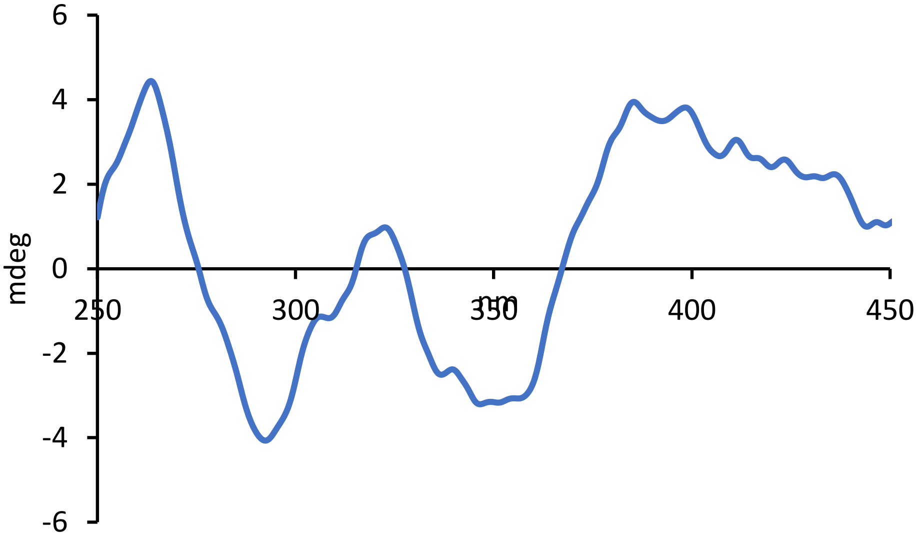

CD measurement was taken at 0.05 mM total analyte concentration in dichloroethane.

Supplementary Figure 112. CD sensing of training set 16: Mixture of 2.81 mM of (*R*)-**PEA**, 1.69 mM of (*S*)-**PEA**, 3.44 mM of (*R*)-**PMP**, 2.06 mM of (*S*)**-PMP**, 2.81 mM of (*R*)-**PPA**, 1.69 mM of (*S*)-**PPA**, 3.44 mM of (*R*)-**PGL** and 2.06 mM of (*S*)**-PGL**

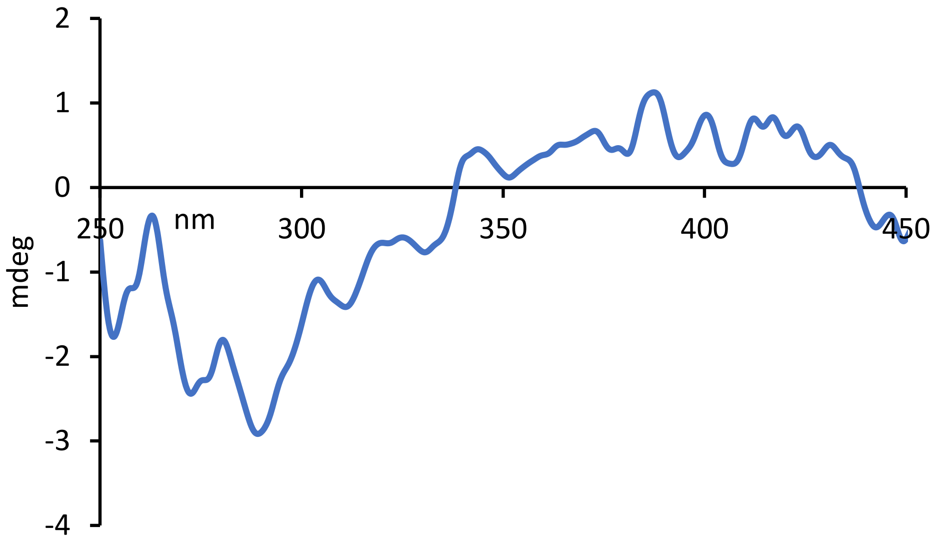

CD measurement was taken at 0.05 mM total analyte concentration in dichloroethane.

Supplementary Figure 113. CD sensing of training set 17: Mixture of 2.75 mM of (*R*)-**PEA**, 2.75 mM of (*S*)-**PEA**, 2.25 mM of (*R*)-**PMP**, 2.25 mM of (*S*)**-PMP**, 2.75 mM of (*R*)-**PPA**, 2.75 mM of (*S*)-**PPA**, 2.25 mM of (*R*)-**PGL** and 2.25 mM of (*S*)**-PGL**

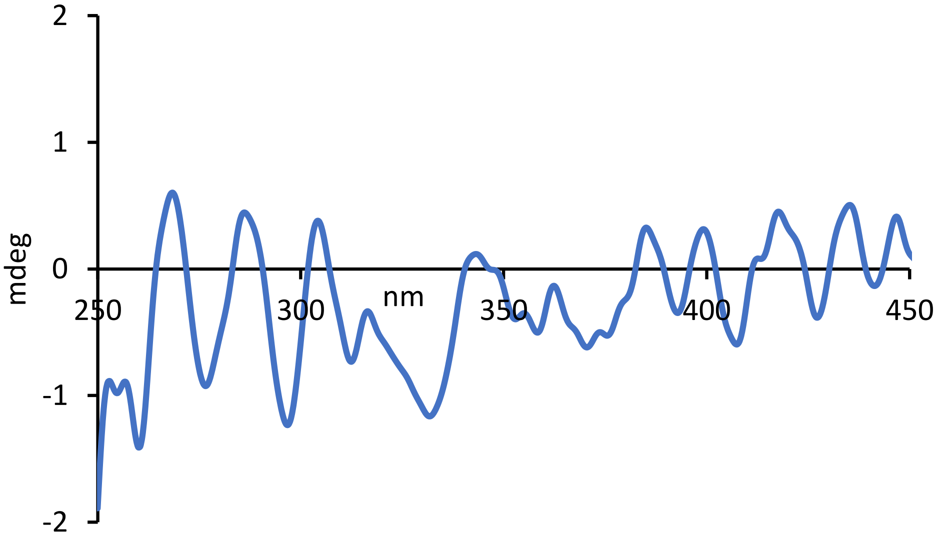

CD measurement was taken at 0.05 mM total analyte concentration in dichloroethane.

Supplementary Figure 114. CD sensing of training set 18: Mixture of 4.06 mM of (*R*)-**PEA**, 2.44 mM of (*S*)-**PEA**, 2.19 mM of (*R*)-**PMP**, 1.31 mM of (*S*)**-PMP**, 4.06 mM of (*R*)-**PPA**, 2.44 mM of (*S*)-**PPA**, 2.19 mM of (*R*)-**PGL** and 1.31 mM of (*S*)**-PGL**

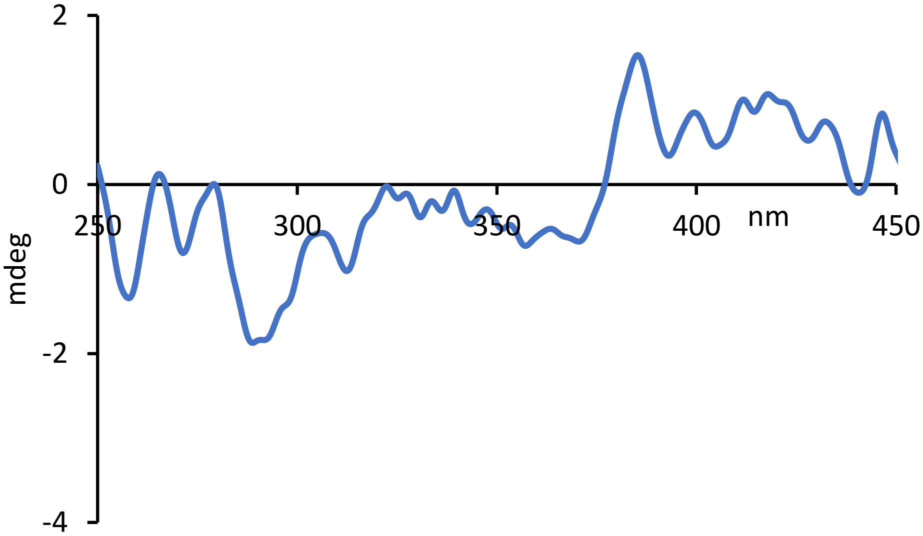

CD measurement was taken at 0.05 mM total analyte concentration in dichloroethane.

Supplementary Figure 115. CD sensing of training set 19: Mixture of 6.00 mM of (*R*)-**PEA**, 1.50 mM of (*S*)-**PEA**, 2.00 mM of (*R*)-**PMP**, 0.50 mM of (*S*)**-PMP**, 6.00 mM of (*R*)-**PPA**, 1.50 mM of (*S*)-**PPA**, 2.00 mM of (*R*)-**PGL** and 0.50 mM of (*S*)**-PGL**

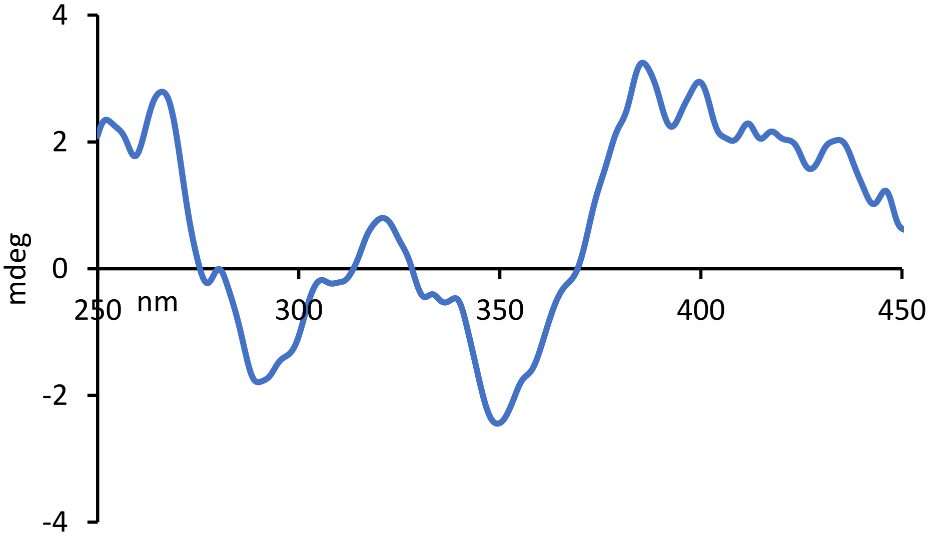

CD measurement was taken at 0.05 mM total analyte concentration in dichloroethane.

Supplementary Figure 116. CD sensing of training set 20: Mixture of 3.50 mM of (*R*)-**PEA**, 0.00 mM of (*S*)-**PEA**, 6.50 mM of (*R*)-**PMP**, 0.00 mM of (*S*)**-PMP**, 3.50 mM of (*R*)-**PPA**, 0.00 mM of (*S*)-**PPA**, 6.50 mM of (*R*)-**PGL** and 0.00 mM of (*S*)**-PGL**

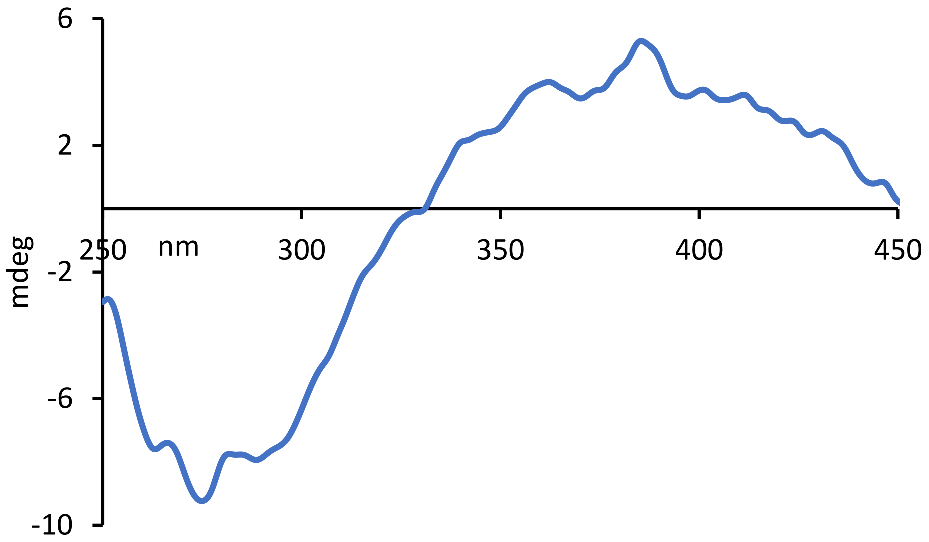

CD measurement was taken at 0.05 mM total analyte concentration in dichloroethane.

Supplementary Figure 117. CD sensing of test set 1: Mixture of 3.38 mM of (*R*)-**PEA**, 1.13 mM of (*S*)-**PEA**, 4.13 mM of (*R*)-**PMP**, 1.38 mM of (*S*)**-PMP**, 3.38 mM of (*R*)-**PPA**, 1.13 mM of (*S*)-**PPA**, 4.13 mM of (*R*)-**PGL** and 1.38 mM of (*S*)**-PGL**

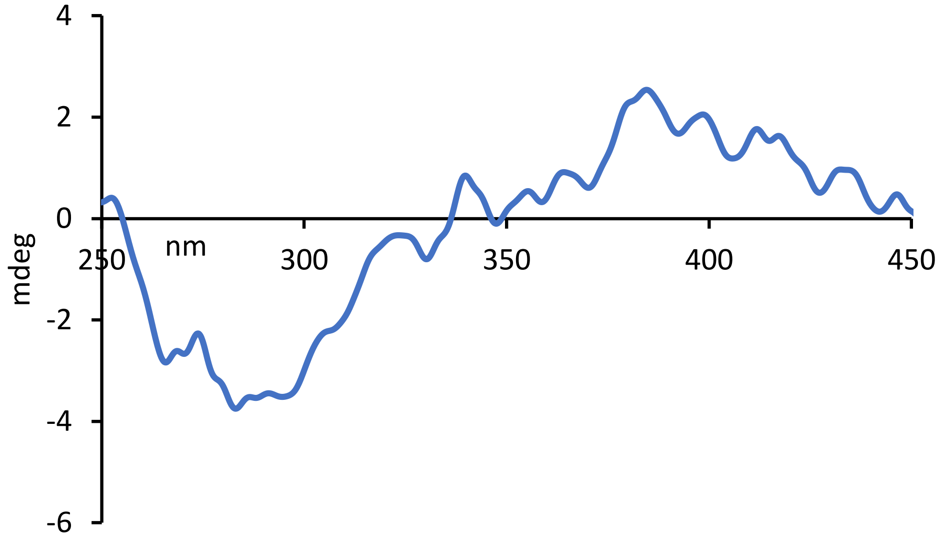

CD measurement was taken at 0.05 mM total analyte concentration in dichloroethane.

Supplementary Figure 118. CD sensing of test set 2: Mixture of 5.69 mM of (*R*)-**PEA**, 0.81 mM of (*S*)-**PEA**, 3.06 mM of (*R*)-**PMP**, 0.44 mM of (*S*)**-PMP**, 5.69 mM of (*R*)-**PPA**, 0.81 mM of (*S*)-**PPA**, 3.06 mM of (*R*)-**PGL** and 0.44 mM of (*S*)**-PGL**

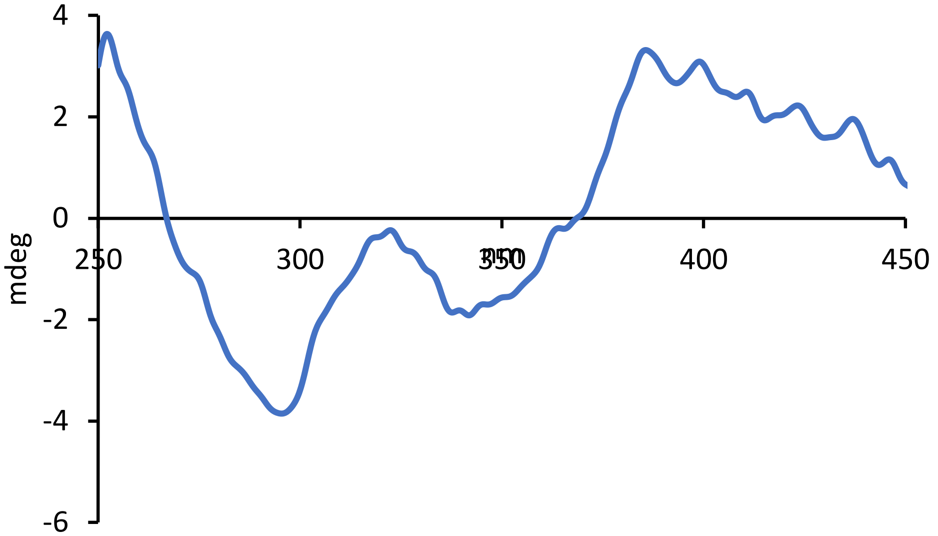

CD measurement was taken at 0.05 mM total analyte concentration in dichloroethane.

Supplementary Figure 119. CD sensing of test set 3: Mixture of 0.94 mM of (*R*)-**PEA**, 1.56 mM of (*S*)-**PEA**, 2.81 mM of (*R*)-**PMP**, 4.69 mM of (*S*)**-PMP**, 0.94 mM of (*R*)-**PPA**, 1.56 mM of (*S*)-**PPA**, 2.81 mM of (*R*)-**PGL** and 4.69 mM of (*S*)**-PGL**

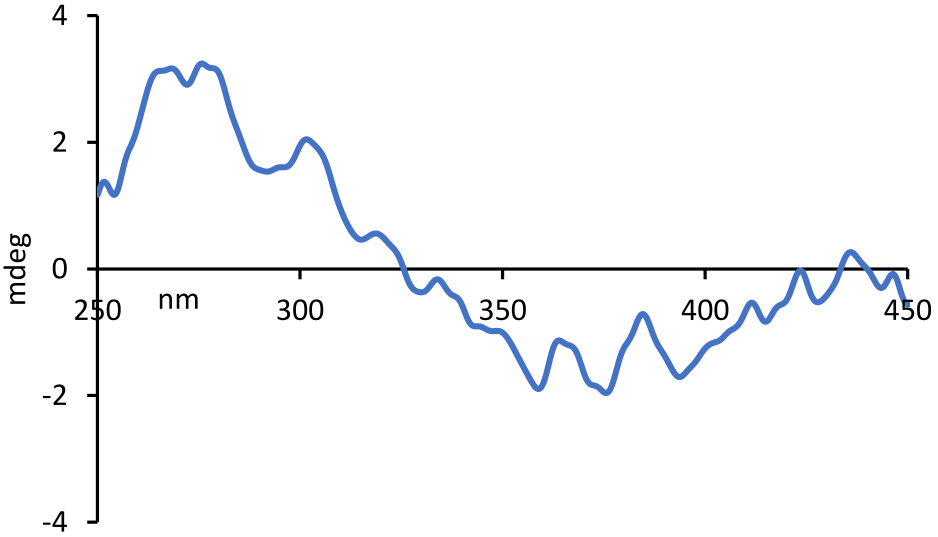

CD measurement was taken at 0.05 mM total analyte concentration in dichloroethane.

Supplementary Figure 120. CD sensing of test set 4: Mixture of 0.56 mM of (*R*)-**PEA**, 3.94 mM of (*S*)-**PEA**, 0.69 mM of (*R*)-**PMP**, 4.81 mM of (*S*)-**PMP**, 0.56 mM of (*R*)-**PPA**, 3.94 mM of (*S*)-**PPA**, 0.69 mM of (*R*)-**PGL** and 4.81 mM of (*S*)-**PGL**

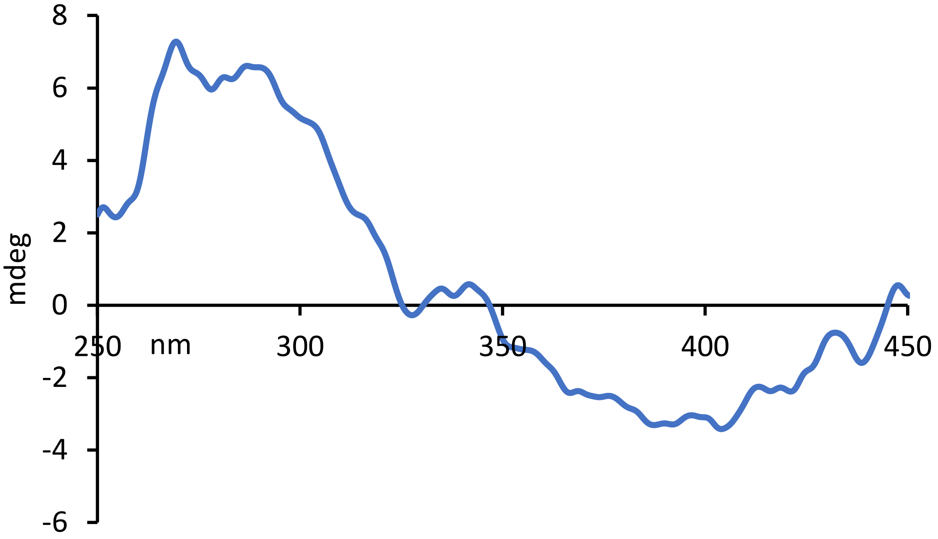

CD measurement was taken at 0.05 mM total analyte concentration in dichloroethane.

Supplementary Figure 121. CD sensing of test set 5: Mixture of 3.85 mM of (*R*)-**PEA**, 1.65 mM of (*S*)-**PEA**, 3.15 mM of (*R*)-**PMP**, 1.35 mM of (*S*)-**PMP**, 3.85 mM of (*R*)-**PPA**, 1.65 mM of (*S*)-**PPA**, 3.15 mM of (*R*)-**PGL** and 1.35 mM of (*S*)-**PGL**

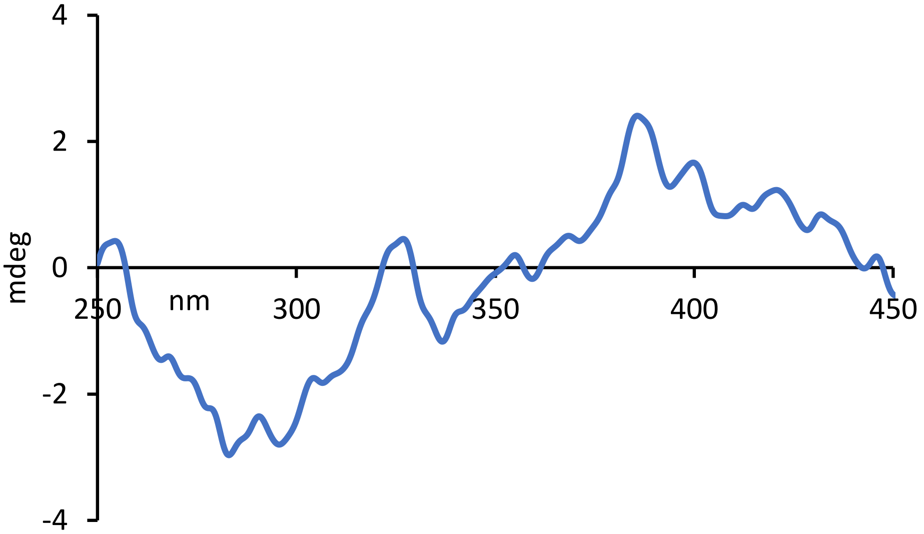

CD measurement was taken at 0.05 mM total analyte concentration in dichloroethane.

Supplementary Figure 122. CD sensing of test set 6: Mixture of 3.94 mM of (*R*)-**PEA**, 0.56 mM of (*S*)-**PEA**, 4.81 mM of (*R*)-**PMP**, 0.69 mM of (*S*)-**PMP**, 3.94 mM of (*R*)-**PPA**, 0.56 mM of (*S*)-**PPA**, 4.81 mM of (*R*)-**PGL** and 0.69 mM of (*S*)-**PGL**

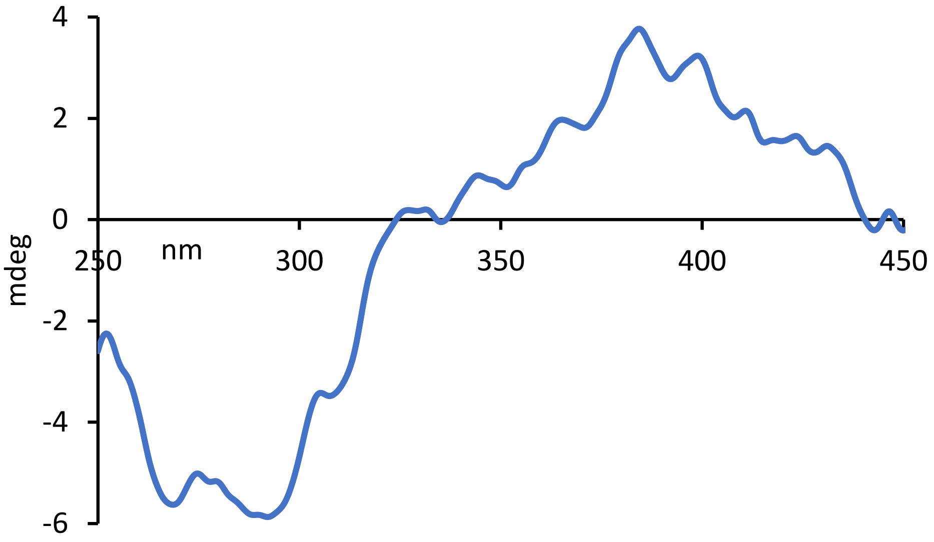

CD measurement was taken at 0.05 mM total analyte concentration in dichloroethane.

Supplementary Figure 123. CD sensing of training set 1: Mixture of 5.50 mM of (*R*)-**PEA**, 0.00 mM of (*S*)-**PEA**, 4.50 mM of (*R*)-**PMP**, 0.00 mM of (*S*)-**PMP**, 5.50 mM of (*R*)-**PPA**, 4.50 mM of (*S*)-**PPA**, 4.50 mM of (*R*)-**PGL** and 0.00 mM of (*S*)-**PGL**

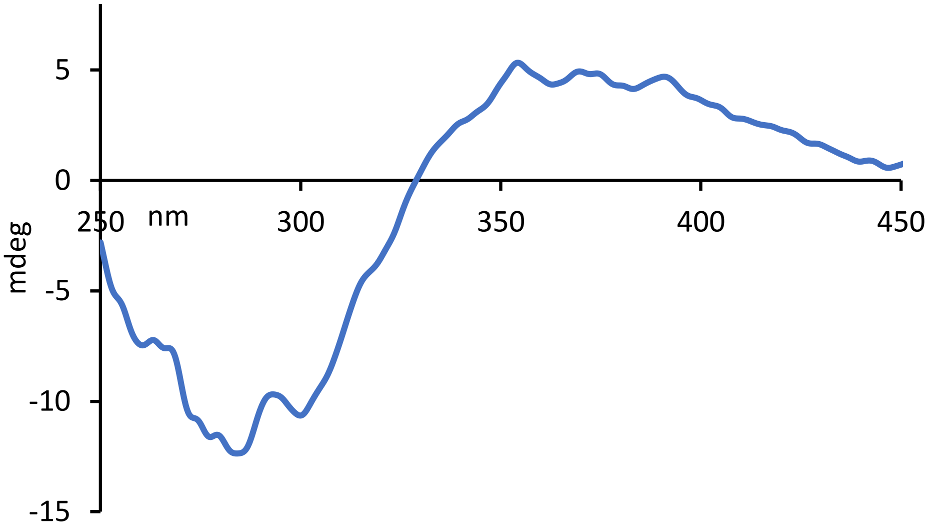

CD measurement was taken at 0.12 mM total analyte concentration in methanol.

Supplementary Figure 124. CD sensing of training set 2: Mixture of 1.88 mM of (*R*)-**PEA**, 0.63 mM of (*S*)-**PEA**, 5.63 mM of (*R*)-**PMP**, 1.88 mM of (*S*)**-PMP**, 1.88 mM of (*R*)-**PPA**, 0.63 mM of (*S*)-**PPA**, 5.63 mM of (*R*)-**PGL** and 1.88 mM of (*S*)**-PGL**

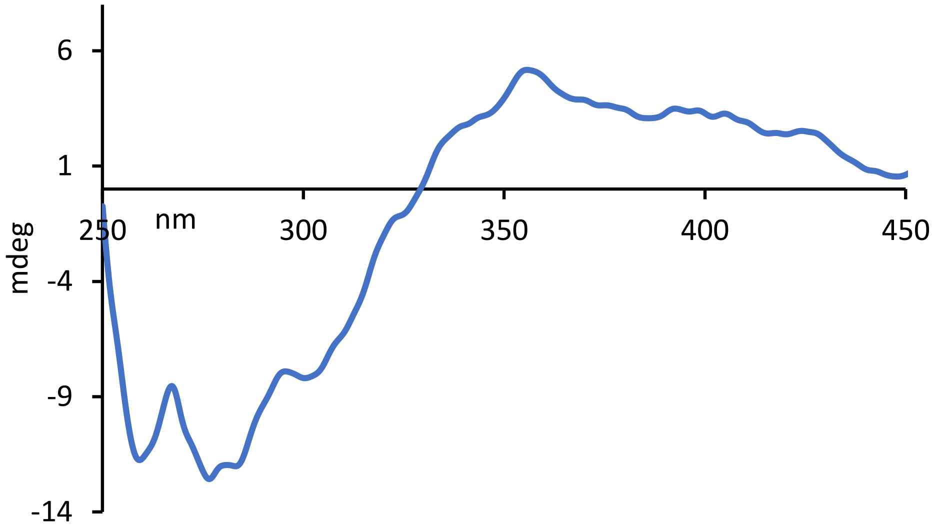

CD measurement was taken at 0.12 mM total analyte concentration in methanol.

Supplementary Figure 125. CD sensing of training set 3: Mixture of 0.70 mM of (*R*)-**PEA**, 2.80 mM of (*S*)-**PEA**, 1.30 mM of (*R*)-**PMP**, 5.20 mM of (*S*)**-PMP**, 0.70 mM of (*R*)-**PPA**, 2.80 mM of (*S*)-**PPA**, 1.30 mM of (*R*)-**PGL** and 5.20 mM of (*S*)**-PGL**

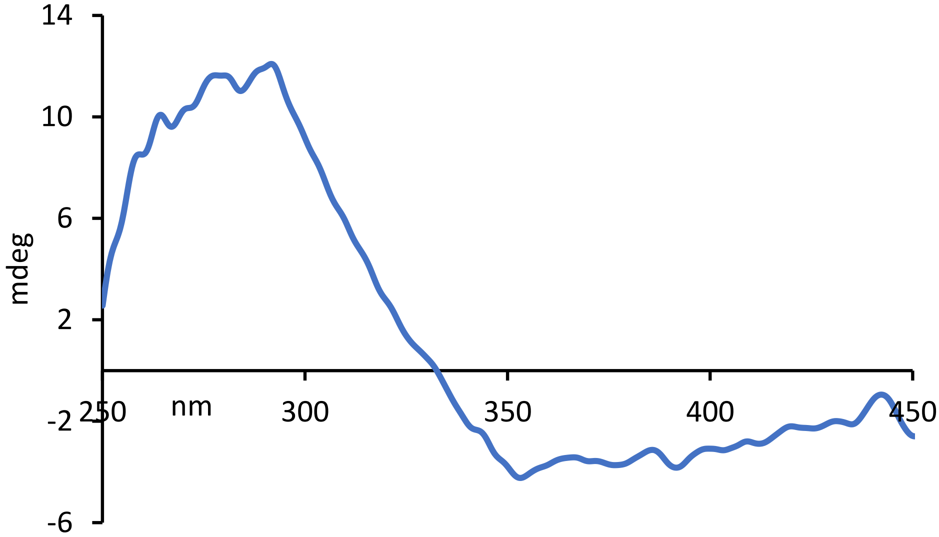

CD measurement was taken at 0.12 mM total analyte concentration in methanol.

Supplementary Figure 126. CD sensing of training set 4: Mixture of 2.80 mM of (*R*)-**PEA**, 0.70 mM of (*S*)-**PEA**, 5.20 mM of (*R*)-**PMP** and 1.30 mM of (*S*)-**PMP**, 2.80 mM of (*R*)-**PPA**, 0.70 mM of (*S*)-**PPA**, 5.20 mM of (*R*)-**PGL** and 1.30 mM of (*S*)-**PGL**

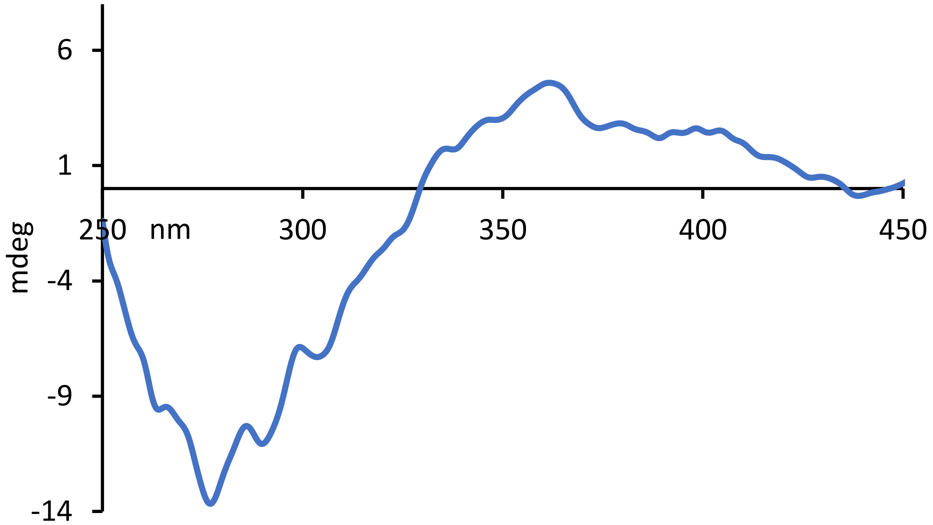

CD measurement was taken at 0.12 mM total analyte concentration in methanol.

Supplementary Figure 127. CD sensing of training set 5: Mixture of 2.19 mM of (*R*)-**PEA**, 0.31 mM of (*S*)-**PEA**, 6.56 mM of (*R*)-**PMP** and 0.94 mM of (*S*)-**PMP**, 2.19 mM of (*R*)-**PPA**, 0.31 mM of (*S*)-**PPA**, 6.56 mM of (*R*)-**PGL** and 0.94 mM of (*S*)-**PGL**

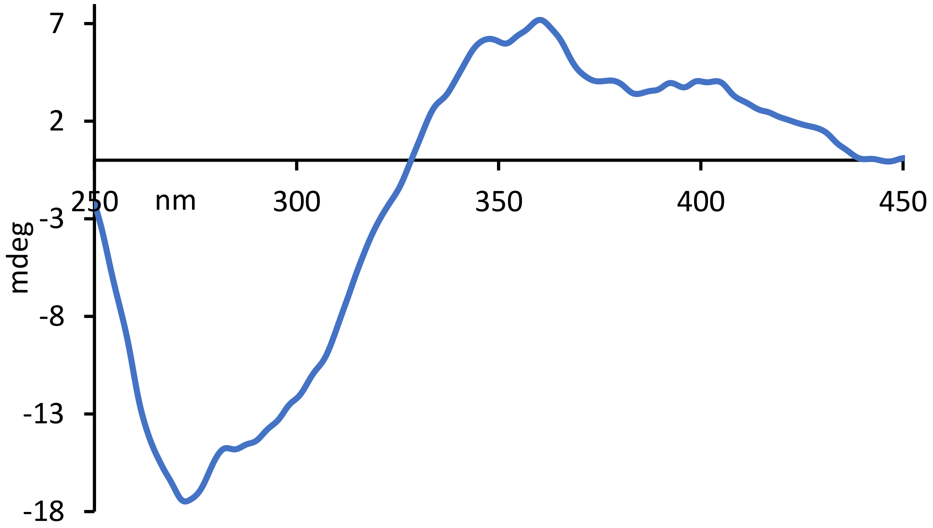

CD measurement was taken at 0.12 mM total analyte concentration in methanol.

Supplementary Figure 128. CD sensing of training set 6: Mixture of 4.40 mM of (*R*)-**PEA**, 1.10 mM of (*S*)-**PEA**, 3.60 mM of (*R*)-**PMP**, 0.90 mM of (*S*)-**PMP**, 4.40 mM of (*R*)-**PPA**, 1.10 mM of (*S*)-**PPA**, 3.60 mM of (*R*)-**PGL** and 0.90 mM of (*S*)-**PGL**

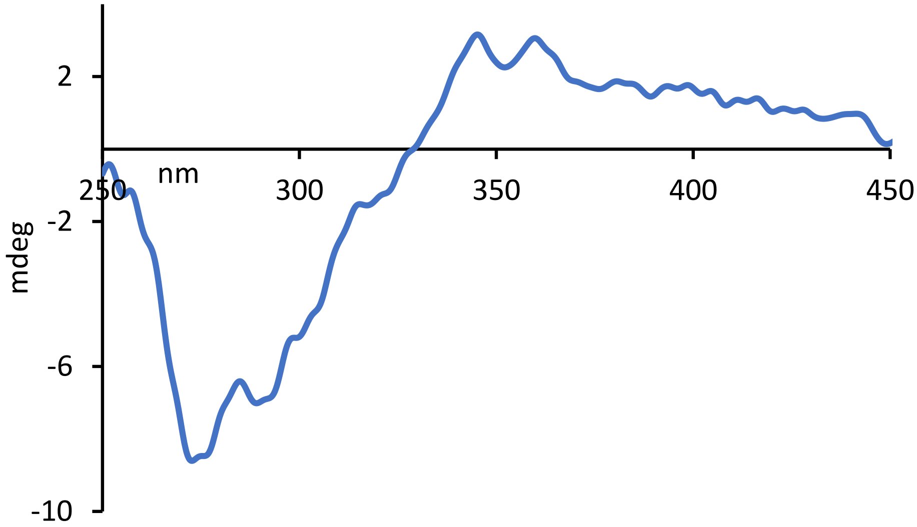

CD measurement was taken at 0.12 mM total analyte concentration in methanol.

Supplementary Figure 129. CD sensing of training set 7: Mixture of 4.88 mM of (*R*)-**PEA**, 1.63 mM of (*S*)-**PEA**, 2.63 mM of (*R*)-**PMP**, 0.88 mM of (*S*)-**PMP**, 4.88 mM of (*R*)-**PPA**, 1.63 mM of (*S*)-**PPA**, 2.63 mM of (*R*)-**PGL** and 0.88 mM of (*S*)-**PGL**

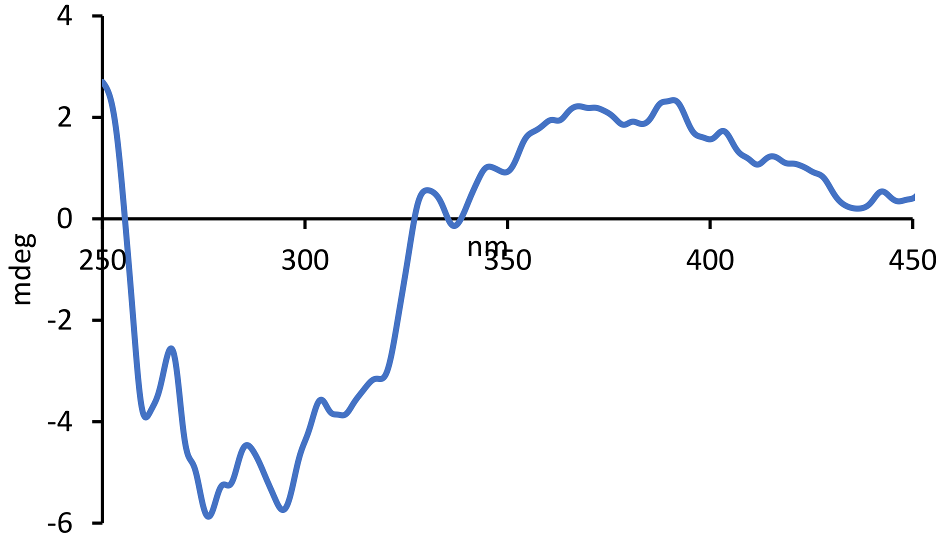

CD measurement was taken at 0.12 mM total analyte concentration in methanol.

Supplementary Figure 130. CD sensing of training set 8: Mixture of 1.75 mM of (*R*)-**PEA**, 1.75 mM of (*S*)-**PEA**, 3.25 mM of (*R*)-**PMP**, 3.25 mM of (*S*)**-PMP**, 1.75 mM of (*R*)-**PPA**, 1.75 mM of (*S*)-**PPA**, 3.25 mM of (*R*)-**PGL** and 3.25 mM of (*S*)**-PGL**

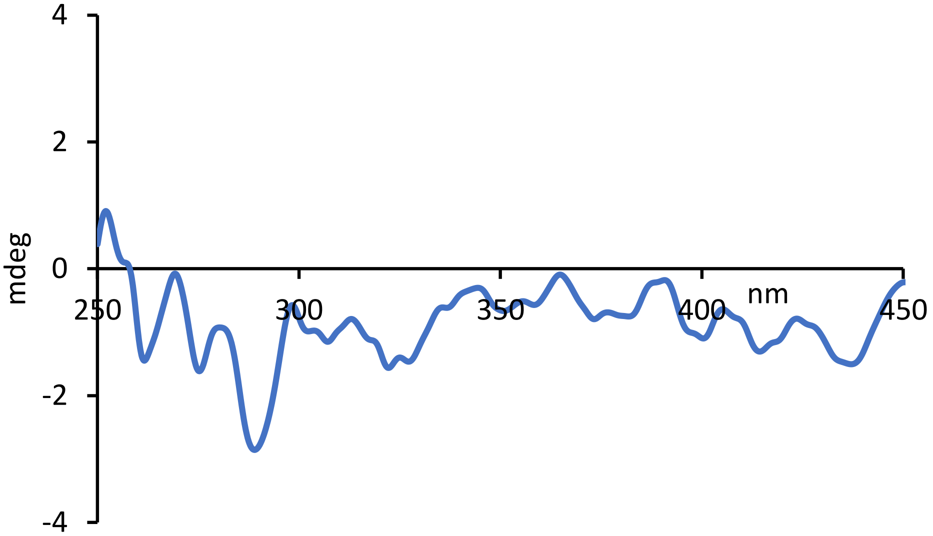

CD measurement was taken at 0.12 mM total analyte concentration in methanol.

Supplementary Figure 131. CD sensing of training set 9: Mixture of 2.45 mM of (*R*)-**PEA**, 1.05 mM of (*S*)-**PEA**, 4.55 mM of (*R*)-**PMP** and 1.95 mM of (*S*)-**PMP**, 2.45 mM of (*R*)-**PPA**, 1.05 mM of (*S*)-**PPA**, 4.55 mM of (*R*)-**PGL** and 1.95 mM of (*S*)-**PGL**

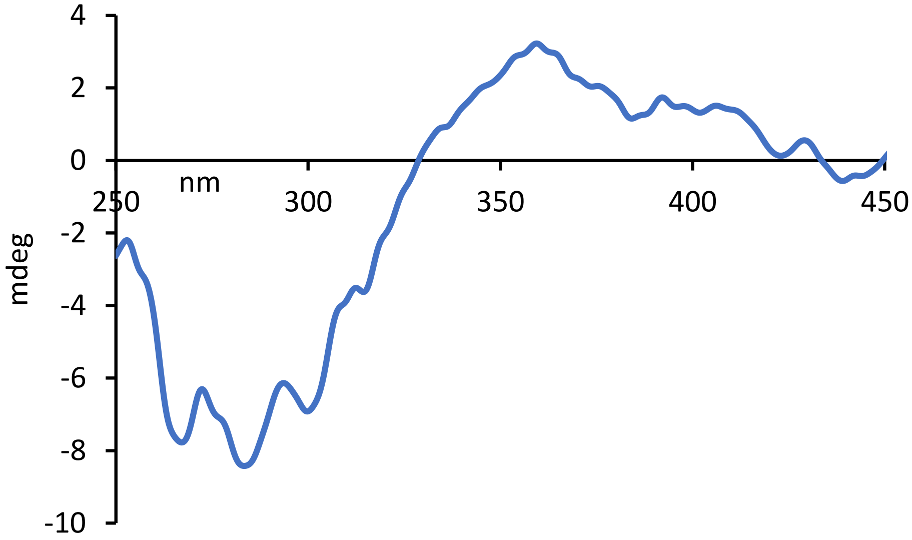

CD measurement was taken at 0.12 mM total analyte concentration in methanol.

Supplementary Figure 132. CD sensing of training set 10: Mixture of 0.75 mM of (*R*)-**PEA**, 1.75 mM of (*S*)-**PEA**, 2.25 mM of (*R*)-**PMP**, 5.25 mM of (*S*)**-PMP**, 0.75 mM of (*R*)-**PPA**, 1.75 mM of (*S*)-**PPA**, 2.25 mM of (*R*)-**PGL** and 5.25 mM of (*S*)**-PGL**

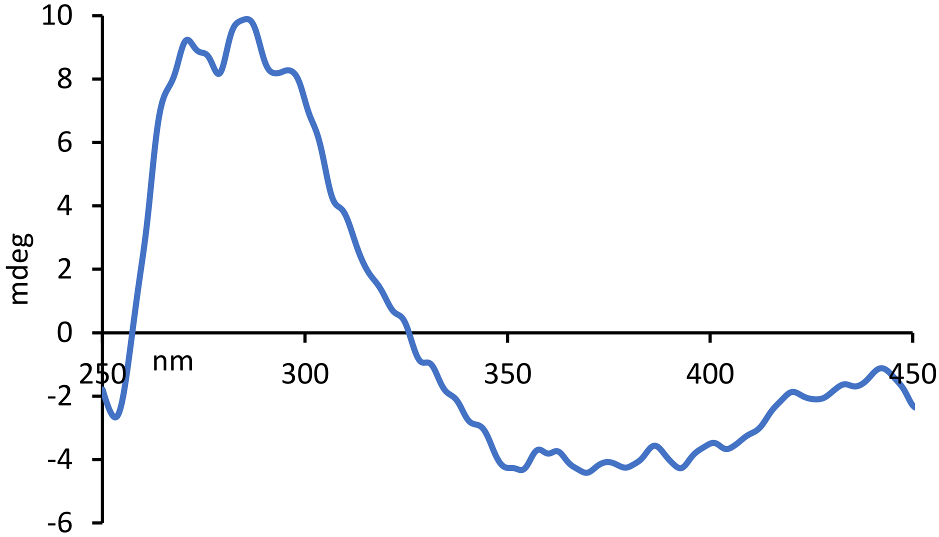

CD measurement was taken at 0.12 mM total analyte concentration in methanol.

Supplementary Figure 133. CD sensing of training set 11: Mixture of 3.75 mM of (*R*)-**PEA**, 3.75 mM of (*S*)-**PEA**, 1.25 mM of (*R*)-**PMP**, 1.25 mM of (*S*)**-PMP**, 3.75 mM of (*R*)-**PPA**, 3.75 mM of (*S*)-**PPA**, 1.25 mM of (*R*)-**PGL** and 1.25 mM of (*S*)**-PGL**

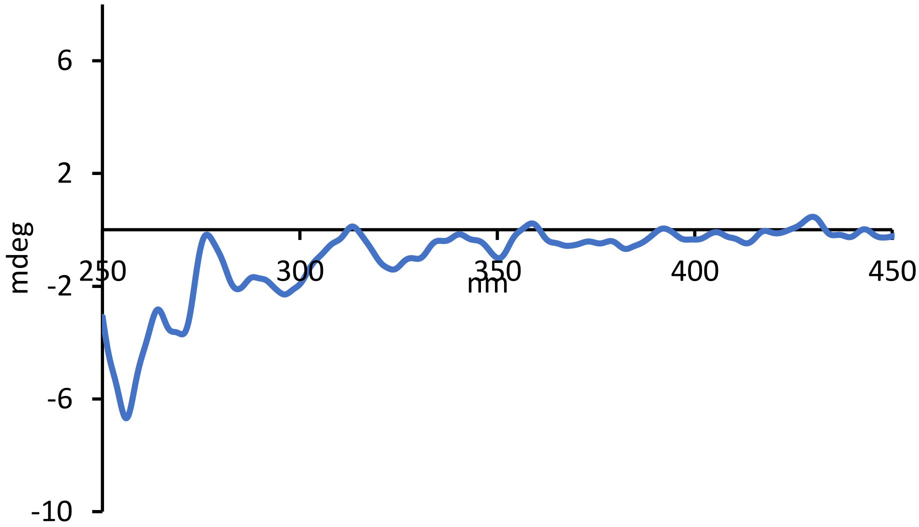

CD measurement was taken at 0.12 mM total analyte concentration in methanol.

Supplementary Figure 134. CD sensing of training set 12: Mixture of 0.63 mM of (*R*)-**PEA**, 1.88 mM of (*S*)-**PEA**, 1.75 mM of (*R*)-**PMP**, 0.75 mM of (*S*)**-PMP**, 5.25 mM of (*R*)-**PPA**, 2.25 mM of (*S*)-**PPA**, 1.75 mM of (*R*)-**PGL** and 0.75 mM of (*S*)**-PGL**

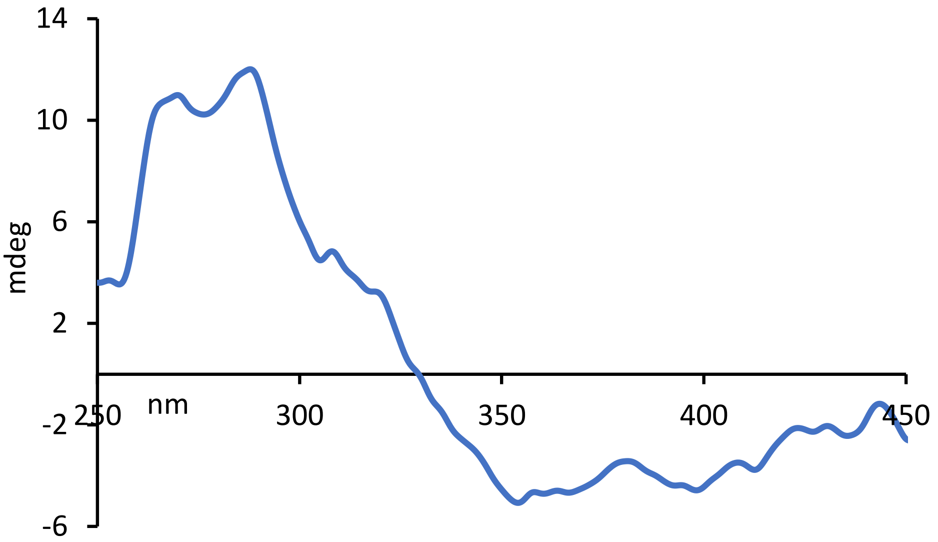

CD measurement was taken at 0.12 mM total analyte concentration in methanol.

Supplementary Figure 135. CD sensing of training set 13: Mixture of 5.25 mM of (*R*)-**PEA**, 2.25 mM of (*S*)-**PEA**, 1.75 mM of (*R*)-**PMP**, 0.75 mM of (*S*)**-PMP**, 5.25 mM of (*R*)-**PPA**, 2.25 mM of (*S*)-**PPA**, 1.75 mM of (*R*)-**PGL** and 0.75 mM of (*S*)**-PGL**

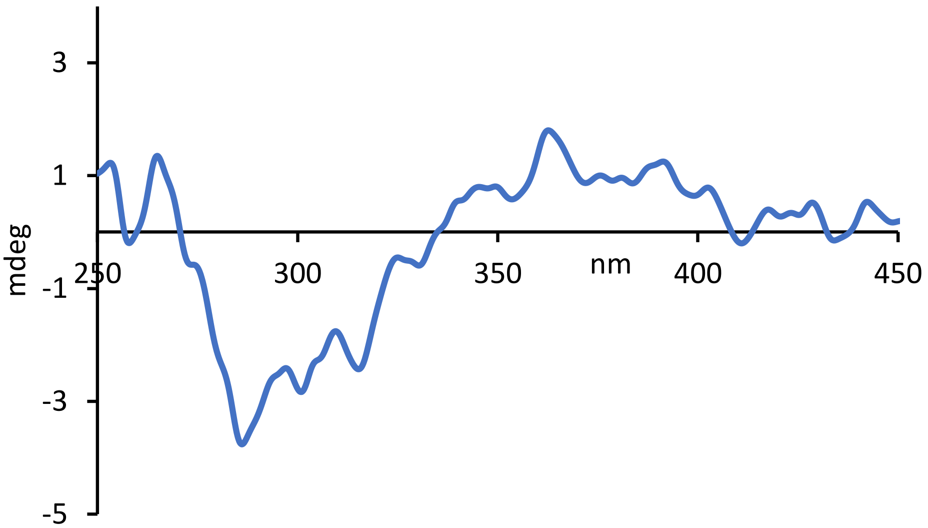

CD measurement was taken at 0.12 mM total analyte concentration in methanol.

Supplementary Figure 135. CD sensing of training set 14: Mixture of 0.00 mM of (*R*)-**PEA**, 4.50 mM of (*S*)-**PEA**, 0.00 mM of (*R*)-**PMP**, 5.50 mM of (*S*)**-PMP**, 0.00 mM of (*R*)-**PPA**, 4.50 mM of (*S*)-**PPA**, 0.00 mM of (*R*)-**PGL** and 5.50 mM of (*S*)**-PGL**

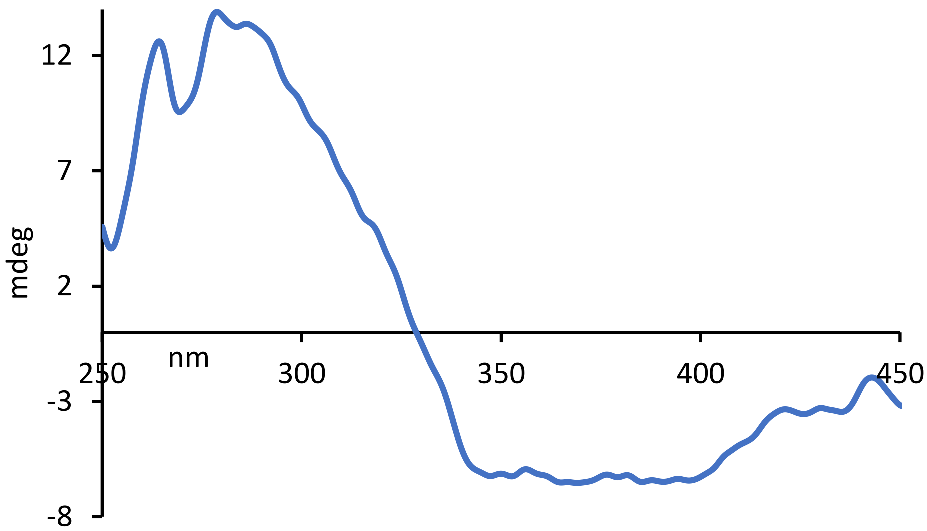

CD measurement was taken at 0.12 mM total analyte concentration in methanol.

Supplementary Figure 136. CD sensing of training set 15: Mixture of 7.50 mM of (*R*)-**PEA**, 0.00 mM of (*S*)-**PEA**, 2.50 mM of (*R*)-**PMP**, 0.00 mM of (*S*)**-PMP**, 7.50 mM of (*R*)-**PPA**, 0.00 mM of (*S*)-**PPA**, 2.50 mM of (*R*)-**PGL** and 0.00 mM of (*S*)**-PGL**

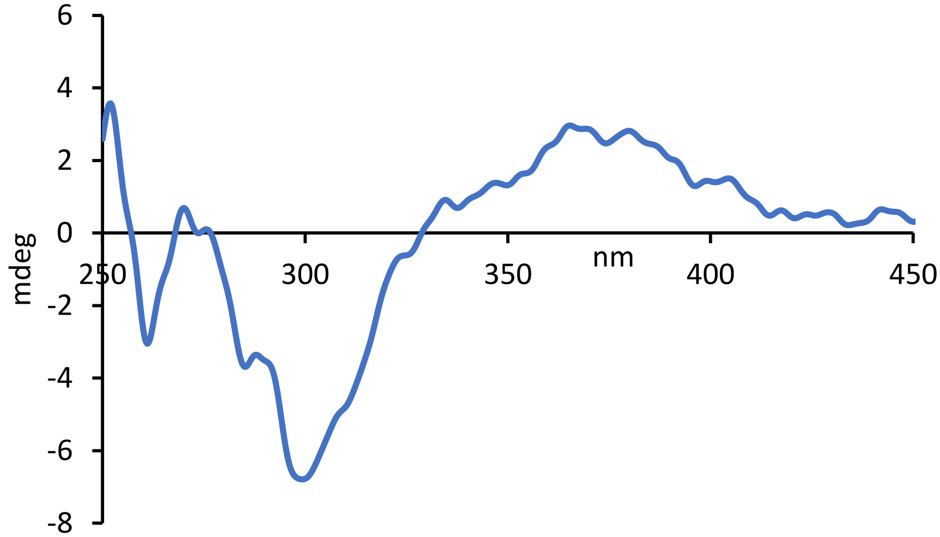

CD measurement was taken at 0.12 mM total analyte concentration in methanol.

Supplementary Figure 137. CD sensing of training set 16: Mixture of 2.81 mM of (*R*)-**PEA**, 1.69 mM of (*S*)-**PEA**, 3.44 mM of (*R*)-**PMP**, 2.06 mM of (*S*)**-PMP**, 2.81 mM of (*R*)-**PPA**, 1.69 mM of (*S*)-**PPA**, 3.44 mM of (*R*)-**PGL** and 2.06 mM of (*S*)**-PGL**

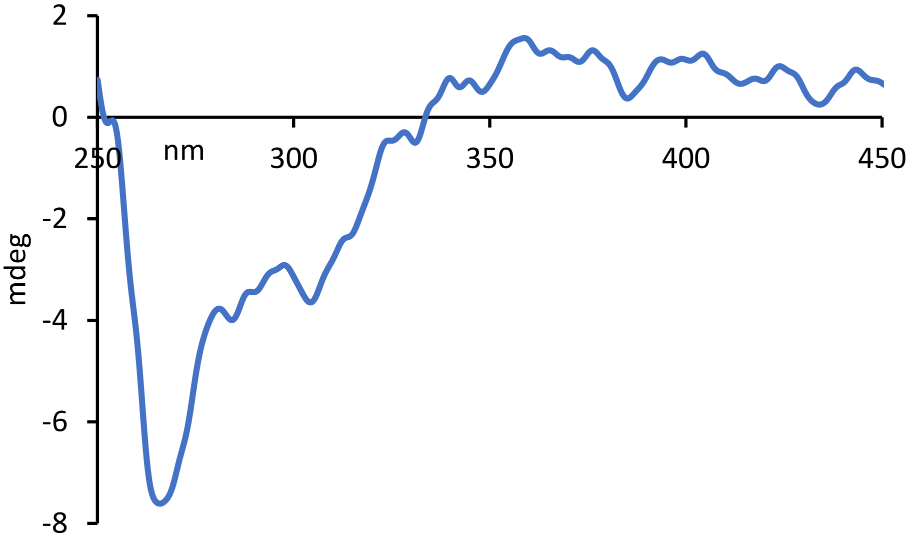

CD measurement was taken at 0.12 mM total analyte concentration in methanol.

Supplementary Figure 138. CD sensing of training set 17: Mixture of 2.75 mM of (*R*)-**PEA**, 2.75 mM of (*S*)-**PEA**, 2.25 mM of (*R*)-**PMP**, 2.25 mM of (*S*)**-PMP**, 2.75 mM of (*R*)-**PPA**, 2.75 mM of (*S*)-**PPA**, 2.25 mM of (*R*)-**PGL** and 2.25 mM of (*S*)**-PGL**

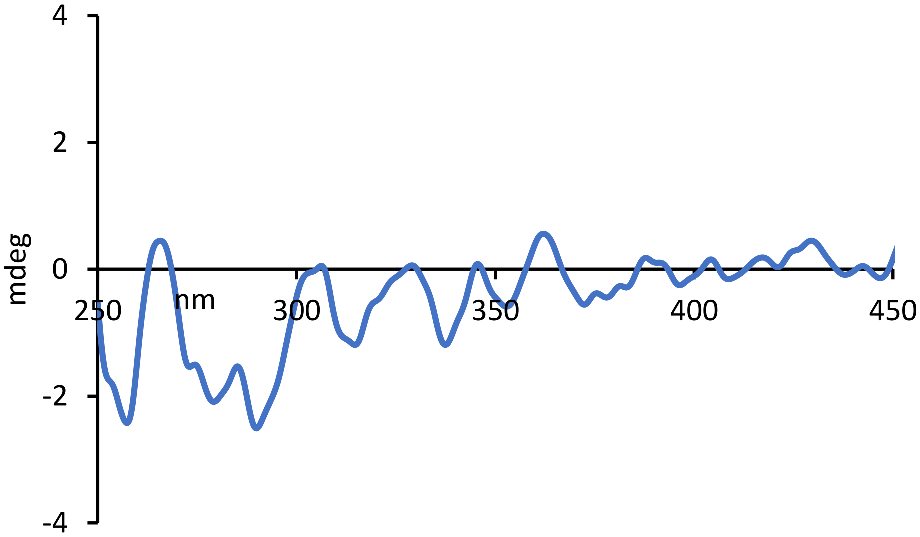

CD measurement was taken at 0.12 mM total analyte concentration in methanol.

Supplementary Figure 139. CD sensing of training set 18: Mixture of 4.06 mM of (*R*)-**PEA**, 2.44 mM of (*S*)-**PEA**, 2.19 mM of (*R*)-**PMP**, 1.31 mM of (*S*)**-PMP**, 4.06 mM of (*R*)-**PPA**, 2.44 mM of (*S*)-**PPA**, 2.19 mM of (*R*)-**PGL** and 1.31 mM of (*S*)**-PGL**

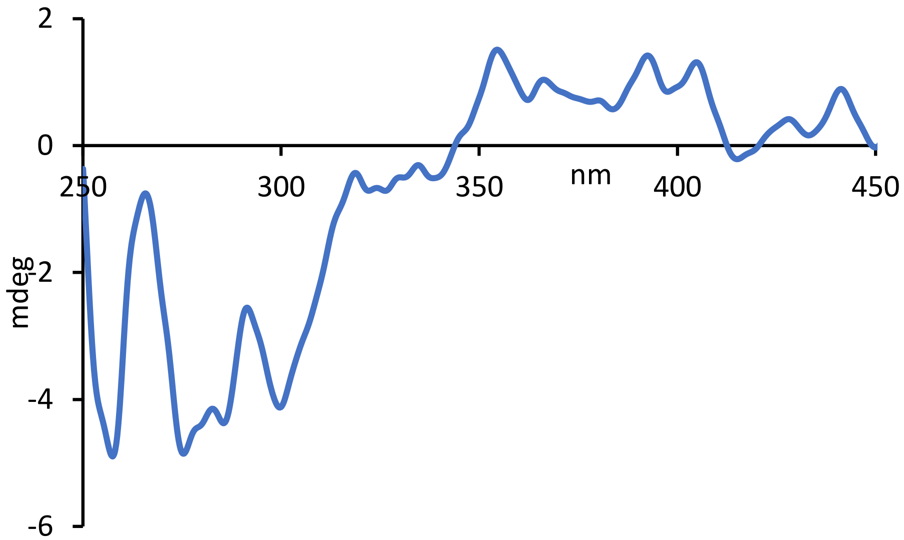

CD measurement was taken at 0.12 mM total analyte concentration in methanol.

Supplementary Figure 140. CD sensing of training set 19: Mixture of 6.00 mM of (*R*)-**PEA**, 1.50 mM of (*S*)-**PEA**, 2.00 mM of (*R*)-**PMP**, 0.50 mM of (*S*)**-PMP**, 6.00 mM of (*R*)-**PPA**, 1.50 mM of (*S*)-**PPA**, 2.00 mM of (*R*)-**PGL** and 0.50 mM of (*S*)**-PGL**

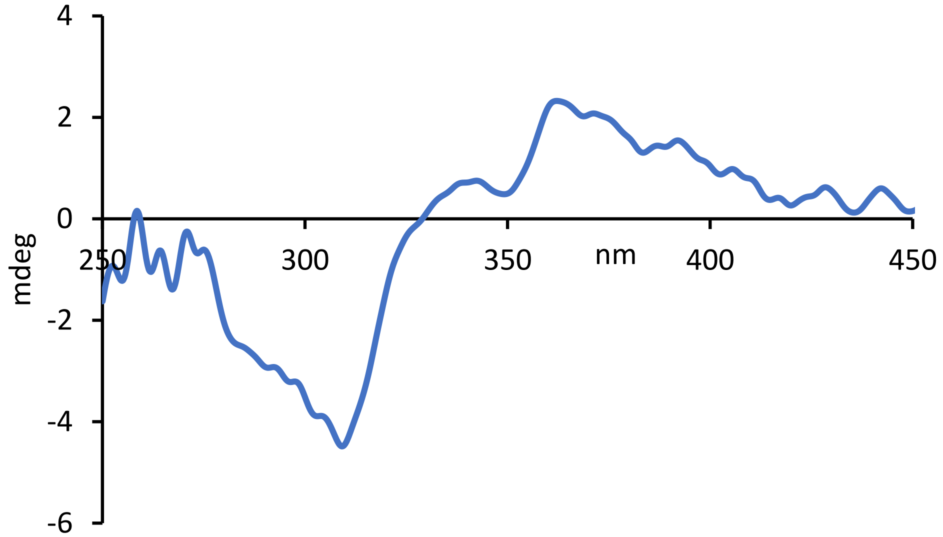

CD measurement was taken at 0.12 mM total analyte concentration in methanol.

Supplementary Figure 141. CD sensing of training set 20: Mixture of 3.50 mM of (*R*)-**PEA**, 0.00 mM of (*S*)-**PEA**, 6.50 mM of (*R*)-**PMP**, 0.00 mM of (*S*)**-PMP**, 3.50 mM of (*R*)-**PPA**, 0.00 mM of (*S*)-**PPA**, 6.50 mM of (*R*)-**PGL** and 0.00 mM of (*S*)**-PGL**

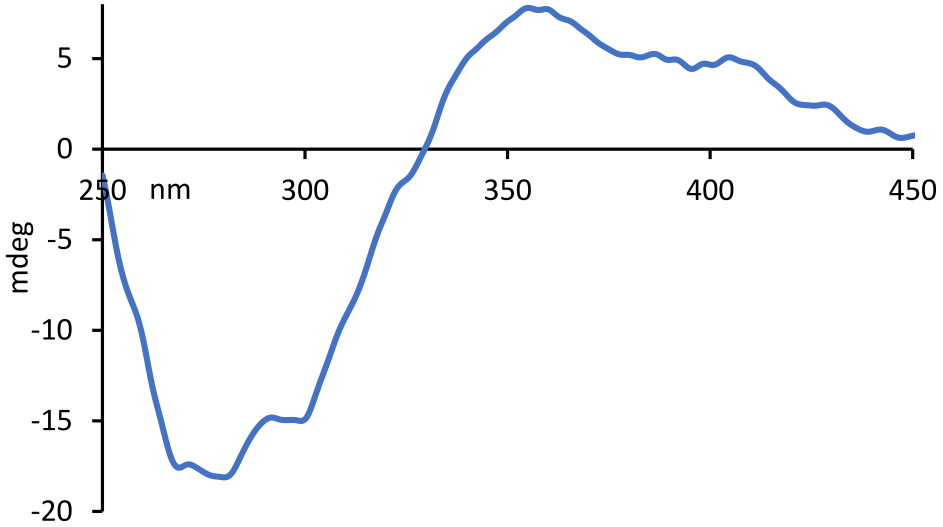

CD measurement was taken at 0.12 mM total analyte concentration in methanol.

Supplementary Figure 142. CD sensing of test set 1: Mixture of 3.38 mM of (*R*)-**PEA**, 1.13 mM of (*S*)-**PEA**, 4.13 mM of (*R*)-**PMP**, 1.38 mM of (*S*)**-PMP**, 3.38 mM of (*R*)-**PPA**, 1.13 mM of (*S*)-**PPA**, 4.13 mM of (*R*)-**PGL** and 1.38 mM of (*S*)**-PGL**

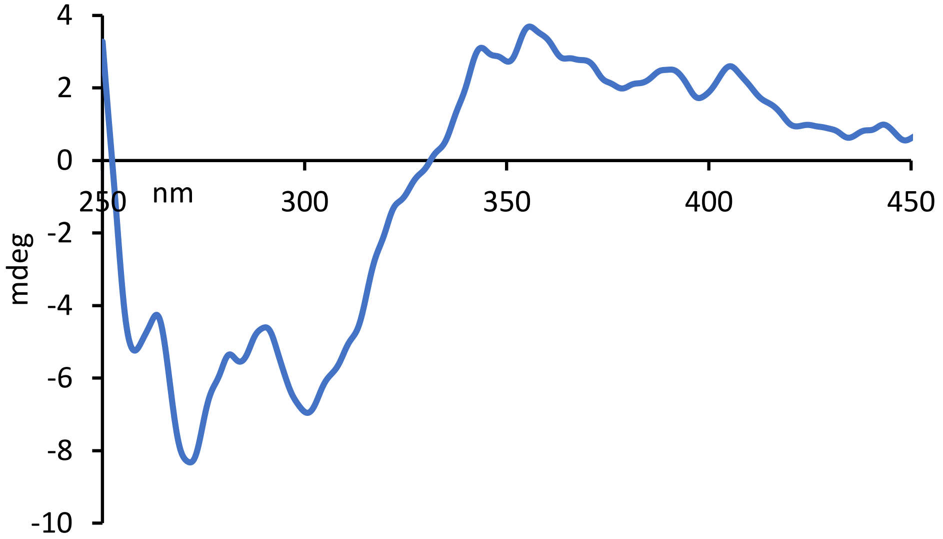

CD measurement was taken at 0.12 mM total analyte concentration in methanol.

Supplementary Figure 143. CD sensing of test set 2: Mixture of 5.69 mM of (*R*)-**PEA**, 0.81 mM of (*S*)-**PEA**, 3.06 mM of (*R*)-**PMP**, 0.44 mM of (*S*)-**PMP**, 5.69 mM of (*R*)-**PPA**, 0.81 mM of (*S*)-**PPA**, 3.06 mM of (*R*)-**PGL** and 0.44 mM of (*S*)-**PGL**

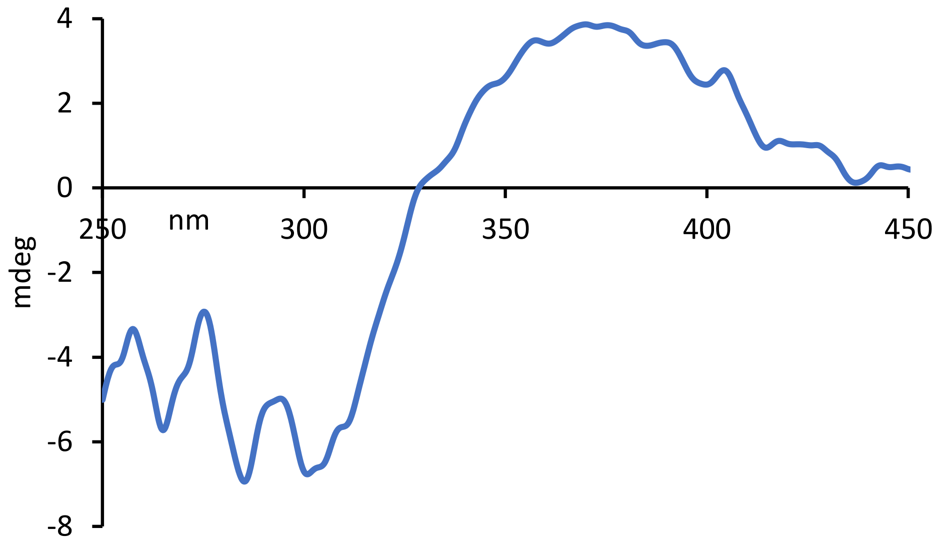

CD measurement was taken at 0.12 mM total analyte concentration in methanol.

Supplementary Figure 144. CD sensing of test set 3: Mixture of 0.94 mM of (*R*)-**PEA**, 1.56 mM of (*S*)-**PEA**, 2.81 mM of (*R*)-**PMP**, 4.69 mM of (*S*)-**PMP**, 0.94 mM of (*R*)-**PPA**, 1.56 mM of (*S*)-**PPA**, 2.81 mM of (*R*)-**PGL** and 4.69 mM of (*S*)-**PGL**

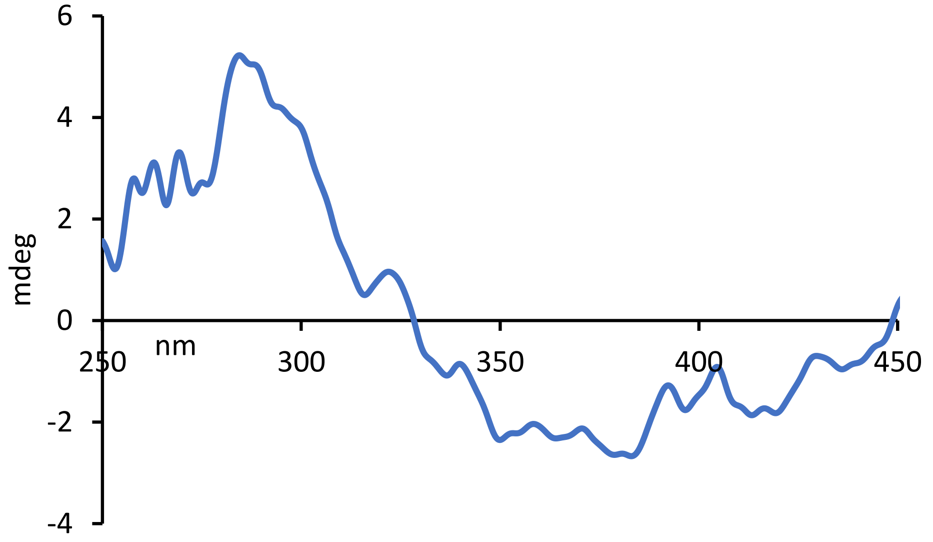

CD measurement was taken at 0.12 mM total analyte concentration in methanol.

Supplementary Figure 145. CD sensing of test set 4: Mixture of 0.56 mM of (*R*)-**PEA**, 3.94 mM of (*S*)-**PEA**, 0.69 mM of (*R*)-**PMP**, 4.81 mM of (*S*)-**PMP**, 0.56 mM of (*R*)-**PPA**, 3.94 mM of (*S*)-**PPA**, 0.69 mM of (*R*)-**PGL** and 4.81 mM of (*S*)-**PGL**

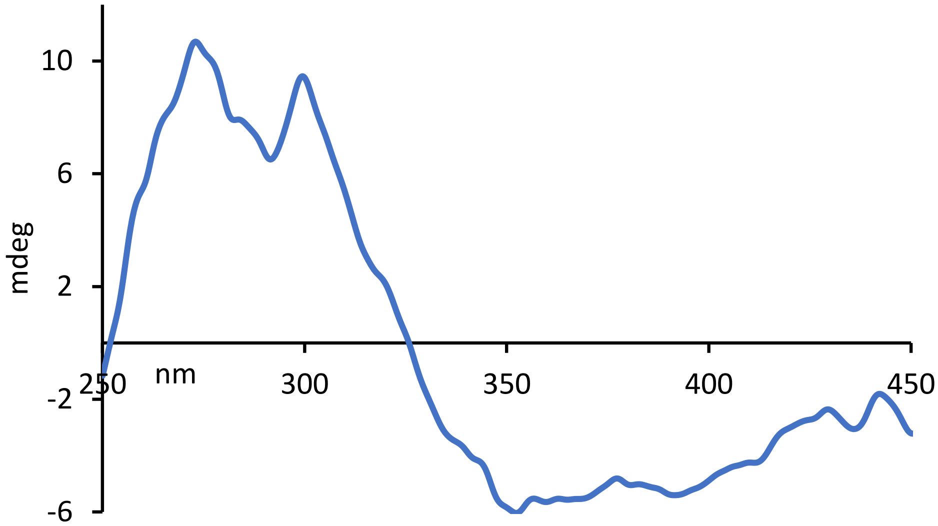

CD measurement was taken at 0.12 mM total analyte concentration in methanol.

Supplementary Figure 146. CD sensing of test set 5: Mixture of 3.85 mM of (*R*)-**PEA**, 1.65 mM of (*S*)-**PEA**, 3.15 mM of (*R*)-**PMP**, 1.35 mM of (*S*)-**PMP**, 3.85 mM of (*R*)-**PPA**, 1.65 mM of (*S*)-**PPA**, 3.15 mM of (*R*)-**PGL** and 1.35 mM of (*S*)-**PGL**

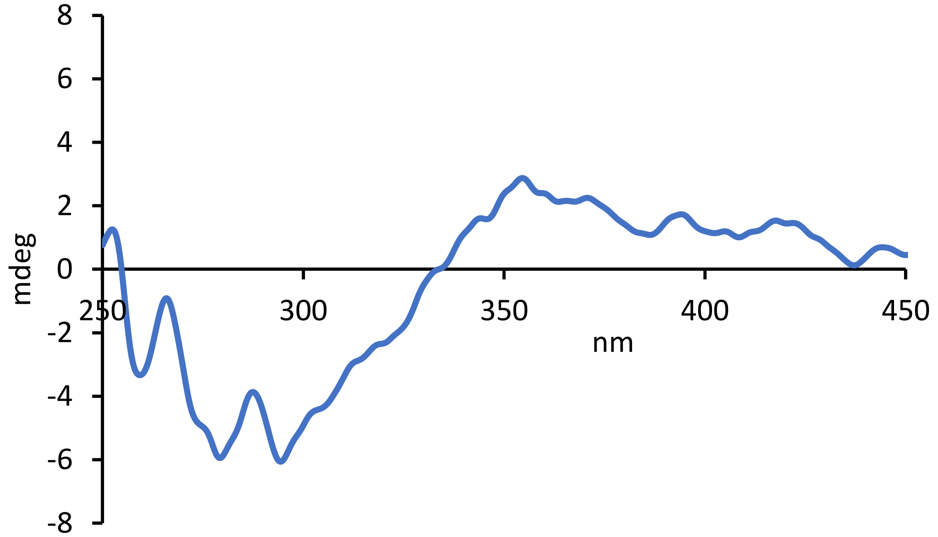

CD measurement was taken at 0.12 mM total analyte concentration in methanol.

Supplementary Figure 147. CD sensing of test set 6: Mixture of 3.94 mM of (*R*)-**PEA**, 0.56 mM of (*S*)-**PEA**, 4.81 mM of (*R*)-**PMP**, 0.69 mM of (*S*)**-PMP**, 3.94 mM of (*R*)-**PPA**, 0.56 mM of (*S*)-**PPA**, 4.81 mM of (*R*)-**PGL** and 0.69 mM of (*S*)**-PGL**

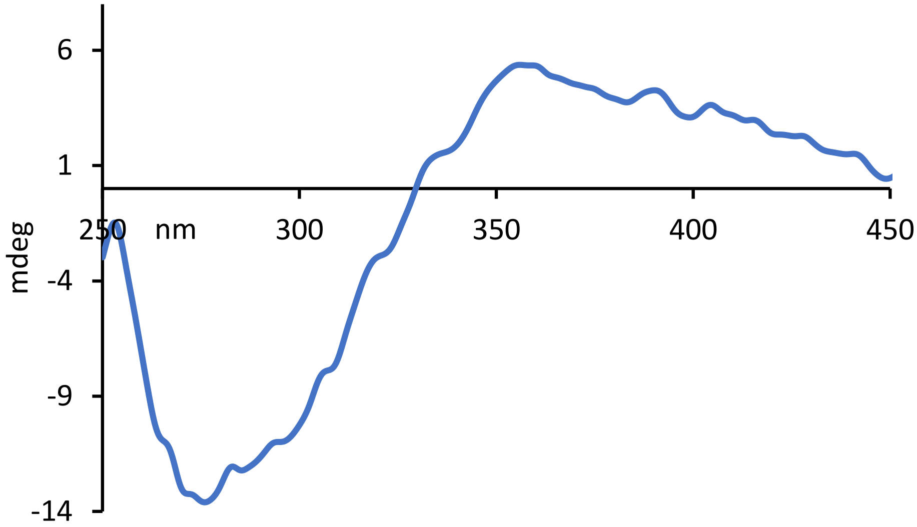

CD measurement was taken at 0.12 mM total analyte concentration in methanol.

Supplementary Figure 148. UV sensing of training set 1: Mixture of 5.50 mM of (*R*)-**PEA**, 0.00 mM of (*S*)-**PEA**, 4.50 mM of (*R*)-**PMP**, 0.00 mM of (*S*)**-PMP**, 5.50 mM of (*R*)-**PPA**, 4.50 mM of (*S*)-**PPA**, 4.50 mM of (*R*)-**PGL** and 0.00 mM of (*S*)**-PGL**

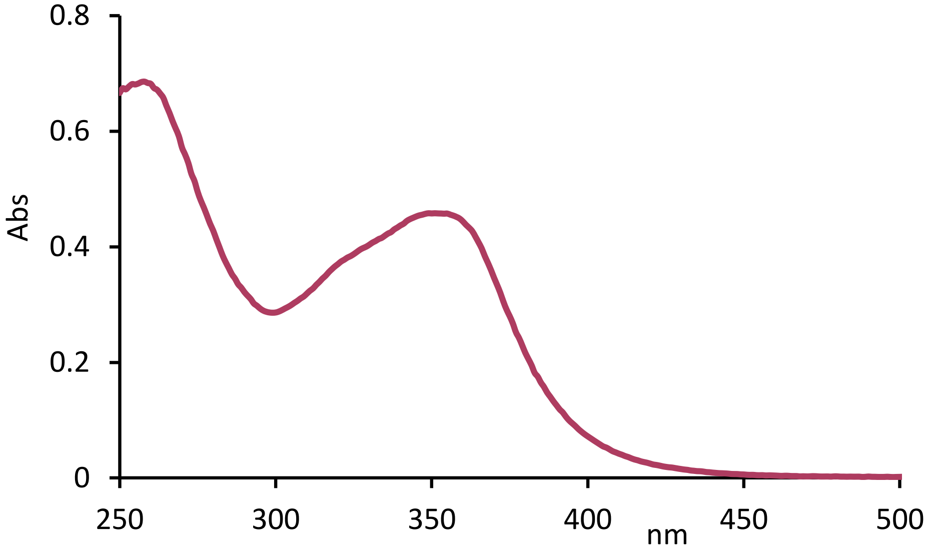

UV measurement was taken at 0.03 mM total analyte concentration in dichloroethane.

Supplementary Figure 149. UV sensing of training set 2: Mixture of 1.88 mM of (*R*)-**PEA**, 0.63 mM of (*S*)-**PEA**, 5.63 mM of (*R*)-**PMP**, 1.88 mM of (*S*)**-PMP**, 1.88 mM of (*R*)-**PPA**, 0.63 mM of (*S*)-**PPA**, 5.63 mM of (*R*)-**PGL** and 1.88 mM of (*S*)**-PGL**

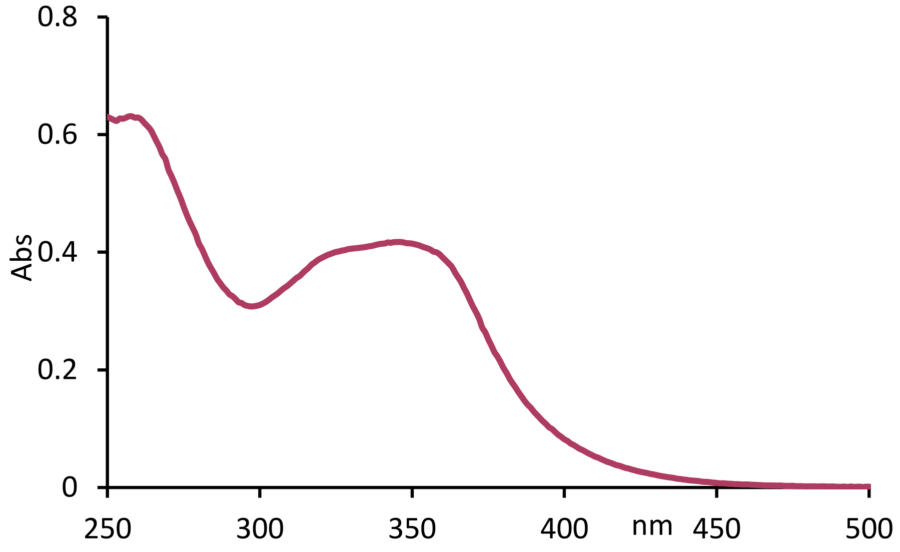

UV measurement was taken at 0.03 mM total analyte concentration in dichloroethane.

Supplementary Figure 150. UV sensing of training set 3: Mixture of 0.70 mM of (*R*)-**PEA**, 2.80 mM of (*S*)-**PEA**, 1.30 mM of (*R*)-**PMP**, 5.20 mM of (*S*)**-PMP**, 0.70 mM of (*R*)-**PPA**, 2.80 mM of (*S*)-**PPA**, 1.30 mM of (*R*)-**PGL** and 5.20 mM of (*S*)**-PGL**

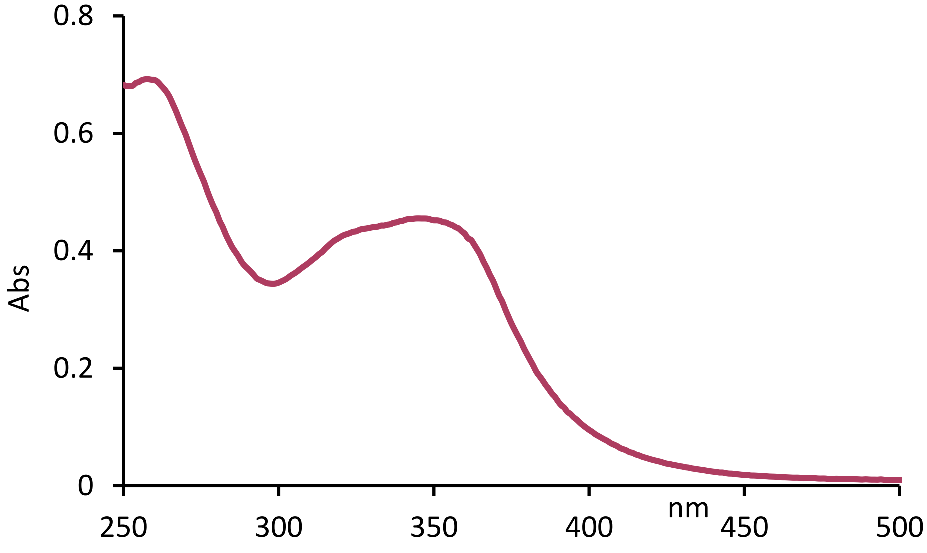

UV measurement was taken at 0.03 mM total analyte concentration in dichloroethane.

Supplementary Figure 151. UV sensing of training set 4: Mixture of 2.80 mM of (*R*)-**PEA**, 0.70 mM of (*S*)-**PEA**, 5.20 mM of (*R*)-**PMP**,1.30 mM of (*S*)-**PMP**, 2.80 mM of (*R*)-**PPA**, 0.70 mM of (*S*)-**PPA**, 5.20 mM of (*R*)-**PGL** and 1.30 mM of (*S*)-**PGL**

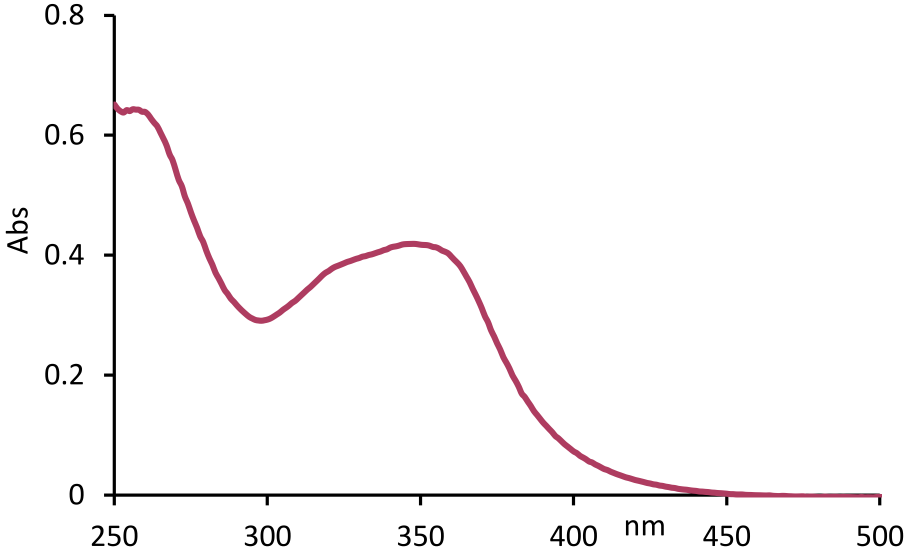

UV measurement was taken at 0.03 mM total analyte concentration in dichloroethane.

Supplementary Figure 152. UV sensing of training set 5: Mixture of 2.19 mM of (*R*)-**PEA**, 0.31 mM of (*S*)-**PEA**, 6.56 mM of (*R*)-**PMP**, 0.94 mM of (*S*)-**PMP**, 2.19 mM of (*R*)-**PPA**, 0.31 mM of (*S*)-**PPA**, 6.56 mM of (*R*)-**PGL** and 0.94 mM of (*S*)-**PGL**

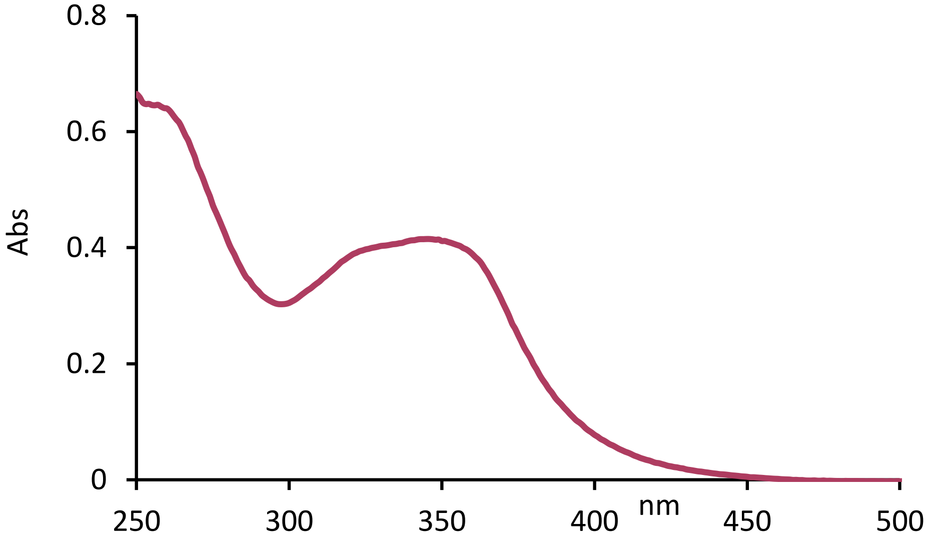

UV measurement was taken at 0.03 mM total analyte concentration in dichloroethane.

Supplementary Figure 153. UV sensing of training set 6: Mixture of 4.40 mM of (*R*)-**PEA**, 1.10 mM of (*S*)-**PEA**, 3,60 mM of (*R*)-**PMP**, 0.90 mM of (*S*)**-PMP**, 4.40 mM of (*R*)-**PPA**, 1.10 mM of (*S*)-**PPA**, 3.60 mM of (*R*)-**PGL** and 0.90 mM of (*S*)**-PGL**

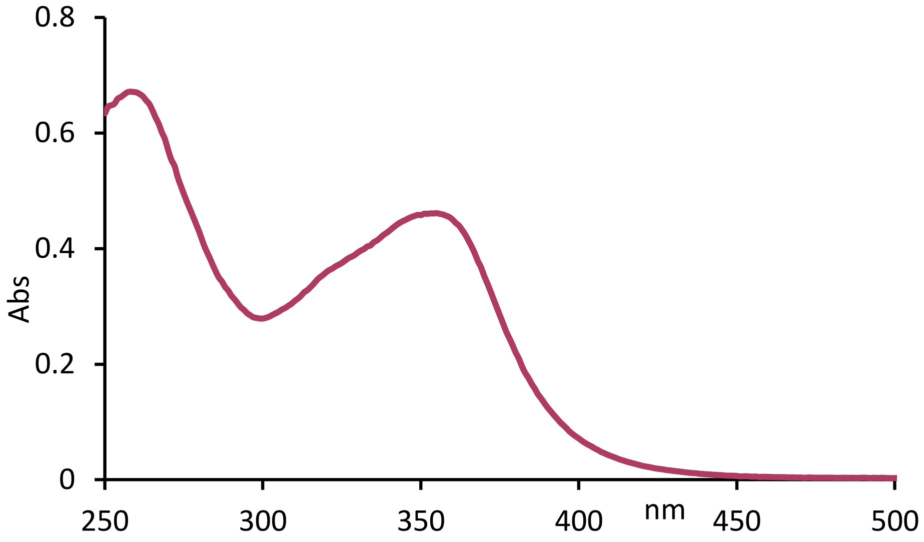

UV measurement was taken at 0.03 mM total analyte concentration in dichloroethane.

Supplementary Figure 154. UV sensing of training set 7: Mixture of 4.88 mM of (*R*)-**PEA**, 1.63 mM of (*S*)-**PEA**, 2.63 mM of (*R*)-**PMP**, 0.88 mM of (*S*)**-PMP**, 4.88 mM of (*R*)-**PPA**, 1.63 mM of (*S*)-**PPA**, 2.63 mM of (*R*)-**PGL** and 0.88 mM of (*S*)**-PGL**

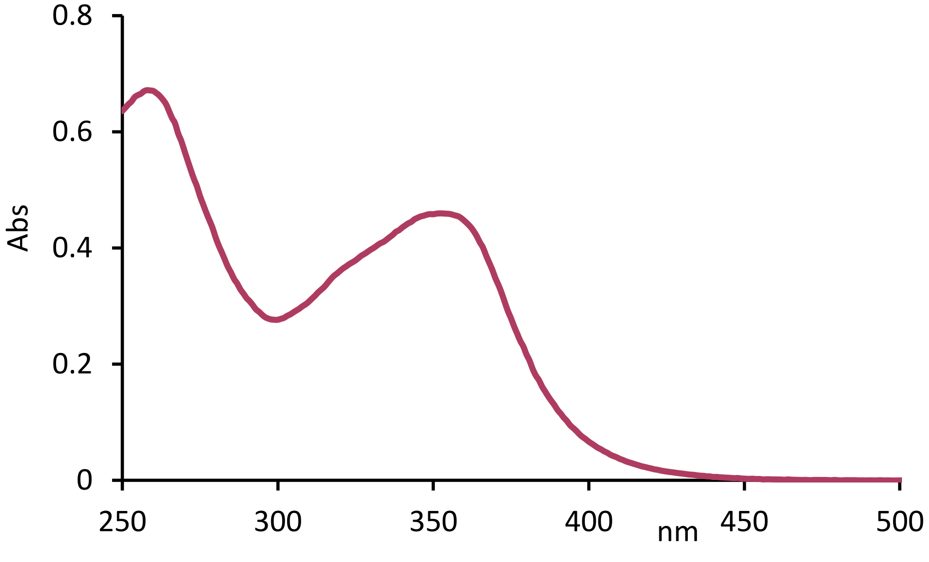

UV measurement was taken at 0.03 mM total analyte concentration in dichloroethane.

Supplementary Figure 155. UV sensing of training set 8: Mixture of 1.75 mM of (*R*)-**PEA**, 1.75 mM of (*S*)-**PEA**, 3.25 mM of (*R*)-**PMP**, 3.25 mM of (*S*)**-PMP**, 1.75 mM of (*R*)-**PPA**, 1.75 mM of (*S*)-**PPA**, 3.25 mM of (*R*)-**PGL** and 3.25 mM of (*S*)**-PGL**

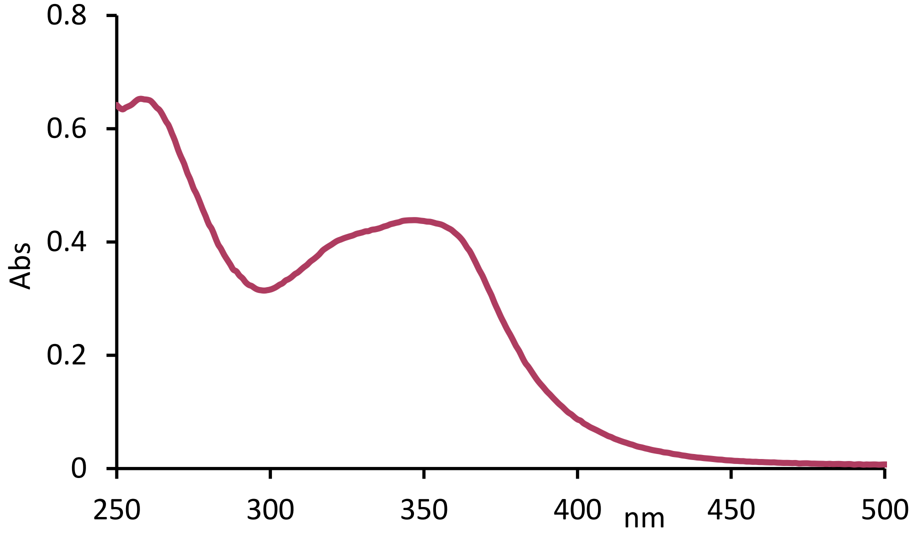

UV measurement was taken at 0.03 mM total analyte concentration in dichloroethane.

Supplementary Figure 156. UV sensing of training set 9: Mixture of 2.45 mM of (*R*)-**PEA**, 1.05 mM of (*S*)-**PEA**, 4.55 mM of (*R*)-**PMP** and 1.95 mM of (*S*)**-PMP**, 2.45 mM of (*R*)-**PPA**, 1.05 mM of (*S*)-**PPA**, 4.55 mM of (*R*)-**PGL** and 1.95 mM of (*S*)**-PGL**

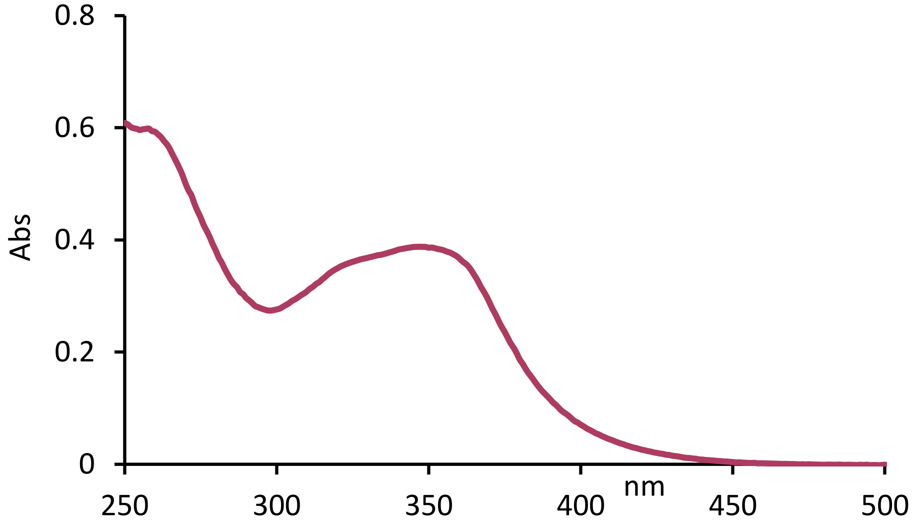

UV measurement was taken at 0.03 mM total analyte concentration in dichloroethane.

Supplementary Figure 157. UV sensing of training set 10: Mixture of 0.75 mM of (*R*)-**PEA**, 1.75 mM of (*S*)-**PEA**, 2.25 mM of (*R*)-**PMP**, 5.25 mM of (*S*)**-PMP**, 0.75 mM of (*R*)-**PPA**, 1.75 mM of (*S*)-**PPA**, 2.25 mM of (*R*)-**PGL** and 5.25 mM of (*S*)**-PGL**

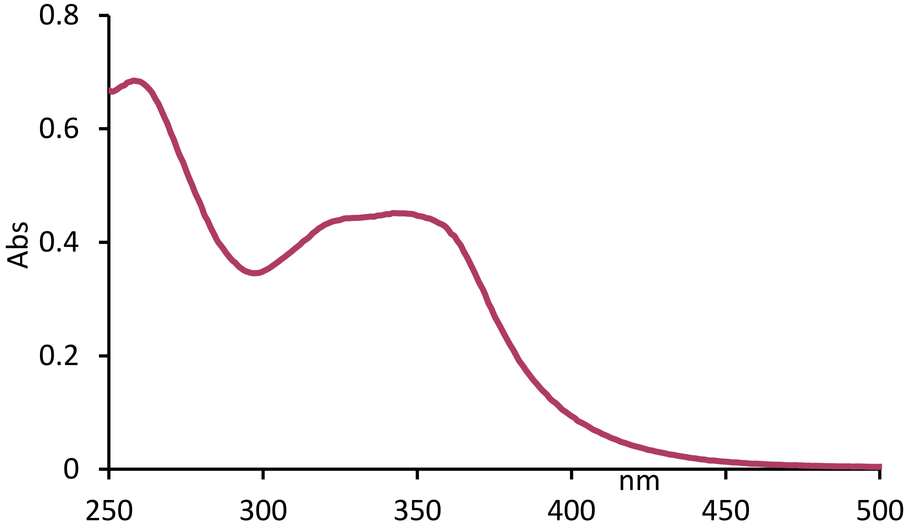

UV measurement was taken at 0.03 mM total analyte concentration in dichloroethane.

Supplementary Figure 158. UV sensing of training set 11: Mixture of 3.75 mM of (*R*)-**PEA**, 3.75 mM of (*S*)-**PEA**, 1.25 mM of (*R*)-**PMP**, 1.25 mM of (*S*)**-PMP**, 3.75 mM of (*R*)-**PPA**, 3.75 mM of (*S*)-**PPA**, 1.25 mM of (*R*)-**PGL** and 1.25 mM of (*S*)**-PGL**

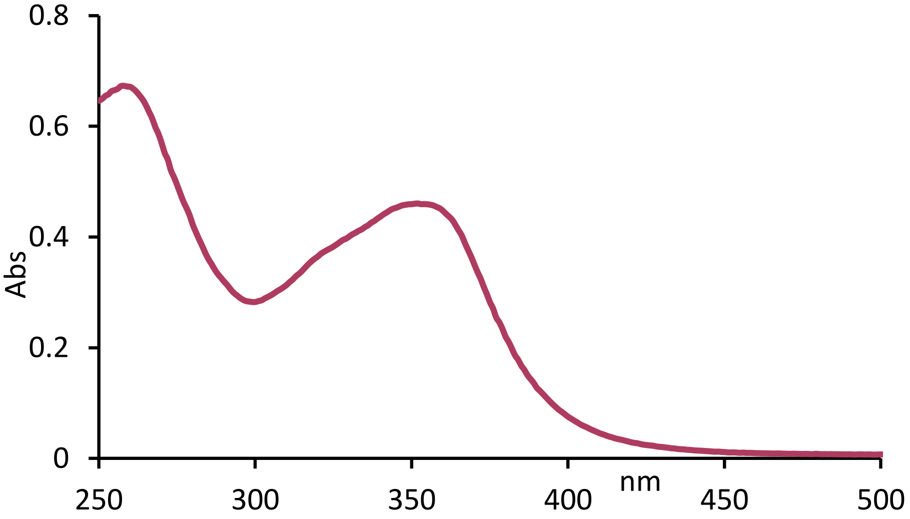

UV measurement was taken at 0.03 mM total analyte concentration in dichloroethane.

Supplementary Figure 159. UV sensing of training set 12: Mixture of 0.63 mM of (*R*)-**PEA**, 1.88 mM of (*S*)-**PEA**, 1.75 mM of (*R*)-**PMP**, 0.75 mM of (*S*)-**PMP**, 5.25 mM of (*R*)-**PPA**, 2.25 mM of (*S*)-**PPA**, 1.75 mM of (*R*)-**PGL** and 0.75 mM of (*S*)-**PGL**

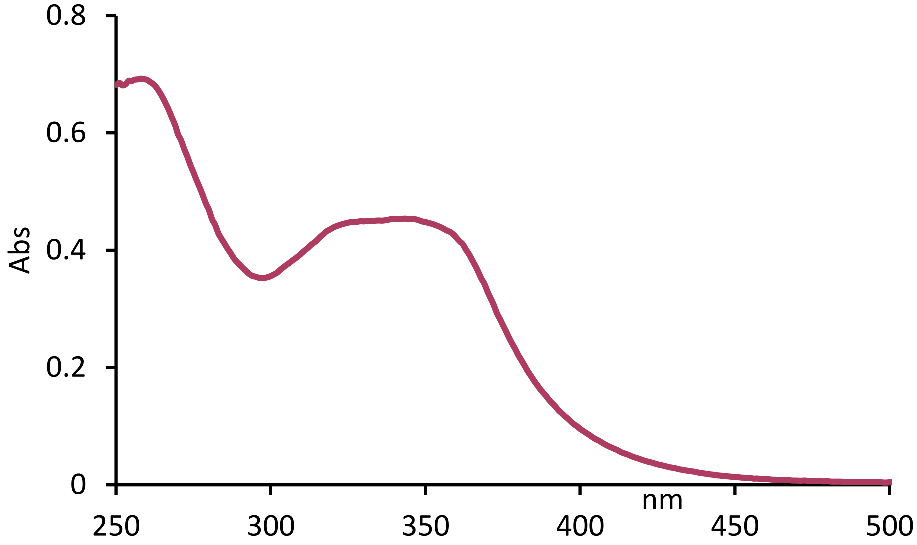

UV measurement was taken at 0.03 mM total analyte concentration in dichloroethane.

Supplementary Figure 160. UV sensing of training set 13: Mixture of 5.25 mM of (*R*)-**PEA**, 2.25 mM of (*S*)-**PEA**, 1.75 mM of (*R*)-**PMP**, 0.75 mM of (*S*)-**PMP**, 5.25 mM of (*R*)-**PPA**, 2.25 mM of (*S*)-**PPA**, 1.75 mM of (*R*)-**PGL** and 0.75 mM of (*S*)-**PGL**

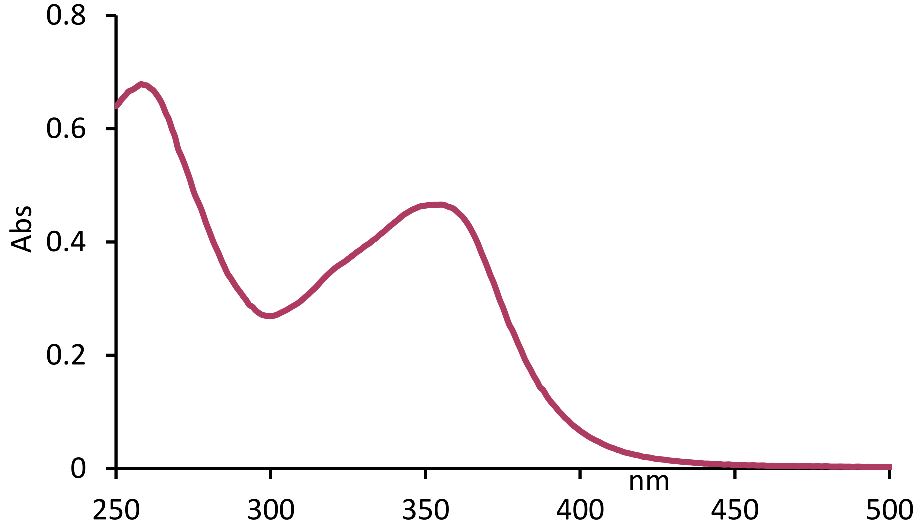

UV measurement was taken at 0.03 mM total analyte concentration in dichloroethane.

Supplementary Figure 161. UV sensing of training set 14: Mixture of 0.00 mM of (*R*)-**PEA**, 4.50 mM of (*S*)-**PEA**, 0.00 mM of (*R*)-**PMP**, 5.50 mM of (*S*)-**PMP**, 0.00 mM of (*R*)-**PPA**, 4.50 mM of (*S*)-**PPA**, 0.00 mM of (*R*)-**PGL** and 5.50 mM of (*S*)-**PGL**

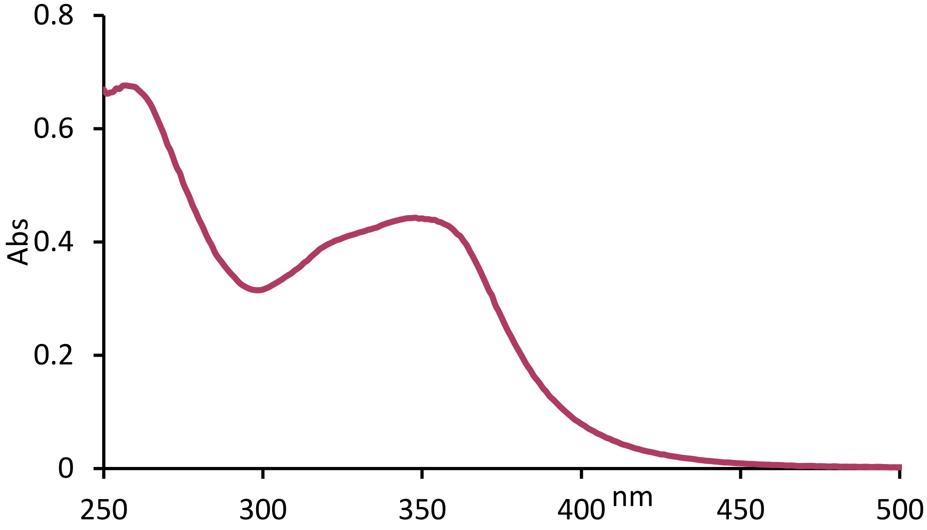

UV measurement was taken at 0.03 mM total analyte concentration in dichloroethane.

Supplementary Figure 162. UV sensing of training set 15: Mixture of 7.50 mM of (*R*)-**PEA**, 0.00 mM of (*S*)-**PEA**, 2.50 mM of (*R*)-**PMP**, 0.00 mM of (*S*)-**PMP**, 7.50 mM of (*R*)-**PPA**, 0.00 mM of (*S*)-**PPA**, 2.50 mM of (*R*)-**PGL** and 0.00 mM of (*S*)-**PGL**

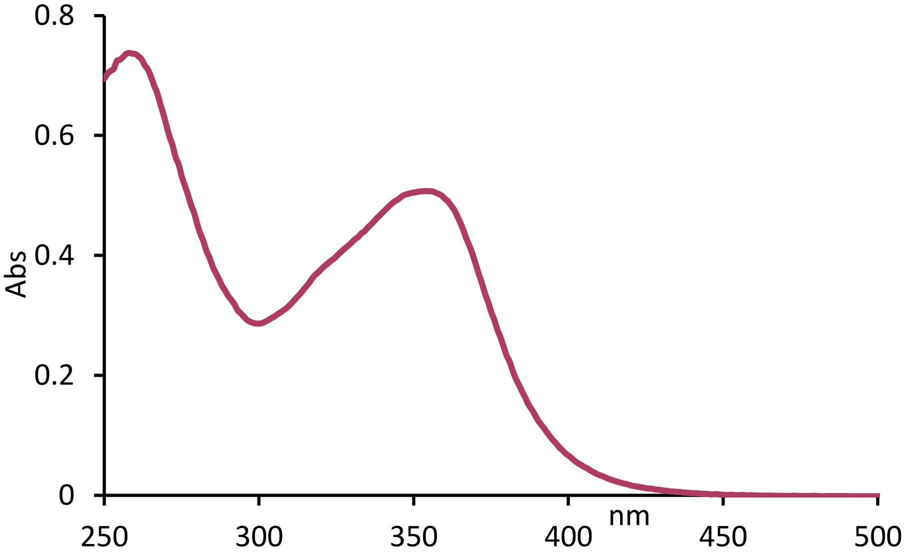

UV measurement was taken at 0.03 mM total analyte concentration in dichloroethane.

Supplementary Figure 163. UV sensing of training set 16: Mixture of 2.81 mM of (*R*)-**PEA**, 1.69 mM of (*S*)-**PEA**, 3.44 mM of (*R*)-**PMP**, 2.06 mM of (*S*)**-PMP**, 2.81 mM of (*R*)-**PPA**, 1.69 mM of (*S*)-**PPA**, 3.44 mM of (*R*)**-PGL** and 2.06 mM of (*S*)**-PGL**

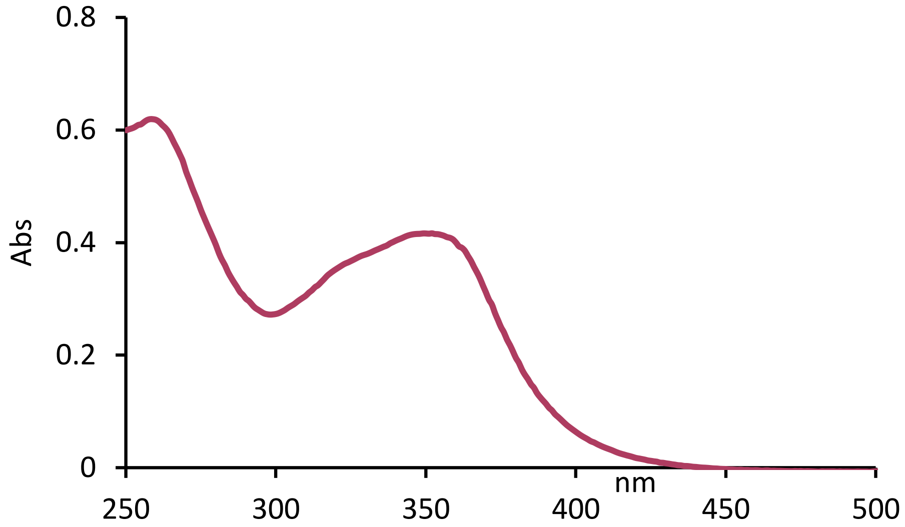

UV measurement was taken at 0.03 mM total analyte concentration in dichloroethane.

Supplementary Figure 164. UV sensing of training set 17: Mixture of 2.75 mM of (*R*)-**PEA**, 2.75 mM of (*S*)-**PEA**, 2.25 mM of (*R*)-**PMP**, 2.25 mM of (*S*)**-PMP**, 2.75 mM of (*R*)-**PPA**, 2.75 mM of (*S*)-**PPA**, 2.25 mM of (*R*)**-PGL** and 2.25 mM of (*S*)**-PGL**

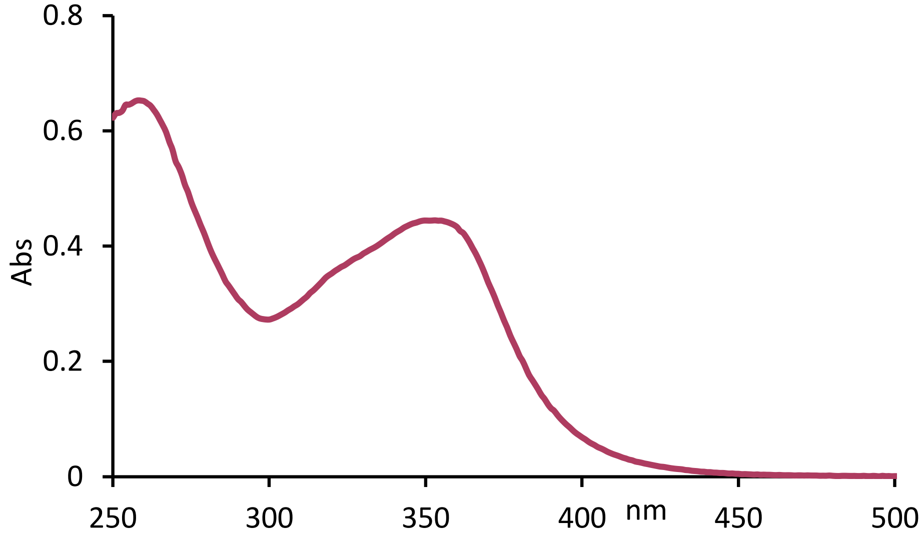

UV measurement was taken at 0.03 mM total analyte concentration in dichloroethane.

Supplementary Figure 165. UV sensing of training set 18: Mixture of 4.06 mM of (*R*)-**PEA**, 2.44 mM of (*S*)-**PEA**, 2.19 mM of (*R*)-**PMP**, 1.31 mM of (*S*)**-PMP**, 4.06 mM of (*R*)-**PPA**, 2.44 mM of (*S*)-**PPA**, 2.19 mM of (*R*)-**PGL** and 1.31 mM of (*S*)**-PGL**

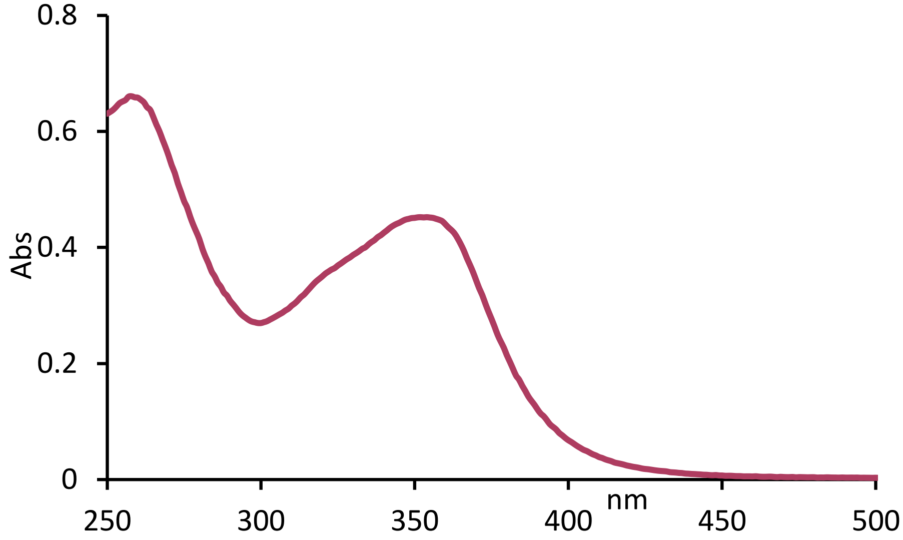

UV measurement was taken at 0.03 mM total analyte concentration in dichloroethane.

Supplementary Figure 166. UV sensing of training set 19: Mixture of 6.00 mM of (*R*)-**PEA**, 1.50 mM of (*S*)-**PEA**, 2.00 mM of (*R*)-**PMP**, 0.50 mM of (*S*)**-PMP**, 6.00 mM of (*R*)-**PPA**, 1.50 mM of (*S*)-**PPA**, 2.00 mM of (*R*)-**PGL** and 0.50 mM of (*S*)**-PGL**

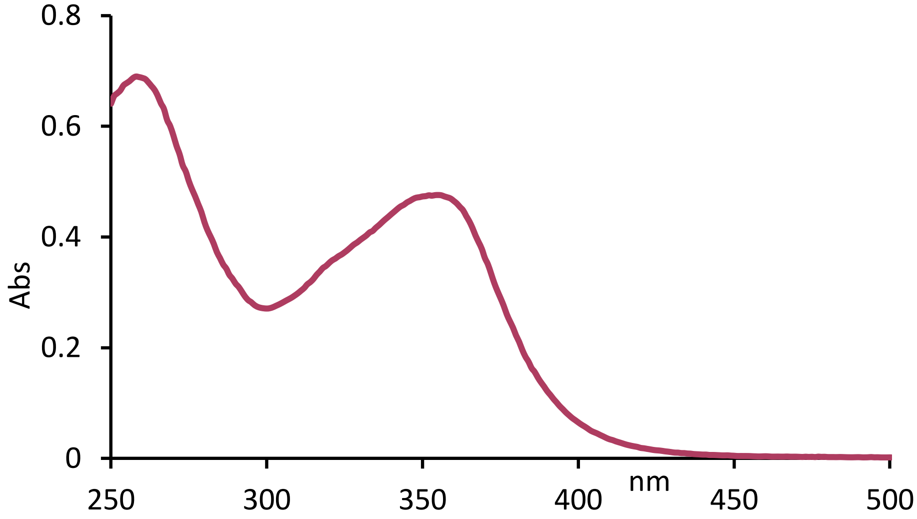

UV measurement was taken at 0.03 mM total analyte concentration in dichloroethane.

Supplementary Figure 167. UV sensing of training set 20: Mixture of 3.50 mM of (*R*)-**PEA**, 0.00 mM of (*S*)-**PEA**, 6.50 mM of (*R*)-**PMP**, 0.00 mM of (*S*)-**PMP**, 3.50 mM of (*R*)-**PPA**, 0.00 mM of (*S*)-**PPA**, 6.50 mM of (*R*)-**PGL** and 0.00 mM of (*S*)-**PGL**

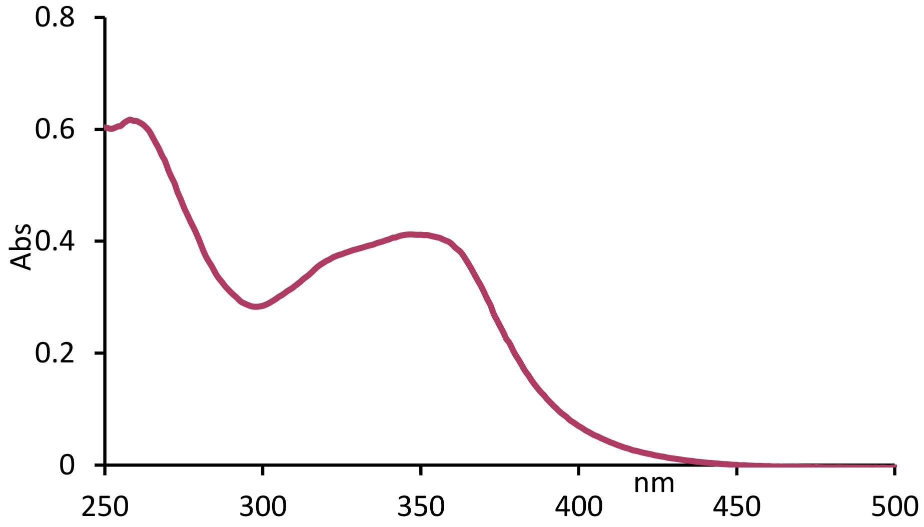

UV measurement was taken at 0.03 mM total analyte concentration in dichloroethane.

Supplementary Figure 168. UV sensing of test set 1: Mixture of 3.38 mM of (*R*)-**PEA**, 1.13 mM of (*S*)-**PEA**, 4.13 mM of (*R*)-**PMP**, 1.38 mM of (*S*)-**PMP**, 3.38 mM of (*R*)-**PPA**, 1.13 mM of (*S*)-**PPA**, 4.13 mM of (*R*)-**PGL** and 1.38 mM of (*S*)-**PGL**

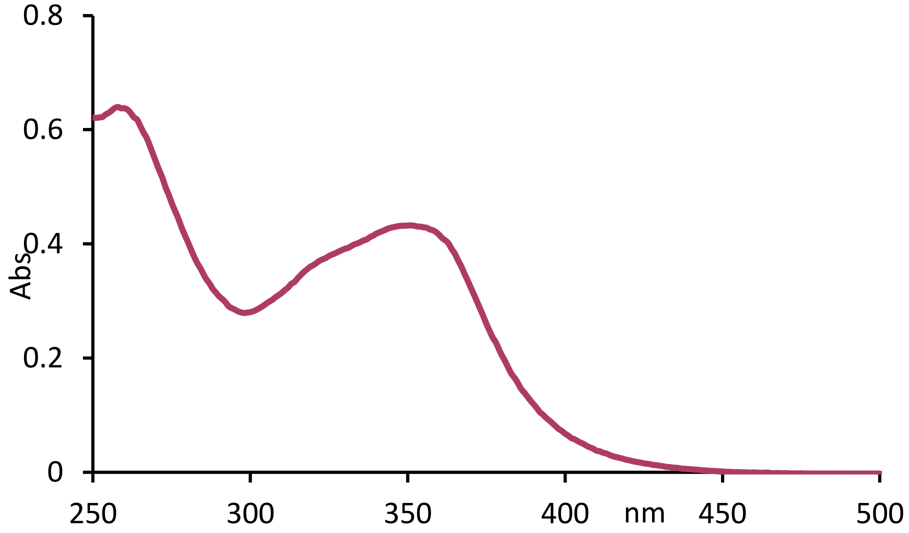

UV measurement was taken at 0.03 mM total analyte concentration in dichloroethane.

Supplementary Figure 169. UV sensing of test set 2: Mixture of 5.69 mM of (*R*)-**PEA**, 0.81 mM of (*S*)-**PEA**, 3.06 mM of (*R*)-**PMP**, 0.44 mM of (*S*)-**PMP**, 5.69 mM of (*R*)-**PPA**, 0.81 mM of (*S*)-**PPA**, 3.06 mM of (*R*)-**PGL** and 0.44 mM of (*S*)-**PGL**

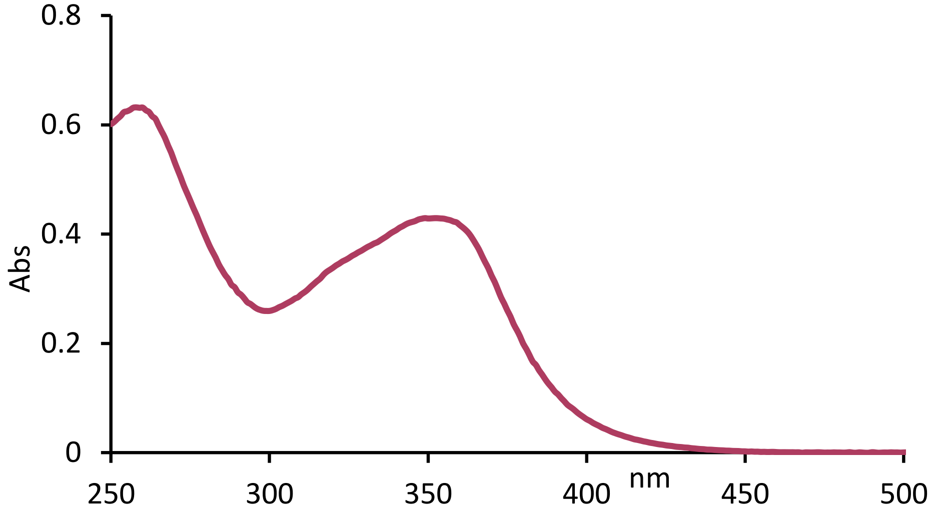

UV measurement was taken at 0.03 mM total analyte concentration in dichloroethane.

Supplementary Figure 170. UV sensing of test set 3: Mixture of 0.94 mM of (*R*)-**PEA**, 1.56 mM of (*S*)-**PEA**, 2.81 mM of (*R*)-**PMP**, 4.69 mM of (*S*)-**PMP**, 0.94 mM of (*R*)-**PPA**, 1.56 mM of (*S*)-**PPA**, 2.81 mM of (*R*)-**PGL** and 4.69 mM of (*S*)-**PGL**

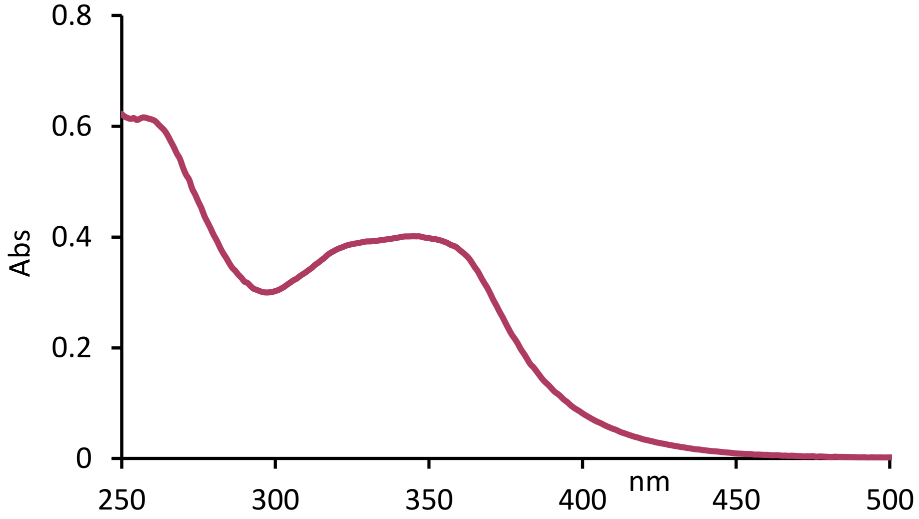

UV measurement was taken at 0.03 mM total analyte concentration in dichloroethane.

Supplementary Figure 171. UV sensing of test set 4: Mixture of 0.56 mM of (*R*)-**PEA**, 3.94 mM of (*S*)-**PEA**, 0.69 mM of (*R*)-**PMP**, 4.81 mM of (*S*)-**PMP**, 0.56 mM of (*R*)-**PPA**, 3.94 mM of (*S*)-**PPA**, 0.69 mM of (*R*)-**PGL** and 4.81 mM of (*S*)-**PGL**

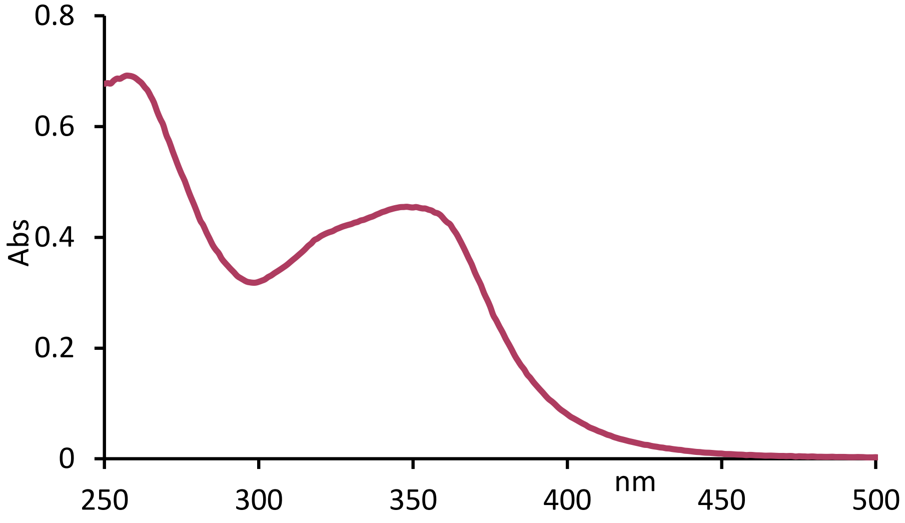

UV measurement was taken at 0.03 mM total analyte concentration in dichloroethane.

Supplementary Figure 172. UV sensing of test set 5: Mixture of 3.85 mM of (*R*)-**PEA**, 1.65 mM of (*S*)-**PEA**, 3.15 mM of (*R*)-**PMP**, 1.35 mM of (*S*)-**PMP**, 3.85 mM of (*R*)-**PPA**, 1.65 mM of **(*S*)-**PPA, 3.15 mM of (*R*)-**PGL** and 1.35 mM of (*S*)-**PGL**

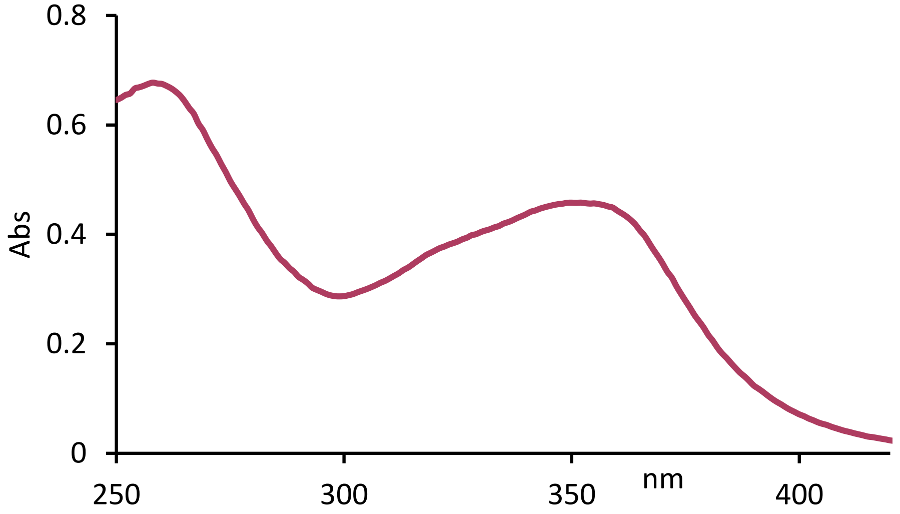

UV measurement was taken at 0.03 mM total analyte concentration in dichloroethane.

Supplementary Figure 173. UV sensing of test set 6: Mixture of 3.94 mM of (*R*)-**PEA**, 0.56 mM of (*S*)-**PEA**, 4.81 mM of (*R*)-**PMP**, 0.69 mM of (*S*)-**PMP**, 3.94 mM of (*R*)-**PPA**, 0.56 mM of (*S*)-**PPA**, 4.81 mM of (*R*)-**PGL** and 0.69 mM of (*S*)-**PGL**

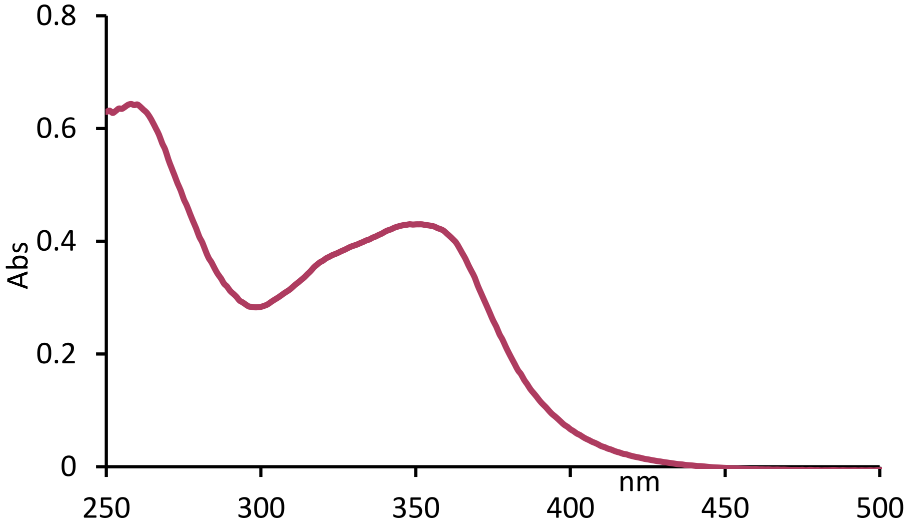

UV measurement was taken at 0.03 mM total analyte concentration in dichloroethane.

Supplementary Figure 174. UV sensing of training set 1: Mixture of 5.50 mM of (*R*)-**PEA**, 0.00 mM of (*S*)-**PEA**, 4.50 mM of (*R*)-**PMP**, 0.00 mM of (*S*)-**PMP**, 5.50 mM of (*R*)-**PPA**, 4.50 mM of (*S*)-**PPA**, 4.50 mM of (*R*)-**PGL** and 0.00 mM of (*S*)-**PGL**

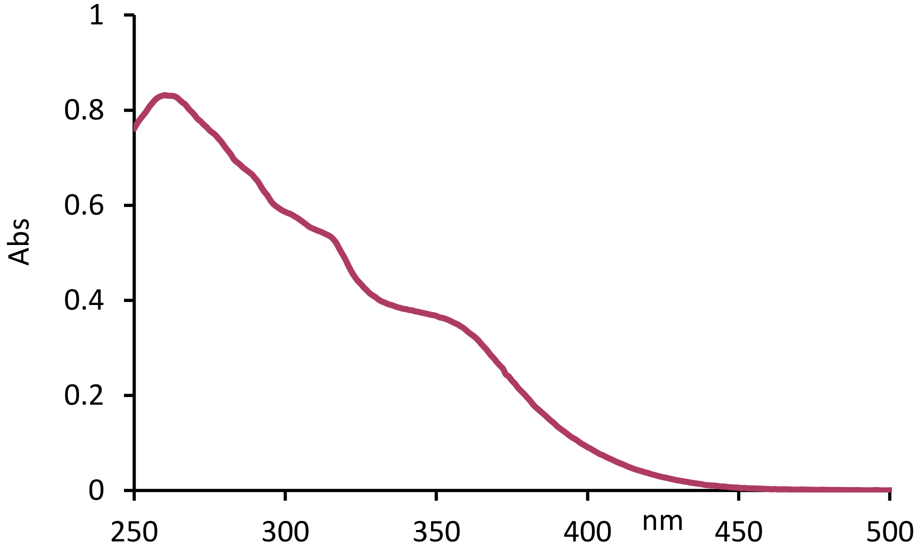

UV measurement was taken at 0.05 mM total analyte concentration in methanol.

Supplementary Figure 175. UV sensing of training set 2: Mixture of 1.88 mM of (*R*)-**PEA**, 0.63 mM of (*S*)-**PEA**, 5.63 mM of (*R*)-**PMP**, 1.88 mM of (*S*)-**PMP**, 1.88 mM of (*R*)-**PPA**, 0.63 mM of (*S*)-**PPA**, 5.63 mM of (*R*)-**PGL** and 1.88 mM of (*S*)-**PGL**

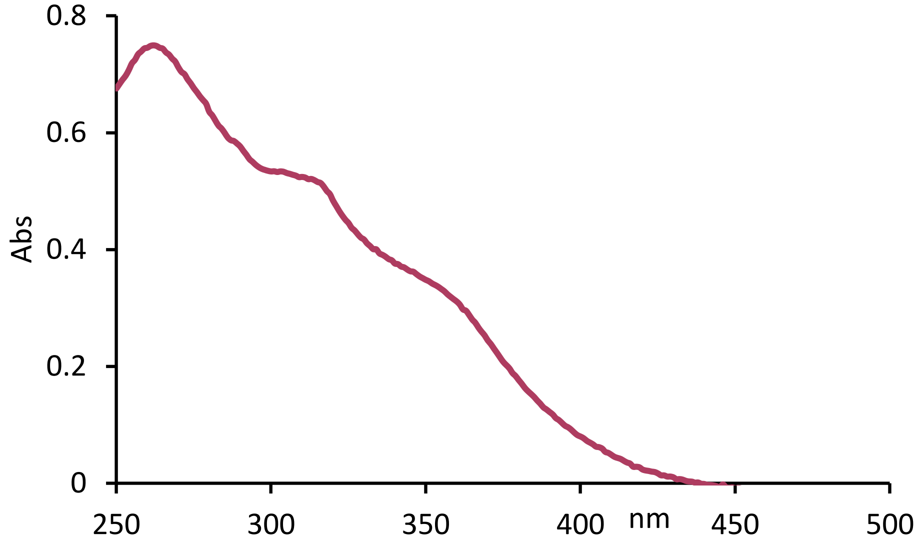

UV measurement was taken at 0.05 mM total analyte concentration in methanol.

Supplementary Figure 176. UV sensing of training set 3: Mixture of 0.70 mM of (*R*)-**PEA**, 2.80 mM of (*S*)-**PEA**, 1.30 mM of (*R*)-**PMP**, 5.20 mM of (*S*)-**PMP**, 0.70 mM of (*R*)-**PPA**, 2.80 mM of (*S*)-**PPA**, 1.30 mM of (*R*)-**PGL** and 5.20 mM of (*S*)-**PGL**

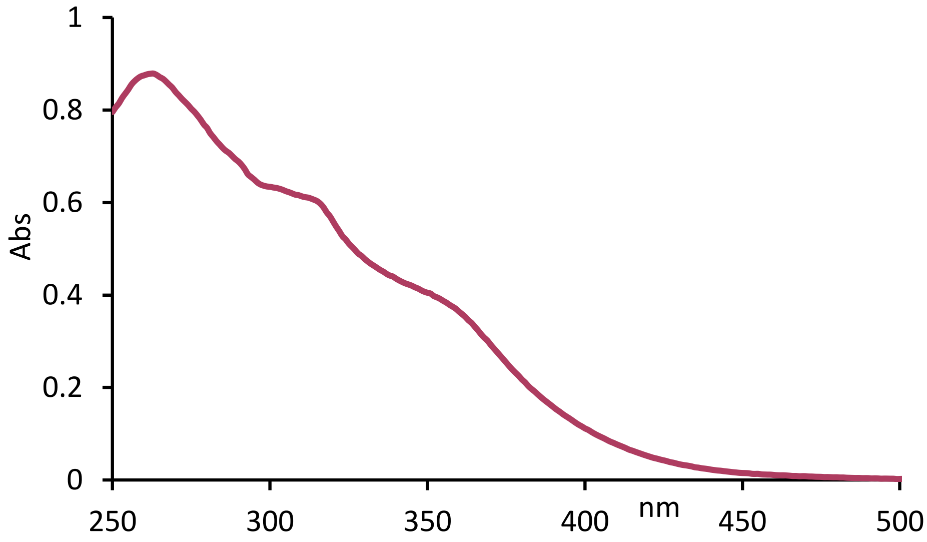

UV measurement was taken at 0.05 mM total analyte concentration in methanol.

Supplementary Figure 177. UV sensing of training set 4: Mixture of 2.80 mM of (*R*)-**PEA**, 0.70 mM of (*S*)-**PEA**, 5.20 mM of (*R*)-**PMP**,1.30 mM of (*S*)-**PMP**, 2.80 mM of (*R*)-**PPA**, 0.70 mM of (*S*)-**PPA**, 5.20 mM of (*R*)-**PGL** and 1.30 mM of (*S*)-**PGL**

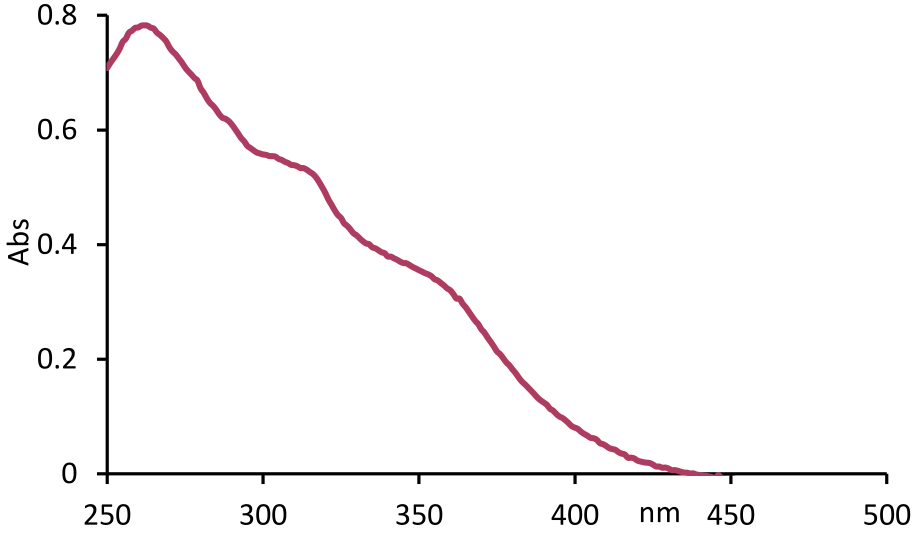

UV measurement was taken at 0.05 mM total analyte concentration in methanol.

Supplementary Figure 178. UV sensing of training set 5: Mixture of 2.19 mM of (*R*)-**PEA**, 0.31 mM of (*S*)-**PEA**, 6.56 mM of (*R*)-**PMP**, 0.94 mM of (*S*)-**PMP**, 2.19 mM of (*R*)-**PPA**, 0.31 mM of (*S*)-**PPA**, 6.56 mM of (*R*)-**PGL** and 0.94 mM of (*S*)-**PGL**

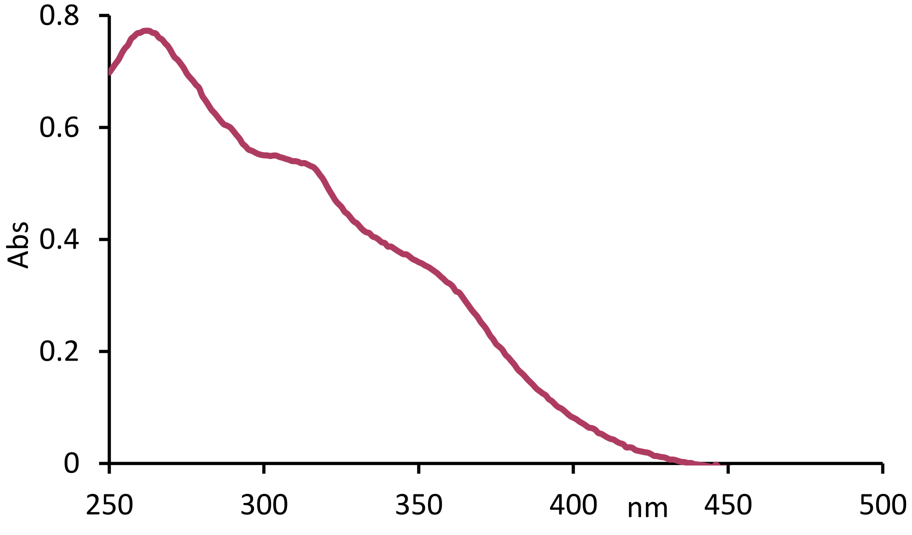

UV measurement was taken at 0.05 mM total analyte concentration in methanol.

Supplementary Figure 179. UV sensing of training set 6: Mixture of 4.40 mM of (*R*)-**PEA**, 1.10 mM of (*S*)-**PEA**, 3,60 mM of (*R*)-**PMP**, 0.90 mM of (*S*)**-PMP**, 4.40 mM of (*R*)-**PPA**, 1.10 mM of (*S*)-**PPA**, 3.60 mM of (*R*)-**PGL** and 0.90 mM of (*S*)**-PGL**

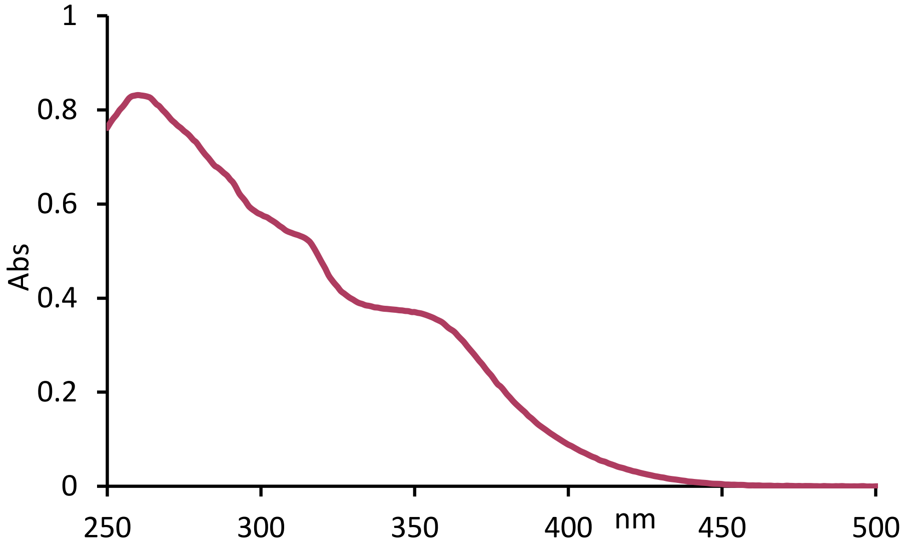

UV measurement was taken at 0.05 mM total analyte concentration in methanol.

Supplementary Figure 180. UV sensing of training set 7: Mixture of 4.88 mM of (*R*)-**PEA**, 1.63 mM of (*S*)-**PEA**, 2.63 mM of (*R*)-**PMP**, 0.88 mM of (*S*)**-PMP**, 4.88 mM of (*R*)-**PPA**, 1.63 mM of (*S*)-**PPA**, 2.63 mM of (*R*)-**PGL** and 0.88 mM of (*S*)**-PGL**

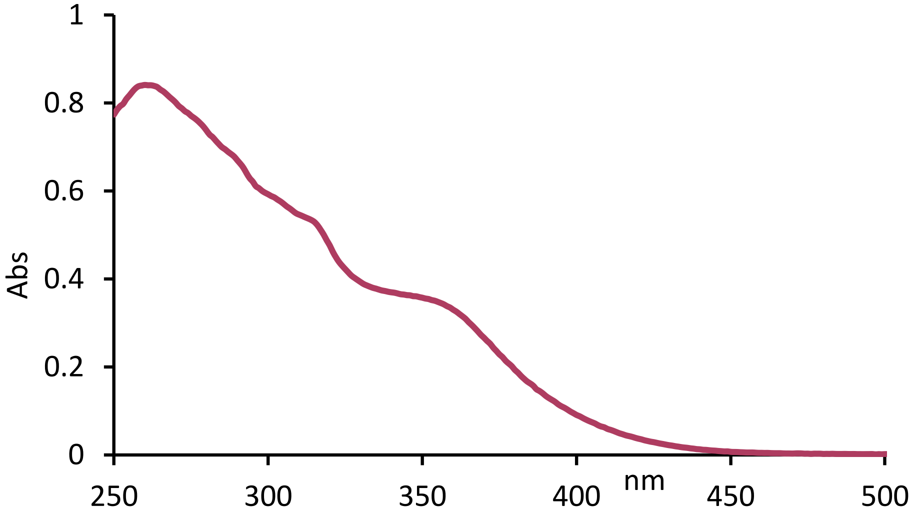

UV measurement was taken at 0.05 mM total analyte concentration in methanol.

Supplementary Figure 181. UV sensing of training set 8: Mixture of 1.75 mM of (*R*)-**PEA**, 1.75 mM of (*S*)-**PEA**, 3.25 mM of (*R*)-**PMP**, 3.25 mM of (*S*)**-PMP**, 1.75 mM of (*R*)-**PPA**, 1.75 mM of (*S*)-**PPA**, 3.25 mM of (*R*)-**PGL** and 3.25 mM of (*S*)**-PGL**

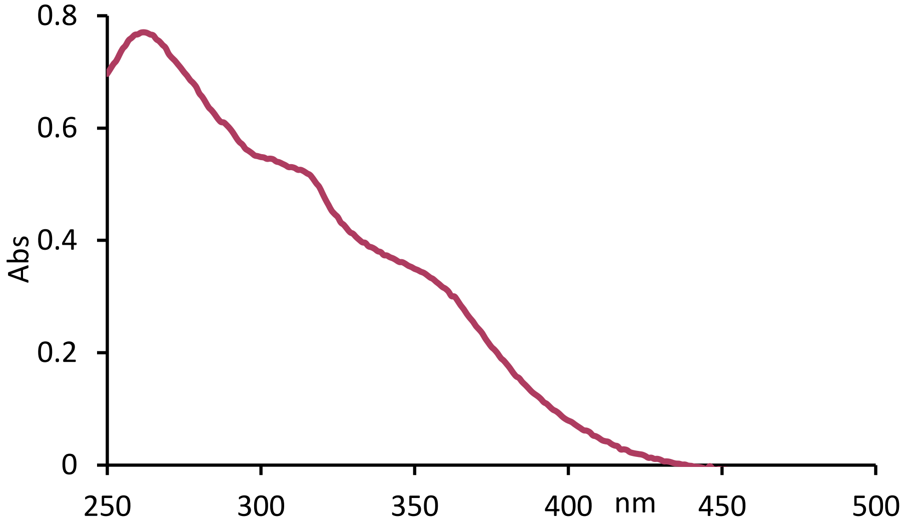

UV measurement was taken at 0.05 mM total analyte concentration in methanol.

Supplementary Figure 182. UV sensing of training set 9: Mixture of 2.45 mM of (*R*)-**PEA**, 1.05 mM of (*S*)-**PEA**, 4.55 mM of (*R*)-**PMP** and 1.95 mM of (*S*)**-PMP**, 2.45 mM of (*R*)-**PPA**, 1.05 mM of (*S*)-**PPA**, 4.55 mM of (*R*)-**PGL** and 1.95 mM of (*S*)**-PGL**

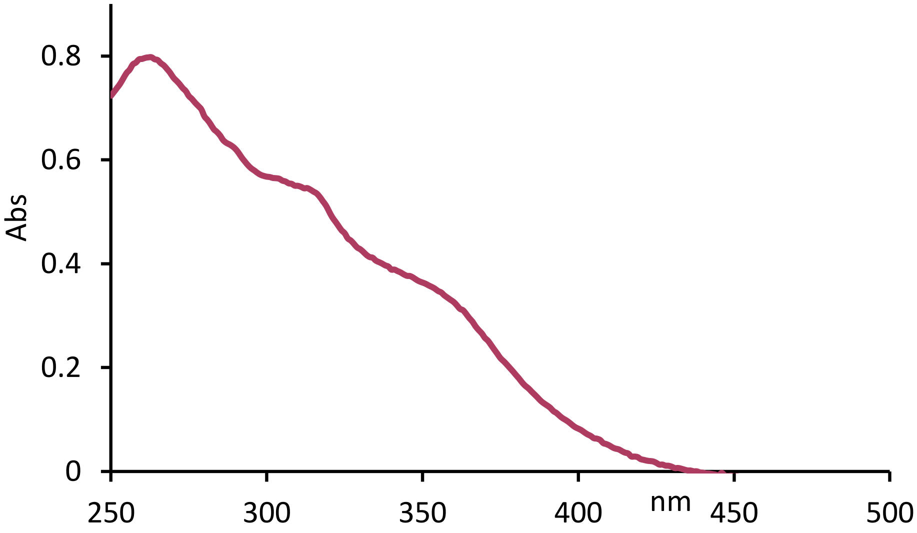

UV measurement was taken at 0.05 mM total analyte concentration in methanol.

Supplementary Figure 183. UV sensing of training set 10: Mixture of 0.75 mM of (*R*)-**PEA**, 1.75 mM of (*S*)-**PEA**, 2.25 mM of (*R*)-**PMP**, 5.25 mM of (*S*)-**PMP**, 0.75 mM of (*R*)-**PPA**, 1.75 mM of (*S*)-**PPA**, 2.25 mM of (*R*)-**PGL** and 5.25 mM of (*S*)-**PGL**

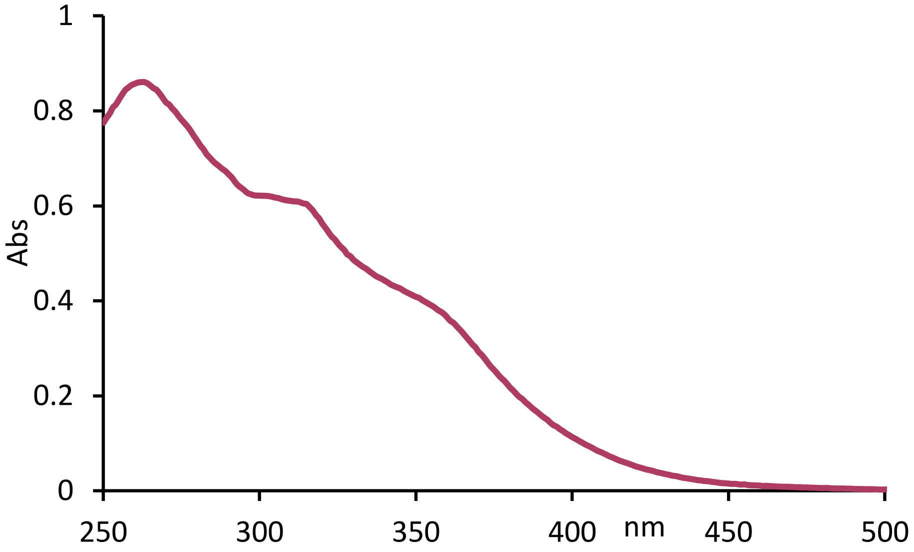

UV measurement was taken at 0.05 mM total analyte concentration in methanol.

Supplementary Figure 184. UV sensing of training set 11: Mixture of 3.75 mM of (*R*)-**PEA**, 3.75 mM of (*S*)-**PEA**, 1.25 mM of (*R*)-**PMP**, 1.25 mM of (*S*)-**PMP**, 3.75 mM of (*R*)-**PPA**, 3.75 mM of (*S*)-**PPA**, 1.25 mM of (*R*)-**PGL** and 1.25 mM of (*S*)-**PGL**

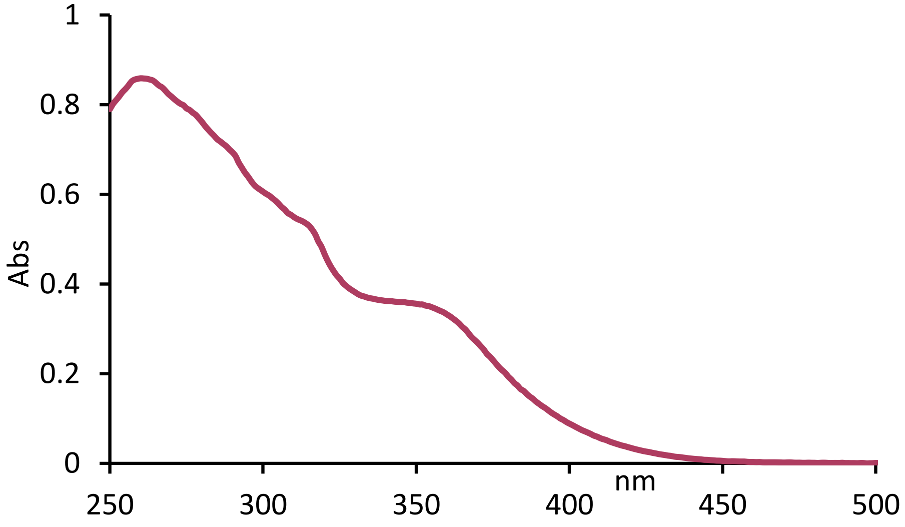

UV measurement was taken at 0.05 mM total analyte concentration in methanol.

Supplementary Figure 185. UV sensing of training set 12: Mixture of 0.63 mM of (*R*)-**PEA**, 1.88 mM of (*S*)-**PEA**, 1.75 mM of (*R*)-**PMP**, 0.75 mM of (*S*)**-PMP**, 5.25 mM of (*R*)-**PPA**, 2.25 mM of (*S*)-**PPA**, 1.75 mM of (*R*)-**PGL** and 0.75 mM of (*S*)**-PGL**

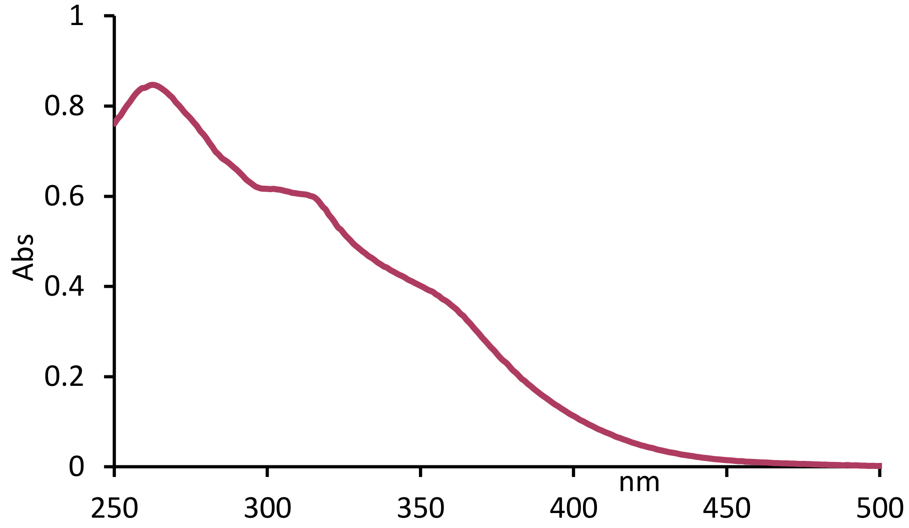

UV measurement was taken at 0.05 mM total analyte concentration in methanol.

Supplementary Figure 186. UV sensing of training set 13: Mixture of 5.25 mM of (*R*)-**PEA**, 2.25 mM of (*S*)-**PEA**, 1.75 mM of (*R*)-**PMP**, 0.75 mM of (*S*)**-PMP**, 5.25 mM of (*R*)-**PPA**, 2.25 mM of (*S*)-**PPA**, 1.75 mM of (*R*)-**PGL** and 0.75 mM of (*S*)**-PGL**

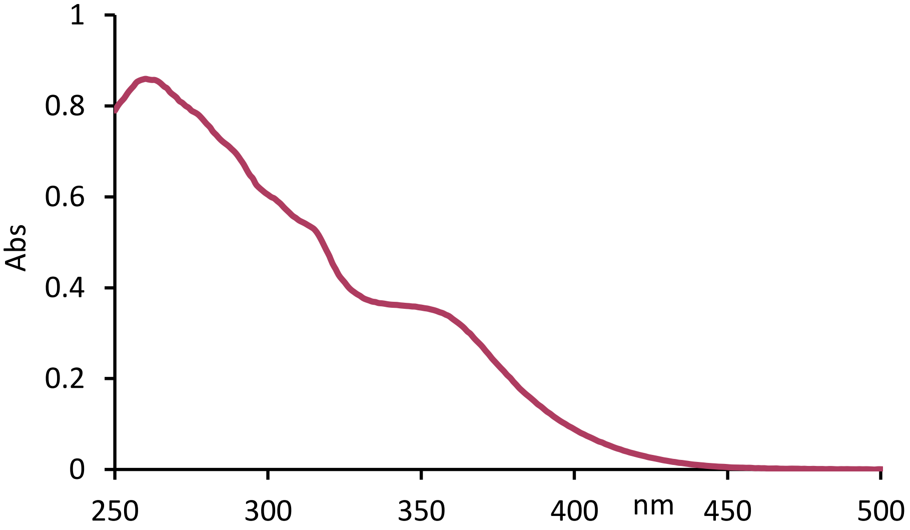

UV measurement was taken at 0.05 mM total analyte concentration in methanol.

Supplementary Figure 187. UV sensing of training set 14: Mixture of 0.00 mM of (*R*)-**PEA**, 4.50 mM of (*S*)-**PEA**, 0.00 mM of (*R*)-**PMP**, 5.50 mM of (*S*)-**PMP**, 0.00 mM of (*R*)-**PPA**, 4.50 mM of (*S*)-**PPA**, 0.00 mM of (*R*)-**PGL** and 5.50 mM of (*S*)-**PGL**

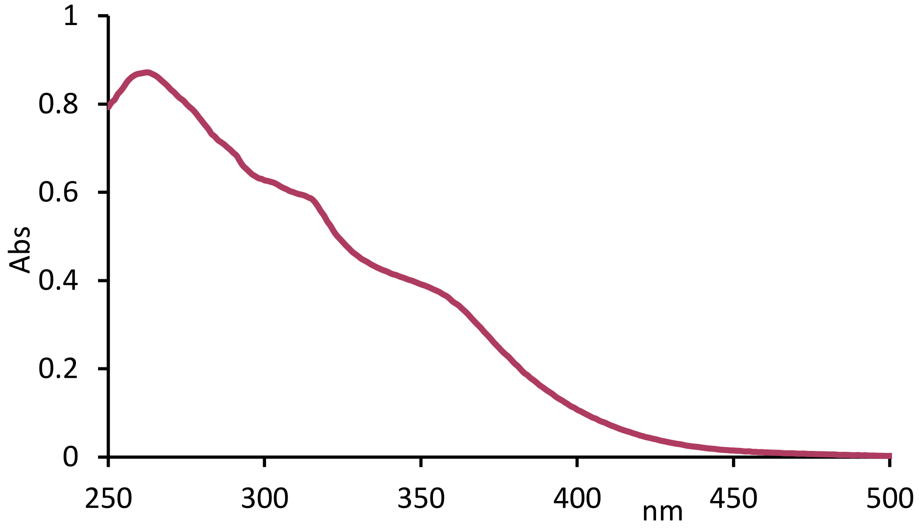

UV measurement was taken at 0.05 mM total analyte concentration in methanol.

Supplementary Figure 188. UV sensing of training set 15: Mixture of 7.50 mM of (*R*)-**PEA**, 0.00 mM of (*S*)-**PEA**, 2.50 mM of (*R*)-**PMP**, 0.00 mM of (*S*)-**PMP**, 7.50 mM of (*R*)-**PPA**, 0.00 mM of (*S*)-**PPA**, 2.50 mM of (*R*)-**PGL** and 0.00 mM of (*S*)-**PGL**

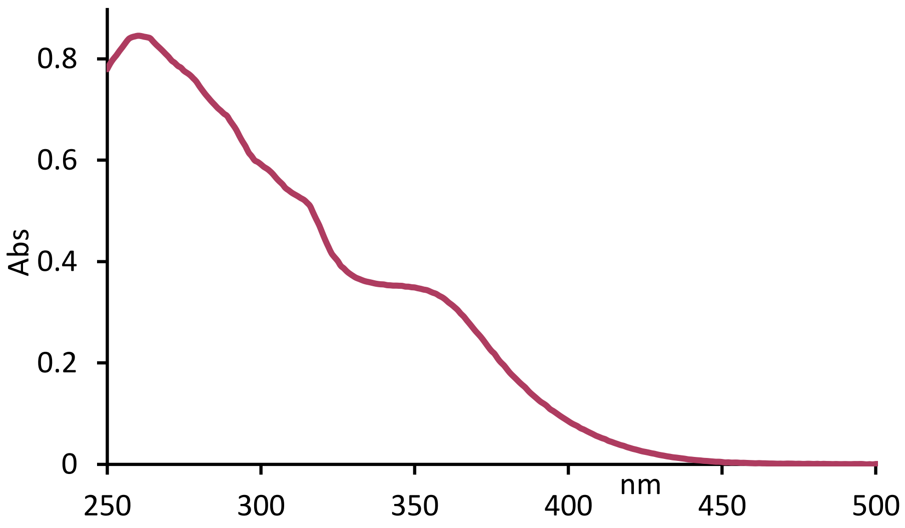

UV measurement was taken at 0.05 mM total analyte concentration in methanol.

Supplementary Figure 189. UV sensing of training set 16: Mixture of 2.81 mM of (*R*)-**PEA**, 1.69 mM of (*S*)-**PEA**, 3.44 mM of (*R*)-**PMP**, 2.06 mM of (*S*)-**PMP**, 2.81 mM of (*R*)-**PPA**, 1.69 mM of (*S*)-**PPA**, 3.44 mM of (*R*)-**PGL** and 2.06 mM of (*S*)-**PGL**

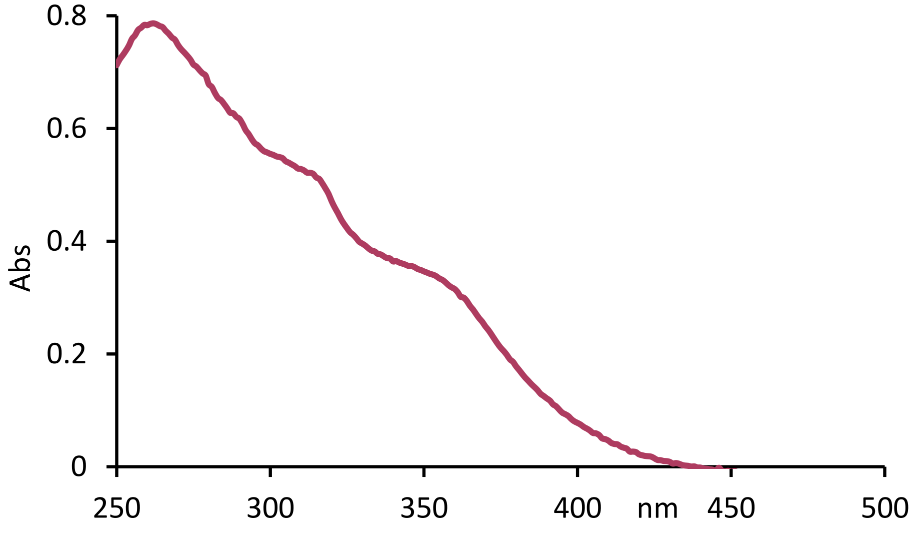

UV measurement was taken at 0.05 mM total analyte concentration in methanol.

Supplementary Figure 190. UV sensing of training set 17: Mixture of 2.75 mM of (*R*)-**PEA**, 2.75 mM of (*S*)-**PEA**, 2.25 mM of (*R*)-**PMP**, 2.25 mM of (*S*)-**PMP**, 2.75 mM of (*R*)-**PPA**, 2.75 mM of (*S*)-**PPA**, 2.25 mM of (*R*)-**PGL** and 2.25 mM of (*S*)-**PGL**

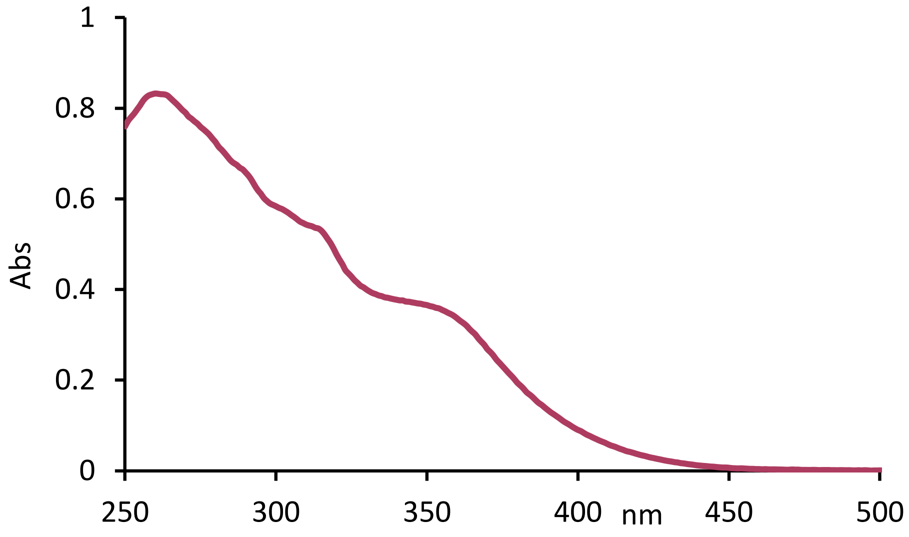

UV measurement was taken at 0.05 mM total analyte concentration in methanol.

Supplementary Figure 191. UV sensing of training set 18: Mixture of 4.06 mM of (*R*)-**PEA**, 2.44 mM of (*S*)-**PEA**, 2.19 mM of (*R*)-**PMP**, 1.31 mM of (*S*)-**PMP**, 4.06 mM of (*R*)-**PPA**, 2.44 mM of (*S*)-**PPA**, 2.19 mM of (*R*)-**PGL** and 1.31 mM of (*S*)-**PGL**

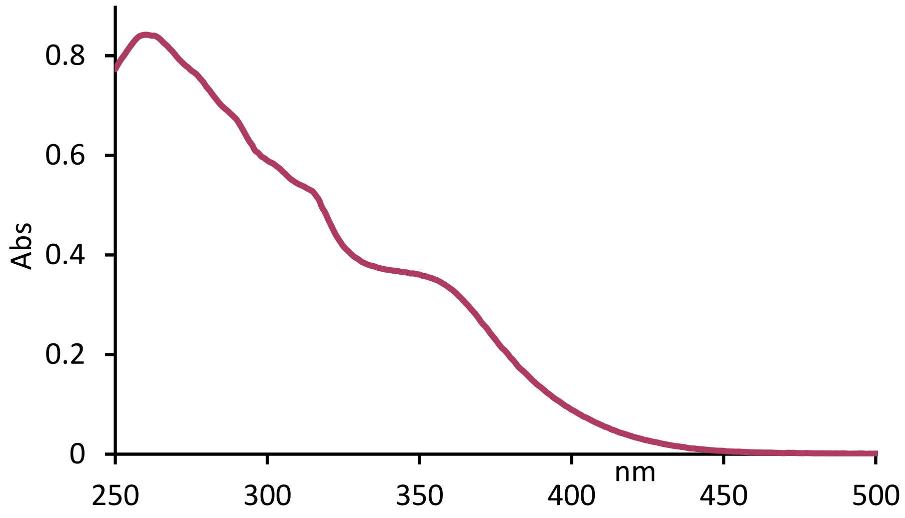

UV measurement was taken at 0.05 mM total analyte concentration in methanol.

Supplementary Figure 192. UV sensing of training set 19: Mixture of 6.00 mM of (*R*)-**PEA**, 1.50 mM of (*S*)-**PEA**, 2.00 mM of (*R*)-**PMP**, 0.50 mM of (*S*)-**PMP**, 6.00 mM of (*R*)-**PPA**, 1.50 mM of (*S*)-**PPA**, 2.00 mM of (*R*)-**PGL** and 0.50 mM of (*S*)-**PGL**

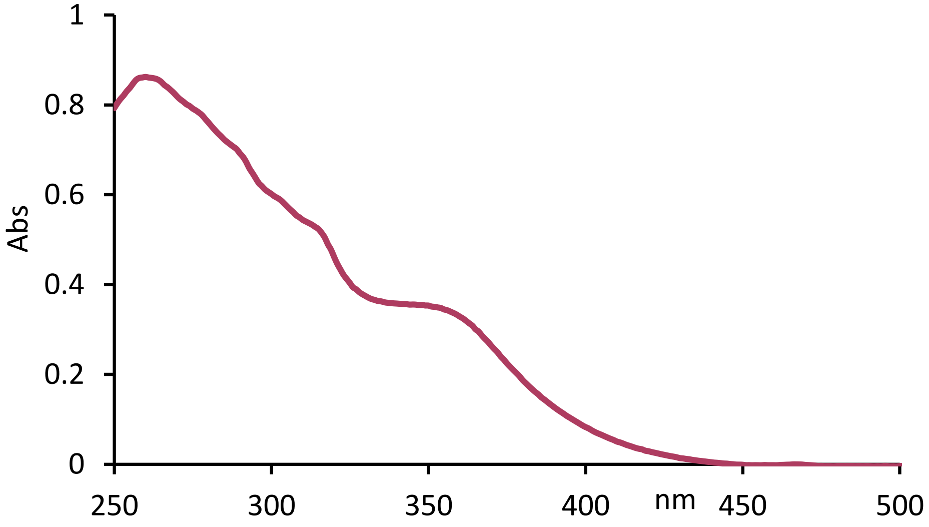

UV measurement was taken at 0.05 mM total analyte concentration in methanol.

Supplementary Figure 193. UV sensing of training set 20: Mixture of 3.50 mM of (*R*)-**PEA**, 0.00 mM of (*S*)-**PEA**, 6.50 mM of (*R*)-**PMP**, 0.00 mM of (*S*)-**PMP**, 3.50 mM of (*R*)-**PPA**, 0.00 mM of (*S*)-**PPA**, 6.50 mM of (*R*)-**PGL** and 0.00 mM of (*S*)-**PGL**

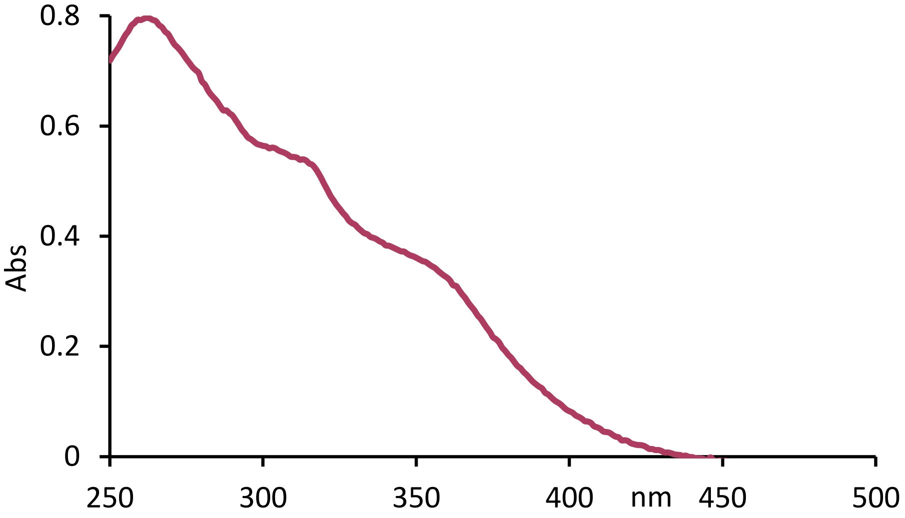

UV measurement was taken at 0.05 mM total analyte concentration in methanol.

Supplementary Figure 194. UV sensing of test set 1: Mixture of 3.38 mM of (*R*)-**PEA**, 1.13 mM of (*S*)-**PEA**, 4.13 mM of (*R*)-**PMP**, 1.38 mM of (*S*)-**PMP**, 3.38 mM of (*R*)-**PPA**, 1.13 mM of (*S*)-**PPA**, 4.13 mM of (*R*)-**PGL** and 1.38 mM of (*S*)-**PGL**

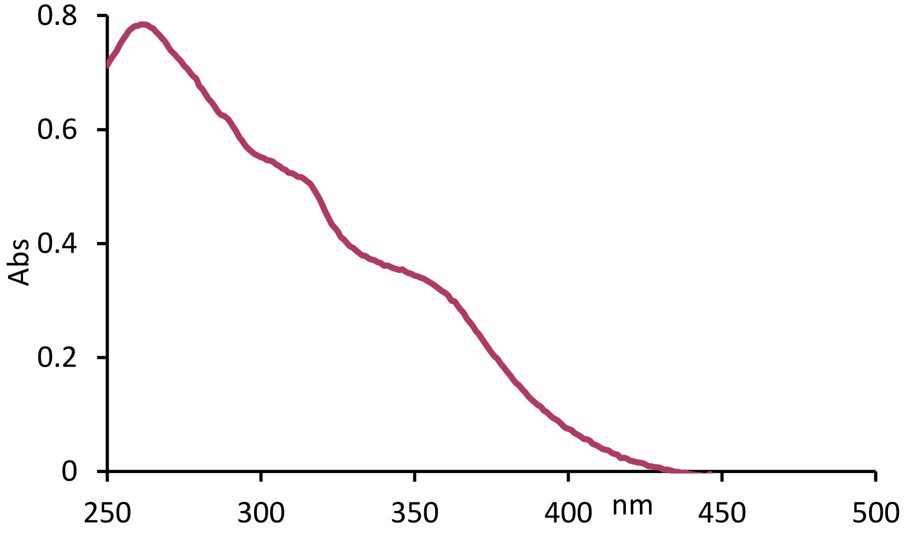

UV measurement was taken at 0.05 mM total analyte concentration in methanol.

Supplementary Figure 195. UV sensing of test set 2: Mixture of 5.69 mM of (*R*)-**PEA**, 0.81 mM of (*S*)-**PEA**, 3.06 mM of (*R*)-**PMP**, 0.44 mM of (*S*)-**PMP**, 5.69 mM of (*R*)-**PPA**, 0.81 mM of (*S*)-**PPA**, 3.06 mM of (*R*)-**PGL** and 0.44 mM of (*S*)-**PGL**

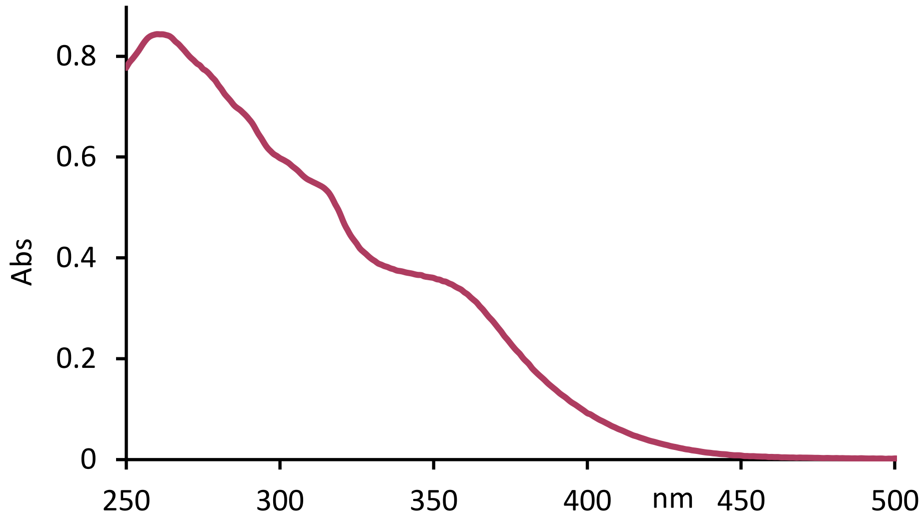

UV measurement was taken at 0.05 mM total analyte concentration in methanol.

Supplementary Figure 196. UV sensing of test set 3: Mixture of 0.94 mM of (*R*)-**PEA**, 1.56 mM of (*S*)-**PEA**, 2.81 mM of (*R*)-**PMP**, 4.69 mM of (*S*)-**PMP**, 0.94 mM of (*R*)-**PPA**, 1.56 mM of (*S*)-**PPA**, 2.81 mM of (*R*)-**PGL** and 4.69 mM of (*S*)-**PGL**

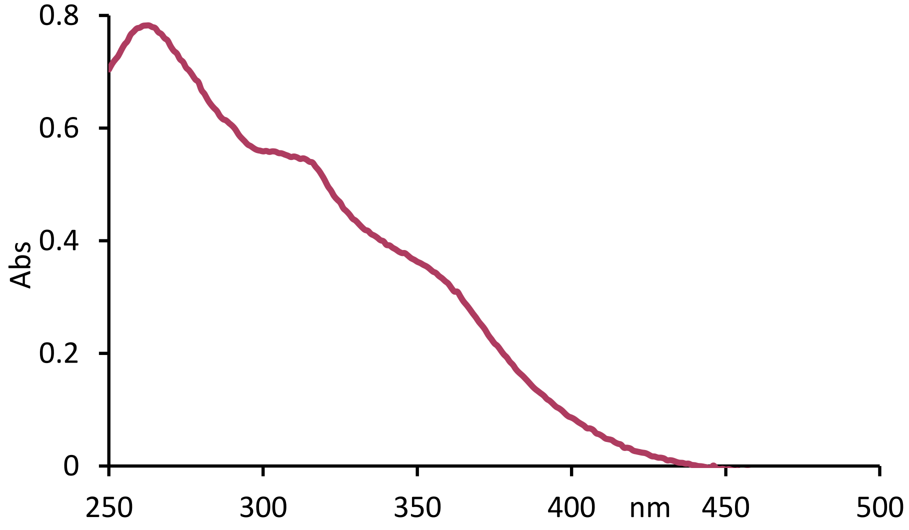

UV measurement was taken at 0.05 mM total analyte concentration in methanol.

Supplementary Figure 197. UV sensing of test set 4: Mixture of 0.56 mM of (*R*)-**PEA**, 3.94 mM of (*S*)-**PEA**, 0.69 mM of (*R*)-**PMP**, 4.81 mM of (*S*)-**PMP**, 0.56 mM of (*R*)-**PPA**, 3.94 mM of (*S*)-**PPA**, 0.69 mM of (*R*)-**PGL** and 4.81 mM of (*S*)-**PGL**

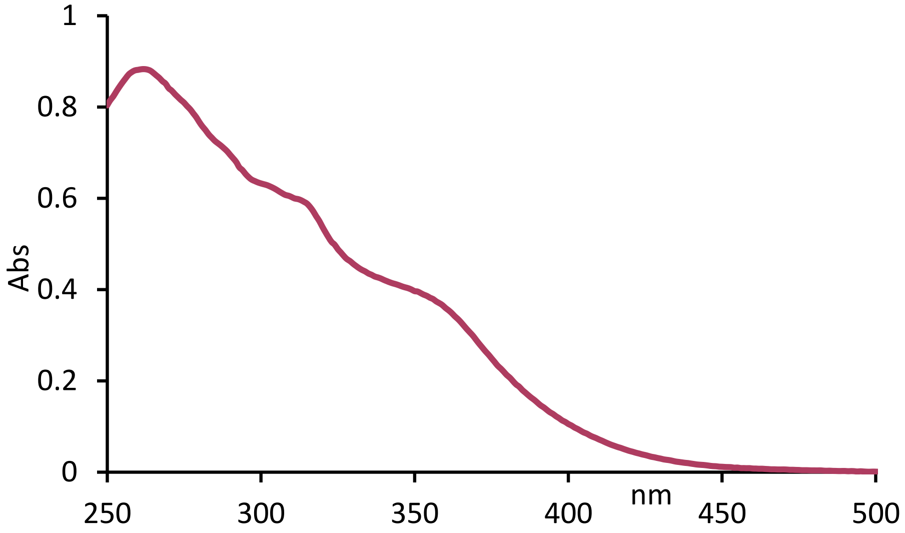

UV measurement was taken at 0.05 mM total analyte concentration in methanol.

Supplementary Figure 198. UV sensing of test set 5: Mixture of 3.85 mM of (*R*)-**PEA**, 1.65 mM of (*S*)-**PEA**, 3.15 mM of (*R*)-**PMP**, 1.35 mM of (*S*)-**PMP**, 3.85 mM of (*R*)-**PPA**, 1.65 mM of (*S*)-**PPA**, 3.15 mM of (*R*)-**PGL** and 1.35 mM of (*S*)-**PGL**

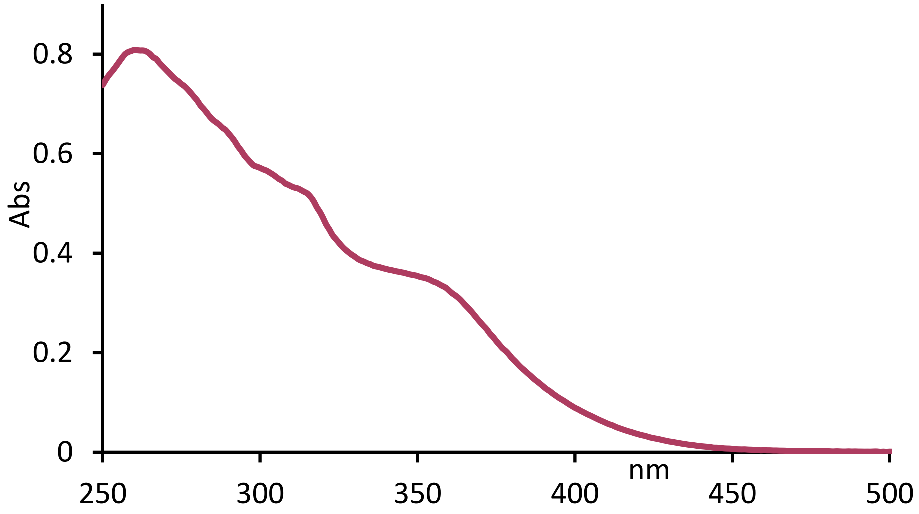

UV measurement was taken at 0.05 mM total analyte concentration in methanol.

Supplementary Figure 199. UV sensing of test set 6: Mixture of 3.94 mM of (*R*)-**PEA**, 0.56 mM of (*S*)-**PEA**, 4.81 mM of (*R*)-**PMP**, 0.69 mM of (*S*)-**PMP**, 3.94 mM of (*R*)-**PPA**, 0.56 mM of (*S*)-**PPA**, 4.81 mM of (*R*)-**PGL** and 0.69 mM of (*S*)-**PGL**

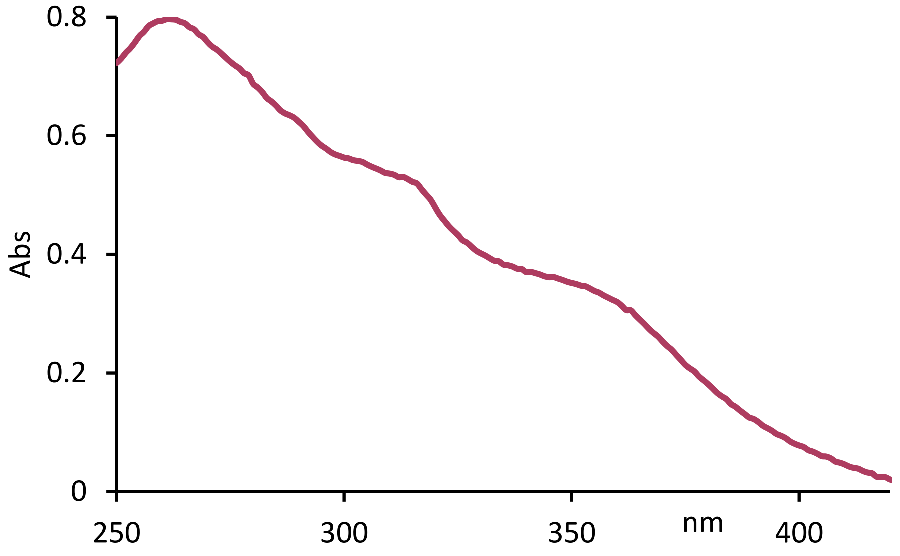

UV measurement was taken at 0.05 mM total analyte concentration in methanol.

Supplementary Figure 200. Number of latent variable versus averaged root mean squared error (RMSE) in training sets for (*R*)-**PEA** using leave-one-out cross-validation

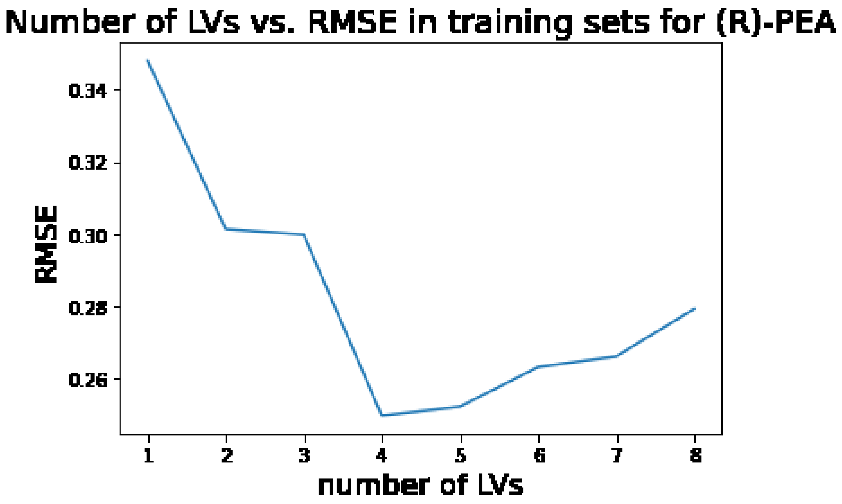

Supplementary Figure 201. Number of latent variables versus averaged root mean squared error (RMSE) in training sets for (*S*)-**PEA** using leave-one-out cross-validation

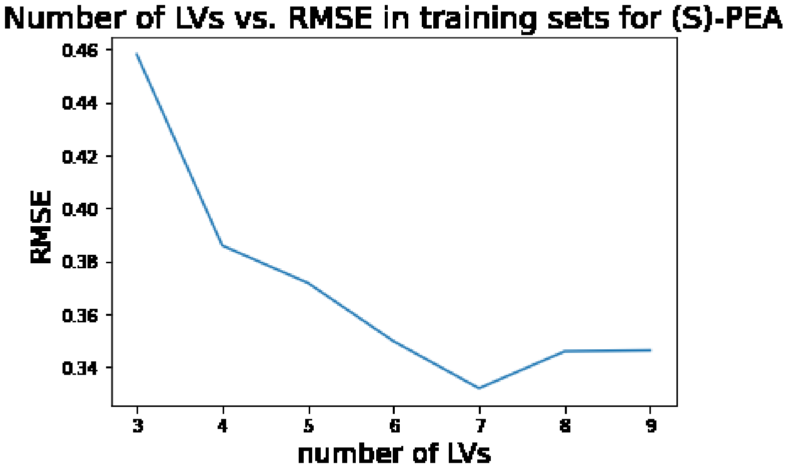

Supplementary Figure 202. Number of latent variables versus root mean-squared error (RMSE) for (*R*)-**PMP** training sets using leave-one-out cross-validation (LOOCV)

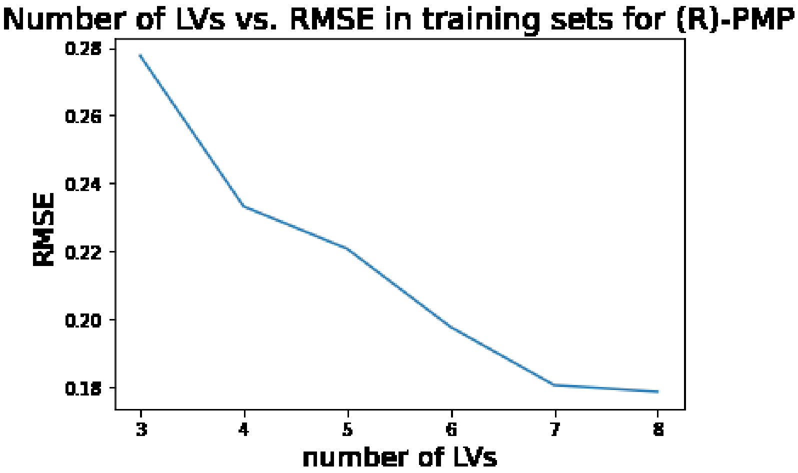

Supplementary Figure 203. Number of latent variable versus averaged root-mean-square (RMSE) error in training sets for (*S*)-**PMP** using leave-one-out cross-validation

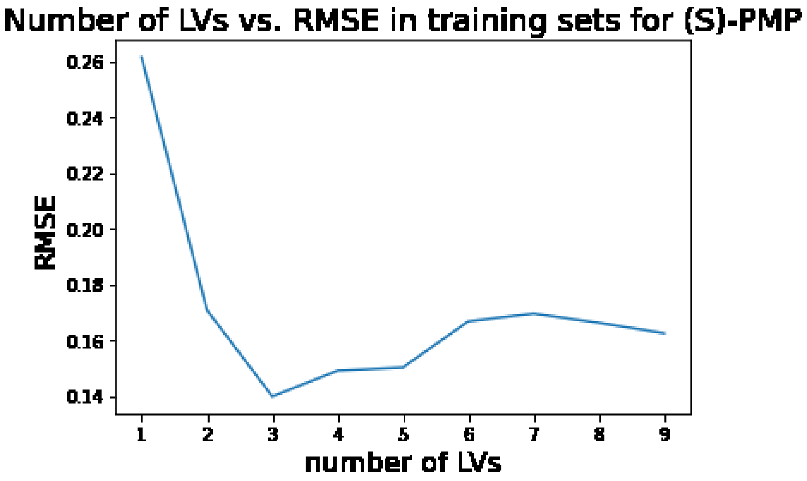

Supplementary Figure 204. Number of latent variables versus averaged root-mean-square (RMSE) error in training sets for (*R*)-**PPA** using leave-one-out cross-validation

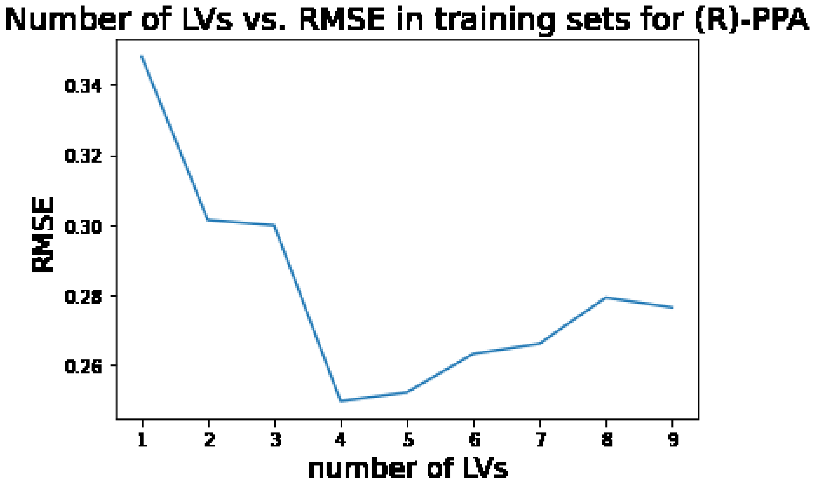

Supplementary Figure 205. Number of latent variables versus averaged root-mean-square (RMSE) error in training sets for (*S*)-**PPA** using leave-one-out cross-validation

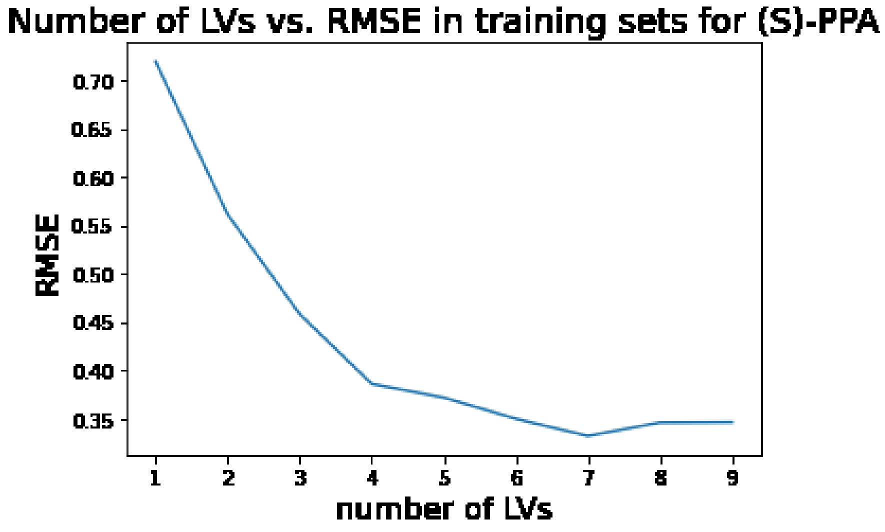

Supplementary Figure 206. Number of latent variables versus averaged root-mean-square (RMSE) error in training sets for (*R*)-**PGL** using leave-one-out cross-validation

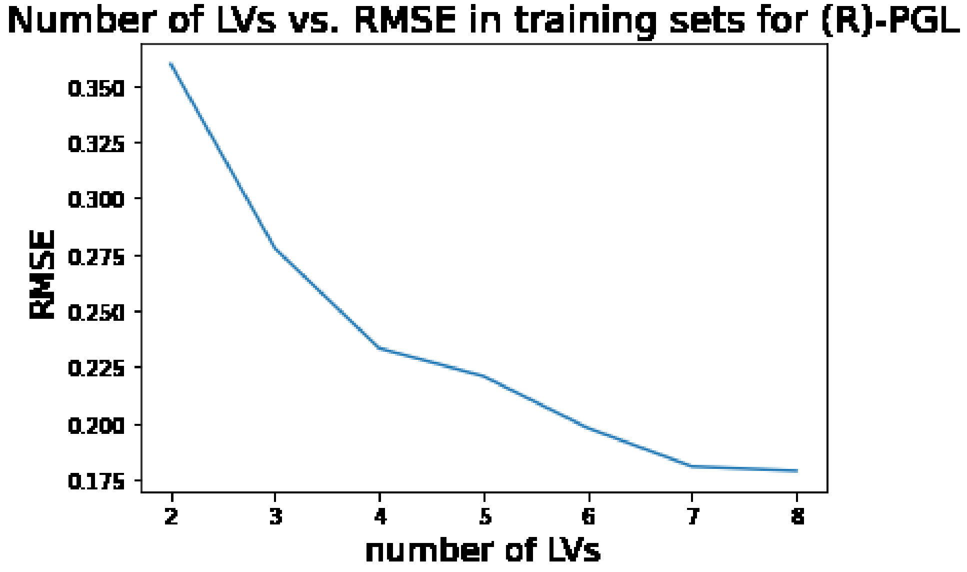

Supplementary Figure 207. Number of latent variables versus averaged root-mean-square (RMSE) error in training sets for (*S*)-**PGL** using leave-one-out cross-validation

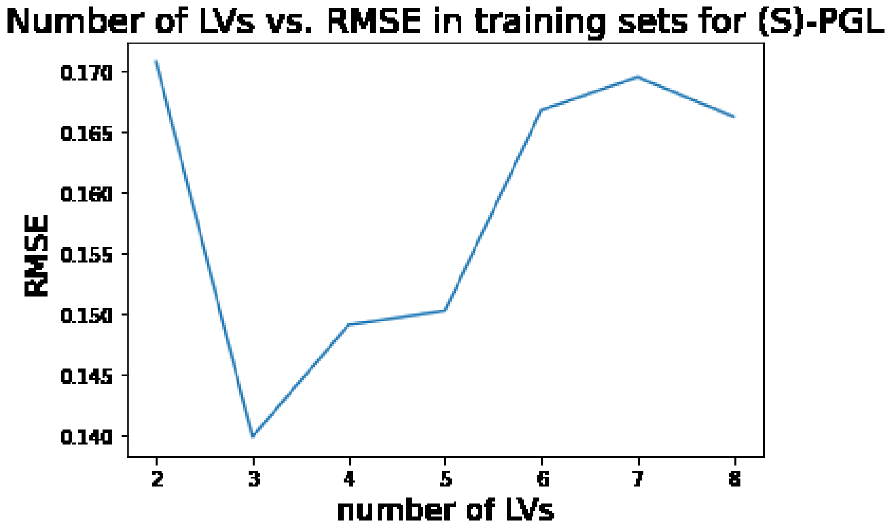

Supplementary Figure 208. MBPLS block loadings for CD data in dichloroethane (DCE) for analysis of (*R*)-**PEA**

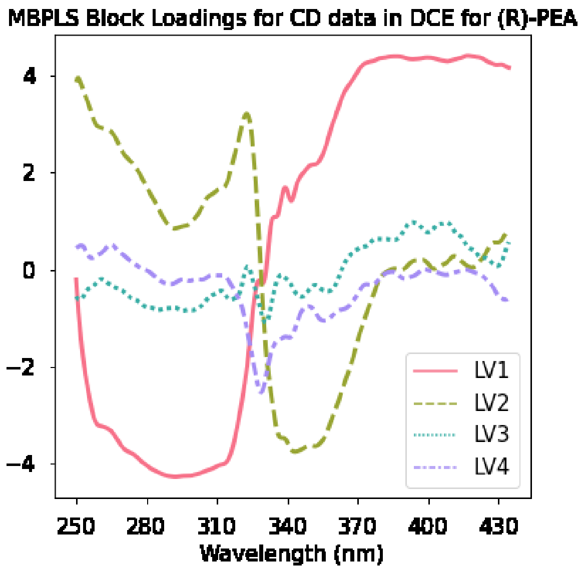

Supplementary Figure 209. MBPLS block loadings for UV data in dichloroethane (DCE) for analysis of (*R*)-**PEA**

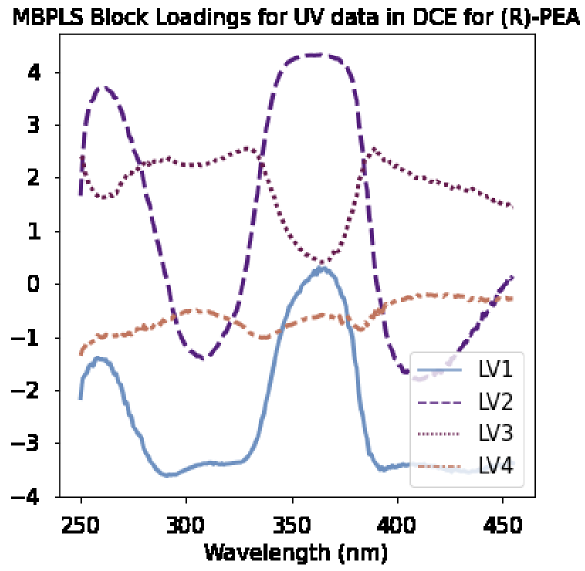

Supplementary Figure 210. MBPLS block loadings for UV data in methanol (MeOH) for analysis of (*R*)-**PEA**

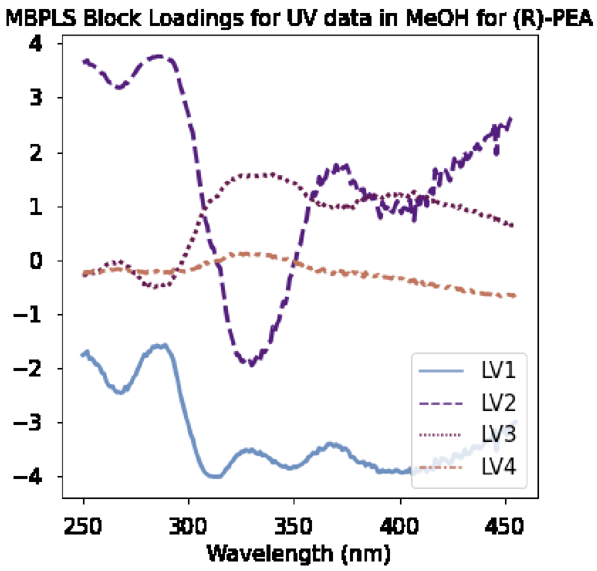

Supplementary Figure 211. MBPLS block loadings for CD data in methanol (MeOH) for analysis of (*R*)-**PEA**

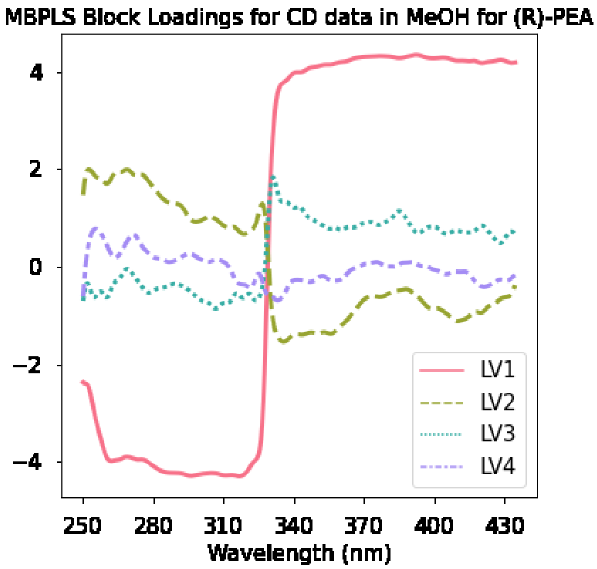

Figure 212. Number of latent variables versus averaged root-mean-square (RMSE) error in training sets for (*R*)-**PEA** using leave-one-out cross-validation for LASSO + PCR

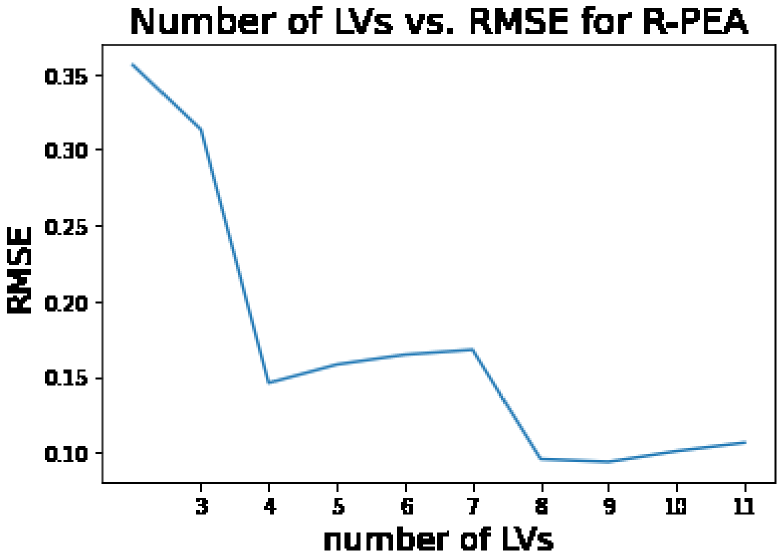

Figure 213. Number of latent variables versus averaged root-mean-square (RMSE) error in training sets for (*S*)-**PEA** using leave-one-out cross-validation for LASSO + PCR

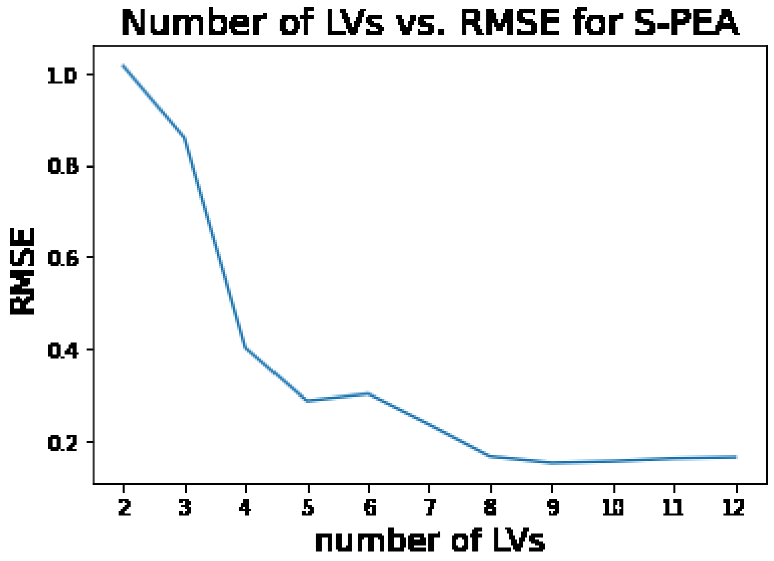

Figure 214. Number of latent variables versus averaged root-mean-square (RMSE) error in training sets for (*R*)-**PMP** using leave-one-out cross-validation for LASSO + PCR

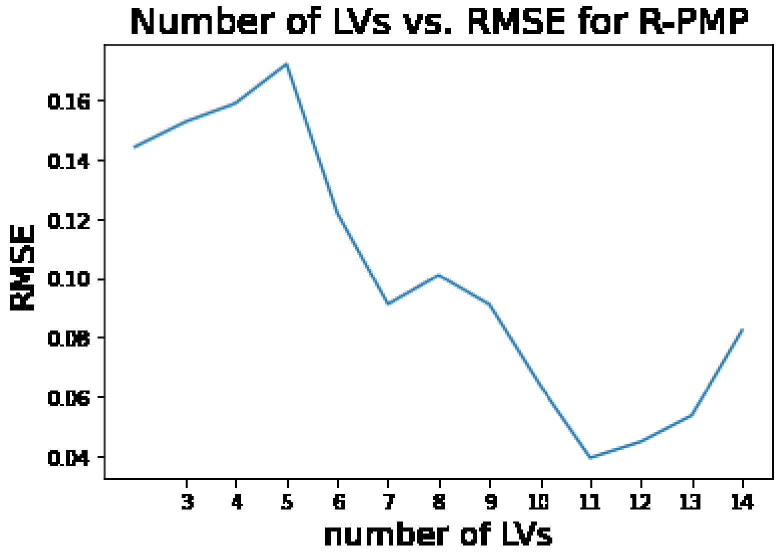

Figure 215. Number of latent variables versus averaged root-mean-square (RMSE) error in training sets for (*S*)-**PMP** using leave-one-out cross-validation for LASSO + PCR

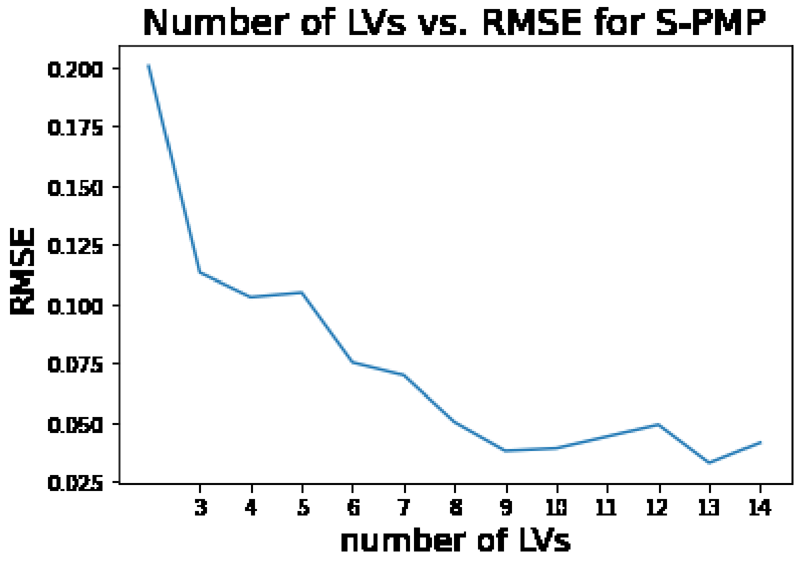

Figure 216. Number of latent variables versus averaged root-mean-square (RMSE) error in training sets for (1*R*, 2*S*)-**PPA** using leave-one-out cross-validation for LASSO + PCR

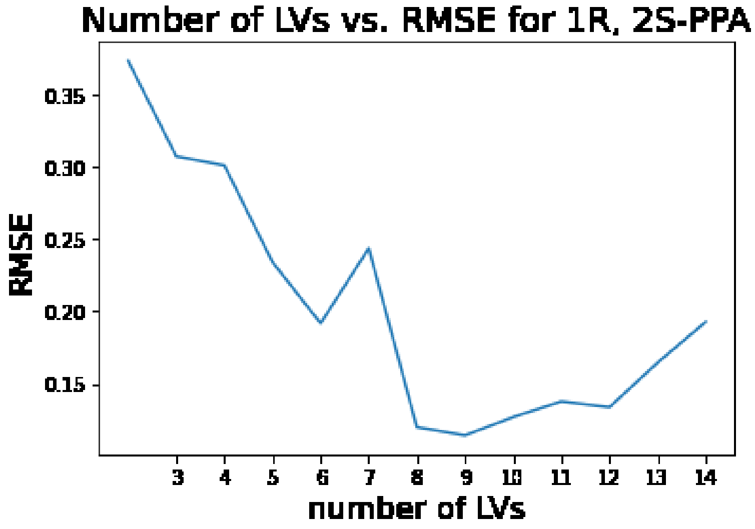

Figure 217. Number of latent variables versus averaged root-mean-square (RMSE) error in training sets for (1*S*, 2*R*)-**PPA** using leave-one-out cross-validation for LASSO + PCR

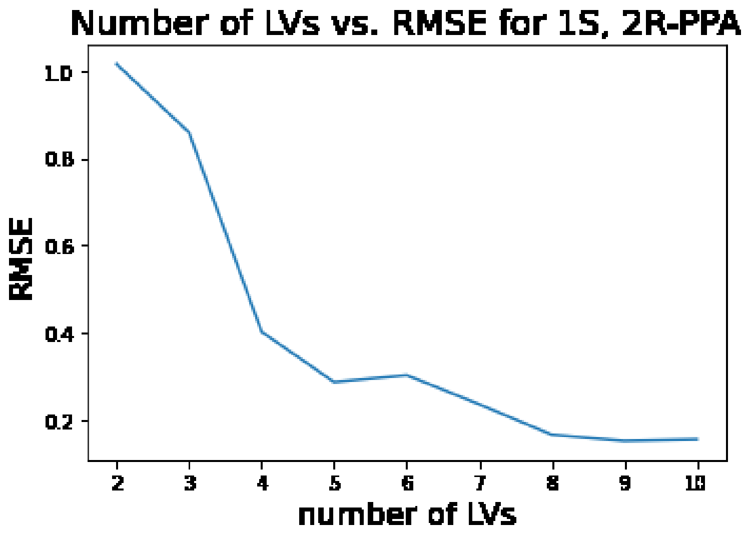

Figure 218. Number of latent variables versus averaged root-mean-square (RMSE) error in training sets for (*R*)-**PGL** using leave-one-out cross-validation for LASSO + PCR

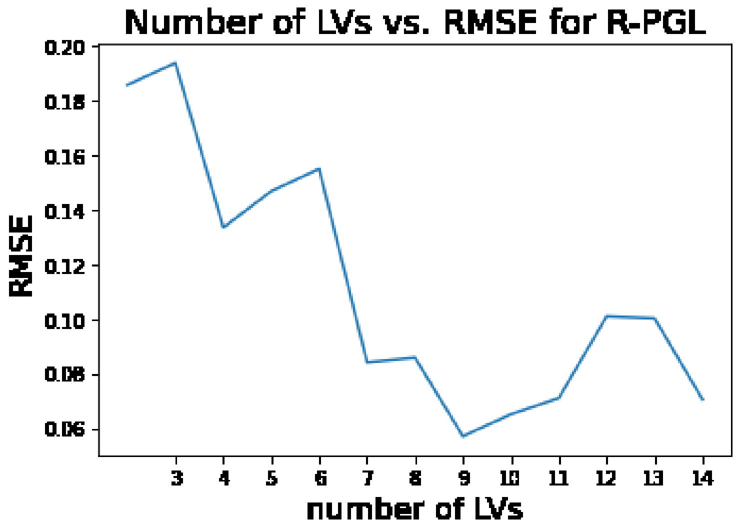

Figure 219. Number of latent variables versus averaged root-mean-square (RMSE) error in training sets for (*S*)-**PGL** using leave-one-out cross-validation for LASSO + PCR

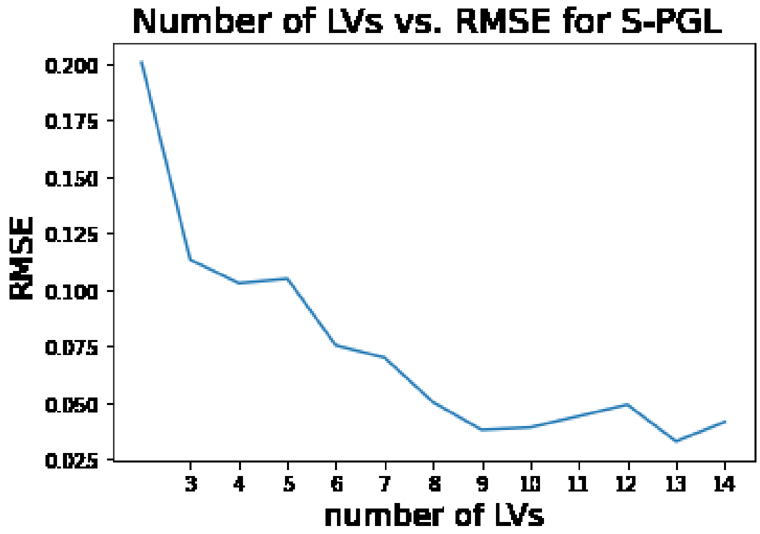

Figure 220. Number of latent variables versus averaged root-mean-square (RMSE) error in training sets for (*R*)-**PEA** using leave-one-out cross-validation for LASSO + PLS

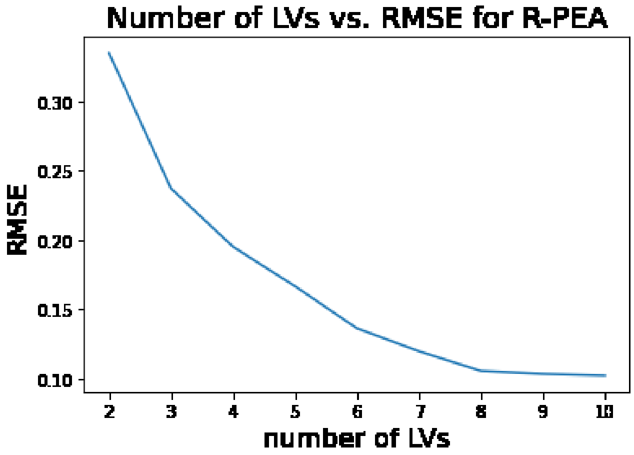

Figure 221. Number of latent variables versus averaged root-mean-square (RMSE) error in training sets for (*S*)-**PEA** using leave-one-out cross-validation for LASSO + PLS

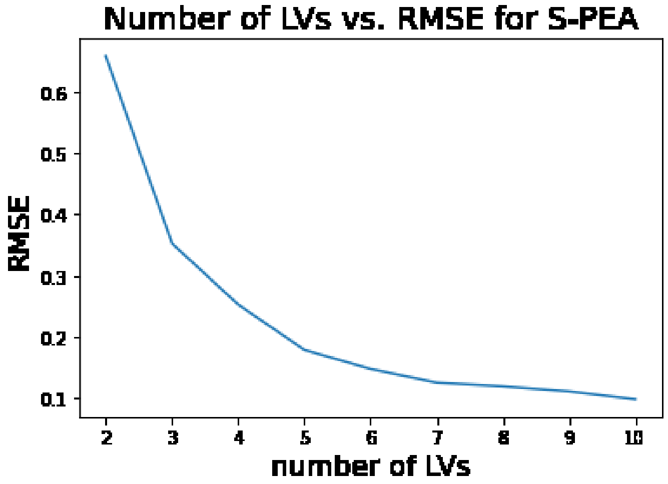

Figure 222. Number of latent variables versus averaged root-mean-square (RMSE) error in training sets for (*R*)-**PMP** using leave-one-out cross-validation for LASSO + PLS

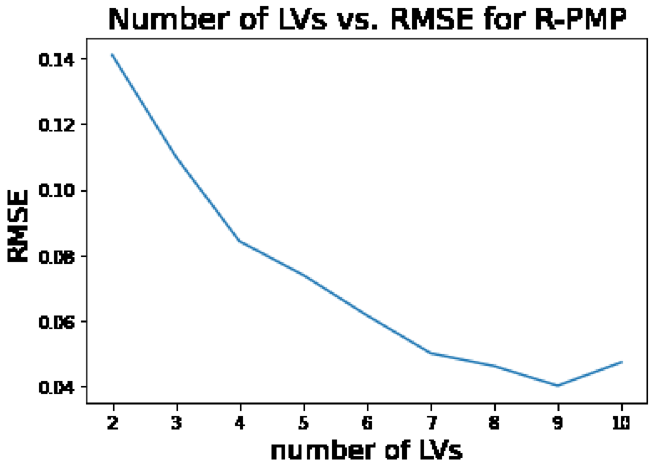

Figure 223. Number of latent variables versus averaged root-mean-square (RMSE) error in training sets for (*S*)-**PMP** using leave-one-out cross-validation for LASSO + PLS

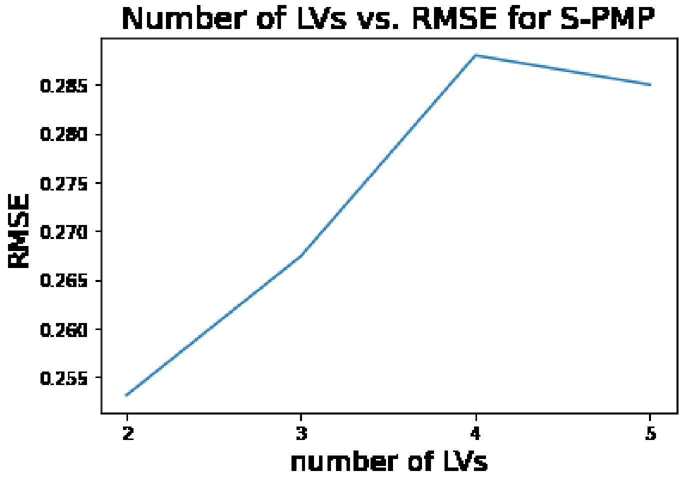

Figure 224. Number of latent variables versus averaged root-mean-square (RMSE) error in training sets for (1*R*, 2*S*)-**PPA** using leave-one-out cross-validation for LASSO + PLS

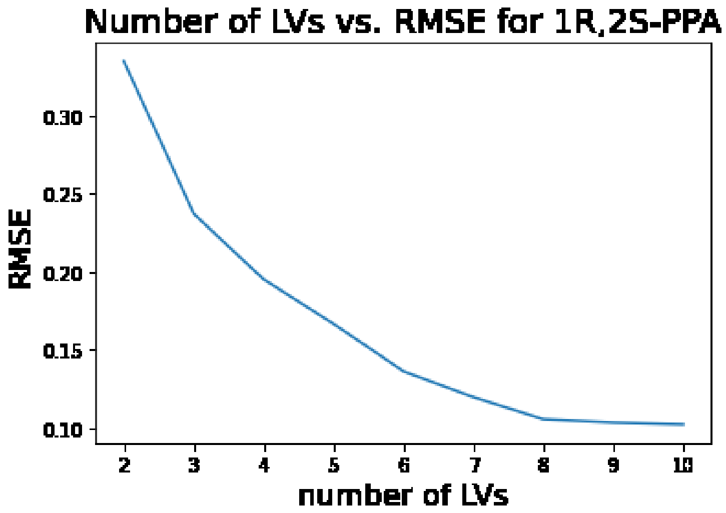

Figure 225. Number of latent variables versus averaged root-mean-square (RMSE) error in training sets for (1*S*, 2*R*)-**PPA** using leave-one-out cross-validation for LASSO + PLS

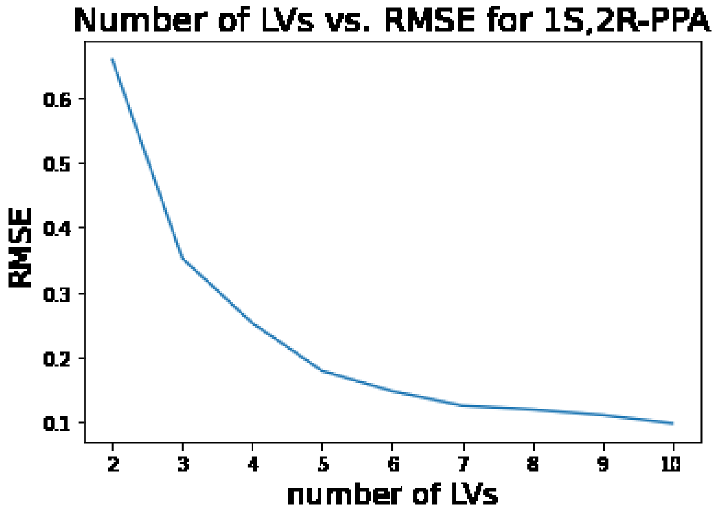

Figure 226. Number of latent variables versus averaged root-mean-square (RMSE) error in training sets for (*R*)-**PGL** using leave-one-out cross-validation for LASSO + PLS

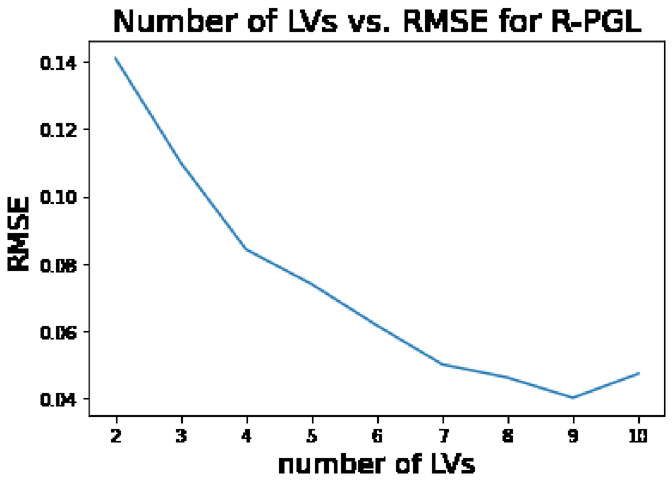

Figure 227. Number of latent variables versus averaged root-mean-square (RMSE) error in training sets for (*S*)-**PGL** using leave-one-out cross-validation for LASSO + PLS

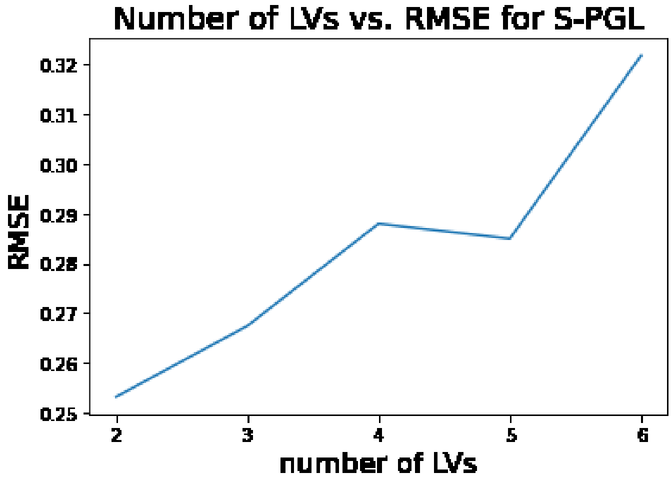

Supplementary Figure 228. Lambda value versus 5-fold cross-validation (CV) score for (*R*)-**PEA**

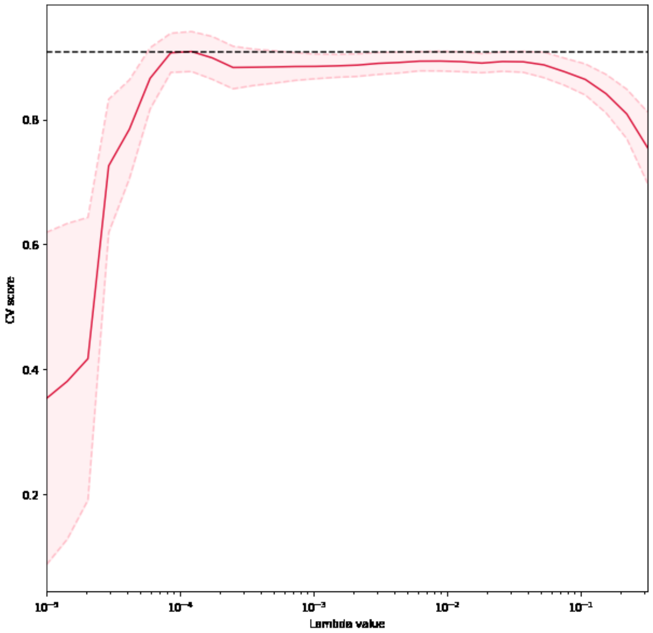

Supplementary Figure 229. Lambda value versus 5-fold cross-validation (CV) score for (*S*)-**PEA**

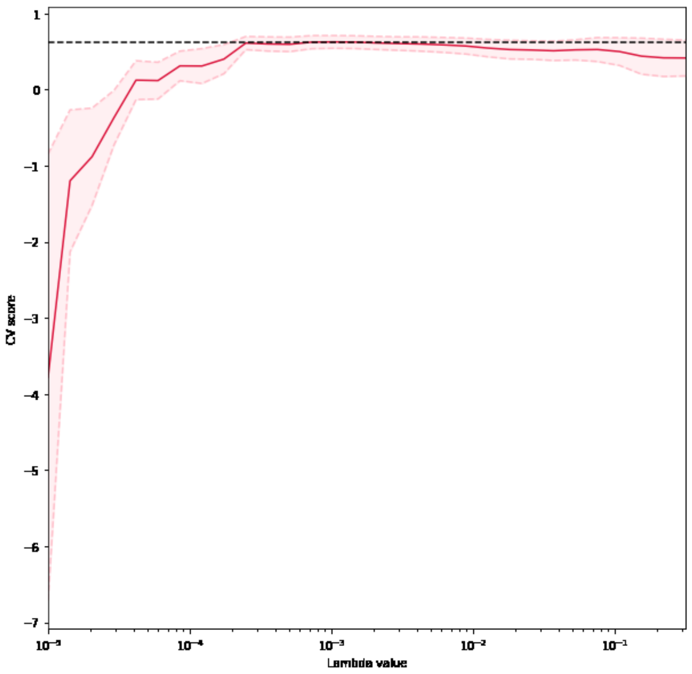

Supplementary Figure 230. Lambda value versus 5-fold cross-validation (CV) score for (*R*)-**PMP**

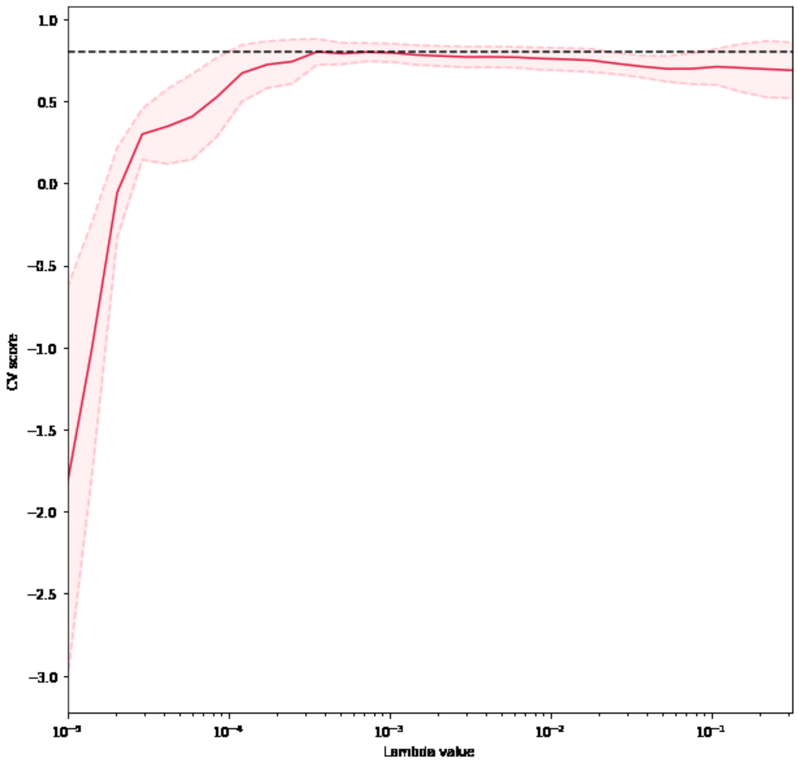

Supplementary Figure 231. Lambda value versus 5-fold cross-validation (CV) score for (*S*)-**PMP**

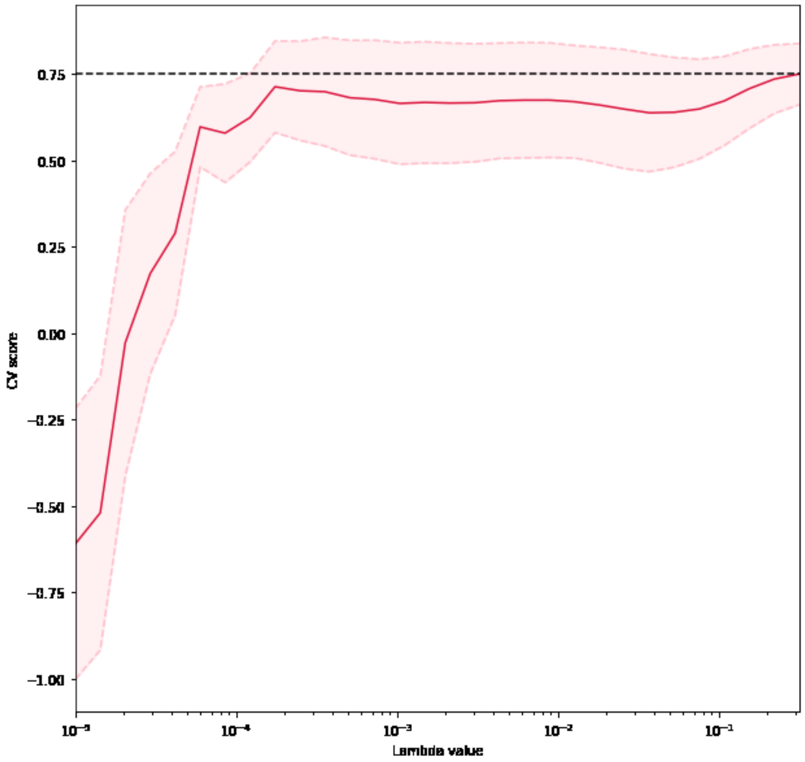

Supplementary Figure 232. Lambda value versus 5-fold cross-validation (CV) score for (1*R*, 2*S*)-**PPA**

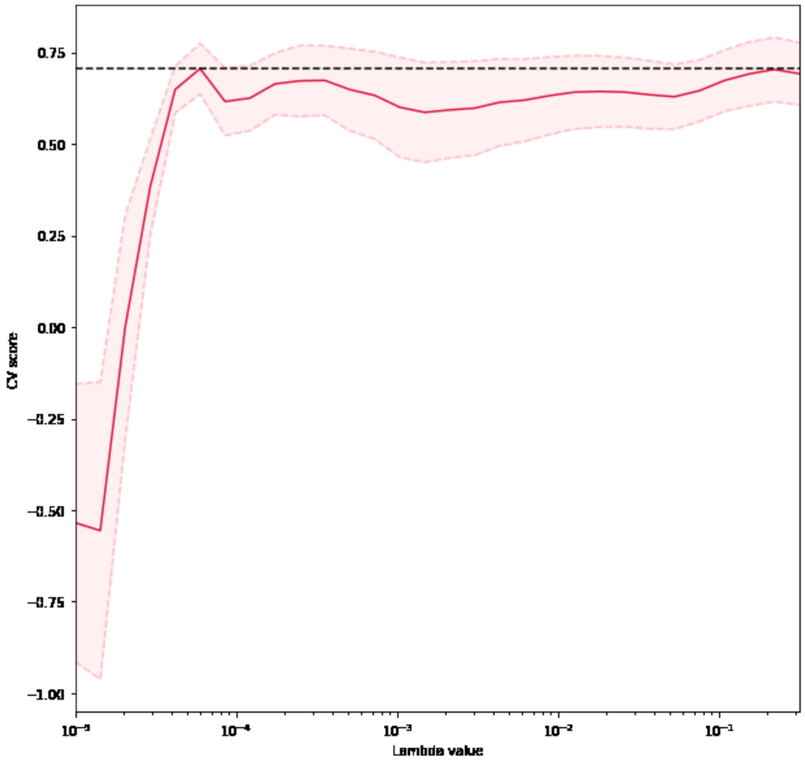

Supplementary Figure 233. Lambda value versus 5-fold cross-validation (CV) score for (1*S*, 2*R*)-**PPA**

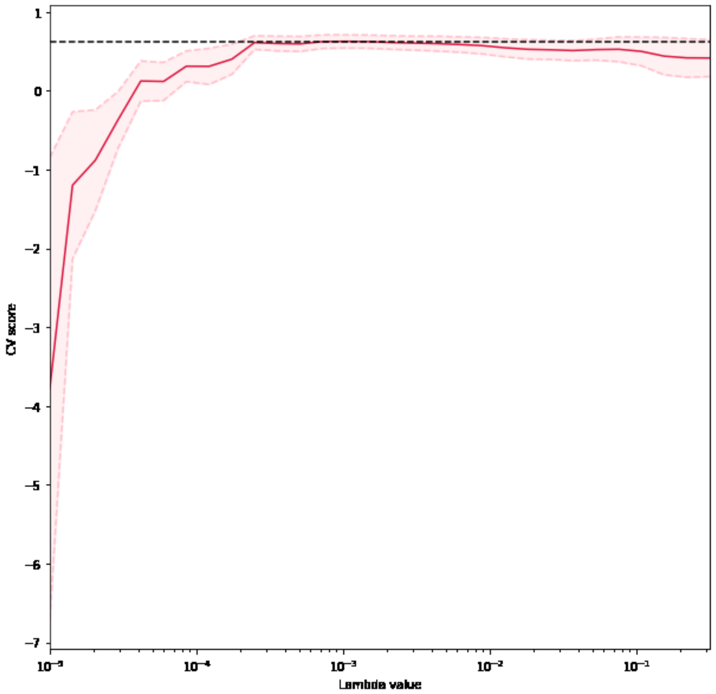

Supplementary Figure 234. Lambda value versus 5-fold cross-validation (CV) score for (*R*)-**PGL**

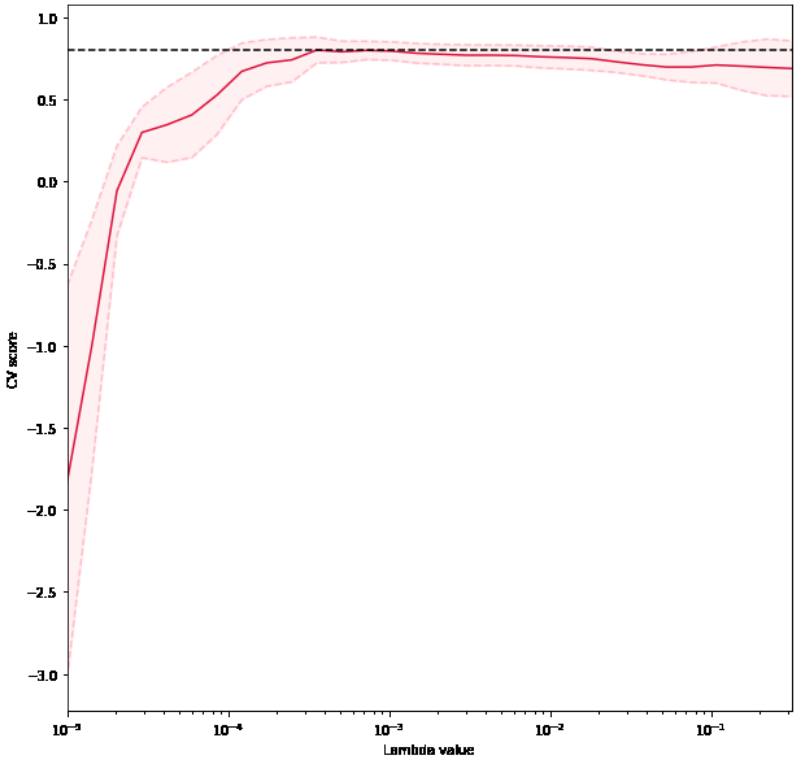

Supplementary Figure 235. Lambda value versus 5-fold cross-validation (CV) score for (*S*)-**PGL**

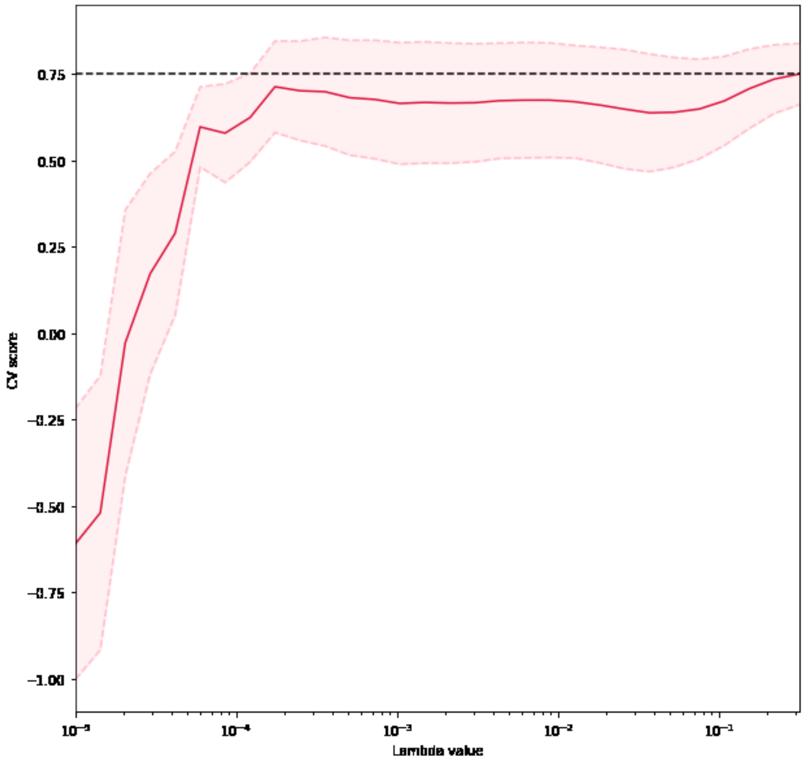

Supplementary Tables

Supplementary Table 1. Actual versus predicted enantiomeric ratios of **PEA** and **PMP** using traditional sensing methodology

Entry	Actual PMP (*R*/*S*)	Actual PEA (*R*/*S*)	Predicted PMP (*R*/*S*)	Predicted PEA (*R*/*S*)
1	100.0: 0.0	100.0 : 0.0	102.1 : 0.0	99.7 : 0.3
2	0.0 : 100.0	100.0 : 0.0	1.8 : 98.2	100.4: 0.0
3	70.0 : 30.0	100.0 : 0.0	70.4 : 29.6	97.3 : 2.7
4	100.0 : 0.0	70.0 : 30.0	98.9 : 1.1	66.5 : 33.4
5	20.0 : 80.0	80.0 : 20.0	20.4 : 79.6	83.8 : 16.2
6	30.0 : 70.0	0.0 : 100.0	29.9 : 70.1	0.0 : 100.0
7	60.0 : 40.0	60.0 : 40.0	61.2 : 38.8	62.3 : 37.5
8	45.0 : 55.0	45.0 : 55.0	41.8 : 58.2	45.7 : 54.3
9	70.0 : 30.0	20.0 : 80.0	67.6 : 32.4	22.3 : 77.6
10	90.0 : 10.0	40.0 : 60.0	90.8 : 9.2	37.5 : 62.4

Supplementary Table 2. Explained variance ratio in the first four Principal Components for CD and UV sensing data. See section 4.2. for more information on explained variance ratio.

	**Explained Variance Ratio**	
**Principal Component**	**CD data**	**UV data**
PC1	0.690	0.660
PC2	0.285	0.286
PC3	0.015	0.054
PC4	0.002	0.0003

Supplementary Table 3. R^2 and RMSE results for all algorithms (**PEA**)

	(*R*)-PEA				(*S*)-PEA			
**Algorithm**	**Train Set R^2**	**Train Set RMSE**	**Test Set R^2**	**Test Set RMSE**	**Train Set R^2**	**Train Set RMSE**	**Test Set R^2**	**Test Set RMSE**
MBPCA + OLS	0.98	0.30	0.98	0.24	0.85	0.47	0.90	0.34
LASSO + PCR	0.99	0.19	0.96	0.34	0.98	0.17	0.86	0.42
MBPLS	0.98	0.25	0.97	0.31	0.92	0.35	0.91	0.29
LASSO + PLS	0.99	0.13	0.96	0.33	0.98	0.16	0.90	0.36

Supplementary Table 4. R^2 and RMSE results for all algorithms (**PMP**)

	(*R*)-PMP				(*S*)-PMP			
**Algorithm**	**Train Set R^2**	**Train Set RMSE**	**Test Set R^2**	**Test Set RMSE**	**Train Set R^2**	**Train Set RMSE**	**Test Set R^2**	**Test Set RMSE**
MBPCA + OLS	0.98	0.26	0.96	0.26	0.99	0.15	0.99	0.20
LASSO + PCR	0.99	0.08	0.98	0.20	0.99	0.09	0.97	0.28
MBPLS	0.99	0.18	0.96	0.26	0.99	0.17	0.98	0.27
LASSO + PLS	0.99	0.08	0.98	0.20	0.99	0.09	0.97	0.28

Supplementary Table 5. R^2 and RMSE results for all algorithms (**PPA**)

	**(1*R*, 2*S*)-PPA**				**(1*S*, 2*R*)-PPA**			
**Algorithm**	**Train Set R^2**	**Train Set RMSE**	**Test Set R^2**	**Test Set RMSE**	**Train Set R^2**	**Train Set RMSE**	**Test Set R^2**	**Test Set RMSE**
MBPCA + OLS	0.98	0.30	0.98	0.24	0.85	0.47	0.90	0.34
LASSO + PCR	0.99	0.13	0.96	0.33	0.98	0.17	0.86	0.41
MBPLS	0.98	0.30	0.97	0.31	0.91	0.35	0.93	0.29
LASSO + PLS	0.99	0.13	0.96	0.33	0.98	0.16	0.90	0.36

Supplementary Table 6. R^2 and RMSE results for all algorithms (**PGL**)

	**(*R*)-PGL**				**(*S*)-PGL**			
**Algorithm**	**Train Set R^2**	**Train Set RMSE**	**Test Set R^2**	**Test Set RMSE**	**Train Set R^2**	**Train Set RMSE**	**Test Set R^2**	**Test Set RMSE**
MBPCA + OLS	0.98	0.26	0.96	0.26	0.99	0.15	0.99	0.20
LASSO + PCR	0.99	0.08	0.98	0.19	0.99	0.09	0.97	0.29
MBPLS	0.99	0.18	0.96	0.25	0.99	0.17	0.98	0.27
LASSO + PLS	0.99	0.08	0.98	0.19	0.99	0.09	0.97	0.28

Supplementary Table 7. Absolute errors for all algorithms (**PEA** and **PMP**)

	**(*R*)-PEA**		**(*S*)-PEA**		**(*R*)-PMP**		**(*S*)-PMP**	
**Algorithm**	**Train Set**	**Test Set**	**Train Set**	**Test Set**	**Train Set**	**Test Set**	**Train Set**	**Test Set**
MBPCA + OLS	0.25	0.22	0.35	0.28	0.19	0.21	0.13	0.14
LASSO + PCR	0.21	0.24	0.25	0.2	0.14	0.2	0.12	0.14
MBPLS	0.16	0.39	0.13	0.34	0.06	0.15	0.07	0.25
LASSO + PLS	0.10	0.26	0.12	0.29	0.06	0.16	0.08	0.25

Supplementary Table 8. Absolute errors for all algorithms (**PPA** and **PGL**)

	**(1*R*, 2S)-PPA**		**(1*S*, 2*R*)-PPA**		**(*R*)-PGL**		**(*S*)-PGL**		**Average**	
**Algorithm**	**Train Set**	**Test Set**	**Train Set**	**Test Set**	**Train Set**	**Test Set**	**Train Set**	**Test Set**	**Train Set**	**Test Set**
MBPCA + OLS	0.25	0.21	0.35	0.28	0.19	0.21	0.12	0.14	0.23	0.21
LASSO + PCR	0.21	0.24	0.26	0.25	0.14	0.2	0.14	0.24	0.18	0.21
MBPLS	0.1	0.25	0.13	0.34	0.06	0.15	0.07	0.25	0.10	0.27
LASSO + PLS	0.1	0.26	0.12	0.29	0.06	0.16	0.08	0.25	0.09	0.24

Supplementary Table 9. Maximum absolute error (mM) for each algorithm across all samples

**Algorithm**	**Maximum Absolute Error (mM)**	**Sample**
MBPCA + OLS	1.09	Training set 11, (*S*)-**PEA**
LASSO + PCR	0.82	Training set 11, (*S*)-**PEA**
MBPLS	0.82	Training set 11, (*S*)-**PEA**
LASSO + PLS	0.70	Test set 2 (*R*)-**PEA** and (1*R*,2*S*)-**PPA**

Supplementary Table 10. Maximum absolute error (mM) for each algorithm in the test sets (**PEA** and **PMP**)

	**(*R*)-PEA**		**(*S*)-PEA**		**(*R*)-PMP**		**(*S*)-PMP**	
**Algorithm**	**Max absolute error in test set**	**Sample**	**Max absolute error in test set**	**Sample**	**Max absolute error in test set**	**Sample**	**Max absolute error in test set**	**Sample**
MBPCA + OLS	0.34	Test set 5	0.68	Test set 3	0.51	Test set 3	0.42	Test set 3
LASSO + PCR	0.57	Test set 2	0.66	Test set 4	0.33	Test set 5	0.44	Test set 2
MBPLS	0.56	Test set 2	0.49	Test set 4	0.54	Test set 6	0.42	Test set 3
LASSO + PLS	0.70	Test set 2	0.58	Test set 3	0.33	Test set 5	0.42	Test set 6

Supplementary Table 11. Average relative error for each algorithm in both training and test set (**PEA** and **PMP**)

	**(*R*)-PEA**		**(*S*)-PEA**		**(*R*)-PMP**		**(*S*)-PMP**	
**Algorithm**	**Train Set**	**Test Set**	**Train Set**	**Test Set**	**Train Set**	**Test Set**	**Train Set**	**Test Set**
MBPCA + OLS	10.10	10.78	23.55	22.42	8.38	9.42	8.73	12.05
MBPLS	8.88	10.74	16.35	13.11	7.14	7.19	6.69	12.20
LASSO + PCR	9.85	17.73	10.13	20.63	2.47	7.01	5.26	31.91
LASSO + PLS	7.06	8.81	8.90	18.11	2.22	7.27	5.49	31.10

Supplementary Table 12. Maximum absolute relative error for each algorithm in test set (**PPA** and **PGL**)

	**(1*R, 2S*)-PPA**		**(1*S*, 2*R*)-PPA**		**(*R*)-PGL**		**(*S*)-PGL**	
**Algorithm**	**Max absolute error in test set**	**Sample**	**Max absolute error in test set**	**Sample**	**Max absolute error in test set**	**Sample**	**Max absolute error in test set**	**Sample**
MBPCA + OLS	0.34	Test set 5	0.68	Test set 3	0.51	Test set 3	0.42	Test set 3
LASSO + PCR	0.66	Test set 2	0.66	Test set 4	0.33	Test set 5	0.44	Test set 2
MBPLS	0.56	Test set 2	0.47	Test set 3	0.51	Test set 6	0.40	Test set 5 and 6
LASSO + PLS	0.7	Test set 2	0.58	Test set 3	0.33	Test set 5	0.42	Test set 6

Supplementary Table 13. Average relative errors for all algorithms (**PPA** and **PGL**) and average relative error across all analytes

	**(1*R*, 2S)-PPA**		**(1*S*, 2*R*)-PPA**		**(*R*)-PGL**		**(*S*)-PGL**		**Average**	
**Algorithm**	**Train Set**	**Test Set**	**Train Set**	**Test Set**	**Train Set**	**Test Set**	**Train Set**	**Test Set**	**Train Set**	**Test Set**
MBPCA + OLS	10.10	10.75	24.46	22.41	8.38	9.42	8.73	12.05	12.80	12.47
MBPLS	8.89	10.74	16.78	17.77	6.41	7.81	8.46	30.40	9.95	13.75
LASSO + PCR	6.68	7.72	11.16	20.63	2.37	7.01	9.46	31.91	7.17	18.07
LASSO + PLS	7.06	8.81	8.90	18.11	2.22	7.27	5.49	31.07	5.92	16.32

Supplementary Table 14. Block loading for CD data in DCE

	PC1	PC2	PC3	PC4
434.5	0.051106	0.060659	0.043556	-0.03481
434	0.051367	0.060663	0.042973	-0.03008
433.5	0.051646	0.060503	0.041566	-0.02628
433	0.05194	0.060076	0.039185	-0.02394
432.5	0.052219	0.059392	0.035969	-0.02318
432	0.052443	0.058558	0.032323	-0.02363
431.5	0.052586	0.057726	0.028734	-0.02463
431	0.05264	0.057028	0.025571	-0.02556
430.5	0.052627	0.056548	0.023009	-0.02605
430	0.052586	0.056304	0.021056	-0.02601
429.5	0.05256	0.056262	0.019645	-0.0256
429	0.052582	0.056335	0.018707	-0.02505
428.5	0.052661	0.056402	0.018182	-0.02456
428	0.052794	0.056337	0.01801	-0.02423
427.5	0.052971	0.056034	0.018108	-0.02409
427	0.053186	0.055426	0.018369	-0.02413
426.5	0.053436	0.054496	0.018656	-0.02441
426	0.053718	0.053278	0.018809	-0.02503
425.5	0.054031	0.051861	0.018678	-0.02608
425	0.054366	0.050377	0.018194	-0.02758
424.5	0.054709	0.048965	0.017442	-0.02934
424	0.055042	0.04774	0.016679	-0.03104
423.5	0.055347	0.046758	0.016271	-0.0323
423	0.055612	0.046017	0.01656	-0.03283
422.5	0.055835	0.045464	0.017749	-0.03251
422	0.056021	0.045021	0.019839	-0.0314
421.5	0.056179	0.044614	0.022621	-0.02972
421	0.056312	0.044193	0.025738	-0.02773
420.5	0.056422	0.043756	0.02877	-0.02568
420	0.056509	0.043341	0.031349	-0.02373
419.5	0.056572	0.04301	0.033262	-0.02194
419	0.056614	0.04281	0.034497	-0.02029
418.5	0.056636	0.042763	0.035252	-0.01871
418	0.056637	0.042861	0.035873	-0.01713
417.5	0.056616	0.043087	0.036762	-0.01544
417	0.056567	0.043422	0.038255	-0.0135
416.5	0.056484	0.043847	0.040502	-0.01124
416	0.056365	0.044343	0.043371	-0.00866
415.5	0.056216	0.044885	0.046444	-0.00591
415	0.056056	0.045443	0.049117	-0.00325
414.5	0.055914	0.045987	0.050849	-0.00091

414	0.055807	0.046491	0.051432	0.000981
413.5	0.055736	0.046941	0.051118	0.002494
413	0.055682	0.047333	0.050483	0.003791
412.5	0.055624	0.047658	0.050142	0.00501
412	0.055557	0.04789	0.050517	0.006157
411.5	0.055484	0.047982	0.051752	0.007115
411	0.055421	0.047884	0.053758	0.007723
410.5	0.055376	0.047569	0.056291	0.007885
410	0.055356	0.047055	0.05902	0.007619
409.5	0.055359	0.046407	0.061575	0.00702
409	0.055386	0.045713	0.063603	0.006185
408.5	0.055438	0.045061	0.064847	0.005151
408	0.05551	0.044511	0.065238	0.003892
407.5	0.055594	0.044083	0.064951	0.00236
407	0.055679	0.043758	0.064349	0.000534
406.5	0.055756	0.043496	0.063837	-0.00156
406	0.055827	0.043253	0.063676	-0.00389
405.5	0.055903	0.042993	0.063872	-0.00637
405	0.055996	0.042701	0.064187	-0.00895
404.5	0.056114	0.04239	0.064284	-0.01152
404	0.056254	0.042102	0.063917	-0.01395
403.5	0.056398	0.0419	0.063044	-0.0161
403	0.056523	0.041855	0.06181	-0.01787
402.5	0.056611	0.042026	0.060418	-0.01922
402	0.05665	0.04244	0.05902	-0.02023
401.5	0.056639	0.043082	0.057694	-0.02098
401	0.056588	0.043893	0.056497	-0.02155
400.5	0.05651	0.044788	0.055535	-0.02189
400	0.056417	0.045672	0.054985	-0.0219
399.5	0.056318	0.04646	0.055038	-0.02143
399	0.056219	0.04709	0.055805	-0.02041
398.5	0.056125	0.047526	0.057245	-0.01887
398	0.056043	0.047762	0.05916	-0.01703
397.5	0.055979	0.047823	0.061273	-0.01521
397	0.055935	0.047757	0.063343	-0.01371
396.5	0.055908	0.047624	0.06524	-0.01276
396	0.05589	0.04748	0.06694	-0.01236
395.5	0.055871	0.047362	0.06846	-0.01236
395	0.055851	0.047274	0.069792	-0.01251
394.5	0.055833	0.047191	0.070882	-0.01254
394	0.055829	0.047062	0.07166	-0.01227
393.5	0.055851	0.046831	0.072078	-0.0116
393	0.055907	0.046464	0.072123	-0.01057

392.5	0.055997	0.045957	0.071795	-0.00934
392	0.056119	0.045346	0.071071	-0.00817
391.5	0.056265	0.044685	0.069893	-0.00732
391	0.056425	0.044033	0.068197	-0.007
390.5	0.056589	0.043438	0.06597	-0.00725
390	0.056746	0.042936	0.063288	-0.00795
389.5	0.056886	0.042551	0.060316	-0.00887
389	0.057002	0.042294	0.057259	-0.00983
388.5	0.057088	0.042163	0.054307	-0.01071
388	0.057144	0.042137	0.051596	-0.01152
387.5	0.057176	0.042171	0.049206	-0.01232
387	0.057192	0.042208	0.047179	-0.01318
386.5	0.057204	0.042189	0.045535	-0.01404
386	0.057223	0.04208	0.044289	-0.01478
385.5	0.057252	0.041878	0.043446	-0.01528
385	0.057291	0.041602	0.043006	-0.01544
384.5	0.057332	0.041275	0.04295	-0.01523
384	0.057366	0.040912	0.043242	-0.01469
383.5	0.057388	0.040513	0.043814	-0.01385
383	0.057398	0.040068	0.044576	-0.01275
382.5	0.057398	0.039573	0.045428	-0.01145
382	0.057398	0.039027	0.046304	-0.01007
381.5	0.057405	0.038432	0.047196	-0.00876
381	0.057425	0.037776	0.048169	-0.00766
380.5	0.057459	0.037037	0.049339	-0.00676
380	0.057502	0.036178	0.050819	-0.00587
379.5	0.057545	0.035167	0.05267	-0.00472
379	0.057579	0.033982	0.054867	-0.00305
378.5	0.0576	0.032621	0.057282	-0.00079
378	0.057613	0.031097	0.059697	0.001937
377.5	0.057629	0.029433	0.061843	0.004872
377	0.057664	0.02766	0.063445	0.007766
376.5	0.057731	0.025807	0.064302	0.010466
376	0.057834	0.023896	0.064355	0.012921
375.5	0.057968	0.021932	0.063728	0.015108
375	0.058117	0.019899	0.062709	0.016982
374.5	0.058261	0.017773	0.061662	0.018477
374	0.058381	0.015537	0.060915	0.019552
373.5	0.058465	0.0132	0.060666	0.020211
373	0.058507	0.010787	0.060941	0.020483
372.5	0.05851	0.008327	0.061606	0.02039
372	0.058479	0.005827	0.062428	0.019973
371.5	0.058424	0.003259	0.06316	0.019359

371	0.058353	0.000561	0.063611	0.018806
370.5	0.058273	-0.00232	0.063684	0.018647
370	0.058189	-0.00541	0.063374	0.019145
369.5	0.058104	-0.00865	0.062745	0.020337
369	0.058013	-0.01196	0.061907	0.02197
368.5	0.057917	-0.01519	0.060996	0.023608
368	0.057811	-0.01826	0.060141	0.024857
367.5	0.057695	-0.02109	0.05941	0.025588
367	0.057566	-0.02371	0.058762	0.026023
366.5	0.057419	-0.02614	0.058047	0.026619
366	0.057249	-0.02848	0.057048	0.027843
365.5	0.057047	-0.03079	0.055575	0.029978
365	0.056798	-0.03315	0.053544	0.033061
364.5	0.056487	-0.03563	0.051003	0.036981
364	0.056096	-0.03826	0.048095	0.041615
363.5	0.055616	-0.04099	0.044997	0.046896
363	0.055045	-0.04375	0.041888	0.052728
362.5	0.054393	-0.04645	0.038947	0.058869
362	0.053681	-0.04904	0.036351	0.064876
361.5	0.052932	-0.05151	0.034244	0.070205
361	0.05217	-0.05388	0.032689	0.074397
360.5	0.051413	-0.05621	0.031642	0.077236
360	0.050671	-0.05857	0.030983	0.078782
359.5	0.049943	-0.06102	0.030578	0.079262
359	0.049218	-0.06363	0.030345	0.078907
358.5	0.048484	-0.06639	0.030279	0.077822
358	0.047735	-0.06924	0.030435	0.075983
357.5	0.04698	-0.07205	0.030873	0.073353
357	0.04624	-0.07468	0.031595	0.070037
356.5	0.045541	-0.07704	0.032521	0.066379
356	0.044894	-0.07909	0.033527	0.062898
355.5	0.044293	-0.08091	0.034526	0.060099
355	0.043721	-0.08259	0.035515	0.058267
354.5	0.043162	-0.08423	0.036541	0.057367
354	0.042622	-0.08582	0.037602	0.057122
353.5	0.042131	-0.08731	0.038553	0.057191
353	0.041725	-0.0886	0.039123	0.057354
352.5	0.041428	-0.08959	0.039034	0.057596
352	0.041231	-0.09026	0.038157	0.058073
351.5	0.041089	-0.09064	0.036589	0.059032
351	0.040938	-0.09084	0.034622	0.060733
350.5	0.040712	-0.09097	0.032618	0.063381
350	0.040362	-0.09113	0.030871	0.067066

349.5	0.039874	-0.09142	0.029509	0.07172
349	0.03926	-0.09187	0.028491	0.077097
348.5	0.038559	-0.09247	0.02769	0.082802
348	0.037819	-0.09319	0.027001	0.088358
347.5	0.037085	-0.094	0.026425	0.093279
347	0.036396	-0.09483	0.026075	0.09715
346.5	0.03577	-0.09563	0.026129	0.099695
346	0.035204	-0.09636	0.026759	0.100831
345.5	0.034653	-0.09702	0.028096	0.100709
345	0.034031	-0.09761	0.030233	0.099704
344.5	0.033227	-0.09819	0.033243	0.098347
344	0.032146	-0.09877	0.037163	0.097151
343.5	0.030773	-0.09933	0.04192	0.096378
343	0.029232	-0.09976	0.047221	0.095846
342.5	0.027786	-0.09991	0.052498	0.09493
342	0.026755	-0.09967	0.056995	0.092822
341.5	0.026366	-0.09897	0.060001	0.088935
341	0.026628	-0.09786	0.061138	0.083223
340.5	0.027309	-0.09651	0.060577	0.076285
340	0.028025	-0.09523	0.059034	0.069173
339.5	0.028401	-0.09439	0.057491	0.063015
339	0.028186	-0.09427	0.056743	0.058656
338.5	0.027296	-0.09491	0.057033	0.05651
338	0.025802	-0.09611	0.057962	0.056602
337.5	0.023889	-0.09748	0.05871	0.058659
337	0.021805	-0.09856	0.058448	0.062145
336.5	0.019808	-0.09894	0.056685	0.066287
336	0.018117	-0.09829	0.05337	0.070149
335.5	0.016879	-0.09646	0.048725	0.072828
335	0.016122	-0.09348	0.043036	0.073734
334.5	0.015705	-0.08957	0.03659	0.072841
334	0.015314	-0.08514	0.029795	0.070742
333.5	0.014535	-0.08065	0.023316	0.068367
333	0.01302	-0.07635	0.018045	0.066425
332.5	0.010654	-0.07216	0.014878	0.064912
332	0.007643	-0.06765	0.014437	0.063018
331.5	0.004452	-0.06225	0.01689	0.05948
331	0.001619	-0.05542	0.021944	0.053069
330.5	-0.00046	-0.04681	0.028978	0.042839
330	-0.00166	-0.03629	0.037166	0.028143
329.5	-0.00214	-0.02405	0.045523	0.008666
329	-0.00226	-0.01068	0.052939	-0.01538
328.5	-0.00248	0.002882	0.058339	-0.04307

328	-0.00317	0.015652	0.060962	-0.07291
327.5	-0.00457	0.02692	0.060587	-0.10319
327	-0.00677	0.036447	0.057575	-0.13232
326.5	-0.00977	0.04442	0.05271	-0.15891
326	-0.01358	0.051233	0.046916	-0.18153
325.5	-0.01807	0.057199	0.040981	-0.19869
325	-0.02301	0.062339	0.035384	-0.209
324.5	-0.02801	0.066368	0.030215	-0.21176
324	-0.03268	0.068904	0.025178	-0.20742
323.5	-0.03677	0.069714	0.019669	-0.19761
323	-0.0402	0.068807	0.013003	-0.18442
322.5	-0.04301	0.066388	0.004744	-0.16972
322	-0.04528	0.062781	-0.00497	-0.15479
321.5	-0.04711	0.058402	-0.01537	-0.14029
321	-0.04861	0.05371	-0.02524	-0.12638
320.5	-0.04987	0.049133	-0.03348	-0.11289
320	-0.05101	0.044965	-0.03944	-0.09953
319.5	-0.05208	0.041316	-0.04308	-0.08609
319	-0.05312	0.03813	-0.04488	-0.07257
318.5	-0.05415	0.035256	-0.04553	-0.05917
318	-0.05512	0.032522	-0.04567	-0.04622
317.5	-0.05601	0.029788	-0.04572	-0.03399
317	-0.05679	0.026968	-0.04582	-0.02264
316.5	-0.05743	0.02405	-0.04593	-0.01218
316	-0.05793	0.021097	-0.04587	-0.00261
315.5	-0.0583	0.018243	-0.04543	0.006007
315	-0.05857	0.015666	-0.04445	0.013526
314.5	-0.05875	0.013531	-0.04287	0.01981
314	-0.05888	0.011941	-0.04083	0.02481
313.5	-0.05897	0.010903	-0.03865	0.028611
313	-0.05904	0.010328	-0.03677	0.031403
312.5	-0.0591	0.010056	-0.03555	0.033419
312	-0.05915	0.009896	-0.03522	0.034852
311.5	-0.0592	0.00968	-0.0357	0.035807
311	-0.05925	0.009296	-0.03674	0.036282
310.5	-0.05931	0.008714	-0.03796	0.036186
310	-0.05937	0.007968	-0.0391	0.035391
309.5	-0.05944	0.007131	-0.04	0.033788
309	-0.05952	0.006276	-0.04069	0.031341
308.5	-0.05959	0.005454	-0.04131	0.028129
308	-0.05966	0.004684	-0.04196	0.024333
307.5	-0.05971	0.003955	-0.04271	0.020193
307	-0.05973	0.003225	-0.04358	0.015953

306.5	-0.05974	0.002434	-0.04454	0.011796
306	-0.05974	0.001521	-0.04558	0.00782
305.5	-0.05972	0.000439	-0.04669	0.00404
305	-0.05969	-0.00082	-0.04786	0.000411
304.5	-0.05965	-0.00223	-0.04906	-0.00314
304	-0.0596	-0.00373	-0.05024	-0.00665
303.5	-0.05955	-0.00524	-0.05136	-0.01012
303	-0.05949	-0.00671	-0.05236	-0.01348
302.5	-0.05943	-0.00809	-0.05324	-0.01656
302	-0.05937	-0.00937	-0.05396	-0.01918
301.5	-0.05931	-0.01054	-0.05452	-0.02114
301	-0.05926	-0.01159	-0.05489	-0.0223
300.5	-0.05921	-0.01252	-0.05501	-0.02264
300	-0.05917	-0.01332	-0.05486	-0.02228
299.5	-0.05915	-0.01398	-0.05445	-0.02145
299	-0.05913	-0.0145	-0.05388	-0.02044
298.5	-0.05912	-0.01489	-0.05333	-0.01952
298	-0.0591	-0.01517	-0.053	-0.01888
297.5	-0.05908	-0.01535	-0.05303	-0.0186
297	-0.05906	-0.01546	-0.05346	-0.01858
296.5	-0.05903	-0.01551	-0.05414	-0.01865
296	-0.05902	-0.01554	-0.05482	-0.01861
295.5	-0.05901	-0.01557	-0.05522	-0.01826
295	-0.05901	-0.01562	-0.05511	-0.01754
294.5	-0.05902	-0.01569	-0.05446	-0.01645
294	-0.05903	-0.01577	-0.05334	-0.01514
293.5	-0.05904	-0.01583	-0.05201	-0.01378
293	-0.05905	-0.01586	-0.05071	-0.01254
292.5	-0.05905	-0.01582	-0.04968	-0.01155
292	-0.05904	-0.0157	-0.04901	-0.01084
291.5	-0.05903	-0.01548	-0.04873	-0.01042
291	-0.05903	-0.01514	-0.04874	-0.0102
290.5	-0.05904	-0.01468	-0.04896	-0.0101
290	-0.05907	-0.01409	-0.04927	-0.01
289.5	-0.05911	-0.01338	-0.04961	-0.00979
289	-0.05915	-0.01257	-0.04995	-0.00941
288.5	-0.05921	-0.01168	-0.05033	-0.00886
288	-0.05926	-0.01071	-0.05078	-0.00823
287.5	-0.05932	-0.0097	-0.05136	-0.00765
287	-0.05936	-0.00864	-0.05209	-0.00727
286.5	-0.05939	-0.00753	-0.05299	-0.00717
286	-0.05942	-0.00636	-0.05401	-0.00737
285.5	-0.05944	-0.00513	-0.05506	-0.00776

285	-0.05946	-0.00386	-0.05601	-0.00824
284.5	-0.05949	-0.00257	-0.05673	-0.00872
284	-0.05952	-0.00128	-0.05718	-0.00919
283.5	-0.05954	-3.42E-05	-0.05744	-0.00969
283	-0.05956	0.001165	-0.05769	-0.01031
282.5	-0.05957	0.002326	-0.05815	-0.01116
282	-0.05956	0.00348	-0.05894	-0.01231
281.5	-0.05952	0.004665	-0.06007	-0.01383
281	-0.05947	0.005909	-0.06143	-0.01574
280.5	-0.0594	0.007228	-0.0628	-0.01801
280	-0.05933	0.00862	-0.06398	-0.02052
279.5	-0.05926	0.010068	-0.06484	-0.02309
279	-0.05919	0.011552	-0.06536	-0.02556
278.5	-0.05911	0.013045	-0.06561	-0.02785
278	-0.05904	0.014525	-0.06571	-0.03001
277.5	-0.05895	0.015976	-0.06581	-0.03223
277	-0.05884	0.017383	-0.06603	-0.03472
276.5	-0.05871	0.018735	-0.06641	-0.0376
276	-0.05857	0.020026	-0.06691	-0.04081
275.5	-0.05843	0.021249	-0.06742	-0.0441
275	-0.0583	0.022406	-0.06774	-0.04714
274.5	-0.0582	0.023498	-0.06769	-0.04954
274	-0.05814	0.024529	-0.06713	-0.05108
273.5	-0.05812	0.025498	-0.06606	-0.05167
273	-0.05813	0.026401	-0.06457	-0.05142
272.5	-0.05815	0.027237	-0.06284	-0.05058
272	-0.05816	0.028026	-0.06104	-0.04943
271.5	-0.05814	0.02881	-0.05927	-0.04826
271	-0.0581	0.029656	-0.0576	-0.04727
270.5	-0.05802	0.030638	-0.05604	-0.04658
270	-0.0579	0.031813	-0.05463	-0.0462
269.5	-0.05774	0.03321	-0.05343	-0.04607
269	-0.05755	0.034821	-0.05248	-0.04611
268.5	-0.05733	0.036612	-0.05181	-0.04617
268	-0.05708	0.038526	-0.05142	-0.04612
267.5	-0.05681	0.040494	-0.05127	-0.04587
267	-0.05652	0.042439	-0.05128	-0.04541
266.5	-0.05623	0.044288	-0.0514	-0.04487
266	-0.05592	0.045983	-0.05158	-0.04447
265.5	-0.05563	0.047489	-0.05179	-0.04446
265	-0.05535	0.048797	-0.05201	-0.04502
264.5	-0.05508	0.049921	-0.05218	-0.04615
264	-0.05482	0.050891	-0.05219	-0.04768

263.5	-0.05457	0.051752	-0.05195	-0.04932
263	-0.05433	0.052555	-0.05143	-0.05075
262.5	-0.05408	0.05335	-0.05071	-0.0517
262	-0.05381	0.054172	-0.05003	-0.05196
261.5	-0.05353	0.055027	-0.04966	-0.05139
261	-0.05323	0.055898	-0.04983	-0.04991
260.5	-0.05293	0.056759	-0.05063	-0.04744
260	-0.05264	0.05762	-0.05197	-0.04407
259.5	-0.05235	0.058551	-0.05371	-0.04004
259	-0.052	0.059688	-0.05569	-0.03584
258.5	-0.05156	0.061199	-0.05781	-0.03199
258	-0.05096	0.063217	-0.05995	-0.02893
257.5	-0.05019	0.065782	-0.06196	-0.02679
257	-0.04922	0.06882	-0.06371	-0.02538
256.5	-0.04809	0.072159	-0.0652	-0.02416
256	-0.04681	0.0756	-0.06666	-0.02246
255.5	-0.04541	0.078971	-0.06859	-0.01971
255	-0.04389	0.082155	-0.07154	-0.0157
254.5	-0.04225	0.085079	-0.07583	-0.01073
254	-0.04051	0.087709	-0.08138	-0.00555
253.5	-0.03868	0.090063	-0.08766	-0.00116
253	-0.03681	0.092211	-0.09396	0.001459
252.5	-0.03487	0.094234	-0.09965	0.001565
252	-0.03283	0.096155	-0.10433	-0.00134
251.5	-0.03058	0.097868	-0.10791	-0.00755
251	-0.02799	0.09911	-0.11042	-0.01705
250.5	-0.02494	0.099492	-0.11181	-0.02937
250	-0.02136	0.098613	-0.11182	-0.04339

Supplementary Table 15. Block loading for UV data in DCE

	PC1	PC2	PC3	PC4
455	-0.08259	-0.02443	0.050572	-0.04496
454	-0.08201	-0.02523	0.050481	-0.04156
453	-0.08204	-0.02382	0.053337	-0.04104
452	-0.08163	-0.02768	0.051478	-0.0429
451	-0.08334	-0.02907	0.052022	-0.0416
450	-0.08222	-0.03045	0.052913	-0.04204
449	-0.08164	-0.03189	0.053335	-0.04385
448	-0.08169	-0.03091	0.053196	-0.0444
447	-0.08126	-0.03681	0.053933	-0.0469
446	-0.08148	-0.03912	0.053656	-0.0423
445	-0.08054	-0.03794	0.054031	-0.04566
444	-0.07949	-0.04239	0.055147	-0.04823
443	-0.08038	-0.04297	0.0555	-0.0445
442	-0.07986	-0.04516	0.056049	-0.04884
441	-0.0812	-0.04417	0.055541	-0.04606
440	-0.08004	-0.04906	0.055838	-0.04746
439	-0.07999	-0.04823	0.057002	-0.04451
438	-0.07957	-0.05229	0.056214	-0.04729
437	-0.08002	-0.05169	0.058194	-0.04063
436	-0.07938	-0.05313	0.059322	-0.04577
435	-0.07927	-0.05559	0.057328	-0.0468
434	-0.07951	-0.05495	0.057971	-0.04553
433	-0.07849	-0.05934	0.05692	-0.04674
432	-0.07765	-0.05889	0.05837	-0.04366
431	-0.07757	-0.06172	0.057438	-0.04539
430	-0.07709	-0.06228	0.05842	-0.04824
429	-0.07571	-0.06538	0.058634	-0.05023
428	-0.07596	-0.06475	0.060092	-0.04688
427	-0.07485	-0.06726	0.059036	-0.05055
426	-0.07646	-0.06758	0.057763	-0.04629
425	-0.07394	-0.07041	0.058895	-0.0486
424	-0.07499	-0.0699	0.059056	-0.0475
423	-0.07406	-0.07166	0.059481	-0.04654
422	-0.07366	-0.07236	0.059862	-0.04603
421	-0.07263	-0.07433	0.059567	-0.04718
420	-0.07311	-0.07371	0.060192	-0.04592
419	-0.0727	-0.07359	0.061368	-0.04773
418	-0.07149	-0.07595	0.060538	-0.05049
417	-0.07216	-0.07453	0.060938	-0.05117
416	-0.07124	-0.07648	0.061098	-0.04878
415	-0.07128	-0.07664	0.061637	-0.05011

414	-0.07151	-0.07698	0.061208	-0.04789
413	-0.07007	-0.07778	0.062539	-0.05226
412	-0.07062	-0.07772	0.063035	-0.05024
411	-0.07021	-0.07834	0.062438	-0.05334
410	-0.07049	-0.07792	0.062917	-0.05072
409	-0.07056	-0.0779	0.06351	-0.05274
408	-0.06981	-0.07789	0.064388	-0.05626
407	-0.06993	-0.07727	0.064897	-0.05792
406	-0.07064	-0.07583	0.066282	-0.05851
405	-0.0715	-0.07453	0.0666	-0.05849
404	-0.0715	-0.0748	0.067581	-0.05522
403	-0.07087	-0.07447	0.068709	-0.05927
402	-0.07251	-0.07157	0.069156	-0.05974
401	-0.0716	-0.07151	0.07074	-0.06361
400	-0.0735	-0.06876	0.071522	-0.06307
399	-0.07355	-0.06865	0.071272	-0.0648
398	-0.0739	-0.06472	0.073887	-0.07224
397	-0.0751	-0.06301	0.074696	-0.07118
396	-0.0756	-0.05976	0.07797	-0.07095
395	-0.07727	-0.05701	0.076124	-0.07815
394	-0.07882	-0.05024	0.077905	-0.08559
393	-0.07942	-0.04463	0.080196	-0.09037
392	-0.08147	-0.03832	0.082112	-0.08518
391	-0.08139	-0.03258	0.085188	-0.08898
390	-0.08154	-0.02789	0.086256	-0.09384
389	-0.08232	-0.0161	0.088622	-0.09568
388	-0.08292	-0.00186	0.088439	-0.09791
387	-0.08354	0.004599	0.088245	-0.09527
386	-0.08126	0.019664	0.087746	-0.0977
385	-0.07886	0.026786	0.086425	-0.10545
384	-0.07499	0.042647	0.084555	-0.10259
383	-0.0731	0.049786	0.081326	-0.10275
382	-0.06911	0.061009	0.078722	-0.09479
381	-0.06104	0.075571	0.070298	-0.09948
380	-0.05772	0.082053	0.067126	-0.09226
379	-0.05051	0.091657	0.061588	-0.08872
378	-0.0459	0.097126	0.058362	-0.08692
377	-0.0453	0.100256	0.05405	-0.07976
376	-0.03648	0.106666	0.050255	-0.07468
375	-0.03571	0.108437	0.045744	-0.0673
374	-0.03417	0.109129	0.043133	-0.07183
373	-0.03096	0.111731	0.037833	-0.06736
372	-0.02994	0.112413	0.038556	-0.06495

371	-0.02656	0.114472	0.038496	-0.06244
370	-0.02535	0.115239	0.036028	-0.06417
369	-0.02452	0.116151	0.034803	-0.06147
368	-0.02306	0.116893	0.032275	-0.05951
367	-0.02336	0.116784	0.031555	-0.05818
366	-0.02173	0.117785	0.030938	-0.05688
365	-0.02189	0.117735	0.03046	-0.05711
364	-0.02304	0.117971	0.030447	-0.05298
363	-0.0212	0.118274	0.030321	-0.05645
362	-0.02453	0.117175	0.031876	-0.05488
361	-0.02298	0.117661	0.032231	-0.05851
360	-0.02458	0.116977	0.033241	-0.05489
359	-0.02538	0.116655	0.03356	-0.05589
358	-0.027	0.115722	0.035076	-0.05654
357	-0.02728	0.115602	0.035602	-0.05644
356	-0.02832	0.11475	0.036658	-0.05953
355	-0.02938	0.114218	0.038158	-0.05944
354	-0.03212	0.113012	0.039106	-0.06021
353	-0.03235	0.11226	0.041314	-0.06237
352	-0.03382	0.111226	0.042577	-0.06508
351	-0.03508	0.109901	0.045031	-0.06494
350	-0.03663	0.108923	0.045661	-0.06865
349	-0.03773	0.107806	0.048648	-0.06729
348	-0.04092	0.105686	0.050105	-0.06947
347	-0.04292	0.103428	0.052753	-0.07441
346	-0.04544	0.100271	0.055946	-0.0769
345	-0.04817	0.09724	0.058564	-0.07906
344	-0.05076	0.093749	0.061841	-0.08193
343	-0.0541	0.089573	0.065067	-0.08419
342	-0.05764	0.084225	0.069019	-0.08684
341	-0.06111	0.07858	0.071747	-0.09006
340	-0.06454	0.071236	0.075526	-0.09337
339	-0.06799	0.064053	0.078822	-0.09548
338	-0.07031	0.057937	0.081328	-0.09558
337	-0.07367	0.04849	0.084264	-0.09738
336	-0.07484	0.040639	0.08713	-0.09916
335	-0.07721	0.031663	0.088781	-0.09882
334	-0.07858	0.02044	0.090332	-0.09929
333	-0.07943	0.014929	0.09117	-0.09793
332	-0.08015	0.005837	0.091923	-0.09523
331	-0.0802	-0.00141	0.092375	-0.09489
330	-0.08034	-0.00888	0.091614	-0.09377
329	-0.07986	-0.01699	0.090482	-0.09192

328	-0.0798	-0.02172	0.089983	-0.08913
327	-0.07951	-0.02725	0.089455	-0.08367
326	-0.07867	-0.033	0.088041	-0.08218
325	-0.07769	-0.03849	0.086498	-0.08172
324	-0.07676	-0.04253	0.085976	-0.07947
323	-0.07646	-0.04479	0.085174	-0.07723
322	-0.07598	-0.04748	0.084112	-0.07639
321	-0.07584	-0.04887	0.083931	-0.07445
320	-0.0756	-0.05122	0.082659	-0.07163
319	-0.07483	-0.054	0.081486	-0.0706
318	-0.07533	-0.05453	0.080246	-0.06915
317	-0.07394	-0.05749	0.080023	-0.06922
316	-0.07422	-0.0588	0.078942	-0.06585
315	-0.07342	-0.06096	0.078359	-0.06452
314	-0.07298	-0.06203	0.078227	-0.06362
313	-0.07307	-0.06365	0.076703	-0.06248
312	-0.07212	-0.06475	0.07689	-0.06241
311	-0.07182	-0.06604	0.076202	-0.06143
310	-0.07222	-0.06631	0.075709	-0.0596
309	-0.07195	-0.06715	0.075028	-0.06027
308	-0.07194	-0.06739	0.074896	-0.05942
307	-0.07261	-0.06662	0.074884	-0.05846
306	-0.07295	-0.06627	0.074898	-0.05853
305	-0.07305	-0.0666	0.074307	-0.05895
304	-0.07374	-0.06525	0.074926	-0.05856
303	-0.07448	-0.0643	0.07511	-0.05818
302	-0.07481	-0.06394	0.075033	-0.05861
301	-0.07558	-0.06286	0.075222	-0.0585
300	-0.07607	-0.06181	0.075204	-0.05996
299	-0.07681	-0.06017	0.075758	-0.06105
298	-0.07794	-0.05779	0.076663	-0.06121
297	-0.07878	-0.05563	0.077055	-0.06246
296	-0.08002	-0.05271	0.077907	-0.06315
295	-0.08142	-0.04824	0.078748	-0.06563
294	-0.08219	-0.04439	0.080473	-0.06801
293	-0.08337	-0.03998	0.081542	-0.06809
292	-0.08452	-0.03303	0.082769	-0.07123
291	-0.08621	-0.02859	0.081564	-0.07425
290	-0.08642	-0.0247	0.083716	-0.07224
289	-0.08709	-0.0197	0.083518	-0.07587
288	-0.08783	-0.01561	0.083653	-0.07334
287	-0.08795	-0.01025	0.083899	-0.07492
286	-0.0878	-0.00607	0.083692	-0.08007

285	-0.08731	-0.00075	0.084399	-0.08301
284	-0.08702	0.003601	0.083975	-0.08426
283	-0.08781	0.01141	0.083352	-0.078
282	-0.08598	0.014662	0.084952	-0.08414
281	-0.08484	0.018209	0.084805	-0.08953
280	-0.08433	0.025207	0.085962	-0.07695
279	-0.08135	0.031909	0.085863	-0.08559
278	-0.08071	0.036323	0.08545	-0.08355
277	-0.08008	0.039903	0.083566	-0.08452
276	-0.08016	0.041802	0.081861	-0.08625
275	-0.07913	0.045151	0.082405	-0.08309
274	-0.07551	0.051062	0.082127	-0.08693
273	-0.07493	0.052959	0.082259	-0.08324
272	-0.07178	0.058974	0.081592	-0.08533
271	-0.0721	0.060856	0.079808	-0.07888
270	-0.06861	0.06684	0.077946	-0.08351
269	-0.06631	0.070121	0.076903	-0.08314
268	-0.06557	0.07293	0.074414	-0.08283
267	-0.06219	0.077673	0.072586	-0.0844
266	-0.06339	0.077688	0.072013	-0.07634
265	-0.06159	0.080651	0.070496	-0.07631
264	-0.05957	0.082979	0.071141	-0.07192
263	-0.05965	0.083068	0.071171	-0.06904
262	-0.05868	0.084646	0.070132	-0.06893
261	-0.05861	0.083469	0.070072	-0.07394
260	-0.05836	0.083896	0.070436	-0.06783
259	-0.05915	0.083553	0.069378	-0.06663
258	-0.05842	0.083049	0.07083	-0.06672
257	-0.0592	0.08071	0.071966	-0.06625
256	-0.06125	0.076552	0.074363	-0.06295
255	-0.06045	0.076006	0.075989	-0.06027
254	-0.06143	0.072346	0.076754	-0.05803
253	-0.06369	0.064824	0.080537	-0.05683
252	-0.06364	0.057865	0.083912	-0.06214
251	-0.06202	0.042599	0.091228	-0.06561
250	-0.06556	0.021205	0.091017	-0.07275

Supplementary Table 16. Comparison of actual **PEA** values vs. MBPCA + OLS prediction for both training and test sets

**Entry**	**Actual (*R*)-PEA (mM)**	**Predicted (*R*)-PEA (mM)**	**Actual (*S*)-PEA (mM)**	**Predicted (*S*)-PEA (mM)**
Training set 1	5.50	5.19	0.00	0.10
Training set 2	1.88	2.48	0.63	0.00
Training set 3	0.70	0.60	2.80	2.43
Training set 4	2.80	2.65	0.70	0.81
Training set 5	2.19	2.04	0.31	0.39
Training set 6	4.40	4.07	1.10	1.95
Training set 7	4.88	5.16	1.63	1.10
Training set 8	1.75	1.25	1.75	2.55
Training set 9	2.45	1.90	1.05	1.58
Training set 10	0.75	0.71	1.75	2.35
Training set 11	3.75	4.09	3.75	2.66
Training set 12	0.63	0.71	1.88	1.81
Training set 13	5.25	5.34	2.25	2.40
Training set 14	0.00	0.07	4.50	4.40
Training set 15	7.50	7.29	0.00	0.00
Training set 16	2.81	2.73	1.69	1.59
Training set 17	2.75	3.16	2.75	2.38
Training set 18	4.06	3.81	2.44	2.67
Training set 19	6.00	6.09	1.50	1.74
Training set 20	3.50	3.76	0.00	0.00
Test set 1	3.38	3.62	1.13	1.00
Test set 2	5.69	6.00	0.81	0.67
Test set 3	0.94	1.19	1.56	2.24
Test set 4	0.56	0.48	3.94	4.16
Test set 5	3.85	4.19	1.65	1.33
Test set 6	3.94	4.00	0.56	0.35

Supplementary Table 17. Comparison of actual **PMP** values vs. MBPCA + OLS prediction for both training and test sets

**Entry**	**Actual (*R*)-PMP (mM)**	**Predicted (*R*)-PMP (mM)**	**Actual (*S*)-PMP (mM)**	**Predicted (*S*)-PMP (mM)**
Training set 1	4.50	4.49	0.00	0.22
Training set 2	5.63	5.93	1.88	1.61
Training set 3	1.30	1.56	5.20	5.42
Training set 4	5.20	5.17	1.30	1.36
Training set 5	6.56	6.60	0.94	0.98
Training set 6	3.60	3.20	0.90	0.78
Training set 7	2.63	2.88	0.88	0.86
Training set 8	3.25	2.72	3.25	3.49
Training set 9	4.55	4.39	1.95	2.12
Training set 10	2.25	1.95	5.25	4.98
Training set 11	1.25	1.92	1.25	1.33
Training set 12	1.88	1.89	5.63	5.59
Training set 13	1.75	1.56	0.75	0.70
Training set 14	0.00	0.02	5.50	5.52
Training set 15	2.50	2.61	0.00	0.21
Training set 16	3.44	3.59	2.06	2.09
Training set 17	2.25	2.29	2.25	2.17
Training set 18	2.19	2.11	1.31	1.40
Training set 19	2.00	1.89	0.50	0.28
Training set 20	6.50	6.42	0.00	0.00
Test set 1	4.13	3.98	1.38	1.40
Test set 2	3.06	3.03	0.44	0.30
Test set 3	2.81	2.30	4.69	4.27
Test set 4	0.69	0.52	4.81	4.84
Test set 5	3.15	3.23	1.35	1.25
Test set 6	4.81	5.12	0.69	0.53

Supplementary Table 18. Comparison of actual **PPA** values vs. MBPCA + OLS prediction for both training and test sets

**Entry**	**Actual (1*R*, 2*S*)-PPA (mM)**	**Predicted (1*R*, 2*S*)-PPA (mM)**	**Actual (1*S*, 2*R*)-PPA (mM)**	**Predicted (1*S*, 2*R*)-PMP (mM)**
Training set 1	5.50	5.19	0.00	0.10
Training set 2	1.88	2.48	0.63	0.00
Training set 3	0.70	0.60	2.80	2.43
Training set 4	2.80	2.65	0.70	0.81
Training set 5	2.19	2.04	0.31	0.39
Training set 6	4.40	4.07	1.10	1.95
Training set 7	4.88	5.16	1.63	1.10
Training set 8	1.75	1.25	1.75	2.55
Training set 9	2.45	1.90	1.05	1.58
Training set 10	0.75	0.71	1.75	2.35
Training set 11	3.75	4.09	3.75	2.66
Training set 12	0.63	0.71	1.88	1.81
Training set 13	5.25	5.34	2.25	2.40
Training set 14	0.00	0.07	4.50	4.40
Training set 15	7.50	7.29	0.00	0.00
Training set 16	2.81	2.73	1.69	1.59
Training set 17	2.75	3.16	2.75	2.38
Training set 18	4.06	3.81	2.44	2.67
Training set 19	6.00	6.09	1.50	1.74
Training set 20	3.50	3.76	0.00	0.00
Test set 1	3.38	3.62	1.13	1.00
Test set 2	5.69	6.00	0.81	0.67
Test set 3	0.94	1.19	1.56	2.24
Test set 4	0.56	0.48	3.94	4.16
Test set 5	3.85	4.19	1.65	1.33
Test set 6	3.94	4.00	0.56	0.35

Supplementary Table 19. Comparison of actual **PGL** values vs. MBPCA + OLS prediction for both training and test sets

**Entry**	**Actual (*R*)-PGL (mM)**	**Predicted (*R*)-PGL (mM)**	**Actual (*S*)-PGL (mM)**	**Predicted (*S*)-PGL (mM)**
Training set 1	4.50	4.49	0.00	0.22
Training set 2	5.63	5.93	1.88	1.61
Training set 3	1.30	1.56	5.20	5.42
Training set 4	5.20	5.17	1.30	1.36
Training set 5	6.56	6.60	0.94	0.98
Training set 6	3.60	3.20	0.90	0.78
Training set 7	2.63	2.88	0.88	0.86
Training set 8	3.25	2.72	3.25	3.49
Training set 9	4.55	4.39	1.95	2.12
Training set 10	2.25	1.95	5.25	4.98
Training set 11	1.25	1.92	1.25	1.33
Training set 12	1.88	1.89	5.63	5.59
Training set 13	1.75	1.56	0.75	0.70
Training set 14	0.00	0.02	5.50	5.52
Training set 15	2.50	2.61	0.00	0.21
Training set 16	3.44	3.59	2.06	2.09
Training set 17	2.25	2.29	2.25	2.17
Training set 18	2.19	2.11	1.31	1.40
Training set 19	2.00	1.89	0.50	0.28
Training set 20	6.50	6.42	0.00	0.00
Test set 1	4.13	3.98	1.38	1.40
Test set 2	3.06	3.03	0.44	0.30
Test set 3	2.81	2.30	4.69	4.27
Test set 4	0.69	0.52	4.81	4.84
Test set 5	3.15	3.23	1.35	1.25
Test set 6	4.81	5.12	0.69	0.53

Supplementary Table 20. MBPLS Block loading for CD data in DCE for (*R*)-**PEA**

	LV1	LV2	LV3	LV4
250	1.003125	-3.72214	0.486258	-0.64728
250.5	1.370417	-3.73309	0.399758	-0.70532
251	1.687384	-3.67829	0.32182	-0.74279
251.5	1.955917	-3.58231	0.260107	-0.75881
252	2.184599	-3.46951	0.214752	-0.75372
252.5	2.383153	-3.3564	0.181947	-0.72864
253	2.559581	-3.25035	0.158379	-0.68619
253.5	2.719247	-3.15186	0.143728	-0.63075
254	2.865192	-3.05791	0.140554	-0.56788
254.5	2.999197	-2.96486	0.15216	-0.50297
255	3.122875	-2.86992	0.179906	-0.43984
255.5	3.237949	-2.77137	0.221593	-0.38016
256	3.345542	-2.66897	0.271609	-0.32392
256.5	3.445092	-2.56482	0.322761	-0.27047
257	3.534086	-2.46394	0.368825	-0.21949
257.5	3.609091	-2.37335	0.406489	-0.17124
258	3.667474	-2.2994	0.435747	-0.12631
258.5	3.70869	-2.24518	0.458795	-0.08526
259	3.734598	-2.20939	0.478576	-0.04865
259.5	3.748938	-2.18723	0.497891	-0.01731
260	3.756295	-2.17267	0.5192	0.00753
260.5	3.760815	-2.1608	0.544572	0.024466
261	3.765235	-2.14912	0.575157	0.032473
261.5	3.770677	-2.1373	0.610281	0.031435
262	3.777241	-2.12602	0.646925	0.022262
262.5	3.784836	-2.11573	0.680293	0.006541
263	3.793685	-2.10603	0.705367	-0.01404
263.5	3.804278	-2.09574	0.71876	-0.03808
264	3.817082	-2.08324	0.719909	-0.06451
264.5	3.832348	-2.06681	0.711093	-0.09219
265	3.850145	-2.04493	0.696379	-0.11958
265.5	3.870449	-2.01633	0.680019	-0.14479
266	3.893141	-1.98026	0.665055	-0.16611
266.5	3.917917	-1.93676	0.652675	-0.18276
267	3.944205	-1.8868	0.642442	-0.19542
267.5	3.971231	-1.83221	0.633128	-0.20603
268	3.998181	-1.77533	0.623664	-0.21704
268.5	4.024306	-1.71851	0.61368	-0.23056
269	4.048938	-1.66388	0.603443	-0.24776
269.5	4.071445	-1.61319	0.593367	-0.26876
270	4.09125	-1.56778	0.583513	-0.29291

270.5	4.107923	-1.52841	0.57347	-0.31929
271	4.121288	-1.49504	0.562653	-0.34718
271.5	4.131493	-1.46678	0.550757	-0.37624
272	4.139028	-1.44208	0.537965	-0.40621
272.5	4.144716	-1.41916	0.524721	-0.43633
273	4.149673	-1.39643	0.51123	-0.46514
273.5	4.155181	-1.37263	0.49708	-0.49083
274	4.162436	-1.3468	0.48131	-0.51212
274.5	4.172212	-1.31819	0.462966	-0.52906
275	4.184572	-1.28624	0.441817	-0.54305
275.5	4.198847	-1.25071	0.41876	-0.5561
276	4.213888	-1.21176	0.395617	-0.56998
276.5	4.228481	-1.16989	0.374464	-0.58562
277	4.241732	-1.12582	0.35681	-0.60303
277.5	4.25326	-1.08021	0.343145	-0.62163
278	4.263184	-1.03359	0.332971	-0.6404
278.5	4.271968	-0.98631	0.325238	-0.65811
279	4.280204	-0.9386	0.318859	-0.67332
279.5	4.288424	-0.89064	0.313044	-0.68459
280	4.29693	-0.84269	0.307348	-0.69092
280.5	4.305678	-0.79512	0.301576	-0.69238
281	4.31425	-0.74842	0.295655	-0.69053
281.5	4.321977	-0.70296	0.289593	-0.68826
282	4.328207	-0.65874	0.283469	-0.68878
282.5	4.332576	-0.61521	0.277365	-0.69426
283	4.335177	-0.57135	0.271231	-0.70477
283.5	4.336544	-0.52611	0.264738	-0.71826
284	4.337463	-0.47889	0.257321	-0.73148
284.5	4.338655	-0.42998	0.248438	-0.74152
285	4.340483	-0.3804	0.237983	-0.74688
285.5	4.342836	-0.33145	0.226556	-0.74774
286	4.345256	-0.28425	0.21538	-0.74537
286.5	4.347202	-0.23937	0.20582	-0.7413
287	4.348292	-0.19691	0.198778	-0.73685
287.5	4.348397	-0.15672	0.194304	-0.73293
288	4.347645	-0.11874	0.19162	-0.73
288.5	4.346349	-0.0832	0.189525	-0.72804
289	4.344922	-0.05066	0.186942	-0.72659
289.5	4.343771	-0.0219	0.183355	-0.7249
290	4.343201	0.002281	0.178987	-0.72222
290.5	4.343355	0.021243	0.174711	-0.71823
291	4.344164	0.034628	0.171808	-0.71332
291.5	4.345357	0.04244	0.17162	-0.70873

292	4.346512	0.045033	0.175206	-0.70625
292.5	4.347172	0.043045	0.183027	-0.7077
293	4.347001	0.037346	0.194692	-0.71408
293.5	4.345897	0.029032	0.208832	-0.72504
294	4.344068	0.019416	0.223208	-0.73869
294.5	4.341993	0.009922	0.235158	-0.75206
295	4.340296	0.001792	0.242406	-0.76207
295.5	4.339509	-0.00433	0.243925	-0.76658
296	4.339863	-0.0087	0.240462	-0.76515
296.5	4.341194	-0.01247	0.234346	-0.75915
297	4.343046	-0.01732	0.228663	-0.75115
297.5	4.344893	-0.02496	0.226143	-0.74384
298	4.346349	-0.03667	0.228378	-0.73893
298.5	4.347277	-0.05311	0.235609	-0.73643
299	4.347786	-0.07418	0.247001	-0.73474
299.5	4.348141	-0.09918	0.261102	-0.73146
300	4.348616	-0.127	0.276205	-0.72442
300.5	4.349349	-0.15658	0.290512	-0.71257
301	4.350293	-0.18728	0.302219	-0.69616
301.5	4.351261	-0.2192	0.309628	-0.67644
302	4.352072	-0.25317	0.311376	-0.65492
302.5	4.352659	-0.29047	0.306672	-0.63273
303	4.353065	-0.33227	0.295429	-0.61031
303.5	4.353322	-0.37893	0.278264	-0.58753
304	4.35335	-0.42942	0.256305	-0.56426
304.5	4.352989	-0.4811	0.230895	-0.54087
305	4.352126	-0.53024	0.203254	-0.51848
305.5	4.350775	-0.57312	0.17427	-0.49856
306	4.348982	-0.6074	0.144444	-0.48226
306.5	4.346645	-0.63289	0.114059	-0.4699
307	4.34343	-0.65156	0.083452	-0.46099
307.5	4.338887	-0.66676	0.053194	-0.45453
308	4.332717	-0.68207	0.024102	-0.44937
308.5	4.324987	-0.70025	-0.00298	-0.44435
309	4.316183	-0.72251	-0.02752	-0.43824
309.5	4.307067	-0.74814	-0.04946	-0.42982
310	4.29841	-0.77473	-0.06903	-0.4184
310.5	4.290782	-0.79879	-0.08616	-0.40442
311	4.284443	-0.81715	-0.09988	-0.39003
311.5	4.279321	-0.82836	-0.10815	-0.379
312	4.274989	-0.83382	-0.1086	-0.37551
312.5	4.270649	-0.83798	-0.09952	-0.38252
313	4.265204	-0.84736	-0.0808	-0.40023

313.5	4.257402	-0.86893	-0.05393	-0.42553
314	4.245927	-0.90818	-0.02126	-0.45272
314.5	4.229347	-0.96769	0.014957	-0.47531
315	4.205982	-1.04645	0.053123	-0.48803
315.5	4.173914	-1.14028	0.092046	-0.48849
316	4.131269	-1.24339	0.130318	-0.47762
316.5	4.076652	-1.35033	0.166099	-0.45906
317	4.009504	-1.45759	0.197712	-0.43758
317.5	3.930323	-1.56402	0.224505	-0.41717
318	3.840759	-1.67051	0.247022	-0.3993
318.5	3.743459	-1.77922	0.265896	-0.38169
319	3.641404	-1.89294	0.279977	-0.35837
319.5	3.53676	-2.01396	0.284974	-0.32148
320	3.429823	-2.14258	0.273905	-0.26489
320.5	3.318818	-2.27541	0.239672	-0.18804
321	3.200682	-2.40508	0.178663	-0.09846
321.5	3.072107	-2.52228	0.092913	-0.01087
322	2.92992	-2.61911	-0.0112	0.057252
322.5	2.770414	-2.69081	-0.12738	0.091045
323	2.588648	-2.73435	-0.25381	0.081511
323.5	2.379145	-2.74545	-0.39399	0.025349
324	2.138658	-2.71708	-0.55368	-0.07632
324.5	1.870236	-2.64136	-0.73619	-0.21821
325	1.585883	-2.51389	-0.93976	-0.39002
325.5	1.304309	-2.33628	-1.15898	-0.57745
326	1.043709	-2.11352	-1.38818	-0.76653
326.5	0.815229	-1.84821	-1.62212	-0.94873
327	0.622558	-1.53808	-1.85274	-1.12259
327.5	0.466174	-1.18039	-2.06602	-1.29053
328	0.346076	-0.77973	-2.24406	-1.45362
328.5	0.259174	-0.35231	-2.37216	-1.60839
329	0.194052	0.077123	-2.44607	-1.7479
329.5	0.12888	0.483459	-2.47352	-1.86544
330	0.035512	0.848326	-2.46945	-1.95691
330.5	-0.11171	1.1623	-2.44836	-2.01975
331	-0.32556	1.42307	-2.41915	-2.05004
331.5	-0.59753	1.633588	-2.38494	-2.04189
332	-0.89654	1.802762	-2.34719	-1.99118
332.5	-1.17948	1.946357	-2.30956	-1.90063
333	-1.40895	2.083777	-2.27718	-1.78065
333.5	-1.56676	2.230263	-2.25203	-1.64484
334	-1.65679	2.389758	-2.22987	-1.50458
334.5	-1.69979	2.553587	-2.20227	-1.36667

335	-1.72427	2.705721	-2.16206	-1.23414
335.5	-1.75661	2.830561	-2.10734	-1.10879
336	-1.81373	2.918409	-2.04134	-0.9939
336.5	-1.9007	2.966976	-1.96933	-0.8954
337	-2.01198	2.980744	-1.89599	-0.82013
337.5	-2.13403	2.969846	-1.82472	-0.77175
338	-2.24846	2.948618	-1.75864	-0.74757
338.5	-2.33643	2.932976	-1.70147	-0.73928
339	-2.38331	2.936461	-1.65688	-0.73721
339.5	-2.38271	2.965948	-1.62628	-0.73504
340	-2.33924	3.019147	-1.60679	-0.73195
340.5	-2.2698	3.085512	-1.59107	-0.73166
341	-2.20116	3.150759	-1.56993	-0.73941
341.5	-2.16139	3.202542	-1.53626	-0.75857
342	-2.16821	3.234047	-1.48803	-0.78865
342.5	-2.22155	3.244534	-1.4288	-0.82592
343	-2.30567	3.23811	-1.3659	-0.86575
343.5	-2.39889	3.221598	-1.30715	-0.90493
344	-2.48362	3.201695	-1.25762	-0.9421
344.5	-2.55137	3.182596	-1.21831	-0.97652
345	-2.602	3.165325	-1.18706	-1.00678
345.5	-2.63996	3.148807	-1.16074	-1.03043
346	-2.6706	3.131464	-1.13715	-1.04489
346.5	-2.69791	3.112354	-1.11568	-1.04854
347	-2.72405	3.0916	-1.09673	-1.04145
347.5	-2.74981	3.070252	-1.08063	-1.02508
348	-2.77522	3.049987	-1.06697	-1.00154
348.5	-2.79981	3.032794	-1.05487	-0.97275
349	-2.82256	3.020545	-1.04385	-0.9402
349.5	-2.84209	3.014438	-1.03443	-0.90508
350	-2.85708	3.014424	-1.02792	-0.86881
350.5	-2.86695	3.018952	-1.02546	-0.83333
351	-2.87243	3.025254	-1.0269	-0.80119
351.5	-2.87562	3.029983	-1.03039	-0.77503
352	-2.87961	3.029961	-1.03285	-0.75687
352.5	-2.88774	3.022813	-1.03104	-0.74711
353	-2.90264	3.007467	-1.02266	-0.74403
353.5	-2.92552	2.984355	-1.00716	-0.74406
354	-2.95594	2.955063	-0.98581	-0.74308
354.5	-2.9925	2.921303	-0.96112	-0.73822
355	-3.03412	2.883682	-0.93576	-0.72897
355.5	-3.0808	2.84109	-0.91168	-0.71686
356	-3.13354	2.791174	-0.88973	-0.70407

356.5	-3.19316	2.731681	-0.86992	-0.69204
357	-3.25901	2.661893	-0.85197	-0.68085
357.5	-3.32848	2.583424	-0.83576	-0.6696
358	-3.39774	2.499931	-0.82153	-0.65717
358.5	-3.46332	2.415849	-0.80976	-0.64257
359	-3.52336	2.334717	-0.80094	-0.62481
359.5	-3.57814	2.25799	-0.79513	-0.60241
360	-3.62957	2.184903	-0.79188	-0.57338
360.5	-3.6801	2.113345	-0.79028	-0.53572
361	-3.73172	2.040945	-0.7895	-0.48834
361.5	-3.78522	1.965853	-0.78915	-0.43194
362	-3.83992	1.887097	-0.78939	-0.36904
362.5	-3.894	1.804744	-0.79061	-0.30319
363	-3.9451	1.720039	-0.79276	-0.23754
363.5	-3.99118	1.635211	-0.79496	-0.17379
364	-4.03109	1.552747	-0.79562	-0.11219
364.5	-4.06471	1.474375	-0.79301	-0.05253
365	-4.0927	1.400397	-0.78625	0.004647
365.5	-4.11616	1.329747	-0.77576	0.057737
366	-4.13632	1.260542	-0.76298	0.104906
366.5	-4.15446	1.190627	-0.74922	0.145422
367	-4.17183	1.117854	-0.73448	0.180295
367.5	-4.18954	1.040229	-0.71725	0.2117
368	-4.20835	0.95633	-0.69548	0.241602
368.5	-4.22846	0.86599	-0.6682	0.270635
369	-4.24947	0.770827	-0.6367	0.297937
369.5	-4.27057	0.674048	-0.60412	0.321995
370	-4.29082	0.579396	-0.57405	0.341705
370.5	-4.30929	0.489723	-0.54892	0.356864
371	-4.32516	0.406022	-0.52933	0.367951
371.5	-4.33786	0.327414	-0.51457	0.375585
372	-4.34718	0.25201	-0.50373	0.380234
372.5	-4.35319	0.178138	-0.49654	0.382436
373	-4.35604	0.105205	-0.49341	0.383323
373.5	-4.35582	0.033727	-0.49479	0.384909
374	-4.35254	-0.03535	-0.50035	0.389802
374.5	-4.34642	-0.10153	-0.50868	0.400249
375	-4.33809	-0.16528	-0.51761	0.416997
375.5	-4.32865	-0.22793	-0.52481	0.438632
376	-4.31944	-0.29106	-0.52836	0.461908
376.5	-4.31165	-0.35575	-0.52701	0.482988
377	-4.30594	-0.42202	-0.52027	0.498943
377.5	-4.30224	-0.48879	-0.50846	0.508641

378	-4.29991	-0.55414	-0.49271	0.512674
378.5	-4.29808	-0.61573	-0.47463	0.512696
379	-4.29598	-0.6712	-0.45586	0.510674
379.5	-4.29323	-0.7187	-0.43741	0.508454
380	-4.28999	-0.75742	-0.41943	0.507519
380.5	-4.28684	-0.78785	-0.40147	0.508842
381	-4.2845	-0.81172	-0.38288	0.51272
381.5	-4.28351	-0.83123	-0.36338	0.518708
382	-4.28397	-0.84831	-0.3432	0.525793
382.5	-4.28548	-0.86404	-0.32297	0.532809
383	-4.28733	-0.87868	-0.30352	0.538861
383.5	-4.28881	-0.892	-0.28575	0.543536
384	-4.28949	-0.90372	-0.27056	0.54688
384.5	-4.28937	-0.91352	-0.25882	0.549263
385	-4.28891	-0.92094	-0.25131	0.551324
385.5	-4.2887	-0.92527	-0.24854	0.553962
386	-4.28922	-0.92577	-0.25066	0.55837
386.5	-4.29049	-0.92224	-0.25747	0.565895
387	-4.29201	-0.91551	-0.26861	0.577794
387.5	-4.29285	-0.90754	-0.28368	0.594974
388	-4.29205	-0.90085	-0.30231	0.617811
388.5	-4.2889	-0.89772	-0.32395	0.646057
389	-4.2831	-0.89956	-0.3475	0.678795
389.5	-4.27478	-0.9068	-0.37117	0.714411
390	-4.26438	-0.91915	-0.39264	0.750674
390.5	-4.25265	-0.93595	-0.40957	0.785115
391	-4.24045	-0.9563	-0.42033	0.815549
391.5	-4.22863	-0.97898	-0.42457	0.840568
392	-4.21796	-1.00231	-0.42318	0.859688
392.5	-4.20906	-1.02425	-0.41773	0.873136
393	-4.20241	-1.04278	-0.40979	0.88143
393.5	-4.19829	-1.05658	-0.40032	0.885067
394	-4.19659	-1.06547	-0.38958	0.884497
394.5	-4.19678	-1.07045	-0.37722	0.880276
395	-4.1981	-1.07319	-0.36261	0.873155
395.5	-4.19984	-1.07535	-0.3451	0.863872
396	-4.20174	-1.07794	-0.32425	0.852669
396.5	-4.20402	-1.08112	-0.30013	0.838956
397	-4.20715	-1.08419	-0.2736	0.821603
397.5	-4.21147	-1.08568	-0.24652	0.799901
398	-4.21693	-1.08373	-0.22162	0.774712
398.5	-4.22309	-1.0765	-0.20186	0.748743
399	-4.22946	-1.06278	-0.18962	0.725667

399.5	-4.23568	-1.04247	-0.18601	0.708572
400	-4.24159	-1.01675	-0.19079	0.698782
400.5	-4.24699	-0.98801	-0.20274	0.695758
401	-4.25148	-0.95954	-0.22047	0.697834
401.5	-4.25431	-0.93488	-0.24314	0.703233
402	-4.25456	-0.91707	-0.27077	0.710662
402.5	-4.2515	-0.90795	-0.30382	0.719086
403	-4.24499	-0.9078	-0.3423	0.727047
403.5	-4.23569	-0.91547	-0.38496	0.732217
404	-4.225	-0.92869	-0.42922	0.731921
404.5	-4.21459	-0.94468	-0.47198	0.724654
405	-4.2057	-0.96076	-0.51096	0.711633
405.5	-4.19861	-0.97504	-0.54537	0.697003
406	-4.19262	-0.98682	-0.57556	0.686149
406.5	-4.18672	-0.99673	-0.60192	0.68315
407	-4.18017	-1.00655	-0.62377	0.68881
407.5	-4.17304	-1.01857	-0.63938	0.700225
408	-4.16623	-1.03487	-0.64676	0.712035
408.5	-4.16093	-1.05643	-0.64493	0.71859
409	-4.15799	-1.08257	-0.6345	0.715962
409.5	-4.15754	-1.11084	-0.61725	0.703055
410	-4.1593	-1.13751	-0.59514	0.681507
410.5	-4.16298	-1.15867	-0.56945	0.654929
411	-4.16846	-1.17132	-0.54092	0.627883
411.5	-4.1755	-1.17418	-0.51027	0.604946
412	-4.18351	-1.16784	-0.47866	0.58973
412.5	-4.19182	-1.15411	-0.44736	0.583841
413	-4.20018	-1.13524	-0.41704	0.585798
413.5	-4.20914	-1.11331	-0.38718	0.590558
414	-4.21981	-1.09016	-0.35657	0.590425
414.5	-4.23286	-1.06739	-0.32444	0.577968
415	-4.24763	-1.04628	-0.29166	0.54967
415.5	-4.26223	-1.02751	-0.2609	0.507874
416	-4.27458	-1.01109	-0.23547	0.459433
416.5	-4.28343	-0.99659	-0.21784	0.412353
417	-4.28852	-0.98353	-0.20861	0.372708
417.5	-4.29026	-0.97177	-0.20644	0.343144
418	-4.28936	-0.96186	-0.20869	0.322716
418.5	-4.28664	-0.95512	-0.21237	0.307676
419	-4.28286	-0.95355	-0.21513	0.292914
419.5	-4.27847	-0.95901	-0.21554	0.273842
420	-4.27351	-0.97231	-0.21303	0.248069
420.5	-4.26764	-0.99247	-0.20778	0.216216

421	-4.26047	-1.01703	-0.20072	0.18152
421.5	-4.25171	-1.04314	-0.19363	0.148507
422	-4.24111	-1.06884	-0.18893	0.121359
422.5	-4.22827	-1.09369	-0.18888	0.102674
423	-4.21273	-1.11862	-0.19485	0.092759
423.5	-4.19426	-1.14542	-0.20669	0.089535
424	-4.17321	-1.17604	-0.22273	0.089032
424.5	-4.15057	-1.21176	-0.24034	0.086605
425	-4.12754	-1.25245	-0.25683	0.07855
425.5	-4.1052	-1.29607	-0.27037	0.063471
426	-4.08421	-1.33904	-0.28039	0.042685
426.5	-4.065	-1.37727	-0.28723	0.019496
427	-4.04786	-1.40739	-0.29149	-0.00213
427.5	-4.03303	-1.42764	-0.29369	-0.01883
428	-4.02079	-1.43814	-0.29439	-0.02871
428.5	-4.01156	-1.44085	-0.29478	-0.03161
429	-4.0058	-1.43912	-0.29692	-0.02881
429.5	-4.00372	-1.43714	-0.30364	-0.02213
430	-4.00457	-1.43907	-0.31759	-0.0125
430.5	-4.00632	-1.44814	-0.34044	0.001182
431	-4.00605	-1.46598	-0.37219	0.022172
431.5	-4.00092	-1.49255	-0.41086	0.054675
432	-3.98934	-1.52587	-0.45232	0.101877
432.5	-3.97167	-1.56201	-0.49055	0.163873
433	-3.95046	-1.59573	-0.51889	0.236514
433.5	-3.92964	-1.62221	-0.53234	0.312196
434	-3.91246	-1.63924	-0.52955	0.382868
434.5	-3.89971	-1.64843	-0.51289	0.443402

Supplementary Table 21. MBPLS Block loading for UV data in DCE for (*R*)-**PEA**

	LV1	LV2	LV3	LV4
250	-2.14891	1.657646	2.343056	-1.35427
251	-1.78643	2.38618	2.375418	-1.22673
252	-1.72828	2.899257	2.099831	-1.19504
253	-1.67096	3.1293	2.003168	-1.11173
254	-1.54687	3.324477	1.845302	-1.14732
255	-1.51358	3.430735	1.803796	-1.10725
256	-1.55442	3.462674	1.750016	-1.0856
257	-1.42805	3.580498	1.681885	-1.06954
258	-1.39607	3.656131	1.65993	-0.97442
259	-1.4255	3.672195	1.605654	-0.99391
260	-1.42309	3.658174	1.628573	-1.01319
261	-1.43686	3.655426	1.624105	-1.00638
262	-1.43544	3.681587	1.640576	-0.961
263	-1.50439	3.648773	1.654834	-0.98919
264	-1.49494	3.628758	1.68033	-0.98093
265	-1.60143	3.587545	1.646112	-0.98711
266	-1.71179	3.503653	1.705509	-0.98015
267	-1.69865	3.487211	1.706686	-0.98228
268	-1.86787	3.366879	1.793303	-0.95206
269	-1.89631	3.293234	1.872055	-1.00741
270	-2.05182	3.182732	1.922203	-0.96123
271	-2.26111	3.016109	1.983354	-0.97369
272	-2.23629	2.977167	2.060155	-0.95546
273	-2.46451	2.778984	2.086459	-0.9225
274	-2.49896	2.725036	2.072617	-0.95823
275	-2.68659	2.540807	2.131975	-0.8773
276	-2.74924	2.44998	2.123951	-0.88773
277	-2.81084	2.36404	2.148615	-0.84828
278	-2.85255	2.269935	2.210095	-0.86481
279	-2.91825	2.136582	2.211684	-0.92079
280	-3.10712	1.918954	2.267153	-0.7833
281	-3.18508	1.695526	2.22544	-0.85962
282	-3.24595	1.561082	2.276083	-0.78496
283	-3.34146	1.464167	2.208896	-0.83874
284	-3.3864	1.200043	2.264523	-0.83005
285	-3.43653	1.04693	2.304067	-0.77377
286	-3.49755	0.858964	2.302703	-0.72637
287	-3.52922	0.71483	2.335446	-0.71535
288	-3.57873	0.540563	2.308658	-0.77235
289	-3.58463	0.374176	2.326782	-0.68527
290	-3.60469	0.200287	2.324248	-0.71446

291	-3.61776	0.074438	2.313511	-0.66035
292	-3.5894	-0.10199	2.364751	-0.67207
293	-3.60623	-0.35959	2.318714	-0.66981
294	-3.59122	-0.52596	2.316557	-0.62634
295	-3.59923	-0.66317	2.28435	-0.58252
296	-3.58064	-0.83235	2.268396	-0.57196
297	-3.55717	-0.94091	2.25601	-0.56055
298	-3.54222	-1.02085	2.247951	-0.56338
299	-3.51907	-1.11225	2.236078	-0.53355
300	-3.50011	-1.17675	2.234109	-0.51611
301	-3.49131	-1.22236	2.225028	-0.53715
302	-3.46856	-1.26716	2.231645	-0.51193
303	-3.45776	-1.28671	2.231338	-0.51902
304	-3.44034	-1.31757	2.230839	-0.53028
305	-3.42148	-1.37155	2.225391	-0.50277
306	-3.41402	-1.35694	2.245321	-0.52749
307	-3.41047	-1.37821	2.22658	-0.52956
308	-3.38874	-1.41062	2.239112	-0.51667
309	-3.38663	-1.39694	2.245892	-0.52645
310	-3.39076	-1.37286	2.256671	-0.5298
311	-3.37217	-1.36557	2.274544	-0.52497
312	-3.37048	-1.31932	2.285692	-0.55698
313	-3.40273	-1.26833	2.27948	-0.5515
314	-3.37444	-1.21917	2.324654	-0.58408
315	-3.3888	-1.17332	2.324062	-0.60028
316	-3.4054	-1.09165	2.328221	-0.61269
317	-3.38421	-1.04423	2.360383	-0.62738
318	-3.40845	-0.93578	2.363144	-0.63303
319	-3.39363	-0.9194	2.387891	-0.64757
320	-3.39197	-0.81874	2.416869	-0.66481
321	-3.38632	-0.73627	2.439438	-0.68216
322	-3.38199	-0.68696	2.439301	-0.68978
323	-3.3769	-0.59051	2.458371	-0.72485
324	-3.3699	-0.50898	2.479886	-0.72527
325	-3.36688	-0.36238	2.487598	-0.75903
326	-3.36378	-0.1695	2.51656	-0.77163
327	-3.34743	0.037434	2.536594	-0.80535
328	-3.31443	0.230668	2.537161	-0.82373
329	-3.27388	0.401828	2.542121	-0.85969
330	-3.22698	0.683715	2.537868	-0.92036
331	-3.15257	0.932125	2.537203	-0.93934
332	-3.09498	1.191261	2.506464	-0.95995
333	-2.98475	1.503547	2.465545	-0.96618

334	-2.90727	1.693214	2.417524	-0.9944
335	-2.74645	2.070078	2.342275	-1.00703
336	-2.5578	2.362926	2.278958	-1.01907
337	-2.44838	2.615556	2.145652	-1.01039
338	-2.2214	2.902672	2.043216	-1.01789
339	-2.0691	3.082999	1.947432	-1.0117
340	-1.86517	3.300956	1.834608	-0.98092
341	-1.66104	3.499417	1.697009	-0.97301
342	-1.44425	3.654319	1.615068	-0.94091
343	-1.26221	3.788686	1.472332	-0.9134
344	-1.0888	3.891462	1.364335	-0.90281
345	-0.94745	3.982412	1.256133	-0.87068
346	-0.80319	4.048657	1.175332	-0.84793
347	-0.67816	4.10484	1.08342	-0.82488
348	-0.58698	4.152686	0.992409	-0.79058
349	-0.43866	4.181715	0.943076	-0.78148
350	-0.3841	4.209878	0.850758	-0.77617
351	-0.29928	4.225918	0.832238	-0.75753
352	-0.24456	4.247938	0.756058	-0.74037
353	-0.17312	4.25707	0.716463	-0.732
354	-0.15351	4.276395	0.654435	-0.70731
355	-0.03761	4.281591	0.628684	-0.68
356	0.009687	4.287706	0.579478	-0.68882
357	0.045686	4.296067	0.546313	-0.65606
358	0.066189	4.299544	0.533789	-0.66209
359	0.135525	4.301236	0.496436	-0.64531
360	0.157936	4.301357	0.478345	-0.63946
361	0.239286	4.296464	0.472198	-0.6368
362	0.171562	4.302812	0.44787	-0.63033
363	0.307159	4.30258	0.410542	-0.61081
364	0.23502	4.311446	0.404348	-0.59353
365	0.281453	4.29329	0.409813	-0.59239
366	0.294125	4.289268	0.416092	-0.62376
367	0.217544	4.280902	0.428255	-0.63808
368	0.241978	4.28486	0.445864	-0.63781
369	0.180453	4.269691	0.553173	-0.61348
370	0.143868	4.25723	0.576946	-0.63606
371	0.089833	4.236022	0.662349	-0.65567
372	-0.04679	4.237702	0.607486	-0.68127
373	-0.10627	4.215352	0.640626	-0.6114
374	-0.23721	4.161325	0.784017	-0.74901
375	-0.28935	4.169083	0.862072	-0.67397
376	-0.43053	4.117453	0.980519	-0.67989

377	-0.79634	4.017469	1.149838	-0.62798
378	-0.84654	3.939688	1.275752	-0.69801
379	-1.02053	3.826519	1.366483	-0.75681
380	-1.41524	3.612879	1.531567	-0.80069
381	-1.59129	3.45142	1.650451	-0.81359
382	-2.0609	3.034641	1.940579	-0.85359
383	-2.35427	2.724608	2.023871	-0.87078
384	-2.50738	2.457186	2.172872	-0.84234
385	-2.7827	1.962771	2.349667	-0.72362
386	-2.93359	1.747353	2.357262	-0.78531
387	-3.21775	1.230112	2.41457	-0.7111
388	-3.21604	1.031889	2.499306	-0.61575
389	-3.33111	0.527436	2.521492	-0.63842
390	-3.38414	0.120325	2.558089	-0.48305
391	-3.43392	-0.09182	2.459525	-0.57277
392	-3.50069	-0.25803	2.433376	-0.43187
393	-3.45764	-0.47737	2.397157	-0.54566
394	-3.51374	-0.68741	2.320608	-0.45635
395	-3.48778	-0.94442	2.331951	-0.37927
396	-3.45414	-1.091	2.346195	-0.41103
397	-3.46831	-1.19709	2.270455	-0.38496
398	-3.42881	-1.27257	2.266615	-0.36245
399	-3.45825	-1.40685	2.200757	-0.33464
400	-3.45187	-1.42158	2.222999	-0.30587
401	-3.42235	-1.54541	2.164071	-0.2976
402	-3.43144	-1.54115	2.153386	-0.30221
403	-3.39637	-1.6521	2.138448	-0.30126
404	-3.42508	-1.66607	2.110019	-0.28847
405	-3.42058	-1.64223	2.107032	-0.25605
406	-3.40298	-1.70614	2.08562	-0.25951
407	-3.3974	-1.76503	2.029516	-0.27359
408	-3.39455	-1.78439	2.021375	-0.2301
409	-3.42059	-1.7827	1.993042	-0.2583
410	-3.40956	-1.80424	1.996748	-0.21128
411	-3.40259	-1.80673	1.989581	-0.23758
412	-3.41443	-1.7943	1.986797	-0.23139
413	-3.39525	-1.78513	2.000395	-0.18966
414	-3.44194	-1.75808	1.945969	-0.1966
415	-3.43975	-1.74607	1.946367	-0.21815
416	-3.43372	-1.75089	1.919782	-0.20887
417	-3.44877	-1.67279	1.923814	-0.21576
418	-3.43311	-1.72587	1.900248	-0.23326
419	-3.45392	-1.64479	1.933436	-0.2151

420	-3.47181	-1.64055	1.900096	-0.20555
421	-3.4553	-1.6778	1.86906	-0.22417
422	-3.4836	-1.59449	1.857798	-0.24584
423	-3.49543	-1.56402	1.863958	-0.20633
424	-3.5131	-1.49794	1.861023	-0.2034
425	-3.47591	-1.5214	1.848488	-0.21951
426	-3.54987	-1.40476	1.78911	-0.23547
427	-3.48081	-1.41135	1.848023	-0.23091
428	-3.50385	-1.31084	1.860405	-0.25149
429	-3.49453	-1.33598	1.830739	-0.21923
430	-3.53277	-1.22172	1.794952	-0.24191
431	-3.53325	-1.19682	1.784123	-0.23369
432	-3.52021	-1.11802	1.77285	-0.24393
433	-3.54454	-1.10962	1.754616	-0.24973
434	-3.55243	-0.95299	1.749187	-0.30957
435	-3.54818	-0.97515	1.734191	-0.30868
436	-3.52635	-0.88817	1.79081	-0.27502
437	-3.53279	-0.85404	1.749848	-0.28171
438	-3.53001	-0.84564	1.714822	-0.27419
439	-3.49587	-0.7123	1.711027	-0.31413
440	-3.51123	-0.74228	1.683623	-0.2791
441	-3.52316	-0.55814	1.646017	-0.30269
442	-3.46902	-0.60544	1.653133	-0.32361
443	-3.47169	-0.53077	1.626155	-0.30642
444	-3.42425	-0.51431	1.643568	-0.30061
445	-3.42406	-0.35828	1.591605	-0.30447
446	-3.47467	-0.40362	1.559589	-0.29573
447	-3.43848	-0.31423	1.581321	-0.311
448	-3.40647	-0.09683	1.54347	-0.28668
449	-3.40972	-0.1433	1.522799	-0.28453
450	-3.41709	-0.09785	1.499081	-0.30107
451	-3.4645	-0.02267	1.477929	-0.26886
452	-3.3665	-0.00579	1.475011	-0.26752
453	-3.34477	0.13634	1.497753	-0.29546
454	-3.35772	0.090287	1.428814	-0.28444
455	-3.38135	0.144613	1.445659	-0.28653

Supplementary Table 22. Explained variance ratio at each latent variable for MBPLS analysis of (*R*)-**PEA**

	CD		UV	
**Latent Variable**	**DCE**	**MeOH**	**DCE**	**MeOH**
1	0.665	0.826	0.403	0.559
2	0.214	0.071	0.301	0.239
3	0.018	0.030	0.184	0.051
4	0.027	0.005	0.023	0.005

Supplementary Table 23. Block importance for MBPLS analysis of (*R*)-**PEA**

	CD		UV	
**Latent Variable**	**DCE**	**MeOH**	**DCE**	**MeOH**
1	0.316	0.323	0.223	0.139
2	0.373	0.114	0.286	0.227
3	0.259	0.176	0.398	0.167
4	0.253	0.216	0.390	0.141

Supplementary Table 24. MBPLS explained variance in the dependent variable (concentration of (*R*)-**PEA**) at each latent variable

**Latent Variable**	**Explained Variance in Y (Concentration of (*R*)-PEA (mM))**
1	0.539
2	0.447
3	0.004
4	0.006

Supplementary Table 25. Explained variance ratio at each latent variable for MBPLS analysis of (*S*)-**PEA**

	CD		UV	
**Latent Variable**	**DCE**	**MeOH**	**DCE**	**MeOH**
1	0.749	0.899	0.356	0.622
2	0.014	0.015	0.218	0.065
3	0.042	0.010	0.042	0.018
4	0.093	0.011	0.239	0.125
5	0.055	0.007	0.079	0.057
6	0.004	0.021	0.025	0.052
7	0.014	0.005	0.017	0.047

Supplementary Table 26. Block importance for MBPLS analysis of (*S*)-**PEA**

	CD		UV	
**Latent Variable**	**DCE**	**MeOH**	**DCE**	**MeOH**
1	0.374	0.477	0.029	0.121
2	0.188	0.083	0.530	0.200
3	0.335	0.159	0.356	0.150
4	0.341	0.240	0.130	0.288
5	0.434	0.061	0.325	0.180
6	0.193	0.382	0.211	0.214
7	0.276	0.157	0.168	0.399

Supplementary Table 27. MBPLS explained variance for the dependent variable (concentration of (*S*)-**PEA** (mM)) at each latent variable

**Latent Variable**	**Explained Variance in Y (Concentration of (*S*)-PEA (mM))**
1	0.539
2	0.338
3	0.079
4	0.010
5	0.018
6	0.006
7	0.004

Supplementary Table 28. Explained variance ratio at each latent variable for MBPLS analysis of (*R*)-**PMP**

	**CD**		**UV**	
**Latent Variable**	**DCE**	**MeOH**	**DCE**	**MeOH**
1	0.748	0.877	0.366	0.637
2	0.103	0.015	0.338	0.174
3	0.047	0.037	0.164	0.048
4	0.023	0.011	0.066	0.009
5	0.026	0.008	0.012	0.012
6	0.006	0.006	0.020	0.073
7	0.016	0.014	0.012	0.033
8	0.006	0.009	0.004	0.002

Supplementary Table 29. MBPLS block importance for analysis of (*R*)-**PMP**

	CD		UV	
**Latent Variable**	**DCE**	**MeOH**	**DCE**	**MeOH**
1	0.371	0.409	0.068	0.152
2	0.164	0.026	0.494	0.315
3	0.315	0.185	0.375	0.125
4	0.129	0.185	0.554	0.132
5	0.511	0.343	0.073	0.073
6	0.250	0.281	0.126	0.343
7	0.242	0.142	0.178	0.438
8	0.310	0.537	0.109	0.043

Supplementary Table 30. Explained variance in dependent variable (concentration of (*R*)-**PMP** (mM)) at each latent variable

**Latent Variable**	**Explained Variance in Y (concentration of (*R*)-PMP (mM))**
1	0.769
2	0.184
3	0.031
4	0.011
5	0.003
6	0.001
7	0.001
8	0.000

Supplementary Table 31. Explained variance ratio at each latent variable for MBPLS analysis of (*S*)-**PMP**

	CD		UV	
**Latent Variable**	**DCE**	**MeOH**	**DCE**	**MeOH**
1	0.729	0.883	0.398	0.622
2	0.146	0.013	0.304	0.183
3	0.031	0.017	0.030	0.089

Supplementary Table 32. Block importance for analysis of (*S*)-**PMP**

	CD		UV	
**Latent Variable**	**DCE**	**MeOH**	**DCE**	**MeOH**
1	0.327	0.410	0.126	0.138
2	0.306	0.023	0.385	0.286
3	0.370	0.113	0.137	0.379

Supplementary Table 33. Explained variance in the dependent variable (concentration of (*R*)-**PMP** (mM))

Latent Variable	Explained Variance in Y (Concentration of (*R*)-PMP (mM))
1	0.820
2	0.169
3	0.009

Supplementary Table 34. Explained variance ratio at each latent variable for MBPLS analysis of (*R*)-**PPA**

	CD		UV	
Latent Variable	DCE	MeOH	DCE	MeOH
1	0.665	0.826	0.403	0.559
2	0.214	0.071	0.301	0.239
3	0.018	0.030	0.184	0.051
4	0.027	0.005	0.023	0.005

Supplementary Table 35. Block importance for analysis of (*R*)-**PPA**

	CD		UV	
Latent Variable	DCE	MeOH	DCE	MeOH
1	0.316	0.323	0.223	0.139
2	0.373	0.114	0.286	0.227
3	0.259	0.176	0.398	0.167
4	0.253	0.216	0.390	0.141
5	0.657	0.133	0.098	0.112

Supplementary Table 36. Explained variance for the dependent variable (concentration of (*R*)-**PPA** (mM)) at each latent variable

Latent Variable	Explained Variance in Y (Concentration of (*R*)-PPA (mM))
1	0.539
2	0.447
3	0.004
4	0.006
5	0.002

Supplementary Table 37. Explained variance ratio at each latent variable for MBPLS analysis of (*S*)-**PPA**

	CD		UV	
**Latent Variable**	**DCE**	**MeOH**	**DCE**	**MeOH**
1	0.749	0.899	0.356	0.622
2	0.014	0.015	0.218	0.065
3	0.042	0.010	0.042	0.018
4	0.093	0.011	0.239	0.125
5	0.055	0.007	0.079	0.057
6	0.004	0.021	0.025	0.052
7	0.014	0.005	0.017	0.047

Supplementary Table 38. Block importance for analysis of (*S*)-**PPA**

	CD		UV	
**Latent Variable**	**DCE**	**MeOH**	**DCE**	**MeOH**
1	0.374	0.477	0.029	0.121
2	0.188	0.083	0.530	0.200
3	0.335	0.159	0.356	0.150
4	0.341	0.240	0.130	0.288
5	0.434	0.061	0.325	0.180
6	0.193	0.382	0.211	0.214
7	0.276	0.157	0.168	0.399

Supplementary Table 39. Explained variance for the dependent variable (concentration of (*S*)-**PPA** (mM)) at each latent variable

Latent Variable	Explained Variance in Y (Concentration of (*S*)-PPA (mM))
1	0.539
2	0.338
3	0.079
4	0.010
5	0.018
6	0.006
7	0.004

Supplementary Table 40. Explained variance ratio at each latent variable for MBPLS analysis of (*R*)-**PGL**

	CD		UV	
**Latent Variable**	**DCE**	**MeOH**	**DCE**	**MeOH**
1	0.748	0.877	0.366	0.637
2	0.103	0.015	0.338	0.174
3	0.047	0.037	0.164	0.048
4	0.023	0.011	0.066	0.009
5	0.026	0.008	0.012	0.012
6	0.006	0.006	0.020	0.073
7	0.016	0.014	0.012	0.033
8	0.006	0.009	0.004	0.002

Supplementary Table 41. Block importance for analysis of (*R*)-**PGL**

	CD		UV	
**Latent Variable**	**DCE**	**MeOH**	**DCE**	**MeOH**
1	0.371	0.409	0.068	0.152
2	0.164	0.026	0.494	0.315
3	0.315	0.185	0.375	0.125
4	0.129	0.185	0.554	0.132
5	0.511	0.343	0.073	0.073
6	0.250	0.281	0.126	0.353
7	0.242	0.142	0.178	0.438
8	0.310	0.537	0.109	0.043

Supplementary Table 42. Explained variance for the dependent variable (concentration of (*R*)-**PGL** (mM)) at each latent variable

Latent Variable	Explained Variance in Y (Concentration of (*R*)-PGL (mM))
1	0.769
2	0.184
3	0.031
4	0.011
5	0.003
6	0.001
7	0.001
8	0.000

Supplementary Table 43. Explained variance ratio at each latent variable for MBPLS analysis of (*S*)-**PGL**

	CD		UV	
Latent Variable	DCE	MeOH	DCE	MeOH
1	0.729	0.883	0.398	0.622
2	0.146	0.013	0.304	0.183
3	0.031	0.017	0.030	0.089

Supplementary Table 44. Block importance for MBPLS analysis of (*S*)-**PGL**

	CD		UV	
**Latent Variable**	**DCE**	**MeOH**	**DCE**	**MeOH**
1	0.327	0.410	0.126	0.138
2	0.306	0.023	0.385	0.286
3	0.370	0.113	0.137	0.379

Supplementary Table 45. Explained variance for the dependent variable (concentration of (*S*)-**PGL** (mM)) at each latent variable

**Latent Variable**	**Explained Variance in Y (Concentration of (*S*)-PGL (mM))**
1	0.820
2	0.169
3	0.009

Supplementary Table 46. Comparison of actual **PEA** values vs. MBPLS prediction for both training and test sets

Entry	Actual (*R*)-PEA (mM)	Predicted (*R*)-PEA (mM)	Actual (*S*)-PEA (mM)	Predicted (*S*)-PEA (mM)
Training set 1	5.50	5.13	0.00	0.00
Training set 2	1.88	2.11	0.63	0.59
Training set 3	0.70	0.70	2.80	2.28
Training set 4	2.80	2.80	0.70	0.60
Training set 5	2.19	2.25	0.31	0.50
Training set 6	4.40	4.20	1.10	1.59
Training set 7	4.88	5.23	1.63	1.14
Training set 8	1.75	1.97	1.75	1.71
Training set 9	2.45	1.84	1.05	1.54
Training set 10	0.75	1.01	1.75	2.03
Training set 11	3.75	3.65	3.75	2.93
Training set 12	0.63	0.48	1.88	2.01
Training set 13	5.25	5.33	2.25	2.26
Training set 14	0.00	0.00	4.50	4.60
Training set 15	7.50	7.25	0.00	0.00
Training set 16	2.81	2.63	1.69	1.81
Training set 17	2.75	3.21	2.75	2.25
Training set 18	4.06	3.74	2.44	2.68
Training set 19	6.00	6.04	1.50	1.86
Training set 20	3.50	3.80	0.00	0.00
Test set 1	3.38	3.30	1.13	1.29
Test set 2	5.69	6.25	0.81	0.77
Test set 3	0.94	0.84	1.56	1.89
Test set 4	0.56	0.72	3.94	3.44
Test set 5	3.85	4.22	1.65	1.65
Test set 6	3.94	3.73	0.56	0.70

Supplementary Table 47. Comparison of actual **PMP** values vs. MBPLS prediction for both training and test sets

**Entry**	**Actual (*R*)-PMP (mM)**	**Predicted (*R*)-PMP (mM)**	**Actual (*S*)-PMP (mM)**	**Predicted (*S*)-PMP (mM)**
Training set 1	4.50	4.46	0.00	0.00
Training set 2	5.63	5.81	1.88	0.59
Training set 3	1.30	1.64	5.20	2.28
Training set 4	5.20	5.20	1.30	0.60
Training set 5	6.56	6.55	0.94	0.50
Training set 6	3.60	3.40	0.90	1.59
Training set 7	2.63	2.90	0.88	1.14
Training set 8	3.25	3.02	3.25	1.71
Training set 9	4.55	4.58	1.95	1.54
Training set 10	2.25	1.86	5.25	2.03
Training set 11	1.25	1.92	1.25	2.93
Training set 12	1.88	1.83	5.63	2.01
Training set 13	1.75	1.71	0.75	2.26
Training set 14	0.00	0.00	5.50	4.60
Training set 15	2.50	2.46	0.00	0.00
Training set 16	3.44	3.46	2.06	1.81
Training set 17	2.25	2.28	2.25	2.25
Training set 18	2.19	2.15	1.31	2.68
Training set 19	2.00	1.87	0.50	1.86
Training set 20	6.50	6.41	0.00	0.00
Test set 1	4.13	4.06	1.38	1.29
Test set 2	3.06	2.97	0.44	0.03
Test set 3	2.81	2.53	4.69	4.57
Test set 4	0.69	0.78	4.81	4.98
Test set 5	3.15	3.27	1.35	1.02
Test set 6	4.81	5.36	0.69	0.35

Supplementary Table 48. Comparison of actual **PPA** values vs. MBPLS prediction for both training and test sets

**Entry**	**Actual (1*R*, 2*S*)-PPA (mM)**	**Predicted (1*R*, 2*S*)-PPA (mM)**	**Actual (1*S*, 2*R*)-PPA (mM)**	**Predicted (1*S*, 2*R*)-PPA (mM)**
Training set 1	5.50	5.13	0.00	0.12
Training set 2	1.88	2.11	0.63	0.45
Training set 3	0.70	0.70	2.80	2.29
Training set 4	2.80	2.80	0.70	0.67
Training set 5	2.19	2.25	0.31	0.48
Training set 6	4.40	4.20	1.10	1.60
Training set 7	4.88	5.23	1.63	1.12
Training set 8	1.75	1.97	1.75	1.68
Training set 9	2.45	1.84	1.05	1.44
Training set 10	0.75	1.01	1.75	2.08
Training set 11	3.75	3.65	3.75	2.81
Training set 12	0.63	0.48	1.88	2.05
Training set 13	5.25	5.33	2.25	2.26
Training set 14	0.00	-0.04	4.50	4.66
Training set 15	7.50	7.25	0.00	0.00
Training set 16	2.81	2.63	1.69	1.81
Training set 17	2.75	3.21	2.75	2.32
Training set 18	4.06	3.74	2.44	2.64
Training set 19	6.00	6.04	1.50	1.87
Training set 20	3.50	3.80	0.00	0.00
Test set 1	3.38	3.30	1.13	1.33
Test set 2	5.69	6.25	0.81	0.69
Test set 3	0.94	0.84	1.56	2.04
Test set 4	0.56	0.72	3.94	3.49
Test set 5	3.85	4.22	1.65	1.50
Test set 6	3.94	3.73	0.56	0.69

Supplementary Table 49. Comparison of actual **PGL** values vs. MBPLS prediction for both training and test sets

**Entry**	**Actual (*R*)-PGL (mM)**	**Predicted (*R*)-PGL (mM)**	**Actual (*S*)-PGL (mM)**	**Predicted (*S*)-PGL (mM)**
Training set 1	4.50	4.47	0.00	0.20
Training set 2	5.63	5.73	1.88	1.63
Training set 3	1.30	1.58	5.20	5.48
Training set 4	5.20	5.21	1.30	1.35
Training set 5	6.56	6.48	0.94	0.84
Training set 6	3.60	3.38	0.90	0.77
Training set 7	2.63	2.89	0.88	0.75
Training set 8	3.25	3.04	3.25	3.34
Training set 9	4.55	4.57	1.95	2.05
Training set 10	2.25	2.04	5.25	4.90
Training set 11	1.25	1.88	1.25	1.52
Training set 12	1.88	1.83	5.63	5.64
Training set 13	1.75	1.69	0.75	0.71
Training set 14	0.00	0.00	5.50	5.54
Training set 15	2.50	2.50	0.00	0.25
Training set 16	3.44	3.50	2.06	2.15
Training set 17	2.25	2.28	2.25	2.18
Training set 18	2.19	2.17	1.31	1.40
Training set 19	2.00	1.83	0.50	0.27
Training set 20	6.50	6.36	0.00	0.02
Test set 1	4.13	4.07	1.38	1.30
Test set 2	3.06	2.95	0.44	0.06
Test set 3	2.81	2.52	4.69	4.68
Test set 4	0.69	0.80	4.81	4.96
Test set 5	3.15	3.29	1.35	0.95
Test set 6	4.81	5.33	0.69	0.29

Supplementary Table 50. Wavelengths selected by LASSO algorithm (unoptimized lambda) for different solvent systems

Wavelengths Selected			
CD		UV	
DCE	MeOH	DCE	MeOH
250.0	326.0	404.0	409.0
342.5	370.5	365.0	285.0
343.0	371.0	306.0	
355.5		251.0	
356.0		250.0	
393.0			
393.5			
394.0			
408.5			
409.0			
424.5			
425.0			

Supplementary Table 51. Comparison between actual **PEA** values vs. LASSO + PCR prediction for both training and test sets

**Entry**	**Actual (*R*)-PEA (mM)**	**Predicted (*R*)-PEA (mM)**	**Actual (*S*)-PEA (mM)**	**Predicted (*S*)-PEA (mM)**
Training set 1	5.50	5.33	0.00	0.00
Training set 2	1.88	2.10	0.63	0.84
Training set 3	0.70	0.34	2.80	2.41
Training set 4	2.80	2.78	0.70	0.55
Training set 5	2.19	2.24	0.31	0.49
Training set 6	4.40	4.42	1.10	1.18
Training set 7	4.88	4.91	1.63	1.59
Training set 8	1.75	1.95	1.75	1.59
Training set 9	2.45	2.25	1.05	1.13
Training set 10	0.75	0.67	1.75	1.80
Training set 11	3.75	3.99	3.75	3.42
Training set 12	0.63	0.88	1.88	1.99
Training set 13	5.25	5.54	2.25	2.30
Training set 14	0.00	0.21	4.50	4.64
Training set 15	7.50	7.24	0.00	0.00
Training set 16	2.81	2.69	1.69	1.61
Training set 17	2.75	2.56	2.75	2.57
Training set 18	4.06	3.95	2.44	2.63
Training set 19	6.00	6.08	1.50	1.63
Training set 20	3.50	3.43	0.00	0.00
Test set 1	3.38	3.27	1.13	1.22
Test set 2	5.69	6.26	0.81	1.09
Test set 3	0.94	1.18	1.56	2.22
Test set 4	0.56	0.86	3.94	3.28
Test set 5	3.85	4.00	1.65	1.34
Test set 6	3.94	3.49	0.56	0.58

Supplementary Table 52. Comparison between actual **PMP** values vs. LASSO + PCR prediction for both training and test sets

Entry	Actual (*R*)-PMP (mM)	Predicted (*R*)-PMP (mM)	Actual (*S*)-PMP (mM)	Predicted (*S*)-PMP (mM)
Training set 1	4.50	4.51	0.00	0.05
Training set 2	5.63	5.68	1.88	1.91
Training set 3	1.30	1.43	5.20	5.40
Training set 4	5.20	5.16	1.30	1.27
Training set 5	6.56	6.63	0.94	0.89
Training set 6	3.60	3.50	0.90	0.93
Training set 7	2.63	2.69	0.88	0.73
Training set 8	3.25	3.14	3.25	3.32
Training set 9	4.55	4.51	1.95	2.05
Training set 10	2.25	2.26	5.25	5.11
Training set 11	1.25	1.41	1.25	1.38
Training set 12	1.88	1.84	5.63	5.61
Training set 13	1.75	1.69	0.75	0.68
Training set 14	0.00	0.00	5.50	5.53
Training set 15	2.50	2.48	0.00	0.12
Training set 16	3.44	3.62	2.06	2.01
Training set 17	2.25	2.25	2.25	2.29
Training set 18	2.19	2.19	1.31	1.39
Training set 19	2.00	1.95	0.50	0.43
Training set 20	6.50	6.48	0.00	0.00
Test set 1	4.13	4.14	1.38	1.33
Test set 2	3.06	3.09	0.44	0.00
Test set 3	2.81	2.64	4.69	4.58
Test set 4	0.69	0.56	4.81	4.99
Test set 5	3.15	3.48	1.35	1.08
Test set 6	4.81	5.06	0.69	0.26

Supplementary Table 53. Comparison between actual **PPA** values vs. LASSO + PCR prediction for both training and test sets

Entry	Actual (1*R*, 2*S*)-PPA (mM)	Predicted (1*R*, 2*S*)-PPA (mM)	Actual (1*S*, 2*R*)-PPA (mM)	Predicted (1*S*, 2*R*)-PPA (mM)
Training set 1	5.50	5.33	0.00	0.00
Training set 2	1.88	2.01	0.63	0.84
Training set 3	0.70	0.41	2.80	2.41
Training set 4	2.80	2.80	0.70	0.55
Training set 5	2.19	2.25	0.31	0.49
Training set 6	4.40	4.36	1.10	1.18
Training set 7	4.88	5.09	1.63	1.59
Training set 8	1.75	1.57	1.75	1.59
Training set 9	2.45	2.28	1.05	1.13
Training set 10	0.75	0.76	1.75	1.80
Training set 11	3.75	3.74	3.75	3.42
Training set 12	0.63	0.81	1.88	1.99
Training set 13	5.25	5.27	2.25	2.30
Training set 14	0.00	0.10	4.50	4.64
Training set 15	7.50	7.41	0.00	0.00
Training set 16	2.81	2.69	1.69	1.61
Training set 17	2.75	2.94	2.75	2.57
Training set 18	4.06	4.03	2.44	2.63
Training set 19	6.00	5.97	1.50	1.63
Training set 20	3.50	3.52	0.00	0.00
Test set 1	3.38	3.22	1.13	1.22
Test set 2	5.69	6.35	0.81	1.09
Test set 3	0.94	0.94	1.56	2.22
Test set 4	0.56	0.65	3.94	3.28
Test set 5	3.85	4.21	1.65	1.34
Test set 6	3.94	3.70	0.56	0.58

Supplementary Table 54. Comparison between actual **PGL** values vs. LASSO + PCR prediction for both training and test sets

Entry	Actual (*R*)-PGL (mM)	Predicted (*R*)-PGL (mM)	Actual (*S*)-PGL (mM)	Predicted (*S*)-PGL (mM)
Training set 1	4.50	4.51	0.00	0.05
Training set 2	5.63	5.68	1.88	1.91
Training set 3	1.30	1.43	5.20	5.40
Training set 4	5.20	5.16	1.30	1.27
Training set 5	6.56	6.63	0.94	0.89
Training set 6	3.60	3.50	0.90	0.93
Training set 7	2.63	2.69	0.88	0.73
Training set 8	3.25	3.14	3.25	3.32
Training set 9	4.55	4.51	1.95	2.05
Training set 10	2.25	2.26	5.25	5.11
Training set 11	1.25	1.41	1.25	1.38
Training set 12	1.88	1.84	5.63	5.61
Training set 13	1.75	1.69	0.75	0.68
Training set 14	0.00	0.00	5.50	5.53
Training set 15	2.50	2.48	0.00	0.12
Training set 16	3.44	3.62	2.06	2.01
Training set 17	2.25	2.25	2.25	2.29
Training set 18	2.19	2.19	1.31	1.39
Training set 19	2.00	1.95	0.50	0.43
Training set 20	6.50	6.48	0.00	0.00
Test set 1	4.13	4.14	1.38	1.33
Test set 2	3.06	3.09	0.44	0.00
Test set 3	2.81	2.64	4.69	4.58
Test set 4	0.69	0.56	4.81	4.99
Test set 5	3.15	3.48	1.35	1.08
Test set 6	4.81	5.06	0.69	0.26

Supplementary Table 55. Comparison between actual **PEA** values vs. LASSO + PLS prediction for both training and test sets

Entry	Actual R-PEA (mM)	Predicted R-PEA (mM)	Actual S-PEA (mM)	Predicted S-PEA (mM)
Training set 1	5.50	5.33	0.00	0.02
Training set 2	1.88	2.05	0.63	0.68
Training set 3	0.70	0.40	2.80	2.48
Training set 4	2.80	2.78	0.70	0.57
Training set 5	2.19	2.23	0.31	0.45
Training set 6	4.40	4.31	1.10	1.20
Training set 7	4.88	5.05	1.63	1.52
Training set 8	1.75	1.61	1.75	1.76
Training set 9	2.45	2.36	1.05	1.22
Training set 10	0.75	0.71	1.75	1.85
Training set 11	3.75	3.78	3.75	3.37
Training set 12	0.63	0.87	1.88	1.99
Training set 13	5.25	5.31	2.25	2.33
Training set 14	0.00	0.09	4.50	4.60
Training set 15	7.50	7.38	0.00	0.00
Training set 16	2.81	2.74	1.69	1.62
Training set 17	2.75	2.89	2.75	2.52
Training set 18	4.06	3.99	2.44	2.61
Training set 19	6.00	6.02	1.50	1.67
Training set 20	3.50	3.50	0.00	0.03
Test set 1	3.38	3.21	1.13	1.25
Test set 2	5.69	6.38	0.81	1.00
Test set 3	0.94	1.13	1.56	2.15
Test set 4	0.56	0.58	3.94	3.41
Test set 5	3.85	4.08	1.65	1.34
Test set 6	3.94	3.68	0.56	0.59

Supplementary Table 56. Comparison between actual **PMP** values vs. LASSO + PLS prediction for both training and test sets

Entry	Actual (*R*)-PMP (mM)	Predicted (*R*)-PMP (mM)	Actual (*S*)-PMP (mM)	Predicted (*S*)-PMP (mM)
Training set 1	4.50	4.50	0.00	0.03
Training set 2	5.63	5.63	1.88	1.85
Training set 3	1.30	1.41	5.20	5.33
Training set 4	5.20	5.22	1.30	1.35
Training set 5	6.56	6.65	0.94	0.83
Training set 6	3.60	3.49	0.90	0.97
Training set 7	2.63	2.70	0.88	0.76
Training set 8	3.25	3.12	3.25	3.33
Training set 9	4.55	4.51	1.95	2.10
Training set 10	2.25	2.27	5.25	5.14
Training set 11	1.25	1.35	1.25	1.40
Training set 12	1.88	1.85	5.63	5.57
Training set 13	1.75	1.78	0.75	0.66
Training set 14	0.00	0.00	5.50	5.58
Training set 15	2.50	2.54	0.00	0.15
Training set 16	3.44	3.61	2.06	2.03
Training set 17	2.25	2.26	2.25	2.27
Training set 18	2.19	2.17	1.31	1.30
Training set 19	2.00	1.95	0.50	0.45
Training set 20	6.50	6.41	0.00	0.00
Test set 1	4.13	4.14	1.38	1.33
Test set 2	3.06	3.09	0.44	0.02
Test set 3	2.81	2.63	4.69	4.54
Test set 4	0.69	0.55	4.81	5.00
Test set 5	3.15	3.48	1.35	1.07
Test set 6	4.81	5.08	0.69	0.27

Supplementary Table 57. Comparison between actual **PPA** values vs. LASSO + PLS prediction for both training and test sets

Entry	Actual (1*R*, 2*S*)-PPA (mM)	Predicted (1*R*, 2*S*)-PPA (mM)	Actual (1*S*, 2*R*)-PPA (mM)	Predicted (1*S*, 2*R*)-PPA (mM)
Training set 1	5.50	5.33	0.00	0.02
Training set 2	1.88	2.05	0.63	0.68
Training set 3	0.70	0.40	2.80	2.48
Training set 4	2.80	2.78	0.70	0.57
Training set 5	2.19	2.23	0.31	0.45
Training set 6	4.40	4.31	1.10	1.20
Training set 7	4.88	5.05	1.63	1.52
Training set 8	1.75	1.61	1.75	1.76
Training set 9	2.45	2.36	1.05	1.22
Training set 10	0.75	0.71	1.75	1.85
Training set 11	3.75	3.78	3.75	3.37
Training set 12	0.63	0.87	1.88	1.99
Training set 13	5.25	5.31	2.25	2.33
Training set 14	0.00	0.09	4.50	4.60
Training set 15	7.50	7.38	0.00	0.00
Training set 16	2.81	2.74	1.69	1.62
Training set 17	2.75	2.89	2.75	2.52
Training set 18	4.06	3.99	2.44	2.61
Training set 19	6.00	6.02	1.50	1.67
Training set 20	3.50	3.50	0.00	0.03
Test set 1	3.38	3.21	1.13	1.25
Test set 2	5.69	6.38	0.81	1.00
Test set 3	0.94	1.13	1.56	2.15
Test set 4	0.56	0.58	3.94	3.41
Test set 5	3.85	4.08	1.65	1.34
Test set 6	3.94	3.68	0.56	0.59

Supplementary Table 58. Comparison between actual **PGL** values vs. LASSO + PLS prediction for both training and test sets

Entry	Actual (*R*)-PGL (mM)	Predicted (*R*)-PGL (mM)	Actual (*S*)-PGL (mM)	Predicted (*S*)-PGL (mM)
Training set 1	4.50	4.50	0.00	0.03
Training set 2	5.63	5.63	1.88	1.85
Training set 3	1.30	1.41	5.20	5.33
Training set 4	5.20	5.22	1.30	1.35
Training set 5	6.56	6.65	0.94	0.83
Training set 6	3.60	3.49	0.90	0.97
Training set 7	2.63	2.70	0.88	0.76
Training set 8	3.25	3.12	3.25	3.33
Training set 9	4.55	4.51	1.95	2.10
Training set 10	2.25	2.27	5.25	5.14
Training set 11	1.25	1.35	1.25	1.40
Training set 12	1.88	1.85	5.63	5.57
Training set 13	1.75	1.78	0.75	0.66
Training set 14	0.00	0.00	5.50	5.58
Training set 15	2.50	2.54	0.00	0.15
Training set 16	3.44	3.61	2.06	2.03
Training set 17	2.25	2.26	2.25	2.27
Training set 18	2.19	2.17	1.31	1.30
Training set 19	2.00	1.95	0.50	0.45
Training set 20	6.50	6.41	0.00	0.00
Test set 1	4.13	4.14	1.38	1.33
Test set 2	3.06	3.09	0.44	0.02
Test set 3	2.81	2.63	4.69	4.54
Test set 4	0.69	0.55	4.81	5.00
Test set 5	3.15	3.48	1.35	1.07
Test set 6	4.81	5.08	0.69	0.27

Supplementary Table 59. R^2 and RMSE for LASSO + PCR using data pre-processed by unit variance scaling, hard block scaling and soft block scaling (**PEA**)

	(*R*)-PEA				(*S*)-PEA			
**LASSO + PCR**	**Train Set R^2**	**Train Set RMSE**	**Test Set R^2**	**Test Set RMSE**	**Train Set R^2**	**Train Set RMSE**	**Test Set R^2**	**Test Set RMSE**
Unit Variance scaling	0.99	0.10	0.95	0.39	0.98	0.17	0.96	0.21
Hard block scaling	0.99	0.10	0.99	0.17	0.98	0.16	0.93	0.30
Soft block scaling	0.99	0.27	0.98	0.23	0.98	0.16	0.91	0.33

Supplementary Table 60. R^2 and RMSE for LASSO + PCR using data pre-processed by unit variance scaling, hard block scaling and soft block scaling (**PMP**)

	(*R*)-PMP				(*S*)-PMP			
**LASSO + PCR**	**Train Set R^2**	**Train Set RMSE**	**Test Set R^2**	**Test Set RMSE**	**Train Set R^2**	**Train Set RMSE**	**Test Set R^2**	**Test Set RMSE**
Unit variance scaling	0.99	0.07	0.98	0.16	0.99	0.04	0.97	0.29
Hard block scaling	0.99	0.18	0.98	0.20	0.99	0.14	0.99	0.19
Soft block scaling	0.99	0.18	0.97	0.21	0.99	0.14	0.99	0.19

Supplementary Table 61. R^2 and RMSE for LASSO + PCR using data pre-processed by unit variance scaling, hard block scaling and soft block scaling (**PPA**)

	**(1*R*, 2S)-PPA**				**(1*S*, 2*R*)-PPA**			
**LASSO + PCR**	**Train Set R^2**	**Train Set RMSE**	**Test Set R^2**	**Test Set RMSE**	**Train Set R^2**	**Train Set RMSE**	**Test Set R^2**	**Test Set RMSE**
Unit variance scaling	0.98	0.30	0.97	0.31	0.91	0.35	0.93	0.29
Hard block scaling	0.98	0.25	0.99	0.20	0.92	0.33	0.92	0.31
Soft block scaling	0.99	0.25	0.99	0.20	0.92	0.33	0.92	0.31

Supplementary Table 62. R^2 and RMSE for LASSO + PCR using data pre-processed by unit variance scaling, hard block scaling and soft block scaling (**PGL**)

	**(*R*)-PGL**				**(*S*)-PGL**			
**LASSO + PCR**	**Train Set R^2**	**Train Set RMSE**	**Test Set R^2**	**Test Set RMSE**	**Train Set R^2**	**Train Set RMSE**	**Test Set R^2**	**Test Set RMSE**
Unit variance scaling	0.99	0.07	0.99	0.13	0.99	0.04	0.97	0.29
Hard block scaling	0.99	0.18	0.97	0.21	0.99	0.14	0.99	0.19
Soft block scaling	0.99	0.18	0.97	0.21	0.99	0.14	0.99	0.19

Supplementary Table 63. Averaged R^2 and RMSE for LASSO + PCR using data pre-processed by unit variance scaling, hard block scaling and soft block scaling

	Average			
**LASSO + PCR**	**Train Set R^2**	**Train Set RMSE**	**Test Set R^2**	**Test Set RMSE**
Unit variance scaling	0.98	0.14	0.97	0.26
Hard block scaling	0.98	0.19	0.97	0.22
Soft block scaling	0.98	0.21	0.97	0.23

Supplementary Table 64. Comparison of actual **(*R*)-PEA** values vs. LASSO + PCR prediction for test sets using data pre-processed by unit variance (uv) scaling, hard block scaling and soft block scaling

Entry	Actual (*R*)-PEA (mM)	Predicted (*R*)-PEA (mM) using uv scaling	Predicted (*R*)-PEA (mM) using soft block scaling	Predicted (*R*)-PEA (mM) using hard block scaling
Test set 1	3.38	3.06	3.39	3.61
Test set 2	5.69	6.46	5.76	5.78
Test set 3	0.94	1.08	1.15	1.14
Test set 4	0.56	0.63	0.80	0.85
Test set 5	3.85	4.20	4.09	4.11
Test set 6	3.94	3.63	4.04	4.21

Supplementary Table 65. Comparison of actual **(*S*)-PEA** values vs. LASSO + PCR prediction for test sets using data pre-processed by unit variance scaling (uv), hard block scaling and soft block scaling

Entry	Actual (*S*)-PEA (mM)	Predicted (*S*)-PEA (mM) using uv scaling	Predicted (*S*)-PEA (mM) using soft block scaling	Predicted (*S*)-PEA (mM) using hard block scaling
Test set 1	1.13	1.38	1.24	1.21
Test set 2	0.81	0.68	0.83	1.01
Test set 3	1.56	1.82	2.07	2.17
Test set 4	3.94	3.89	4.15	3.99
Test set 5	1.65	1.32	1.22	1.19
Test set 6	0.56	0.65	0.42	0.40

Supplementary Table 66. Comparison of actual **(*R*)-PMP** values vs. LASSO + PCR prediction for test sets using data pre-processed by unit variance (uv) scaling, hard block scaling and soft block scaling

Entry	Actual (*R*)-PMP (mM)	Predicted (*R*)-PMP (mM) using uv scaling	Predicted (*R*)-PMP (mM) using soft block scaling	Predicted (*R*)-PMP (mM) using hard block scaling
Test set 1	4.13	4.09	4.06	4.05
Test set 2	3.06	3.07	3.17	3.14
Test set 3	2.81	2.73	2.49	2.56
Test set 4	0.69	0.85	0.45	0.46
Test set 5	3.15	3.15	3.32	3.31
Test set 6	4.81	5.08	4.90	4.94

Supplementary Table 67. Comparison of actual **(*S*)-PMP** values vs. LASSO + PCR prediction for test sets using data pre-processed by unit variance (uv) scaling, hard block scaling and soft block scaling

Entry	Actual (*S*)-PMP (mM)	Predicted (*S*)-PMP (mM) using uv scaling	Predicted (*S*)-PMP (mM) using soft block scaling	Predicted (*S*)-PMP (mM) using hard block scaling
Test set 1	1.38	1.35	1.39	1.39
Test set 2	0.44	0.06	0.31	0.32
Test set 3	4.69	4.59	4.22	4.25
Test set 4	4.81	4.99	4.85	4.83
Test set 5	1.35	0.96	1.14	1.13
Test set 6	0.69	0.28	0.39	0.40

Supplementary Table 68. Comparison of actual **(1*R*, 2*S*)-PPA** values vs. LASSO + PCR prediction for test sets using data pre-processed by unit variance (uv) scaling, hard block scaling and soft block scaling

Entry	Actual (1*R*, 2*S*)-PPA (mM)	Predicted (1*R*, 2*S*)-PPA (mM) using uv scaling	Predicted (1*R*, 2*S*)-PPA (mM) using soft block scaling	Predicted (1*R*, 2*S*)-PPA (mM) using hard block scaling
Test set 1	3.38	3.05	3.39	3.49
Test set 2	5.69	6.36	5.76	5.44
Test set 3	0.94	1.10	1.15	0.97
Test set 4	0.56	0.74	0.80	0.84
Test set 5	3.85	4.14	4.09	4.14
Test set 6	3.94	3.66	4.04	4.00

Supplementary Table 69. Comparison of actual **(1*S*, 2*R*)-PPA** values vs. LASSO + PCR prediction for test sets using data pre-processed by unit variance (uv) scaling, hard block scaling and soft block scaling

Entry	Actual (1*S*, 2*R*)-PPA (mM)	Predicted (1*S*, 2*R*)-PPA (mM) using uv scaling	Predicted (1*S*, 2*R*)-PPA (mM) using soft block scaling	Predicted (1*S*, 2*R*)-PPA (mM) using hard block scaling
Test set 1	1.13	1.45	1.24	1.21
Test set 2	0.81	0.75	0.83	1.01
Test set 3	1.56	1.92	2.07	2.17
Test set 4	3.94	3.89	4.15	3.99
Test set 5	1.65	1.18	1.22	1.19
Test set 6	0.56	0.54	0.42	0.40

Supplementary Table 70. Comparison of actual **(*R*)-PGL** values vs. LASSO + PCR prediction for test sets using data pre-processed by unit variance (uv) scaling, hard block scaling and soft block scaling

Entry	Actual (*R*)-PGL (mM)	Predicted (*R*)-PGL (mM) using uv scaling	Predicted (*R*)-PGL (mM) using soft block scaling	Predicted (*R*)-PGL (mM) using hard block scaling
Test set 1	4.13	4.09	4.06	4.05
Test set 2	3.06	3.07	3.16	3.14
Test set 3	2.81	2.73	2.49	2.56
Test set 4	0.69	0.85	0.46	0.46
Test set 5	3.15	3.15	3.31	3.31
Test set 6	4.81	5.08	4.89	4.94

Supplementary Table 71. Comparison of actual **(*S*)-PGL** values vs. LASSO + PCR prediction for test sets using data pre-processed by unit variance (uv) scaling, hard block scaling and soft block scaling

Entry	Actual (*S*)-PGL (mM)	Predicted (*S*)-PGL (mM) using uv scaling	Predicted (*S*)-PGL (mM) using soft block scaling	Predicted (*S*)-PGL (mM) using hard block scaling
Test set 1	4.13	4.09	4.06	4.05
Test set 2	3.06	3.07	3.16	3.14
Test set 3	2.81	2.73	2.49	2.56
Test set 4	0.69	0.85	0.46	0.46
Test set 5	3.15	3.15	3.31	3.31
Test set 6	4.81	5.08	4.89	4.94

Supplementary Table 72. R^2 and RMSE for MBPLS using data pre-processed by unit variance scaling, hard block scaling and soft block scaling (**PEA**)

	(*R*)-PEA				(*S*)-PEA			
**MBPLS**	**Train Set R^2**	**Train Set RMSE**	**Test Set R^2**	**Test Set RMSE**	**Train Set R^2**	**Train Set RMSE**	**Test Set R^2**	**Test Set RMSE**
Unit variance scaling	0.98	0.25	0.97	0.31	0.92	0.35	0.91	0.29
Hard block scaling	0.98	0.25	0.99	0.20	0.92	0.34	0.94	0.27
Soft block scaling	0.98	0.27	0.99	0.20	0.92	0.33	0.92	0.31

Supplementary Table 73. R^2 and RMSE for MBPLS using data pre-processed by unit variance scaling, hard block scaling and soft block scaling (**PMP**)

	**(*R*)-PMP**				**(*S*)-PMP**			
**MBPLS**	**Train Set R^2**	**Train Set RMSE**	**Test Set R^2**	**Test Set RMSE**	**Train Set R^2**	**Train Set RMSE**	**Test Set R^2**	**Test Set RMSE**
Unit variance scaling	0.99	0.18	0.96	0.26	0.99	0.17	0.98	0.27
Hard block scaling	0.99	0.18	0.97	0.23	0.92	0.34	0.94	0.27
Soft block scaling	0.99	0.18	0.97	0.21	0.99	0.14	0.99	0.19

Supplementary Table 74. R^2 and RMSE for MBPLS using data pre-processed by unit variance scaling, hard block scaling and soft block scaling (**PPA**)

	**(1*R*, 2*S*)-PPA**				**(1*S*, 2*R*)-PPA**			
**MBPLS**	**Train Set R^2**	**Train Set RMSE**	**Test Set R^2**	**Test Set RMSE**	**Train Set R^2**	**Train Set RMSE**	**Test Set R^2**	**Test Set RMSE**
Unit variance scaling	0.98	0.30	0.97	0.31	0.91	0.35	0.93	0.29
Hard block scaling	0.98	0.25	0.99	0.20	0.92	0.34	0.94	0.27
Soft block scaling	0.98	0.25	0.99	0.20	0.92	0.33	0.92	0.31

Supplementary Table 75. R^2 and RMSE for MBPLS using data pre-processed by unit variance scaling, hard block scaling and soft block scaling (**PGL**)

MBPLS	**(*R*)-PGL**				**(*S*)-PGL**			
	**Train Set R^2**	**Train Set RMSE**	**Test Set R^2**	**Test Set RMSE**	**Train Set R^2**	**Train Set RMSE**	**Test Set R^2**	**Test Set RMSE**
Unit variance scaling	0.99	0.18	0.96	0.25	0.99	0.17	0.98	0.27
Hard block scaling	0.98	0.25	0.99	0.20	0.92	0.34	0.94	0.27
Soft block scaling	0.99	0.18	0.97	0.21	0.99	0.19	0.99	0.14

Supplementary Table 76. Comparison of actual **(*R*)-PEA** values vs. MBPLS prediction for test sets using data pre-processed by unit variance (uv) scaling, hard block scaling and soft block scaling

**Entry**	**Actual (*R*)-PEA (mM)**	**Predicted (*R*)-PEA (mM) using uv scaling**	**Predicted (*R*)-PEA (mM) using soft block scaling**	**Predicted (*R*)-PEA (mM) using hard block scaling**
Test set 1	3.38	3.30	3.59	3.59
Test set 2	5.69	6.25	5.76	5.76
Test set 3	0.94	0.84	1.03	1.03
Test set 4	0.56	0.72	0.76	0.76
Test set 5	3.85	4.22	4.15	4.15
Test set 6	3.94	3.73	4.14	4.14

Supplementary Table 77. Comparison of actual **(*S*)-PEA** values vs. MBPLS prediction for test sets using data pre-processed by unit variance (uv) scaling, hard block scaling and soft block scaling

Entry	Actual (*S*)-PEA (mM)	Predicted (*S*)-PEA (mM) using uv scaling	Predicted (*S*)-PEA (mM) using soft block scaling	Predicted (*S*)-PEA (mM) using hard block scaling
Test set 1	1.13	1.29	1.10	1.07
Test set 2	0.81	0.77	0.97	0.91
Test set 3	1.56	1.89	2.27	2.17
Test set 4	3.94	3.44	3.82	3.78
Test set 5	1.65	1.65	1.51	1.58
Test set 6	0.56	0.70	0.42	0.41

Supplementary Table 78. Comparison of actual **(*R*)-PMP** values vs. MBPLS prediction for test sets using data pre-processed by unit variance (uv) scaling, hard block scaling and soft block scaling

Entry	Actual (*R*)-PMP (mM)	Predicted (*R*)-PMP (mM) using uv scaling	Predicted (*R*)-PMP (mM) using soft block scaling	Predicted (*R*)-PMP (mM) using hard block scaling
Test set 1	4.13	4.06	3.97	3.95
Test set 2	3.06	2.97	2.96	2.95
Test set 3	2.81	2.53	2.36	2.33
Test set 4	0.69	0.78	0.65	0.62
Test set 5	3.15	3.27	3.14	3.20
Test set 6	4.81	5.36	4.99	5.00

Supplementary Table 79. Comparison of actual **(*S*)-PMP** values vs. MBPLS prediction for test sets using data pre-processed by unit variance (uv) scaling, hard block scaling and soft block scaling

Entry	Actual (*S*)-PMP (mM)	Predicted (*S*)-PMP (mM) using unit variance scaling	Predicted (*S*)-PMP (mM) using soft block scaling	Predicted (*S*)-PMP (mM) using hard block scaling
Test set 1	1.38	1.40	1.38	1.38
Test set 2	0.44	0.30	0.35	0.35
Test set 3	4.69	4.27	4.33	4.33
Test set 4	4.81	4.84	4.73	4.73
Test set 5	1.35	1.25	1.22	1.22
Test set 6	0.69	0.53	0.43	0.43

Supplementary Table 80. Comparison of actual **(1*R*, 2*S*)-PPA** values vs. MBPLS prediction for test sets using data pre-processed by unit variance (uv) scaling, hard block scaling and soft block scaling

Entry	Actual (1*R*, 2*S*)-PPA (mM)	Predicted (1*R*, 2*S*)-PPA (mM) using uv scaling	Predicted (1*R*, 2*S*)-PPA (mM) using soft block scaling	Predicted (1*R*, 2*S*)-PPA (mM) using hard block scaling
Test set 1	3.38	3.30	3.57	3.57
Test set 2	5.69	6.25	5.83	5.83
Test set 3	0.94	0.84	0.97	0.97
Test set 4	0.56	0.72	0.80	0.80
Test set 5	3.85	4.22	4.15	4.15
Test set 6	3.94	3.73	4.09	4.09

Supplementary Table 81. Comparison of actual **(1*R*, 2*S*)-PPA** values vs. MBPLS prediction for test sets using data pre-processed by unit variance (uv) scaling, hard block scaling and soft block scaling

Entry	Actual (1*S*, 2*R*)-PPA (mM)	Predicted (1*S*, 2*R*)-PPA (mM) using uv scaling	Predicted (1*S*, 2*R*)-PPA (mM) using soft block scaling	Predicted (1*S*, 2*R*)-PPA (mM) using hard block scaling
Test set 1	1.13	1.33	1.10	1.10
Test set 2	0.81	0.69	0.97	0.97
Test set 3	1.56	2.04	2.27	2.27
Test set 4	3.94	3.49	3.82	3.82
Test set 5	1.65	1.50	1.51	1.51
Test set 6	0.56	0.69	0.42	0.42

Supplementary Table 82. Comparison of actual **(*R*)-PGL** values vs. MBPLS prediction for test sets using data pre-processed by unit variance (uv) scaling, hard block scaling and soft block scaling

Entry	Actual (*R*)-PGL (mM)	Predicted (*R*)-PGL (mM) using uv scaling	Predicted (*R*)-PGL (mM) using soft block scaling	Predicted (*R*)-PGL (mM) using hard block scaling
Test set 1	4.13	4.07	3.97	3.97
Test set 2	3.06	2.95	2.96	2.96
Test set 3	2.81	2.52	2.36	2.36
Test set 4	0.69	0.80	0.65	0.65
Test set 5	3.15	3.29	3.14	3.14
Test set 6	4.81	5.33	4.99	4.99

Supplementary Table 83. Comparison of actual **(*S*)-PGL** values vs. MBPLS prediction for test sets using data pre-processed by unit variance (uv) scaling, hard block scaling and soft block scaling

Entry	Actual (*S*)-PGL (mM)	Predicted (*S*)-PGL (mM) using uv scaling	Predicted (*S*)-PGL (mM) using soft block scaling	Predicted (*S*)-PGL (mM) using hard block scaling
Test set 1	1.38	1.30	1.38	1.38
Test set 2	0.44	0.06	0.35	0.35
Test set 3	4.69	4.68	4.33	4.33
Test set 4	4.81	4.96	4.73	4.73
Test set 5	1.35	0.95	1.22	1.22
Test set 6	0.69	0.29	0.43	0.43

Supplementary Table 84. Averaged R^2 and RMSE for MBPLS using data pre-processed by unit variance scaling, hard block scaling and soft block scaling

	Average			
MBPLS	Train Set R^2	Train Set RMSE	Test Set R^2	Test Set RMSE
Unit variance scaling	0.97	0.24	0.96	0.28
Hard block scaling	0.95	0.30	0.97	0.24
Soft block scaling	0.97	0.23	0.97	0.22

Supplementary Table 85. Explained variance ratio at each latent variable for MBPLS analysis of **(*R*)-PEA** using data pre-processed with unit variance scaling

Unit Variance Scaling ((*R*)-PEA)				
**LV**	**Explained variance for CD data in DCE**	**Explained variance for CD data in MeOH**	**Explained variance for UV data in DCE**	**Explained variance for UV data in MeOH**
1	0.729	0.883	0.398	0.622
2	0.146	0.013	0.304	0.183
3	0.031	0.017	0.03	0.089
4	0.023	0.024	151	0.035

Supplementary Table 86. Explained variance ratio at each latent variable for MBPLS analysis of **(*R*)-PEA** using data pre-processed with block scaling

Block scaling ((*R*)-PEA)				
**LV**	**Explained variance for CD data in DCE**	**Explained variance for CD data in MeOH**	**Explained variance for UV data in DCE**	**Explained variance for UV data in MeOH**
1	0.665	0.826	0.403	0.559
2	0.214	0.071	0.301	0.239
3	0.018	0.03	0.184	0.051
4	0.027	0.005	0.023	0.005
5	0.036	0.005	0.012	0.016
6	0.003	0.016	0.012	0.023

Supplementary Table 87. Block importance for MBPLS analysis of **(*R*)-PEA** using data pre-processed with unit variance scaling

Unit Variance Scaling ((*R*)-PEA)				
**LV**	**Block importance for CD data in DCE**	**Block importance for CD data in MeOH**	**Block importance for UV data in DCE**	**Block importance for UV data in MeOH**
1	0.327	0.410	0.126	0.138
2	0.306	0.023	0.385	0.286
3	0.370	0.113	0.137	0.379
4	0.124	0.203	0.464	0.209

Supplementary Table 88. Block importance for MBPLS analysis of **(*R*)-PEA** using data pre-processed with block scaling

**Block scaling ((*R*)-PEA)**				
**LV**	**Block importance for CD data in DCE**	**Block importance for CD data in MeOH**	**Block importance for UV data in DCE**	**Block importance for UV data in MeOH**
1	0.316	0.323	0.223	0.139
2	0.373	0.114	0.286	0.227
3	0.259	0.176	0.398	0.167
4	0.253	0.216	0.39	0.141
5	0.657	0.133	0.098	0.112
6	0.230	0.389	0.258	0.123

Supplementary Table 89. Explained variance ratio at each latent variable for MBPLS analysis of **(*S*)-PEA** using data pre-processed with unit variance scaling

**Unit Variance Scaling ((*S*)-PEA)**				
**LV**	**Explained variance for CD data in DCE**	**Explained variance for CD data in MeOH**	**Explained variance for UV data in DCE**	**Explained variance for UV data in MeOH**
1	0.749	0.899	0.356	0.622
2	0.014	0.015	0.218	0.065
3	0.042	0.010	0.042	0.018
4	0.093	0.011	0.239	0.125
5	0.055	0.007	0.079	0.057
6	0.004	0.021	0.025	0.052
7	0.014	0.005	0.017	0.047

Supplementary Table 90. Block importance for MBPLS analysis of **(*S*)-PEA** using data pre-processed with unit variance scaling

**Unit Variance Scaling ((*S*)-PEA)**				
**LV**	**Block importance for CD data in DCE**	**Block importance for CD data in MeOH**	**Block importance for UV data in DCE**	**Block importance for UV data in MeOH**
1	0.374	0.477	0.029	0.121
2	0.188	0.083	0.530	0.200
3	0.335	0.159	0.356	0.150
4	0.341	0.240	0.130	0.288
5	0.434	0.061	0.325	0.180
6	0.193	0.382	0.211	0.214
7	0.276	0.157	0.168	0.399

Supplementary Table 91. Explained variance ratio at each latent variable for MBPLS analysis of **(*S*)-PEA** using data pre-processed with block scaling

**Block Scaling ((*S*)-PEA)**				
**LV**	**Explained variance for CD data in DCE**	**Explained variance for CD data in MeOH**	**Explained variance for UV data in DCE**	**Explained variance for UV data in MeOH**
1	0.749	0.899	0.356	0.622
2	0.014	0.015	0.218	0.065
3	0.042	0.010	0.042	0.018
4	0.093	0.011	0.239	0.125
5	0.055	0.007	0.079	0.057
6	0.004	0.021	0.025	0.052
7	0.014	0.005	0.017	0.047
8	0.004	0.008	0.005	0.005
9	0.003	0.007	0.003	0.000

Supplementary Table 92. Block importance for MBPLS analysis of **(*S*)-PEA** using data pre-processed with block scaling

**Block Scaling ((*S*)-PEA)**				
**LV**	**Block importance for CD data in DCE**	**Block importance for CD data in MeOH**	**Block importance for UV data in DCE**	**Block importance for UV data in MeOH**
1	0.374	0.477	0.029	0.121
2	0.188	0.083	0.530	0.200
3	0.335	0.159	0.356	0.150
4	0.341	0.240	0.130	0.288
5	0.434	0.061	0.325	0.180
6	0.193	0.382	0.211	0.214
7	0.276	0.157	0.168	0.399
8	0.240	0.414	0.152	0.193
9	0.254	0.617	0.105	0.024

Supplementary Table 93. Explained variance ratio at each latent variable for MBPLS analysis of **(*R*)-PMP** using data pre-processed with unit variance scaling

**Unit Variance Scaling ((*R*)-PMP)**				
**LV**	**Explained variance for CD data in DCE**	**Explained variance for CD data in MeOH**	**Explained variance for UV data in DCE**	**Explained variance for UV data in MeOH**
1	0.748	0.877	0.366	0.637
2	0.103	0.015	0.338	0.174
3	0.047	0.038	0.164	0.048
4	0.023	0.011	0.066	0.009
5	0.026	0.008	0.012	0.012
6	0.006	0.006	0.020	0.073
7	0.016	0.014	0.012	0.033
8	0.006	0.009	0.004	0.002

Supplementary Table 94. Explained variance ratio at each latent variable for MBPLS analysis of **(*R*)-PMP** using data pre-processed with block scaling

**Block Scaling ((*R*)-PMP)**				
**LV**	**Explained variance for CD data in DCE**	**Explained variance for CD data in MeOH**	**Explained variance for UV data in DCE**	**Explained variance for UV data in MeOH**
1	0.748	0.877	0.366	0.637
2	0.103	0.015	0.338	0.174
3	0.047	0.038	0.164	0.048
4	0.023	0.011	0.066	0.009
5	0.026	0.008	0.012	0.012
6	0.006	0.006	0.020	0.073
7	0.016	0.014	0.012	0.033
8	0.006	0.009	0.004	0.002
9	0.004	0.005	0.001	0.000
10	0.003	0.002	0.001	0.000

Supplementary Table 95. Block importance for MBPLS analysis of **(*R*)-PMP** using data pre-processed with unit variance scaling

**Unit Variance Scaling ((*R*)-PMP)**				
**LV**	**Block importance for CD data in DCE**	**Block importance for CD data in MeOH**	**Block importance for UV data in DCE**	**Block importance for UV data in MeOH**
1	0.371	0.477	0.029	0.121
2	0.164	0.083	0.530	0.200
3	0.315	0.159	0.356	0.150
4	0.129	0.240	0.130	0.288
5	0.511	0.061	0.325	0.180
6	0.250	0.382	0.211	0.214
7	0.242	0.157	0.168	0.399
8	0.310	0.537	0.109	0.043

Supplementary Table 96. Block importance for MBPLS analysis of **(*R*)-PMP** using data pre-processed with block scaling

**Block Scaling ((*R*)-PMP)**				
**LV**	**Block importance for CD data in DCE**	**Block importance for CD data in MeOH**	**Block importance for UV data in DCE**	**Block importance for UV data in MeOH**
1	0.371	0.409	0.068	0.152
2	0.164	0.026	0.494	0.315
3	0.315	0.185	0.375	0.125
4	0.129	0.185	0.554	0.132
5	0.511	0.343	0.073	0.073
6	0.250	0.281	0.126	0.343
7	0.242	0.142	0.178	0.438
8	0.310	0.537	0.109	0.043
9	0.361	0.561	0.044	0.034
10	0.581	0.326	0.069	0.023

Supplementary Table 97. Explained variance ratio at each latent variable for MBPLS analysis of **(*S*)-PMP** using data pre-processed with unit variance scaling

**Unit Variance Scaling ((*S*)-PMP)**				
**LV**	**Explained variance for CD data in DCE**	**Explained variance for CD data in MeOH**	**Explained variance for UV data in DCE**	**Explained variance for UV data in MeOH**
1	0.729	0.883	0.398	0.622
2	0.146	0.013	0.304	0.183
3	0.031	0.017	0.030	0.089
4	0.023	0.024	0.151	0.035
5	0.003	0.011	0.077	0.025

Supplementary Table 98. Explained variance ratio at each latent variable for MBPLS analysis of **(*S*)-PMP** using data pre-processed with block scaling

**Block Scaling ((*S*)-PMP)**				
**LV**	**Explained variance for CD data in DCE**	**Explained variance for CD data in MeOH**	**Explained variance for UV data in DCE**	**Explained variance for UV data in MeOH**
1	0.729	0.883	0.398	0.622
2	0.146	0.013	0.304	0.183
3	0.031	0.017	0.030	0.089
4	0.023	0.024	0.151	0.035

Supplementary Table 99. Block importance for MBPLS analysis of **(*S*)-PMP** using data pre-processed with unit variance scaling

**Unit Variance Scaling ((*S*)-PMP)**				
**LV**	**Block importance for CD data in DCE**	**Block importance for CD data in MeOH**	**Block importance for UV data in DCE**	**Block importance for UV data in MeOH**
1	0.327	0.410	0.126	0.138
2	0.306	0.023	0.385	0.286
3	0.370	0.113	0.137	0.379
4	0.124	0.203	0.464	0.209
5	0.195	0.223	0.523	0.059

Supplementary Table 100. Block importance for MBPLS analysis of **(*S*)-PMP** using data pre-processed with block scaling

**Block Scaling ((*S*)-PMP)**				
**LV**	**Block importance for CD data in DCE**	**Block importance for CD data in MeOH**	**Block importance for UV data in DCE**	**Block importance for UV data in MeOH**
1	0.327	0.410	0.126	0.138
2	0.306	0.023	0.385	0.286
3	0.370	0.113	0.137	0.379
4	0.124	0.203	0.464	0.209

Supplementary Table 101. Explained variance ratio at each latent variable for MBPLS analysis of **(1*R*,2*S*)-PPA** using data pre-processed with unit variance scaling

**Unit Variance Scaling ((1*R*,2*S*)-PPA)**				
**LV**	**Explained variance for CD data in DCE**	**Explained variance for CD data in MeOH**	**Explained variance for UV data in DCE**	**Explained variance for UV data in MeOH**
1	0.665	0.826	0.403	0.559
2	0.214	0.071	0.301	0.239
3	0.018	0.030	0.184	0.051
4	0.027	0.005	0.023	0.005
5	0.036	0.005	0.012	0.016

Supplementary Table 102. Explained variance ratio at each latent variable for MBPLS analysis of **(1*R*,2*S*)-PPA** using data pre-processed with block scaling

**Block Scaling ((1*R*,2*S*)-PPA)**				
**LV**	**Explained variance for CD data in DCE**	**Explained variance for CD data in MeOH**	**Explained variance for UV data in DCE**	**Explained variance for UV data in MeOH**
1	0.665	0.826	0.403	0.559
2	0.214	0.071	0.301	0.239
3	0.018	0.030	0.184	0.051
4	0.027	0.005	0.023	0.005
5	0.036	0.005	0.012	0.016

Supplementary Table 103. Block importance for MBPLS analysis of **(1*R*,2*S*)-PPA** using data pre-processed with unit variance scaling

**Unit Variance Scaling ((1*R*,2*S*)-PPA)**				
**LV**	**Block importance for CD data in DCE**	**Block importance for CD data in MeOH**	**Block importance for UV data in DCE**	**Block importance for UV data in MeOH**
1	0.316	0.323	0.223	0.139
2	0.373	0.114	0.286	0.227
3	0.259	0.176	0.398	0.167
4	0.253	0.216	0.390	0.141
5	0.657	0.133	0.098	0.112

Supplementary Table 104. Block importance for MBPLS analysis of **(1*R*,2*S*)-PPA** using data pre-processed with block scaling

**Block Scaling ((1*R*,2*S*)-PPA)**				
**LV**	**Block importance for CD data in DCE**	**Block importance for CD data in MeOH**	**Block importance for UV data in DCE**	**Block importance for UV data in MeOH**
1	0.316	0.323	0.223	0.139
2	0.373	0.114	0.286	0.227
3	0.259	0.176	0.398	0.167
4	0.253	0.216	0.390	0.141
5	0.657	0.133	0.098	0.112

Supplementary Table 105. Explained variance ratio at each latent variable for MBPLS analysis of **(1*S*,2*R*)-PPA** using data pre-processed with unit variance scaling

**Unit Variance Scaling ((1*S*,2*R*)-PPA)**				
**LV**	**Explained variance for CD data in DCE**	**Explained variance for CD data in MeOH**	**Explained variance for UV data in DCE**	**Explained variance for UV data in MeOH**
1	0.749	0.826	0.403	0.559
2	0.014	0.071	0.301	0.239
3	0.042	0.030	0.184	0.051
4	0.093	0.005	0.023	0.005
5	0.055	0.005	0.012	0.016
6	0.004	0.021	0.025	0.052
7	0.014	0.005	0.017	0.047

Supplementary Table 106. Explained variance ratio at each latent variable for MBPLS analysis of **(1*S*,2*R*)-PPA** using data pre-processed with block scaling

**Block Scaling ((1*S*,2*R*)-PPA)**				
**LV**	**Explained variance for CD data in DCE**	**Explained variance for CD data in MeOH**	**Explained variance for UV data in DCE**	**Explained variance for UV data in MeOH**
1	0.665	0.826	0.403	0.559
2	0.214	0.071	0.301	0.239
3	0.018	0.030	0.184	0.051
4	0.027	0.005	0.023	0.005
5	0.036	0.005	0.012	0.016

Supplementary Table 107. Block importance for MBPLS analysis of **(1*S*,2*R*)-PPA** using data pre-processed with unit variance scaling

**Unit Variance Scaling ((1*S*,2*R*)-PPA)**				
**LV**	**Block importance for CD data in DCE**	**Block importance for CD data in MeOH**	**Block importance for UV data in DCE**	**Block importance for UV data in MeOH**
1	0.374	0.477	0.029	0.121
2	0.188	0.083	0.530	0.200
3	0.335	0.159	0.356	0.150
4	0.341	0.240	0.130	0.288
5	0.434	0.061	0.325	0.180
6	0.193	0.382	0.211	0.214
7	0.276	0.157	0.168	0.399

Supplementary Table 108. Block importance for MBPLS analysis of **(1*S*,2*R*)-PPA** using data pre-processed with block scaling

**Block Scaling ((1*S*,2*R*)-PPA)**				
**LV**	**Block importance for CD data in DCE**	**Block importance for CD data in MeOH**	**Block importance for UV data in DCE**	**Block importance for UV data in MeOH**
1	0.374	0.477	0.029	0.121
2	0.188	0.083	0.530	0.200
3	0.335	0.159	0.356	0.150
4	0.341	0.240	0.130	0.288
5	0.434	0.061	0.325	0.180
6	0.193	0.382	0.211	0.214
7	0.276	0.157	0.168	0.399
8	0.240	0.414	0.152	0.193

Supplementary Table 109. Explained variance ratio at each latent variable for MBPLS analysis of **(*R*)-PGL** using data pre-processed with unit variance scaling

**Unit Variance Scaling ((*R*)-PGL)**				
**LV**	**Explained variance for CD data in DCE**	**Explained variance for CD data in MeOH**	**Explained variance for UV data in DCE**	**Explained variance for UV data in MeOH**
1	0.748	0.877	0.366	0.637
2	0.103	0.015	0.338	0.174
3	0.047	0.038	0.164	0.048
4	0.023	0.011	0.066	0.009
5	0.026	0.008	0.012	0.012

Supplementary Table 110. Explained variance ratio at each latent variable for MBPLS analysis of **(*R*)-PGL** using data pre-processed with block scaling

**Block Scaling ((*R*)-PGL)**				
**LV**	**Explained variance for CD data in DCE**	**Explained variance for CD data in MeOH**	**Explained variance for UV data in DCE**	**Explained variance for UV data in MeOH**
1	0.748	0.877	0.366	0.637
2	0.103	0.015	0.338	0.174
3	0.047	0.038	0.164	0.048
4	0.023	0.011	0.066	0.009
5	0.026	0.008	0.012	0.012
6	0.006	0.006	0.020	0.073
7	0.016	0.014	0.012	0.033
8	0.006	0.009	0.004	0.002
9	0.004	0.005	0.001	0.000

Supplementary Table 111. Block importance for MBPLS analysis of **(*R*)-PGL** using data pre-processed with unit variance scaling

**Unit Variance Scaling ((*R*)-PGL)**				
**LV**	**Block importance for CD data in DCE**	**Block importance for CD data in MeOH**	**Block importance for UV data in DCE**	**Block importance for UV data in MeOH**
1	0.371	0.409	0.068	0.152
2	0.164	0.026	0.494	0.315
3	0.315	0.185	0.375	0.125
4	0.129	0.185	0.544	0.132
5	0.511	0.343	0.073	0.073

Supplementary Table 112. Block importance for MBPLS analysis of **(*R*)-PGL** using data pre-processed with unit variance scaling

**Unit Variance Scaling ((*R*)-PGL)**				
**LV**	**Block importance for CD data in DCE**	**Block importance for CD data in MeOH**	**Block importance for UV data in DCE**	**Block importance for UV data in MeOH**
1	0.371	0.409	0.068	0.152
2	0.164	0.026	0.494	0.315
3	0.315	0.185	0.375	0.125
4	0.129	0.185	0.544	0.132
5	0.511	0.343	0.073	0.073
6	0.250	0.281	0.126	0.343
7	0.242	0.142	0.178	0.438
8	0.310	0.537	0.109	0.043
9	0.361	0.561	0.044	0.034

Supplementary Table 113. Explained variance ratio at each latent variable for MBPLS analysis of **(*S*)-PGL** using data pre-processed with unit variance scaling

**Unit Variance Scaling ((*S*)-PGL)**				
**LV**	**Explained variance for CD data in DCE**	**Explained variance for CD data in MeOH**	**Explained variance for UV data in DCE**	**Explained variance for UV data in MeOH**
1	0.729	0.883	0.398	0.622
2	0.146	0.013	0.304	0.183
3	0.031	0.017	0.030	0.089
4	0.023	0.024	0.151	0.035
5	0.003	0.011	0.077	0.025

Supplementary Table 114. Explained variance ratio at each latent variable for MBPLS analysis of **(*S*)-PGL** using data pre-processed with block scaling

**Block Scaling ((*S*)-PGL)**				
**LV**	**Explained variance for CD data in DCE**	**Explained variance for CD data in MeOH**	**Explained variance for UV data in DCE**	**Explained variance for UV data in MeOH**
1	0.729	0.883	0.398	0.622
2	0.146	0.013	0.304	0.183
3	0.031	0.017	0.030	0.089
4	0.023	0.024	0.151	0.035

Supplementary Table 115. Block importance for MBPLS analysis of **(*S*)-PGL** using data pre-processed with unit variance scaling

**Unit Variance Scaling ((*S*)-PGL)**				
**LV**	**Block importance for CD data in DCE**	**Block importance for CD data in MeOH**	**Block importance for UV data in DCE**	**Block importance for UV data in MeOH**
1	0.327	0.410	0.126	0.138
2	0.306	0.306	0.385	0.286
3	0.370	0.370	0.137	0.379
4	0.124	0.124	0.464	0.209
5	0.195	0.195	0.523	0.059

Supplementary Table 116. Block importance for MBPLS analysis of **(*S*)-PGL** using data pre-processed with block scaling

**Block Scaling ((*S*)-PGL)**				
**LV**	**Block importance for CD data in DCE**	**Block importance for CD data in MeOH**	**Block importance for UV data in DCE**	**Block importance for UV data in MeOH**
1	0.327	0.410	0.126	0.138
2	0.306	0.306	0.385	0.286
3	0.370	0.370	0.137	0.379
4	0.124	0.124	0.464	0.209

Supplementary Table 117. Optimum Lambda value for each analyte

Analyte	Optimum Lambda Value
(*R*)-**PEA**	0.00012
(*S*)-**PEA**	0.00104
(*R*)-**PMP**	0.00036
(*S*)-**PMP**	0.31623
(1*R*,2*S*)-**PPA**	0.00006
(1*S*,2*R*)-**PPA**	0.00104
(*R*)-**PGL**	0.00036
(*S*)-**PGL**	0.31623

Supplementary Table 118. Number of variables with regression coefficient significantly different from zero for each analyte

Analyte	Number of Variables
(*R*)-**PEA**	91
(*S*)-**PEA**	40
(*R*)-**PMP**	70
(*S*)-**PMP**	6
(1*R*,2*S*)-**PPA**	179
(1*S*,2*R*)-**PPA**	40
(*R*)-**PGL**	70
(*S*)-**PGL**	6

Supplementary Table 119. R^2 and RMSE for LASSO + PCR using non-optimized vs. optimized lambda value (**PEA**)

	(*R*)-PEA				(*S*)-PEA			
**LASSO + PCR**	**Train Set R^2**	**Train Set RMSE**	**Test Set R^2**	**Test Set RMSE**	**Train Set R^2**	**Train Set RMSE**	**Test Set R^2**	**Test Set RMSE**
Before optimization (Lambda= 0.01)	0.99	0.19	0.96	0.34	0.98	0.17	0.86	0.42
After optimization (Lambda= 0.00012)	0.99	0.10	0.95	0.39	0.98	0.16	0.94	0.27

Supplementary Table 120. R^2 and RMSE for LASSO + PCR using non-optimized vs. optimized lambda value (**PMP**)

	(*R*)-PMP				(*S*)-PMP			
**LASSO + PCR**	**Train Set R^2**	**Train Set RMSE**	**Test Set R^2**	**Test Set RMSE**	**Train Set R^2**	**Train Set RMSE**	**Test Set R^2**	**Test Set RMSE**
Before optimization (Lambda= 0.01)	0.99	0.08	0.98	0.20	0.99	0.09	0.97	0.28
After optimization (Lambda= 0.00012)	0.99	0.07	0.99	0.13	0.99	0.04	0.97	0.29

Supplementary Table 121. R^2 and RMSE for LASSO + PCR using non-optimized vs. optimized lambda value (**PPA**)

	**(1*R*,2*S*)-PPA**				**(1*S*,2*R*)-PPA**			
**LASSO + PCR**	**Train Set R^2**	**Train Set RMSE**	**Test Set R^2**	**Test Set RMSE**	**Train Set R^2**	**Train Set RMSE**	**Test Set R^2**	**Test Set RMSE**
Before optimization (Lambda= 0.01)	0.99	0.13	0.96	0.33	0.98	0.17	0.86	0.41
After optimization (Lambda= 0.00012)	0.99	0.14	0.96	0.36	0.98	0.15	0.90	0.35

Supplementary Table 122. R^2 and RMSE for LASSO + PCR using non-optimized vs. optimized lambda value (**PGL**)

	**(*R*)-PGL**				**(*S*)-PGL**			
**LASSO + PCR**	**Train Set R^2**	**Train Set RMSE**	**Test Set R^2**	**Test Set RMSE**	**Train Set R^2**	**Train Set RMSE**	**Test Set R^2**	**Test Set RMSE**
Before optimization (Lambda= 0.01)	0.99	0.08	0.98	0.19	0.99	0.09	0.97	0.29
After optimization (Lambda= 0.00012)	0.99	0.07	0.99	0.13	0.99	0.04	0.97	0.29

Supplementary Table 123. R^2 and RMSE for LASSO + PLS using non-optimized vs. optimized lambda value (**PEA**)

	**(*R*)-PEA**				**(*S*)-PEA**			
**LASSO + PLS**	**Train Set R^2**	**Train Set RMSE**	**Test Set R^2**	**Test Set RMSE**	**Train Set R^2**	**Train Set RMSE**	**Test Set R^2**	**Test Set RMSE**
Before optimization (Lambda= 0.01)	0.99	0.13	0.96	0.33	0.98	0.16	0.90	0.36
After optimization (Lambda= 0.00012)	0.99	0.09	0.96	0.35	0.98	0.15	0.95	0.25

Supplementary Table 124. R^2 and RMSE for LASSO + PLS using non-optimized vs. optimized lambda value (**PMP**)

	**(*R*)-PMP**				**(*S*)-PMP**			
**LASSO + PLS**	**Train Set R^2**	**Train Set RMSE**	**Test Set R^2**	**Test Set RMSE**	**Train Set R^2**	**Train Set RMSE**	**Test Set R^2**	**Test Set RMSE**
Before optimization (Lambda= 0.01)	0.99	0.08	0.98	0.20	0.99	0.09	0.97	0.28
After optimization (Lambda= 0.00012)	0.99	0.05	0.98	0.17	0.99	0.03	0.97	0.29

Supplementary Table 125. R^2 and RMSE for LASSO + PLS using non-optimized vs. optimized lambda value (**PPA**)

	**(1*R*,2*S*)-PPA**				**(1*S*,2*R*)-PPA**			
**LASSO + PLS**	**Train Set R^2**	**Train Set RMSE**	**Test Set R^2**	**Test Set RMSE**	**Train Set R^2**	**Train Set RMSE**	**Test Set R^2**	**Test Set RMSE**
Before optimization (Lambda= 0.01)	0.99	0.13	0.96	0.33	0.98	0.16	0.90	0.36
After optimization (Lambda= 0.00012)	0.99	0.09	0.96	0.35	0.98	0.15	0.95	0.24

Supplementary Table 126. R^2 and RMSE for LASSO + PLS using non-optimized vs. optimized lambda value (**PGL**)

	**(*R*)-PGL**				**(*S*)-PGL**			
**LASSO + PLS**	**Train Set R^2**	**Train Set RMSE**	**Test Set R^2**	**Test Set RMSE**	**Train Set R^2**	**Train Set RMSE**	**Test Set R^2**	**Test Set RMSE**
Before optimization (Lambda= 0.01)	0.99	0.08	0.98	0.19	0.99	0.09	0.97	0.28
After optimization (Lambda= 0.00012)	0.99	0.05	0.98	0.17	0.99	0.03	0.97	0.29

Supplementary Table 127. Comparison between actual **PEA** values vs. LASSO + PCR prediction for both training and test sets

LASSO + PCR									
**Entry**		**(*R*)-PMP** (mM)	**(S)-PMP** (mM)	**(*R*)-PEA** (mM)	**(*S*)-PEA** (mM)	**(1*R*,2*S*)-PPA** (mM)	**(1*S*,2*R*)-PPA** (mM)	**(*R*)-PGL** (mM)	**(*S*)-PGL** (mM)
1	Actual	4.13	1.38	3.38	1.13	3.38	1.13	4.13	1.38
	Predicted	4.09	1.00	3.06	1.33	3.21	1.43	4.10	1.06
2	Actual	3.06	0.44	5.69	0.81	5.69	0.81	3.06	0.44
	Predicted	3.07	0.03	6.46	0.32	7.08	0.34	3.00	0.02
3	Actual	2.81	4.69	0.94	1.56	0.94	1.56	2.81	4.69
	Predicted	2.71	4.68	1.08	1.78	1.63	1.72	2.60	4.64
4	Actual	0.69	4.81	0.56	3.94	0.56	3.94	0.69	4.81
	Predicted	0.65	5.10	0.63	3.98	0.00	4.31	0.69	5.01
5	Actual	3.15	1.35	3.85	1.65	3.85	1.65	3.15	1.35
	Predicted	3.39	1.00	4.20	1.48	3.84	1.17	3.46	1.08
6	Actual	4.81	0.69	3.94	0.56	3.94	0.56	4.81	0.69
	Predicted	5.05	0.43	3.63	0.85	3.39	0.77	5.14	0.42

Supplementary Table 128. Comparison between actual **PEA** values vs. LASSO + PLS prediction for both training and test sets

LASSO + PLS									
**Entry**		**(*R*)-PMP** (mM)	**(S)-PMP** (mM)	**(*R*)-PEA** (mM)	**(*S*)-PEA** (mM)	**(1*R*,2*S*)-PPA** (mM)	**(1*S*,2*R*)-PPA** (mM)	**(*R*)-PGL** (mM)	**(*S*)-PGL** (mM)
1	Actual	4.13	1.38	3.38	1.13	3.38	1.13	4.13	1.38
	Predicted	4.09	0.99	3.08	1.24	3.26	1.25	4.09	0.99
2	Actual	3.06	0.44	5.69	0.81	5.69	0.81	3.06	0.44
	Predicted	3.06	0.00	6.36	0.29	6.28	0.30	3.06	0.00
3	Actual	2.81	4.69	0.94	1.56	0.94	1.56	2.81	4.69
	Predicted	2.71	3.80	1.05	1.94	1.03	1.96	2.71	3.80
4	Actual	0.69	4.81	0.56	3.94	0.56	3.94	0.69	4.81
	Predicted	0.66	5.50	0.70	4.11	0.73	4.06	0.66	5.50
5	Actual	3.15	1.35	3.85	1.65	3.85	1.65	3.15	1.35
	Predicted	3.39	1.38	4.19	1.39	4.10	1.40	3.39	1.38
6	Actual	4.81	0.69	3.94	0.56	3.94	0.56	4.81	0.69
	Predicted	5.06	0.65	3.68	0.77	3.66	0.76	5.06	0.64

Supplementary Definitions and Equations

$$ee_{\mathbf{PMP}} = 9.7978\ \mathrm{mdeg}_{400\ \mathrm{nm}} - 0.1383 \qquad \text{(Supplementary Equation 1)}$$

$$ee_{\mathbf{PEA}} = -9.80599\ \mathrm{mdeg}_{340\ \mathrm{nm}} + 1.711244 \qquad \text{(Supplementary Equation 2)}$$

Principal Component Analysis

Principal component analysis (PCA) is an unsupervised learning method often used to reduce the dimension of data. The original matrix is represented by a product of two matrices:

$$X = TL^T \qquad \text{(Supplementary Equation 3)}$$

X is the original matrix with n rows and p columns, T is the scores matrix with n rows and d columns (where d also corresponds to the number of principal components (PC)), L is the loading matrix with d columns and p rows. In this project, singular value decomposition (SVD) is used for PCA. The SVD of an m x n matrix A can be defined by the following[1]:

$$A = U\mathbf{\Sigma}V^T \qquad \text{(Supplementary Equation 4)}$$

U and V are orthogonal matrices where U is the Eigenvector of A^TA and V^T is the Eigenvector of AA^T. Eigenvector is defined as the vector associated to a linear transformation that changes by a scalar factor when a linear transformation is applied. This linear transformation can be expressed by the following equation:

$$Av = \lambda v \qquad \text{(Supplementary Equation 5)}$$

Where A is a matrix, v is the Eigenvector of matrix A, and λ is the scalar Eigenvalue of matrix A. The Eigenvalue can be described as the amount of which the Eigenvector is scaled. The square roots of the eigenvalues associated are contained in $\mathbf{\Sigma}$, which are diagonal and have singular values.

Reconstruction of spectra is possible through inverse transformation of the principal component score. This can be achieved with

$$X' = TL^T + \mu \qquad \text{(Supplementary Equation 6)}$$

Where X' is the original space, T is the scores matrix, L^T is the loading matrix and μ is the mean vector.

A useful metric in PCA is the explained variance ratio, which refers to the percentage of variance the data has that can be explained at each principal component. It is the ratio between the variance of the principal component and total variance. Since PCA aims to maximize the variance of the data, the first principal component will explain the most variance.

Principal Component Regression

Principal component regression (PCR) is a supervised learning method that can be considered a combination of principal component analysis (PCA) and ordinary least-squares (OLS). It involves the decomposition of an X matrix (independent variable) into two orthonormal matrices that correspond to the score (U) and loading matrices (V) and are joined by diagonal matrix $\boldsymbol{\Sigma}$

$$S = U\boldsymbol{\Sigma}V^T \qquad \text{(Supplementary Equation 7)}$$

The regression coefficient b can be calculated through the formation of the pseudo-inverse matrix X^+

$$X^+ = V(\text{diag}(1/w_j)U^T) \qquad \text{(Supplementary Equation 8)}$$

$$b = X^+y \qquad \text{(Supplementary Equation 9)}$$

The pseudo-inverse matrix is equivalent to the generalized inverse from OLS:

$$X^+ = (X^TX)^{-1}X^T \qquad \text{(Supplementary Equation 10)}$$

The above equations can be used to calculate the regression coefficient b.

Least Absolute Shrinkage and Selection Operator (LASSO)

LASSO is an extension of ordinary least squares where the absolute value of the coefficient is added to the residual sum of squares for regularization. It can reduce the coefficient of some independent variables that are irrelevant to 0, which is useful for variable selection.

$$\sum_{i=1}^{n}\left(y_i - \beta_0 - \sum_{j=1}^{p}\beta_j x_{ij}\right)^2 + \lambda\sum_{j=1}^{p}|\beta_j| = RSS + \lambda\sum_{j=1}^{p}|\beta_j| \qquad \text{(Supplementary Equation 11)}$$

Where RSS is the residual sum of squares, $\sum_{j=1}^{p}|\beta_j|$ is the L1 penalty, and λ is the penalty term that tunes the amount of the penalty. The smaller the penalty term is, the closer the result is to the OLS estimates. The parameter λ can be chosen through cross validation.

Multiblock Principal Component Analysis

Multiblock principal component analysis (MBPCA) is an extension of PCA that allows extraction of global components and the contribution of each block in a multi-block data. If **X** is comprised of b blocks, therefore:

$$X = [T_1 \cdot P_1 T_2 \cdot P_2 \ldots T_b \cdot P_b] + \varepsilon \qquad \text{(Supplementary Equation 12)}$$

Where T_b is the corresponding block score for each block and P_b is the corresponding block loading.

$$T_{sup} = \sum_{i=1}^{b} w_i \cdot T_i \qquad \text{(Supplementary Equation 13)}$$

Where T_{sup} is the super score that contains the global trend of the data, T_i is the block score, and w_i is the block weight.

$$X_i = T_i P_i + \varepsilon \qquad \text{(Supplementary Equation 14)}$$

There are multiple versions of MBPCA available. In this study, consensus-PCA (CPCA) is used. A detailed algorithm of CPCA is available in the literature[2]

Multiblock-Partial least squares

Partial least squares (PLS) is a supervised learning method that decomposes both **X** (independent variable) and Y (dependent variable) matrices as a product of a common set of orthogonal factors and a set of specific loadings. It is often used in datasets with large multicollinearity which are often observed in analytical fields such as chemometrics and process control. Equation 15 and 16 show the underlying model of PLS:

$$X= TP^T + E \qquad \text{(Supplementary Equation 15)}$$

$$Y= UQ^T + F \qquad \text{(Supplementary Equation 16)}$$

Where X is the independent variable matrix, T is the X score matrix that contains the projections of X, P is the orthogonal loading matrix for X, Y is the dependent variable matrix, U is the Y score matrix that contains the projection of Y, Q is the orthogonal loading matrix for Y, and E and F are the error terms.

Multiblock partial least squares (MBPLS) is an extension of ordinary PLS and involves several data blocks (X_1, X_2, X_3,…, X_k) that may be independent feature spaces and treated in a parallel mode. Regression is done from each data block X_k with the U score matrix to give each block variable weights (w_k). Next, the block score t_b is obtained and combined into super block (S):

$$S= [t_{k1}, t_{k2}, \ldots, t_{kn}] \qquad \text{(Supplementary Equation 17)}$$

The super-weights (W_t) and super-scores (T_t) can be obtained through super block S through the following equation:

$$W_t= SU/U^TU= [W_{t1}, W_{t2}, \ldots, W_{tn}] \qquad \text{(Supplementary Equation 18)}$$

$$T_t= SW_t/W_t^TW_t \qquad \text{(Supplementary Equation 19)}$$

This method gives additional interpretability than ordinary PLS.

Cross-validation

In cross-validation, one or more samples are randomly left out and are used as “test set”, meanwhile the other samples are used as “training set.” This procedure is done until all samples are left out. Cross-validation is useful to see how well the algorithm can perform on a new data set. For the octonary data set, leave-one-out cross-validation, where a sample is left out each time is used because the number of training samples is relatively low.

Supplementary References

1 Dongarra, J.; Gates, M.; Haidar, A.; Kurzak, J.; Luszczek, P.; Tomov, S.; Yamazaki, I. The singular value decomposition: Anatomy of optimizing an algorithm for extreme scale. *SIAM Rev.* **60**, 808–865 (2018).

2 Westerhuis, J. A.; Kourti, T.; MacGregor, J. F. Analysis of multiblock and hierarchical PCA and PLS models. *J. Chemom.* **12**, 301-321 (1998).
